# Organocatalytic Enantioselective Synthesis of Bicyclo[2.2.2]octenones via Oxaziridinium Catalysed *ortho*‐Hydroxylative Phenol Dearomatization**

**DOI:** 10.1002/anie.202205278

**Published:** 2022-06-10

**Authors:** Tom D. D'Arcy, Mark R. J. Elsegood, Benjamin R. Buckley

**Affiliations:** ^1^ Department of Chemistry Loughborough University Loughborough, Leicestershire LE11 3TU UK

**Keywords:** Enantioselective, Hydroxylative Dearomatization, Natural Products, Organocatalysis, Oxaziridine

## Abstract

Hydroxylative dearomatization reactions of phenols (HPD) offer an efficient way to assemble complex, biologically relevant scaffolds. Despite this, enantioselective hydroxylative phenol dearomatizations for the construction of bicyclo[2.2.2]octenones are classically limited to stoichiometric chiral reagents, and a practical, enantioselective catalytic method has remained elusive. Herein, we describe a highly enantioselective, organocatalytic tandem o‐HPD‐[4+2] reaction. Our methodology utilizes a chiral oxaziridinium organocatalyst, that is available in both enantiomeric forms, to afford dearomatized products in high enantioselectivity over a range of phenol substitution patterns. This approach was applied to the highly enantioselective synthesis of (+)‐biscarvacrol (99 : 1 e.r.) and (−)‐bis(2,6‐xylenol) (94 : 6 e.r.). The practicality of our conditions was demonstrated at gram‐scale, using an amine precatalyst, accessible in a single synthetic step.

## Graphical abstract

An enantioselective, oxaziridinium‐catalysed *ortho*‐hydroxylative dearomatization‐[4+2] dimerization reaction of phenols is described. This classically challenging transformation was successfully employed in the synthesis of bis(monoterpenoid) (+)‐biscarvacrol and bacterial metabolite (−)‐bis(2,6‐xylenol), with high enantioselectivity. Our simple, organocatalytic system tolerates multiple phenol substitution patterns and *ortho* substituents.

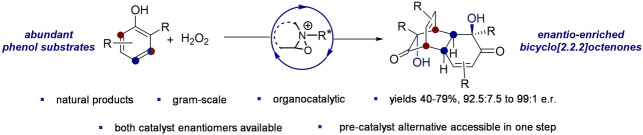

The asymmetric dearomatization of phenols offers a valuable method towards generating biologically relevant target molecules,^[1]^ owing to the high abundance of feedstock phenolic compounds. Hydroxylative phenol dearomatization (HPD)^[2a]^ of *o*‐alkylphenols often leads to [4+2]‐dimerization of the intermediate *o*‐quinol, thus remarkably generating decorated bicyclo[2.2.2]octenones in a single synthetic step (Figure 1a).[^2^, ^3^, ^4^, ^5^] The dimerized *o*‐quinols feature the core of several natural products, for example, the anti‐pancreatic cancer compound grandifloracin,^[6]^ bis(sesquiterpenoid) aquaticol,^[7]^ and the bis(monoterpenoid) biscarvacrol,^[8]^ as well as the bacterial metabolite, bis(2,6‐xylenol)^[9]^ (Figure 1b). Therefore, methods to access such products bear noteworthy importance. The biological significance of non‐natural analogues of such products has also been described,^[10]^ further highlighting the demand for a general enantioselective *o*‐hydroxylative phenol dearomatization method. However, previous efforts towards the enantioselective *o*‐HPD‐[4+2] reaction are limited to stoichiometric chiral reagents, and to the best of our knowledge, a general, practical, catalytic method is yet to be reported. Despite this, efforts towards catalytic enantioselective HPD reactions of naphthols,^[4]^ and resorcinols,^[5]^ have recently been reported.

**Figure 1:**
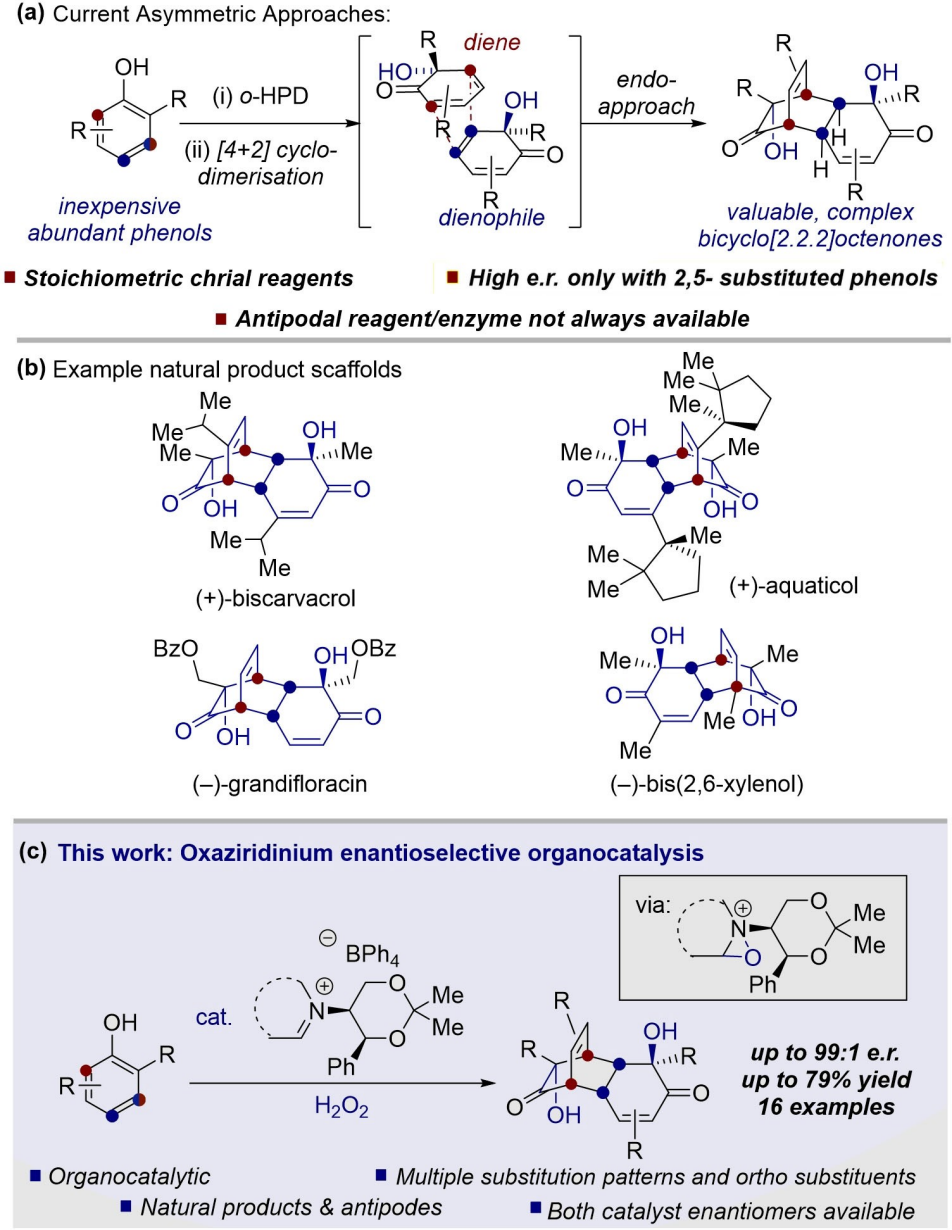
a) *o*‐HPD‐[4+2] dimerization concept and current limitations. b) Representative natural products from dimerized *o*‐quinols. c) Our organocatalytic enantioselective method.

A pioneering example of enantioselective *o*‐HPD‐[4+2] reactions was reported by Porco, which used a copper‐sparteine complex.^[11]^ The reactions required stoichiometric Cu^I^ and (−)‐sparteine, and the scope was limited; only 2,5‐substitution of the phenol was tolerated, where a methyl group was the only successful *ortho*‐substituent. More recently, chiral hypervalent iodine reagents have been utilized for the *o*‐HPD‐[4+2] reaction.

Birman and co‐workers reported an *o*‐oxazoline derived iodine(V) reagent, which could invoke the bicyclo[2.2.2]octenone synthesis,^[12]^ however only moderate enantioselectivity (up to 88.5 : 12.5 e.r.) was achieved. Pouységu, Quideau and co‐workers then published the use of an axially chiral bis‐iodine(V) reagent, which afforded the reaction with selectivities ranging from 70 : 30 e.r. up to 97 : 3 e.r., offering only moderate enantioselectivity for most substrates.^[13]^

With a general, practical, catalytic method for the enantioselective *o*‐HPD‐[4+2] reaction still yet to be discovered, we postulated that the dearomatization could be invoked by a catalytically generated electrophilic oxygen atom source, in the form of an oxaziridinium cation. We speculated that suitable conditions would therefore allow facile access to enantioenriched natural and non‐natural *o*‐quinol dimers (Figure 1c). Since oxaziridinium organocatalysis is classically employed in epoxidation reactions,^[14]^ our strategy additionally aimed to establish the field of oxaziridinium‐catalysed dearomatization.

At the onset of our investigations, we wished to understand whether the hypothesized oxaziridinium‐mediated hydroxylative dearomatization could occur. To achieve this, we initially studied the dearomatization of our model substrate, 2,6‐dimethylphenol (**1 a**), with achiral catalyst **2**, in order to synthesize (±)‐bis‐(2,6‐xylenol) **3 a** (Scheme 1). After optimizing solvent, oxidant stoichiometry, and choice of base (see Table S1), **(±)**‐**3 a** was afforded in 65 % yield, serving as promising proof‐of‐concept. The use of MeCN as a H_2_O_2_ activator was pivotal for reactivity.^[15]^

**Scheme 1:**
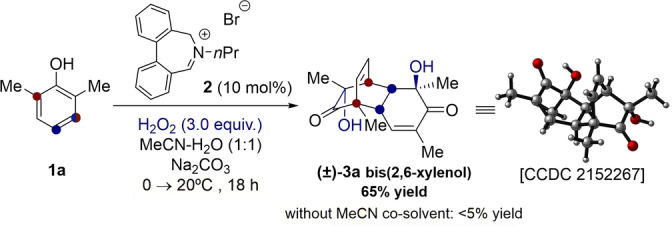
Racemic synthesis of (±)‐bis(2,6‐xylenol) using an achiral catalyst.

With preliminary optimization achieved using the arbitrary achiral catalyst, we turned our attention to the development of the enantioselective variant of the reaction. Again, 2,6‐dimethylphenol (**1 a**) was selected as the model substrate, since symmetrical phenols are particularly challenging for the aforementioned enantioselective methods in the literature. We investigated the oxaziridinium catalysts originally developed by Page and co‐workers for asymmetric epoxidation, derived from a chiral (*S*,*S*)‐(+)‐acetonamine.^[16]^

When biphenylazepinium **4 a**, which is directly analogous to catalyst **2**, was employed, (+)‐bis‐(2,6‐xylenol) (**(+)‐3 a**) was afforded with a promising 79 : 21 e.r. and in 86 % yield (Table 1, entry 1). Changing the catalyst backbone to a dihydroisoquinoline (**5**) caused a significant decrease in selectivity (60 : 40 e.r., Table 1, Entry 2). Alteration of the electronics of the catalyst was also investigated, by means of catalyst **4 b**,^[17]^ which had no effect on the observed enantioselectivity (Table 1, Entry 4).

**Table 1:**
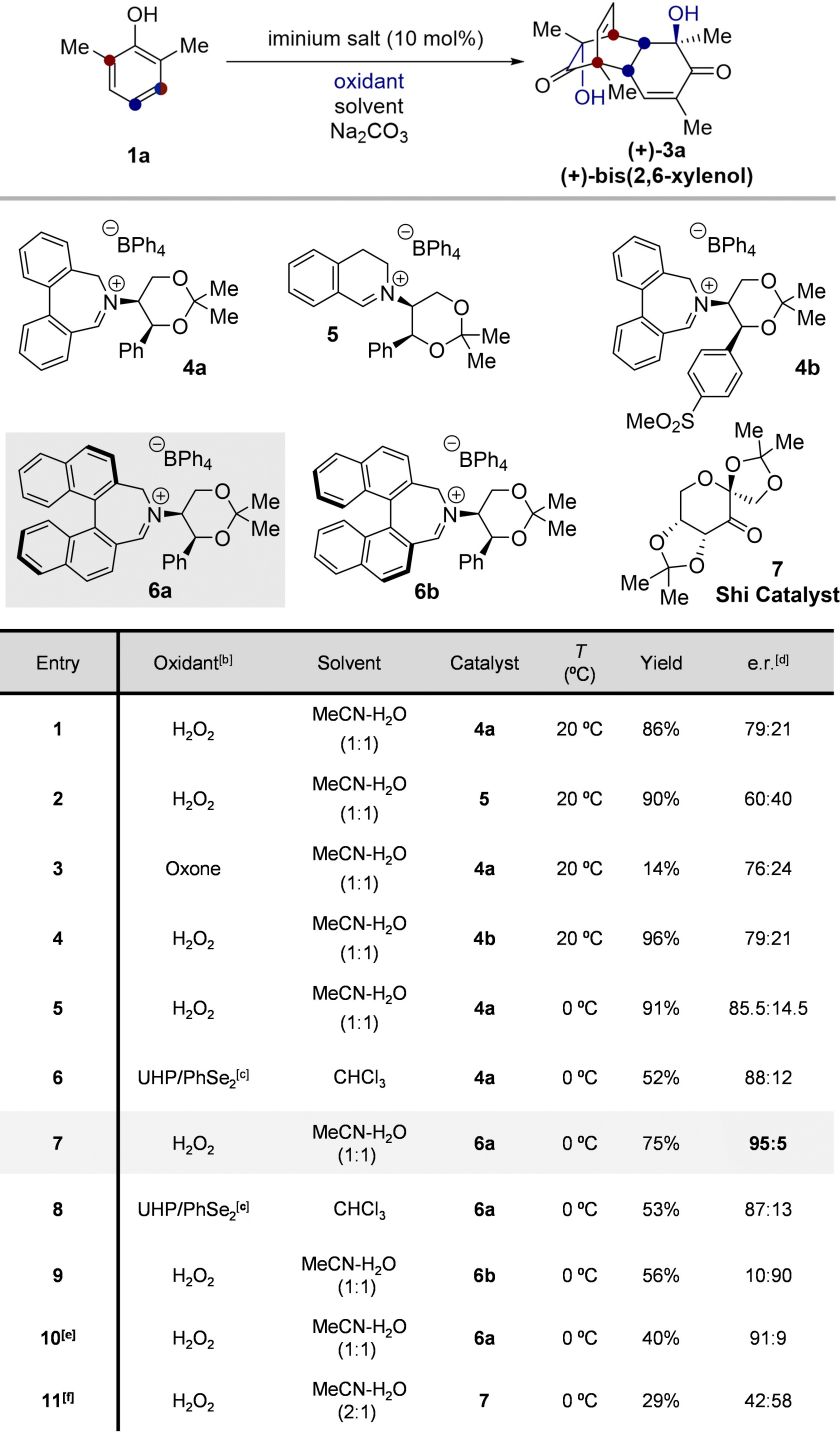
Optimization summary of the catalytic enantioselective *o*‐HPD‐[4+2] reaction.^[a]^

[a] Reactions performed on a 0.4 mmol scale. [b] 3.0 equiv. [c] 5 mol % PhSe_2_, 3.0 equiv UHP. [d] Determined by chiral stationary phase HPLC. [e] Reaction buffered to pH 10. [f] Original conditions reported by Shi (see ref. [15]): substrate (0.5 mmol), MeCN (1 mL), 0.5 mL 1.0 M K_2_CO_3_ in 0.4 mM EDTA, H_2_O_2_ (1.5 mmol), 30 mol % Shi catalyst **7**.

The reaction could also be facilitated using a non‐aqueous, dual‐catalytic system,^[18]^ using PhSe_2_, UHP (urea hydrogen peroxide) and an iminium catalyst (Table 1, Entry 6). This offered a small increase in selectivity relative to the H_2_O_2_‐MeCN system, albeit with reduced yield (88 : 12 e.r., 52 % yield). With various conditions explored for the biphenylazepinium catalyst **4 a**, we turned to binaphthylazepinium catalyst **6 a**.

Since a clear influence of the catalyst backbone was observed (**4 a** vs. **5**, Table 1, Entries 1 and 2), we anticipated that the larger, rigid binaphthyl backbone could increase the enantioselectivity of the reaction. Pleasingly, it was found that the *o*‐HPD‐[4+2] reaction could be achieved with 95 : 5 e.r., and 75 % yield using the MeCN‐H_2_O_2_ system with catalyst **6 a** (Table 1, Entry 7). Attempts to further increase selectivity with a catalyst that features an increased biaryl dihedral angle (Table S2),^[19]^ were unsuccessful. The diastereomeric catalyst (**6 b**) reversed the enantioselectivity, affording (−)‐bis‐(2,6‐xylenol) in 56 % yield and 10 : 90 e.r. (Table 1, Entry 9).

Reducing the pH offered no further improvement in selectivity and diminished the yield. For comparison with other oxygen‐transfer organocatalysts, we found that the Shi catalyst **7** affords a low yield of product, with almost no enantiocontrol (Table 1, Entry 11).

Using our optimized enantioselective conditions for the *o*‐HPD‐[4+2] reaction, we evaluated the performance of alternative phenol substrates (Table 2). 2,4,6‐Trimethylphenol, another highly symmetrical substrate, was successful (**3 b**) with similarly high enantioselectivity and yield. Larger *ortho‐* substituents on symmetrical phenols are also compatible, as shown by the reaction of 2,6‐diethylphenol, which provided **3 c** (79 % yield, 98 : 2 e.r.). 2,3,6‐Substituted phenols also readily reacted under the reaction conditions, showing selective dearomatization at the less‐hindered 6‐position (**3 d**). Benzyl groups were also tolerated as *ortho*‐substituents, as shown by product **3 e**. Electron‐donating (−Me) and electron‐withdrawing (−F) substituents on the 6‐benzyl group afforded **3 f** and **3 g** respectively. The absolute structure and configuration of **3 g** was confirmed through X‐ray diffraction. Our methodology could also furnish the reaction on substrates with only a single *ortho*‐subsituent from 2,5‐substituted phenols, as depicted in examples **3 h**–**3 k**. This allowed the synthesis of the natural diterpenoid (+)‐biscarvacrol **(+)‐3 i** (61 % yield, 99 : 1 e.r.). Thymol, a substrate with a sterically demanding isopropyl substitutent at the *ortho*‐position, afforded **3 h** with 99 : 1 e.r. and 69 % yield. Non‐symmetrical 2,6‐substituted phenols were also successful in the reaction. For example, 2‐methyl‐6‐*tert*‐butyl phenol was converted into bicyclo[2.2.2]octenone **3 l**, by selective dearomatization at the 2‐position. However, dearomatization at both the 2‐ and 6‐ position occurred with 2‐benzyl‐6‐methylphenol, giving rise to homo‐dimer **3 m** and hetero‐dimer **3 n** in a 1 : 1 ratio.

**Table 2:**
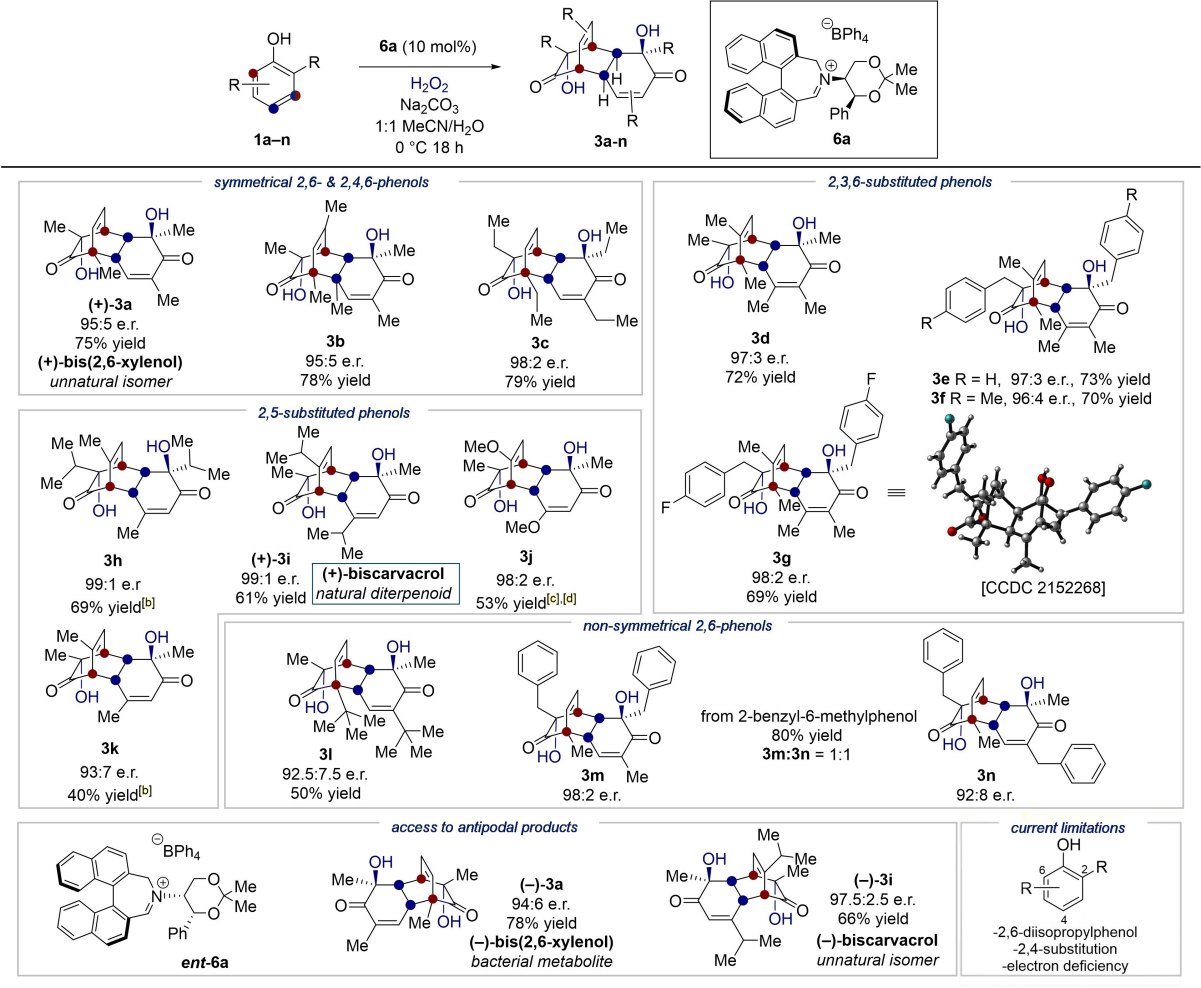
Reaction scope.^[a]^

[a] Reactions ran at 0.26–0.41 mmol scale. Enantioselectivities determined by chiral stationary phase HPLC. [b] Performed at room temp. (20 °C). [c] Performed using tertiary amine precatalyst **8** (*see* Figure 2a). [d] Crude mixture heated at 70 °C for 1 h to allow for the [4+2] cycloaddition.

Using oppositely configured catalyst ***ent*** **‐6 a**, the natural isomer (−)‐bis(2,6‐xylenol), as well as **(−)‐3 i** were able to be prepared, demonstrating access to antipodal bicyclo[2.2.2]octenone products. Unsuccessful substrates include 2,4‐substituted phenols, as well as highly hindered substrates such as 2,6‐diisopropyl phenol.^[20]^

To further highlight the utility of our methodology, we were able to perform the synthesis of (+)‐bis‐(2,6‐xylenol) (**(+)‐3 a**), using amine **8** as a precatalyst, wherein the amine is oxidized under the reaction conditions to form the active iminium ion.^[21]^ The dearomatization of 2,6‐dimethylphenol **1 a** was successful when the amine precatalyst **8** was employed, with near‐identical yield and enantioselectivity to the parent iminium **6 a** (75 % yield, 94.5 : 5.5 e.r., Figure 2a).

**Figure 2:**
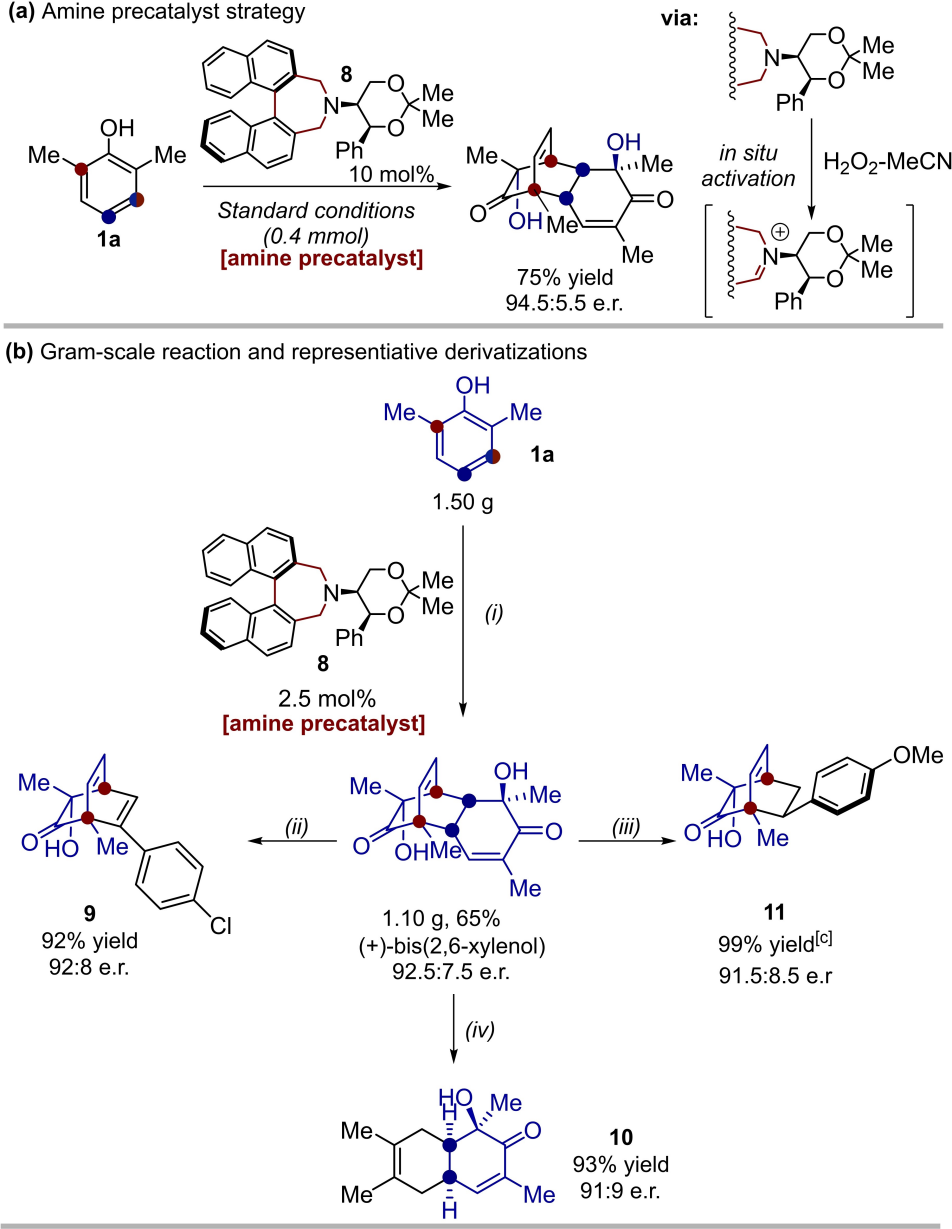
a) Use of amine **8** as a precatalyst in the dearomatization reaction. b) Gram‐scale preparation of (+)‐bis(2,6‐xylenol) using amine **8**, with reduced loading, and subsequent retro‐[4+2]‐[4+2] transformations. c) ca. 10 : 1 regiomeric ratio. i) aq. H_2_O_2_ (3.0 equiv), 1 : 1 MeCN‐H_2_O, 2.5 mol % **8**, Na_2_CO_3_ (5 equiv), 0 °C, 18 h. ii) 15 equiv 4‐chlorophenylacetylene, μW 140 °C, 3.5 h. iii) 10 equiv 4‐vinylanisole, μW 130 °C, 2 h. iv) 15 equiv 2,3‐dimethyl butadiene, μW 130 °C, 4 h.

This approach was also employed in a gram‐scale reaction, with reduced catalyst loading (2.5 mol %).

One of the most useful transformations of the described bicyclo[2.2.2]octenones are retro‐[4+2]‐[4+2] reactions.^[22]^ Following our gram‐scale synthesis, (+)‐bis(2,6‐xylenol) was derivatized using retro‐[4+2]‐[4+2] reactions (Figure 2b), using a modified method to that reported by Porco.^[23]^ A terminal alkyne, as well as a terminal alkene successfully behaved as dienophile partners to form compounds **9** and **11** respectively. 1,3‐Dimethyl butadiene engaged in the retro‐[4+2]‐[4+2] reaction leading to the *cis*‐decalin framework **10**. All the described reactions proceeded with excellent retention of enantiopurity. These divergent derivatisations highlight rapid access to further diverse, enantioenriched scaffolds.

To confirm the mechanism of our dearomatization reaction, we sought to provide evidence of the active oxaziridinium ion. Due to the inherent instability of the species in question, we employed a direct HRMS injection of the iminium catalyst **6 a** after exposure to oxone, which proved fruitful in observing oxaziridinium cation **12** (Figure 3a). Oxaziridinium tetrafluoroborate^[24]^ **14** is more stable than **12**. Therefore, **14** was able to be employed in a stoichiometric reaction with phenol **1 a**, furnishing **(±)‐3 a** in 37 % yield (Figure 3c). This demonstrated the ability of the oxaziridinium, as a structural motif, to perform the hydroxylative dearomatization.

**Figure 3:**
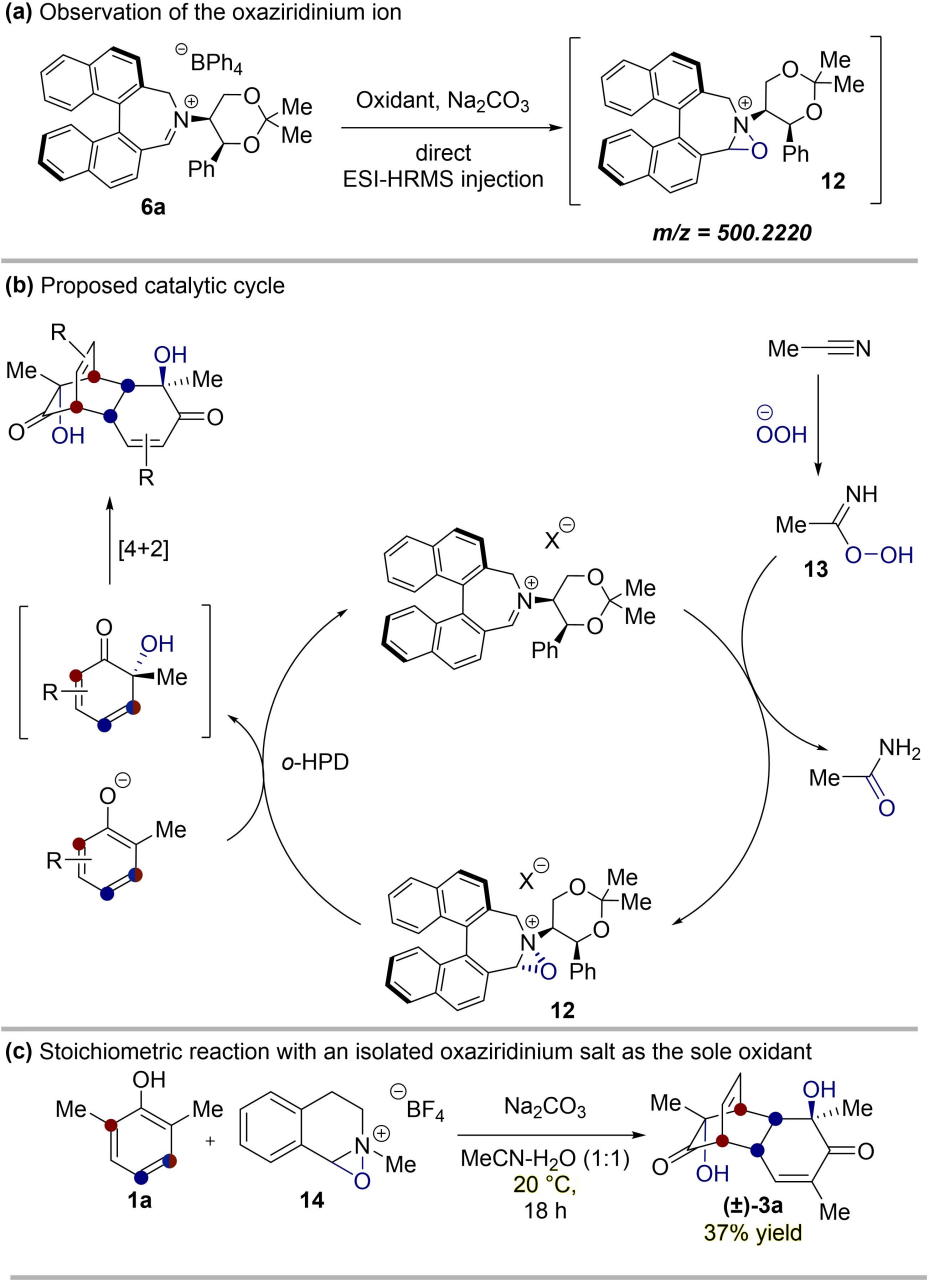
a) Observation of the reactive oxaziridinium ion by direct HRMS. b) Proposed catalytic cycle. c) Stoichiometric dearomatization with oxaziridinium **14**.

With these findings, we can propose a mechanism as shown in Figure 3b, in which H_2_O_2_ is activated by reacting with MeCN, in an analogous manner to the Payne oxidation.^[15]^ The intermediate peroxyimidic acid **13** can attack the iminium catalyst **6 a**, to form oxaziridinium **12**. This oxaziridinium formation is thought to be diastereoselective.^[25]^ Nucleophilic attack on the oxaziridinium by the phenolate gives rise to the *o*‐quinol, in the enantiodetermining step of the reaction. The *o*‐quinol then dimerizes in a regio‐ and diastereoselective manner,^[26]^ giving rise to the bicyclo[2.2.2]octenone product.

In summary, we have developed an organocatalytic, highly enantioselective method for *o*‐hydroxylative phenol dearomatization‐[4+2] reactions. Multiple phenol substitution patterns were compatible with our methodology, which resolves the limitation of previous literature methods that can only afford high enantioselectivities with 2,5‐substituted phenols. We applied our chemistry to natural products (+)‐biscarvacrol, and (−)‐bis‐(2,6‐xylenol). We demonstrated the practicality of our conditions by the use of a simpler, amine precatalyst alternative, which can be synthesized in one step from commercial materials. Several retro‐[4+2]‐[4+2] reactions were performed on (+)‐bis(2,6‐xylenol), to highlight that the described bicyclo[2.2.2]octenones can be rapidly diversified into alternative enantioenriched scaffolds, in a divergent manner. It is envisioned that the reported dearomatization methodology offers a viable tool when studying biologically active *o*‐quinol dimers. We also hope this report establishes a new use of oxaziridinium organocatalysts in dearomative chemistry.

## Conflict of interest

The authors declare no conflict of interest.

## Supporting information

As a service to our authors and readers, this journal provides supporting information supplied by the authors. Such materials are peer reviewed and may be re‐organized for online delivery, but are not copy‐edited or typeset. Technical support issues arising from supporting information (other than missing files) should be addressed to the authors.

Supporting Information

## Data Availability

The data that support the findings of this study are available in the Supporting Information of this article.
