# Supplementary material for: Organocatalytic Enantioselective Synthesis of Bicyclo[2.2.2]octenones via Oxaziridinium Catalysed ortho‐Hydroxylative Phenol Dearomatization
Source: Angew Chem Int Ed Engl. 2022 Jun 10;61(30):e202205278. doi: 10.1002/anie.202205278 (PMC9401573; doi:10.1002/anie.202205278)
Supplement: Supplementary file 1 — Supporting Information [file ANIE-61-0-s001.pdf]

Angewandte Chemie
Eine Zeitschrift der Gesellschaft Deutscher Chemiker

Supporting Information

Organocatalytic Enantioselective Synthesis of Bicyclo[2.2.2]octenones via Oxaziridinium Catalysed *ortho*-Hydroxylative Phenol Dearomatization

*T. D. D'Arcy, M. R. J. Elsegood, B. R. Buckley**

Table of Contents

General……………………………………………………………………………………………….. 2

Catalyst synthesis …………………………………………………………………………………... 3

Substrate synthesis …………………………………………………………………………………11

Extended optimisation of the enantioselective phenol dearomatization reaction …………………….. 14

General procedure for the enantioselective phenol dearomatization …………………………...…… 17

Mechanistic studies……………………………………………………………………………… 41

X-ray Crystallography………………………………………………………………………………45

NMR spectra ……………………………………………………………………………………….. 50

References …………………………………………………………………………………………101

General

All reagents and solvents were supplied from Sigma-Aldrich, Fisher Scientific, Alfa Aesar or Fluorochem and were used without further purification. Compound purification by flash column chromatography was performed with Apollo Scientific 40-63 μm silica gel, or using automated flash chromatography (Teledyne ISCO CombiFlash), where crude mixtures were dry loaded.Melting points were measured in open capillary tubes using a Stuart scientific SMP3 melting point apparatus and are uncorrected. Thin layer chromatography (TLC) was performed using Merck Kieselgel 60 F254 plates and visualisation was achieved under UV as well as staining with $KMnO_4$ or vanillin. Infrared spectra (IR) were recorded using a Perkin-Elmer Spectrum 65 FT-IR spectrophotometer; samples were prepared as KBr discs. High Resolution Mass spectrometry (HRMS) was performed using a Thermo Scientific Exactive Orbitrap mass spectrometer. High performance liquid chromatography on a chiral stationary phase was performed in a Waters 2695 chromatograph coupled to a Waters 2998 photodiode array detector.

NMR spectra were recorded at 298 K using a Jeol ECZ 400 or 500 MHz spectrometer. ^{1}H, ^{13}C and ^{19}F spectra were recorded at 400/500 MHz, 101/126 MHz and 376 MHz respectively. Chemical shifts (δ) are reported in parts per million (ppm) relative to residual $CDCl_3$ or d_4-MeOH, and J values are given in Hertz (Hz). Abbreviations for multiplets are singlet (s), broad singlet (br s), doublet (d), triplet (t), quartet (q), doublet of doublets (dd), doublet of triplets (td), heptet (hept), multiplet (m).

The chiral (*S*,*S*)-(+)-acetonamine, (+)-(4*S*,5*S*)-2,2-Dimethyl-4-phenyl-1,3-dioxan-5-amine, was prepared according to Page.[1b] Both the binaphthyl di-bromide and (*S*,*S*)-(+)-acetonamine are also available from commercial sources.

Catalyst Synthesis

2-(2-bromoethyl)-benzaldehyde[1a] (S1)

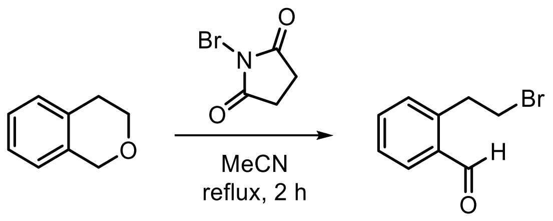

N-bromosuccinimide (3.18 g, 17.9 mmol) was added to a stirred solution of isochroman (1.87 mL, 14.9 mmol) in MeCN (15 mL) at r.t. The resulting suspension was then gradually heated to reflux for 2 h, before removing solvent *in vacuo*. EtOAc (ca. 15 mL) was then added, and the resulting suspension was filtered. The filtrate was then diluted with further EtOAc (100 mL), washed with 0.1 M NaOH (aq., 100 mL), followed by saturated brine solution (aq., 100 mL). The organic layer was then extracted, dried over $MgSO_4$, filtered, and concentrated *in vacuo*. The crude product was purified by flash column chromatography (0 → 5% v/v EtOAc in hexane) to yield the title compound as a pale-yellow oil (2.69 g, 85%). When not in use, samples were stored at -18 ºC under N_2.

TLC R_f: 0.5 (Hexane/EtOAc 10:1).
^{1}H NMR (400 MHz, $CDCl_3$): δ 10.16 (s, 1H), 7.83 (dd, *J* = 7.5, 1.5 Hz, 1H), 7.57 (td, *J* = 7.5, 1.6 Hz, 1H), 7.49 (td, *J* = 7.5, 1.3 Hz, 1H), 7.36 – 7.33 (m, 1H), 3.65 – 3.55 (m, 4H).
^{13}C NMR (101 MHz, $CDCl_3$): δ 193.05, 140.65, 134.63, 134.02, 133.84, 132.24, 127.80, 36.42, 32.88.

2,2'-biphenyldimethanol[2] (S2)

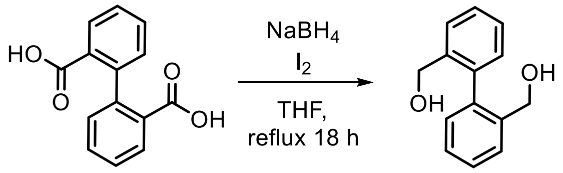

$NaBH_4$ (4.4 g, 115 mmol) was added to a stirred solution of diphenic acid (6.8 g, 28.0 mmol) in dry THF, (150 mL) at room temperature (20 ºC), under N_2. To the stirred suspension, a solution of I_2 (14.6 g, 57.5 mmol) in dry THF (70 mL) was added dropwise at 0 ºC *via* a pressure equalising dropping funnel, in approximately 20 min. The resulting mixture was then heated to reflux for 18 h, before cooling to 0 ºC, and carefully quenching with MeOH (200 mL). Solvent was then removed *in vacuo*, followed by the addition of 20% w/v KOH (150 mL). The resulting suspension was then stirred for 1 h, before extracting with CH_2Cl_2 (3 x 200 mL). The combined organic layers were then dried over $MgSO_4$, filtered and concentrated *in vacuo* to afford an off-white solid of 2,2'-biphenyldimethanol (6.0 g, >99%) which required no further purification.
^{1}H NMR (400 MHz, $CDCl_3$): δ 7.46 (dd, *J* = 7.5, 1.1 Hz, 2H), 7.37 (td, *J* = 7.4, 1.4 Hz, 2H), 7.32 (td, *J* = 7.4, 1.4 Hz, 2H), 7.13 (dd, *J* = 7.4, 1.2 Hz, 2H), 4.38 – 4.24 (m, 2H), 3.30 (br s, 2H).
^{13}C NMR (101 MHz, $CDCl_3$): δ 140.11, 138.74, 129.76, 129.70, 128.19, 127.75, 62.83.
LCMS (ESI$^+$): *m/z* calcd. for $C_{14}H_{14}O_2$ 237.1; found 236.9 [M+Na].

5,7-dihydrodibenzo[c,e]oxepine[3] (S3)

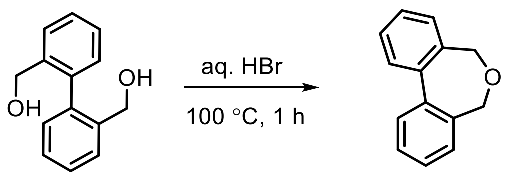

2,2'-biphenyldimethanol (5.99 g, 28.0 mmol) was added to HBr (43 mL, 48% aq. solution) in H_2O (43 mL) at r.t., before heating to reflux for 1 h. The reaction mixture was then cooled to r.t., before diluting with Et_2O (200 mL). The aqueous layer was then separated, and washed with Et_2O (3 x 200 mL). The combined organics were then washed with sat. $NaHCO_3$ (150 mL) followed by sat. brine solution (150 mL). The organics were then dried over $MgSO_4$, filtered and concentrated *in vacuo* to afford a pale-yellow oil, which crystallised upon standing. The crude solid was then recrystallised from hexanes to afford the oxepine as an off-white solid (4.83 g, 88%).

mp: 63-65 ºC.

^{1}H NMR (400 MHz, $CDCl_3$): δ 7.57 (d, *J* = 7.3 Hz, 2H), 7.55 – 7.48 (m, 2H), 7.47 – 7.40 (m, 4H), 4.37 (s, 4H).

^{13}C NMR (126 MHz, $CDCl_3$): δ 141.35, 135.30, 129.84, 129.07, 128.40, 127.62, 67.68.

LCMS (ESI$^+$): *m/z* calcd. for $C_{14}H_{13}O$ 197.1; found 197.0 [M+H].

2-[2-(bromomethyl)phenyl]benzene carbaldehyde[3] (S4)

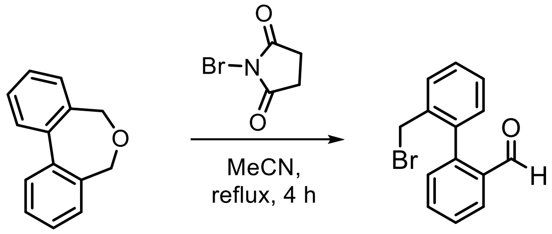

NBS (5.29 g, 29.7 mmol) was added to a stirred solution of 5,7-dihydrodibenzo[c,e]oxepine (4.49 g, 22.9 mmol) in MeCN (90 mL). The resulting suspension was then heated to reflux for 4 h. The reaction was then cooled to r.t., before removing solvent *in vacuo*. The mixture was then suspended in EtOAc (*ca.* 25 mL) before filtering under suction. The filtrate was then diluted with further EtOAc (75 mL) and was washed with 0.1 M NaOH (100 mL), followed by sat. brine solution (100 mL). The organic layer was then extracted, dried over $MgSO_4$, filtered, and then concentrated *in vacuo* to afford an orange oil. The crude product was then purified by flash column chromatography (0 → 6% EtOAc in hexane) to furnish the title compound as a white solid (3.26 g, 52%).

mp: 62-63 ºC.

^{1}H NMR (400 MHz, $CDCl_3$): δ 9.74 (d, *J* = 0.8 Hz, 1H), 8.07 (dd, *J* = 7.8, 1.2 Hz, 1H), 7.67 (td, *J* = 7.5, 1.4 Hz, 1H), 7.61 – 7.53 (m, 2H), 7.47 – 7.34 (m, 3H), 7.21 (dd, *J* = 7.5, 1.2 Hz, 1H), 4.30 (dd, *J* = 40.9, 10.1 Hz, 2H).

^{13}C NMR (126 MHz, $CDCl_3$): δ 191.77, 143.39, 137.97, 136.09, 134.27, 133.67, 131.16, 130.83, 130.79, 129.17, 128.65, 128.47, 127.77, 31.47.

HRMS (ESI$^+$): *m/z* calcd. for $C_{14}H_{11}BrONa$ 296.9886; found 296.9885 [M+Na].

9-propyl-9-azatricyclo[9.4.0.0^{2,7}]pentadeca-1(11),2,4,6,8,12,14-heptaen-9-ium bromide (2)

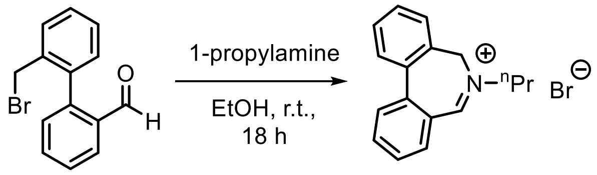

1-propylamine (0.25 mL, 3.04 mmol), was added dropwise to a stirred solution of 2-[2-(bromomethyl)phenyl]benzene carbaldehyde (1.00 g, 3.63 mmol) in EtOH (5 mL), at 0 ºC. The reaction was then warmed to r.t., and stirred for 18 h. A mixture of hexane-Et_2O was then added, causing the product to directly precipitate. The precipitate was filtered under suction, washing with further hexane and Et_2O, to afford biphenylazepinium **2** as a white solid (871 mg, 91%).

mp: 120-122 ºC.

IR v_{max} (cm^{-1}, KBr disc): 2976, 2962, 2875, 1662.

^{1}H NMR (400 MHz, CD_3OD): δ 9.33 (s, 1H), 8.08 (d, *J* = 7.9 Hz, 1H), 8.06 – 7.94 (m, 2H), 7.85 – 7.74 (m, 2H), 7.73 – 7.68 (m, 1H), 7.66 – 7.58 (m, 2H), 5.08 (s, 1H), 4.58 (s, 1H), 4.24 (s, 2H), 2.01 (s, 2H), 0.87 (t, *J* = 7.4 Hz, 3H).

^{13}C NMR (101 MHz, CD_3OD): δ 170.90, 143.07, 138.41, 137.06, 135.72, 135.66, 131.67, 131.53, 131.30, 130.88, 130.06, 129.88, 128.05, 65.63, 57.07, 22.57, 10.62.

HRMS (ESI$^+$): *m/z* calcd. for $C_{17}H_{18}N^+$ 236.1434; found 236.1434 [M+].

2,2-Dimethyl-4-phenyl-1,3-dioxan-5-amine

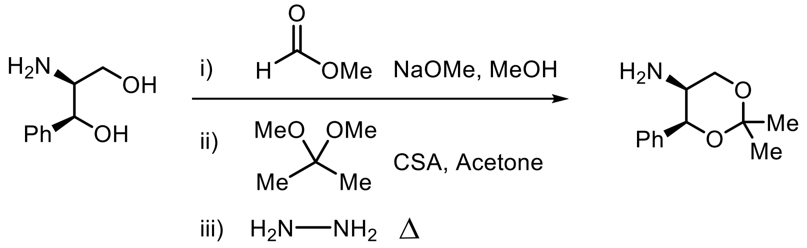

Methyl formate (0.2 mL, 3.3 mmol) and NaOMe (16 mg, 0.3 mmol) were added to a solution of the amino diol (500 mg, 3.0 mmol) in MeOH (5 mL). The resulting reaction mixture was then stirred for 3.5 h at r.t., before removing solvent *in vacuo*. The residue was then dissolved in acetone (25 mL), before camphorsulfonic acid (70 mg, 0.3 mmol), and 2,2-dimethoxypropane (3.7 mL, 30 mmol) were added. The reaction mixture was then stirred for 4 h, at r.t., before removing solvents *in vacuo*. The resulting oil was then redissolved in EtOAc (30 mL), before washing with sat. $NaHCO_3$ (30 mL). The organics were then dried over $MgSO_4$, filtered, and concentrated *in vacuo*. The formamide was dissolved in hydrazine hydrate (50-60%, 20 mL), and then heated to reflux for 3 h. After cooling to room temperature (20 ºC), the mixture was extracted with EtOAc (3 x 20 mL). The combined organic layers were then washed with H_2O (2 x 20 mL), dried over $MgSO_4$, filtered, and concentrated *in vacuo*. The product was afforded as a pale-yellow oil (561 mg, 90% yield over 3 steps) and was used without further purification.

^{1}H NMR (400 MHz, $CDCl_3$): δ 7.40 – 7.23 (m, 5H), 5.09 (d, *J* = 1.3 Hz, 1H), 4.29 (dd, *J* = 11.7, 2.3 Hz, 1H), 3.89 (dd, *J* = 11.7, 1.8 Hz, 1H), 2.74 (q, *J* = 2.0 Hz, 1H), 1.53 (2 x s, 6H), 1.30 (br s, 2H).
^{13}C NMR (101 MHz, $CDCl_3$): δ 139.67, 128.50, 127.47, 125.78, 99.24, 73.91, 66.20, 49.81, 29.84, 18.69.
LCMS (ESI, m/z): calcd. for $C_{12}H_{17}NO_2$ 208.1; found 207.9 $[M+H]^+$.

General Procedure A: Synthesis of Dihydroisoquinolinium and Biphenylazepinium Tetraphenylborate Salts 4a, 4b and 5

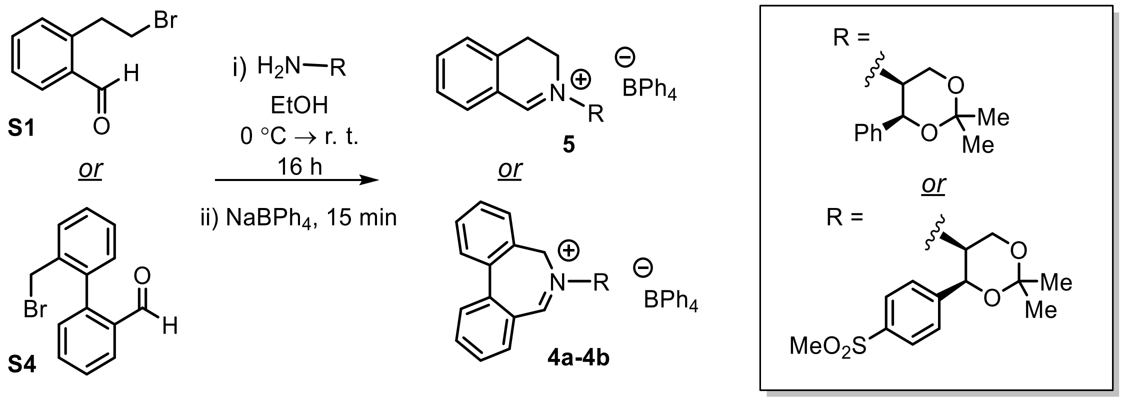

The (*S*,*S*)-acetonamine (1.0 eq.) is added slowly to a solution of 2-(2-bromoethyl)-benzaldehyde **S1** or 2-[2-(bromomethyl)phenyl]benzene carbaldehyde **S4** (1.1 eq,), in absolute EtOH (1 mL $mmol^{-1}$), at 0 ºC. The reaction mixture is then allowed to warm to room temperature (20 ºC) and was stirred for 16 h, before adding $NaBPh_4$ (1.1 eq.) in a minimal amount of MeCN. The resulting precipitate is then filtered under suction, and washed with cold EtOH, H_2O, and Et_2O to afford the pure dihydroisoquinolinium or biphenylazepinium salt. Characterization for catalysts **4a-5** was consistent with the literature.[1a,1b]

(*R*)-2'-(trifluoromethanesulfonyloxy)-[1,1'-binaphthalen]-2-yl trifluoromethanesulfonate[4,5] (S5)

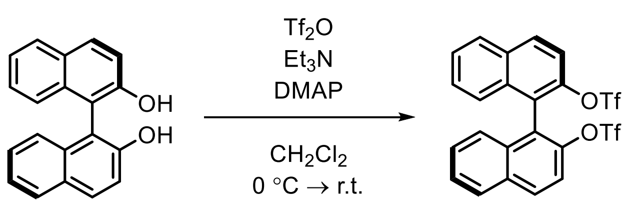

Tf_2O (8.8 mL, 52.5 mmol) was added dropwise, at 0 ºC, to a solution of (*R*)-BINOL (5.00 g, 17.5 mmol), DMAP (855 mg, 7.0 mmol) and Et_3N (7.28 mL, 52.5 mmol), in dry CH_2Cl_2 (125 mL). The resulting reaction mixture was then stirred at rt for 1 h, before quenching with $NaHCO_3$ (250 mL). The organic layer was separated, and the aqueous layer was then washed with CH_2Cl_2 (3 x 250 mL). The combined organic extracts were then dried over $MgSO_4$, filtered and concentrated *in vacuo*. The crude product was purified by flash column chromatography (10% EtOAc in hexane) to afford the triflate as a white solid (9.60 g, >99%).

mp: 84-85 ºC.

^{1}H NMR (500 MHz, CDCl$_3$): δ 8.15 (d, *J* = 9.1 Hz, 2H), 8.01 (d, *J* = 8.3 Hz, 2H), 7.63 (d, *J* = 9.1 Hz, 2H), 7.61 – 7.57 (m, 2H), 7.43 – 7.40 (m, 2H), 7.28 – 7.25 (m, 2H).

^{13}C NMR (126 MHz, CDCl$_3$): δ 145.53, 133.29, 132.49, 132.12, 128.49, 128.12, 127.46, 126.89, 123.58, 119.46, 118.21 (q, *J* = 320.2 Hz).

HRMS (ESI$^+$): *m/z* calcd. for $C_{22}H_{12}F_6O_6S_2$ 572.9872; found 572.9872 [M+Na].

(*R*)-2,2'-dimethyl-1,1'-binaphthalene[4,5] (S6)

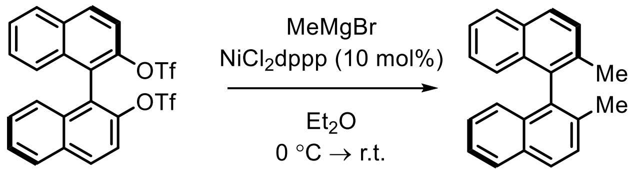

To a flame dried 100 mL flask, MeMgBr (23.4 mL, 3.0 M in Et_2O) was added dropwise, at 0 ºC, to the (*R*)-triflate (9.60 g, 17.4 mmol), and $NiCl_2$dppp (945 mg, 1.74 mmol) in dry Et_2O (100 mL). The resulting mixture was then stirred at r.t. for 18 h, before filtering the reaction through celite, and washing with copious CH_2Cl_2. The filtrate was then washed with 1M HCl (250 mL), followed by brine (250 mL). The organic layer was then dried, filtered, and concentrated *in vacuo*. The crude product was then purified by flash column chromatography (10% EtOAc in hexane) to afford the title compound as a colourless oil which solidified upon standing. (4.7 g, 96%).

mp: 68-71 ºC.

^{1}H NMR (500 MHz CDCl$_3$): δ 7.91 (t, *J* = 7.9 Hz, 4H), 7.54 (d, *J* = 8.4 Hz, 2H), 7.44 – 7.39 (m, 2H), 7.25 – 7.20 (m, 2H), 7.08 (d, *J* = 8.5 Hz, 2H), 2.07 (s, 6H).

^{13}C NMR (126 MHz, CDCl$_3$): δ 135.27, 134.43, 132.91, 132.36, 128.87, 128.07, 127.58, 126.23, 125.79, 125.03, 20.18.

HRMS (ESI$^+$): *m/z* calcd. for $C_{22}H_{18}Na$ 305.1301; found 305.1299 [M+Na].

(*R*)-2,2'-bis(bromomethyl)-1,1'-binaphthalene[4,5] (S7)

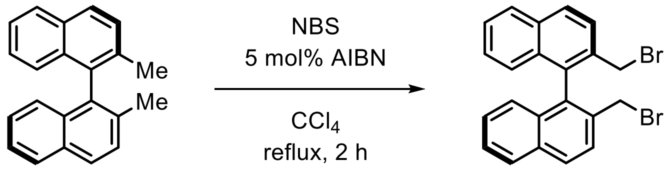

NBS (6.0 g, 33.7 mmol), was added to (*R*)-2,2'-dimethyl-1,1'-binaphthalene (4.3 g, 15.4 mmol), in CCl_4 (40 mL). AIBN (130 mg, 0.8 mmol) was then added, and the resulting mixture was then heated at reflux for 2 h. The reaction mixture was then cooled to r.t., and the resulting suspension was filtered under suction. The filtrate was concentrated *in vacuo*, before re-dissolving in EtOAc (100 mL). The organics were then washed with 0.1 M NaOH (2 x 100 mL), dried over $MgSO_4$, filtered, and concentrated *in vacuo*. The crude product was purified by recrystallisation from $CHCl_3$/hexane, to afford the product as an off-white solid (3.8 g, 56%).

mp: 130-133 ºC.

^{1}H NMR (500 MHz, $CDCl_3$): δ 8.03 (d, *J* = 8.6 Hz, 2H), 7.94 (d, *J* = 8.2 Hz, 2H), 7.76 (d, *J* = 8.6 Hz, 2H), 7.52 – 7.48 (m, 2H), 7.31 – 7.24 (m, 2H), 7.09 (d, *J* = 8.5 Hz, 2H), 4.27 (s, 4H).

^{13}C NMR (126 MHz, $CDCl_3$): δ 134.32, 134.23, 133.40, 132.64, 129.51, 128.17, 127.89, 127.00 126.96, 126.94, 32.78.

General Procedure B: Binaphthylazepine synthesis

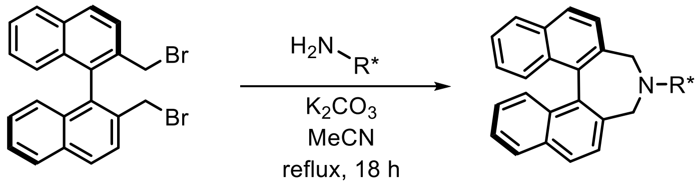

To a stirred suspension of the (*R*)-dibromide (1.00 eq.) and K_2CO_3 (3.00 eq.) in MeCN (50 mL per g dibromide), the chiral amine (1.00 eq.) was added dropwise, at room temperature (20 ºC). The resulting mixture was stirred at r.t. for 1 h, before heating at reflux for 18 h. The reaction was then cooled, and diluted with CH_2Cl_2. H_2O was added, and the organics were extracted with CH_2Cl_2 (x2). The combined organic extracts were then dried over $MgSO_4$, filtered, and concentrated *in vacuo*. The crude products were purified by flash column chromatography to afford the desired azepine.
Characterisation of the binapthylazepines was consistent with the literature[6-9]

General Procedure C: Binaphthylazepinium tetraphenylborate synthesis

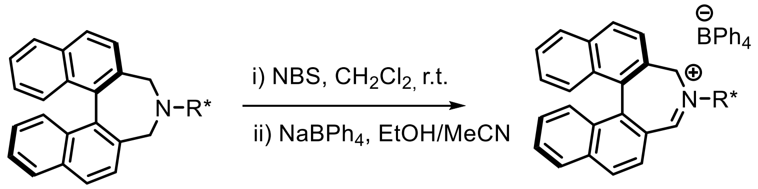

6a-6h

NBS (1.1 eq.) was added to a stirred solution of the azepine (1.0 eq.) in CH_2Cl_2 (50 mL per g of amine). The resulting solution was then stirred at r.t. until the amine is completely consumed (TLC, ~10 min), before removing CH_2Cl_2 *in vacuo*. The resulting residue is then dissolved in EtOH (30 mL per g amine), and $NaBPh_4$ (1.1 eq.) in the minimum volume of MeCN is then added slowly. The resulting mixture was stirred at r.t. for 15 minutes, before concentrating *in vacuo*. The crude product was then re-dissolved in CH_2Cl_2 (50 mL per g amine), and washed with H_2O (50 mL per g amine), followed by brine (50 mL per g amine). The organic layer was then dried over $MgSO_4$, filtered, and concentrated to afford the iminium salts as yellow-orange solids which were used without further purification.
Characterisation of the binaphthylazepinium salts **6a-6h** was consistent with the literature[6-9]

(R)-[(4S,5S)-2,2-dimethyl-4-phenyl-1,3-dioxan-5-yl]-3H-4-azepinie-cyclohepta[2,1-a;3,4-a']dinaphthalene[6,9] (8)

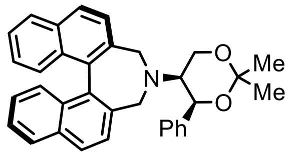

Prepared according to general procedure B, using (+)-(4*S*,5*S*)-2,2-Dimethyl-4-phenyl-1,3-dioxan-5-amine.[1b] The crude product was purified by flash column chromatography (0 → 20% EtOAc in hexane), to afford the title compound as a white solid in 68% yield.

mp: 122-124 ºC.

^{1}H NMR (500 MHz, $CDCl_3$): δ 7.91 – 7.84 (m, 4H), 7.45 – 7.33 (m, 8H), 7.32 – 7.23 (m, 3H), 7.22 – 7.16 (m, 2H), 5.17 (d, *J* = 3.3 Hz, 1H), 4.23 (dd, *J* = 12.5, 3.7 Hz, 1H), 4.14 – 4.09 (m, 1H), 3.93 (d, *J* = 12.2 Hz, 2H), 3.36 (d, *J* = 12.2 Hz, 2H), 2.72 (td, *J* = 3.5, 1.4 Hz, 1H), 1.71 (s, 3H), 1.62 (s, 3H).

^{13}C NMR (126 MHz, $CDCl_3$): 140.43, 134.96, 134.77, 132.95, 131.36, 128.51, 128.22, 127.94, 127.72, 127.65, 126.87, 126.55, 125.53, 125.22, 99.41, 75.18, 65.98, 62.01, 59.97, 53.27, 29.94, 19.19.

LCMS (ESI, m/z): calcd. for $C_{34}H_{31}NO_2$ 486.2; found 486.2 $[M+H]^+$.

***(R)*-[(4*S*,5*S*)-2,2-dimethyl-4-phenyl-1,3-dioxan-5-yl]-3H-4-azepiniumcyclohepta[2,1-a;3,4-a']dinaphthalene tetraphenylborate**[15] **(6a)**

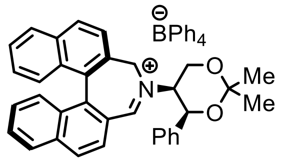

Prepared according to general procedure C to afford the title compound in quantitative yield.

$[\alpha]_D^{25}$ **=** -241.1º (c = 1.05, acetone).

^{1}H NMR (400 MHz, *d*^{6}-DMSO): δ 9.15 (s, 1H), 8.23 (d, J = 8.5 Hz, 2H), 8.17 (d, J = 8.2 Hz, 1H), 8.09 (d, J = 8.2 Hz, 1H), 7.82 (d, J = 8.4 Hz, 1H), 7.78 – 7.71 (m, 1H), 7.54 (t, J = 7.5 Hz, 1H), 7.49 – 7.33 (m, 4H), 7.29 – 7.14 (m, 11H), 7.05 (br s, 2H), 6.98 – 6.86 (m, 9H), 6.79 (t, J = 7.1 Hz, 4H), 5.89 (s, 1H), 5.81 (s, J = 10.3 Hz, 1H), 4.68 (dd, J = 13.4, 2.2 Hz, 1H), 4.59 (s, 1H), 4.30 – 4.15 (m, H), 1.79 (s, 3H), 1.74 (s, 3H).

^{13}C NMR (101 MHz, *d*^{6}-DMSO): δ 170.05, 164.09, 163.60, 163.11, 162.62, 140.39, 136.15, 135.53, 134.62, 134.51, 133.36, 131.34, 131.19, 130.64, 130.38, 130.12, 129.01, 128.88, 128.84, 128.63, 128.26, 127.66, 127.53, 127.49, 126.97, 126.62, 126.49, 125.81, 125.26, 125.24, 125.13, 124.89, 121.47, 100.23, 70.89, 66.19, 60.72, 29.33, 18.65.

LCMS (ESI, m/z): calcd. for $C_{34}H_{30}NO_2^+$ 484.2; found 484.1 $[M^+]$.

(*R*$_{ax}$)-4-((4*S*,5*S*)-2,2-Dimethyl-4-phenyl-1,3-dioxan-5-yl)-3-methyl-4,5-dihydro-3H-dinaphtho[2,1-c:1′,2′-e]azepine[9] (6h)

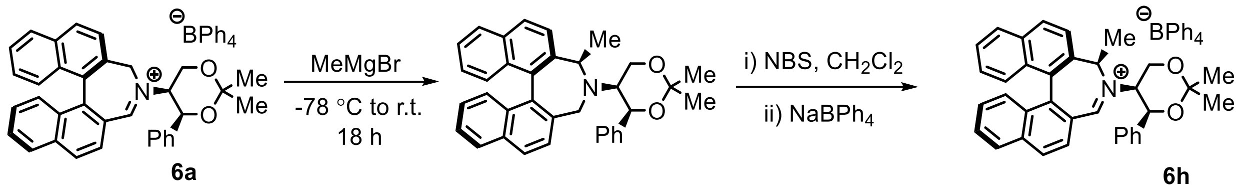

The title compound was prepared according to the procedure by Page.[9] Iminium **6a** (300 mg, 0.37 mmol), was dissolved in dry THF (50 mL). The resulting solution was cooled to -78 ºC, before adding MeMgBr (1.24 mL, 3.0 M in Et_2O) dropwise. The reaction was kept at – 78 ºC for 1 h, before slowly allowing to warm to room temperature (20 ºC) overnight. Sat. aq. NH_4Cl (50 mL) was then added to quench the reaction. THF was removed *in vacuo*, and then $CHCl_3$ (50 mL) was added. The organic layer was separated, before washing with H_2O (50 mL), followed by brine (50 mL). The organics were then dried over $MgSO_4$, filtered, and concentrated. The crude product was purified by flash column chromatography (0 → 20% EtOAc in hexane) to afford the azepine as a white solid (165 mg, 89%).

The azepine was then dissolved in CH_2Cl_2 (5 mL), before adding NBS (54 mg, 0.30 mmol). The resulting solution was stirred at r.t. until the amine was completely consumed (TLC, 10 min), before removing CH_2Cl_2 *in vacuo*. EtOH (2 mL) was added, and $NaBPh_4$ (94.5 mg, 0.30 mmol) in MeCN (1 mL) was added slowly. The resulting mixture was stirred at r.t. for 15 minutes, before concentrating *in vacuo*. The crude product was re-dissolved in CH_2Cl_2 (15 mL), and washed with H_2O (15 mL), followed by brine (15 mL). The organic layer was then dried over $MgSO_4$, filtered, and concentrated to afford the iminium salt as an orange solid (230 mg, 76% over 2 steps). Characterisation of the azepine, and azepinium salt **6i** was in agreement with the literature.[9]

Substrate synthesis

General Procedure D: phenol *o*-benzylation

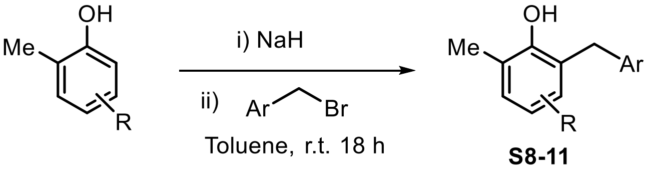

S8-11

The reactions were performed using an adapted, *unoptimized* version of a procedure reported by Hori *et. al.*[10] Note: Newer, more reactive NaH was found to be detrimental to the reaction.

To a vigorously stirred solution of the desired phenol (1.0 eq.) in dry toluene (2.5 mL mmol^{-1}), NaH (60% wt. dispersion in mineral oil, 1.5 eq.) is added at 0 ºC. After *ca.* 20 minutes, the benzyl bromide (1.1 eq.) is added dropwise, before slowly allowing the reaction mixture to warm to room temperature (20 ºC). The resulting suspension is then stirred vigorously for 18 h, before quenching with saturated NH_4Cl. The organics are then extracted with EtOAc (x3), dried, filtered and concentrted *in vacuo*. The crude products are then purified by flash column chromatography to afford the pure 2-benzyl phenols.

2-benzyl-6-methylphenol[11] (S8)

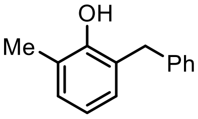

Prepared from *o*-cresol and benzyl bromide on a 9.2 mmol scale, to afford the product in 27% yield. Characterization was consistent with the literature.[11]

6-benzyl-2,3-dimethylphenol (S9)

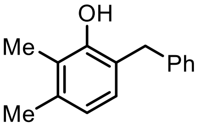

Prepared from 2,3-dimethylphenol and benzyl bromide on a 4.1 mmol scale, to afford the product in 20% yield.

^{1}H NMR (400 MHz, $CDCl_3$): δ 7.33 – 7.28 (m, 2H), 7.25 – 7.19 (m, 3H), 6.90 (d, *J* = 7.6 Hz, 1H), 6.74 (d, *J* = 7.6 Hz, 1H), 4.60 (s, 1H), 3.98 (s, 2H), 2.28 (s, 3H), 2.15 (s, 3H).

^{13}C NMR (126 MHz, $CDCl_3$): δ 152.08, 139.94, 136.56, 128.88, 128.73, 127.73, 126.60, 123.93, 122.79, 122.13, 37.04, 20.22, 11.80.

HRMS (ESI$^+$): *m/z* calcd. for $C_{15}H_{16}ONa$ 235.1093; found 235.1093 [M+Na].

2,3-dimethyl-6-[(4-methylphenyl)methyl]phenol (S10)

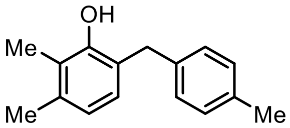

Prepared from 2,3-dimethylphenol and 4-methylbenzyl bromide on a 2.5 mmol scale, to afford the product in 47% yield.

^{1}H NMR (400 MHz, $CDCl_3$): δ 7.16 – 7.09 (m, 4H), 6.91 (d, *J* = 7.6 Hz, 1H), 6.74 (d, *J* = 7.6 Hz, 1H), 4.67 – 4.62 (m, 1H), 3.94 (s, 2H), 2.33 (s, 3H), 2.28 (s, 3H), 2.15 (s, 3H).

^{13}C NMR (101 MHz, $CDCl_3$): δ 152.14, 136.68, 136.51, 136.21, 129.62, 128.59, 127.65, 124.08, 122.90, 122.05, 36.75, 21.15, 20.20, 11.78.

HRMS (ESI$^+$): *m/z* calcd. for $C_{16}H_{18}ONa$ 249.1250; found 249.1250 [M+Na].

6-[(4-fluorophenyl)methyl]-2,3-dimethylphenol (S11)

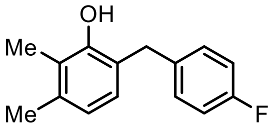

Prepared from 2,3-dimethylphenol and 4-fluorobenzyl bromide on a 4.1 mmol scale, to afford the product in 48% yield.

^{1}H NMR (400 MHz, $CDCl_3$): δ 7.24 – 7.17 (m, 2H), 7.02 – 6.95 (m, 2H), 6.89 (d, *J* = 7.6 Hz, 1H), 6.76 (d, *J* = 7.6 Hz, 1H), 4.60 (s, *J* = 2.5 Hz, 1H), 3.95 (s, 2H), 2.29 (s, 3H), 2.16 (s, 3H).

^{13}C NMR (101 MHz, $CDCl_3$): δ 162.89, 160.46, 151.92, 136.59, 135.83, 135.80, 130.18, 130.10, 127.62, 123.97, 122.55, 122.23, 115.63, 115.42, 36.05, 20.23, 11.77.

^{19}F NMR (376 MHz, $CDCl_3$): δ -116.76 – -116.86 (m).

HRMS (ESI$^+$): *m/z* calcd. for $C_{15}H_{15}FONa$ 253.0999; found 253.0999 [M+Na].

Optimisation of the synthesis of (±)-bis(2,6-xylenol)

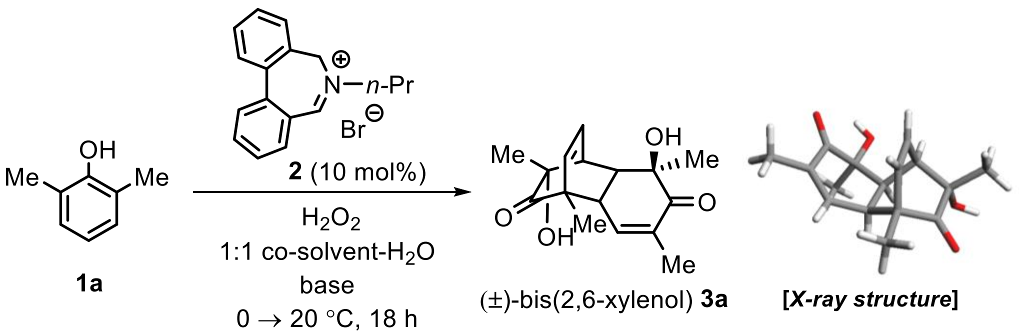

Entry	Solvent	Base[a]	H_2O_2 eq.[b]	Yield[c]
**1**[d]	MeCN-H_2O (1:1)	Na_2CO_3	1.5	17%
**2**	MeCN-H_2O (1:1)	Na_2CO_3	2.0	34%
**3**	MeCN-H_2O (1:1)	Na_2CO_3	3.0	65%
**4**	MeCN-H_2O (1:1)	NaOH	3.0	< 5%
**5**	MeOH-H_2O (1:1)	Na_2CO_3	3.0	< 10%
**6**	CH_2Cl_2-H_2O (1:1)	Na_2CO_3	3.0	< 5%
**7**	THF-H_2O (1:1)	Na_2CO_3	3.0	< 5%
**8**	PhCN-H_2O (1:1)	Na_2CO_3	3.0	55%
**9**	MeCN-H_2O (1:1)	none	3.0	< 5%
**10**	MeCN-H_2O (9:1)	Na_2CO_3	3.0	< 5%

Table S1. Optimization of the racemic synthesis of **3a**. Reactions performed on a 0.4 mmol scale. [a]2.5 mmol. [b]Used as a 30% aq. solution. [c]Isolated yields after chromatography. [d]4 h reaction time.

Extended optimisation of the enantioselective phenol dearomatization reaction

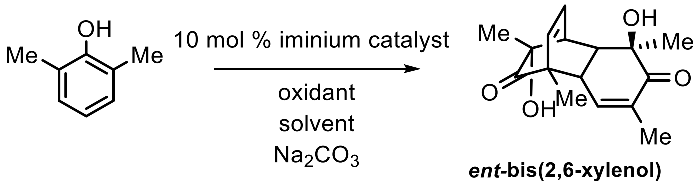

Entry	Oxidant[a]	Solvent	Catalyst	*T* (ºC)	Yield	e.r.[c]
**1**	H_2O_2	MeCN-H_2O (1:1)	**4a**	20 ºC	86%	79:21
**2**	H_2O_2	MeCN-H_2O (1:1)	**5**	20 ºC	90%	60:40
**3**	Oxone	MeCN-H_2O (1:1)	**4a**	20 ºC	14%	76:24
**4**	H_2O_2	MeCN-H_2O (1:1)	**4b**	20 ºC	96%	79:21
**5**	H_2O_2	MeCN-H_2O (1:1)	**4a**	0 ºC	91%	85.5:14.5
**6**	UHP/$PhSe_2$[b]	$CHCl_3$	**4a**	0 ºC	52%	88:12
**7**	H_2O_2	MeCN-H_2O (1:1)	**6a**	0 ºC	75%	**95:5**
**8**	UHP/$PhSe_2$[b]	$CHCl_3$	**6a**	0 ºC	53%	87:13
**9**	H_2O_2	MeCN-H_2O (1:1)	**6b**	0 ºC	56%	10:90
**10**[d]	H_2O_2	MeCN-H_2O (1:1)	**6a**	0 ºC	40%	91:9
**11**[e]	H_2O_2	MeCN-H_2O (2:1)	**7**	0 ºC	29%	42:58
**12**	H_2O_2	MeCN-H_2O (1:1)	**6c**	0 ºC	74%	88:12
**13**	H_2O_2	MeCN-H_2O (1:1)	**6d**	0 ºC	42%	79:21
**14**	H_2O_2	MeCN-H_2O (1:1)	**6e**	0 ºC	53%	84:16
**15**	H_2O_2	MeCN-H_2O (1:1)	**6f**	0 ºC	49%	74:26
**16**	H_2O_2	MeCN-H_2O (1:1)	**6g**	0 ºC	61%	79:21
**17**	H_2O_2	MeCN-H_2O (1:1)	**6h**	0 ºC	69%	95:5

Table S2. Extended optimization of the enantioselective *o*-HPD-[4+2] reaction. All reactions performed on an 0.4 mmol scale.[a] 3.0 eq. [b]3.0 eq. UHP, 5 mol% $PhSe_2$. [c] Measured by chiral stationary phase HPLC. [d] Performed at pH = 10 using Na_2CO_3-$NaHCO_3$ buffer.[e] Employed using Shi's reported conditions: substrate (0.5 mmol), MeCN (1 mL), 0.5 mL 1.0 M K_2CO_3 in 0.4 mM EDTA, H_2O_2 (1.5 mmol), 30 mol% D-epoxone (Shi catalyst).

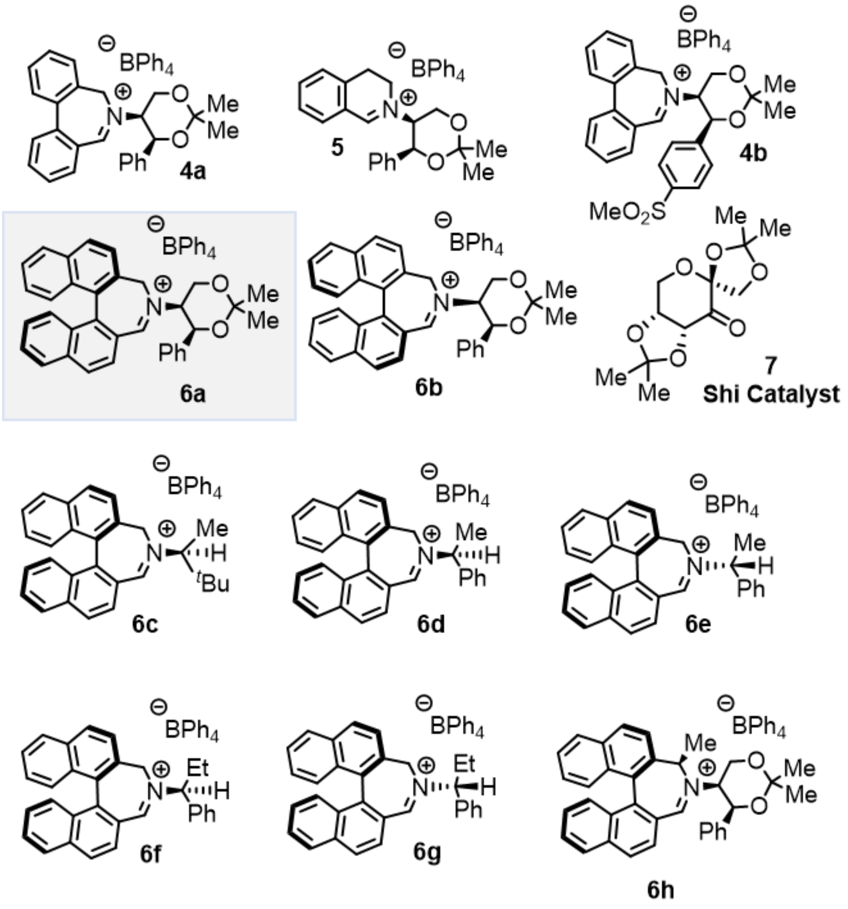

Figure S1. Screened chiral catalysts

General Procedure E for the preparation of racemic compounds

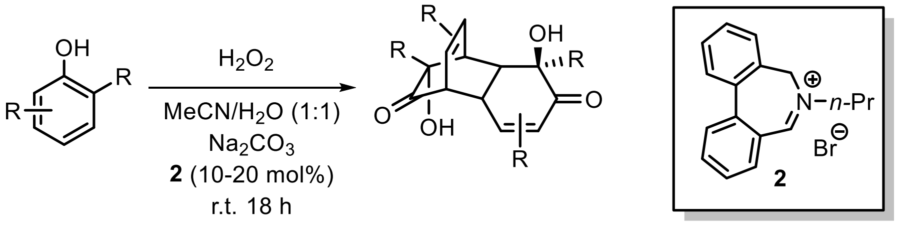

To a stirred mixture of the phenol (0.41 mmol), catalyst **2** (10 or 20 mol%), and Na_2CO_3 (2.46 mmol) in 1:1 MeCN-H_2O (2.5 mL), H_2O_2 (126 µL, 30% aq. soln) was added at 0 ºC. The resulting mixture was then warmed to room temperature (20 ºC) and stirred overnight, before quenching the peroxide with $Na_2S_2O_3$. The reaction was then diluted with CH_2Cl_2, and extracted with CH_2Cl_2 (3 x 15 mL). The combined organics were then dried over $MgSO_4$, filtered, and concentrated *in vacuo*. Purification was achieved using flash column chromatography.

Note: *Where stated, preparative TLC was performed to obtain a racemic sample suitable for HPLC. In general, reactions perform considerably better with chiral catalysts* ***4a-6a****.*

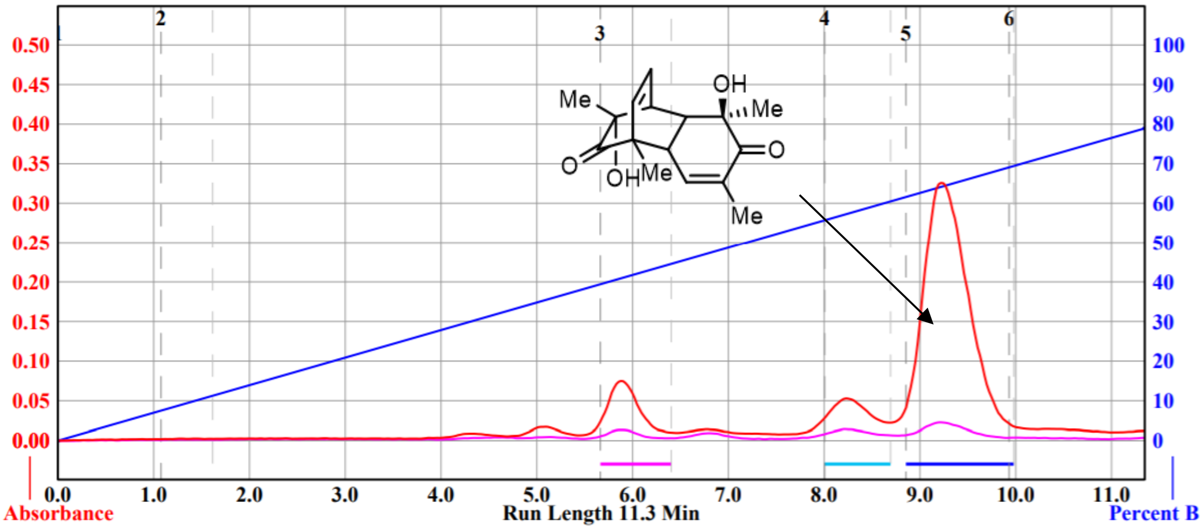

Figure S2. Representative Combiflash trace for the tandem HPD-[4+2] cycloaddition using **2**, exemplified for the dearomatization of 2,6-dimethylphenol. t_R = 9.5 min corresponds to the bicyclo[2.2.2]octenone product

General Procedure F for the enantioselective phenol dearomatization reactions

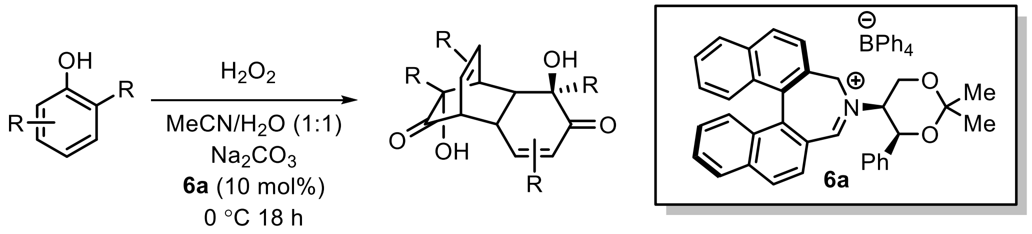

To a stirred mixture of the phenol (0.41 mmol), catalyst **6a** (10 mol%), and Na_2CO_3 (2.46 mmol) in 1:1 MeCN-H_2O (2.5 mL) at 0 ºC, H_2O_2 (126 µL, 30% aq. soln) was added. The resulting mixture was then stirred overnight at 0 ºC, before quenching the peroxide with $Na_2S_2O_3$. The reaction was then diluted with CH_2Cl_2 and extracted with CH_2Cl_2 (2 x 15 mL). The combined organics were then dried over $MgSO_4$, filtered, and concentrated *in vacuo*. Purification was achieved using flash column chromatography.

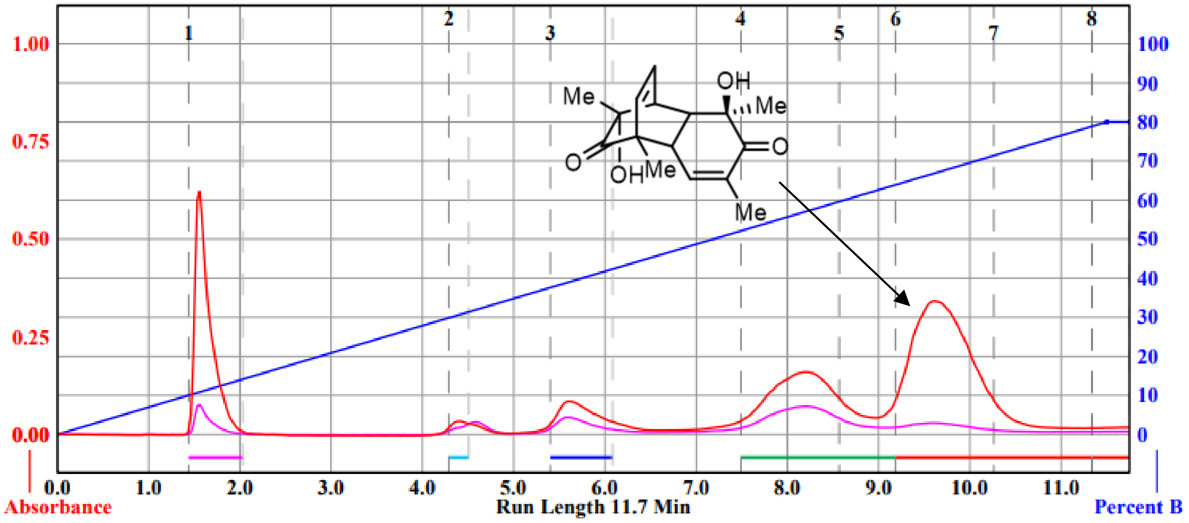

Figure S3. Representative Combiflash trace for the tandem HPD-[4+2] cycloaddition using chiral catalyst **6a**, exemplified for the dearomatization of 2,6-dimethylphenol. t_R = 9.5 min corresponds to the bicyclo[2.2.2]octenone product

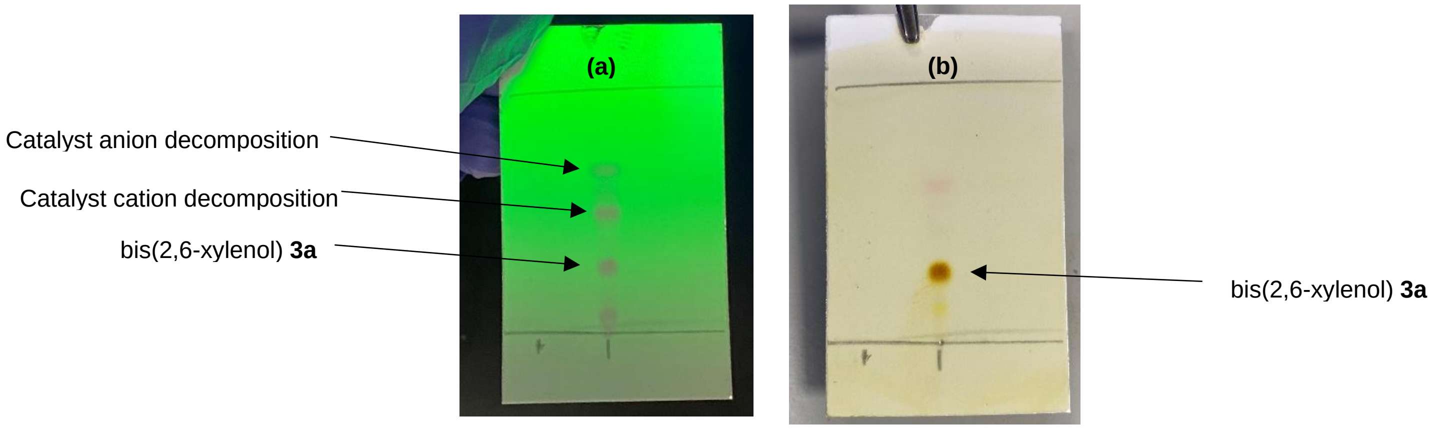

Figure S4. Representative TLC (1:1 hexane:EtOAc) for the *o*-HPD-[4+2] reaction of 2,6-dimethylphenol. (a) short wave UV visualisation (b) vanillin staining highlights the product.

(+)-bis(2,6-xylenol)[12,13] (+)-3a

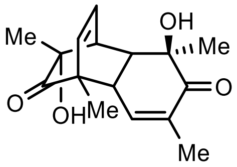

The racemic material was prepared according to general procedure E. The crude product was purified by automated flash column chromatography (0 → 80% EtOAc in hexane) to yield the title compound as a white solid (37 mg, 66%). Chiral HPLC achieved with Chiralpak IC column (75:25 Hexane:IPA) t_R = 12.5 min (enantiomer 1), 21.5 min (enantiomer 2). Flow rate 0.75 mL min^{-1}.

The enantioselective reaction was achieved according to general procedure F. The crude mixture was purified by flash column chromatography (hexanes-EtOAc), to obtain the product as a white solid (42 mg, 75% yield, 95:5 e.r.).

mp: 171-173 ºC.

^{1}H NMR (400 MHz, CDCl$_3$): δ 6.30 – 6.23 (m, 2H), 5.51 (dd, *J* = 8.1, 0.9 Hz, 1H), 4.01 (s, 1H), 3.38 (dt, *J* = 6.8, 1.7 Hz, 1H), 3.25 (dd, *J* = 8.4, 2.0 Hz, 1H), 2.87 (dd, *J* = 6.9, 5.1 Hz, 1H), 2.40 (s, 1H), 1.85 (t, *J* = 1.4 Hz, 3H), 1.34 (s, 3H), 1.31 (s, 3H), 1.24 (s, 3H).

^{13}C NMR (101 MHz, CDCl$_3$): δ 214.99, 203.15, 139.43, 135.91, 135.50, 133.38, 73.80, 73.08, 53.89, 44.37, 43.81, 42.87, 31.90, 26.38, 16.52, 15.80.

LCMS (ESI$^+$): *m/z* calcd. for $C_{16}H_{20}O_4$ 277.1; found 276.9 [M+H].

$[\alpha]_D^{25}$ = + 113.5º (c = 0.16, CHCl$_3$).

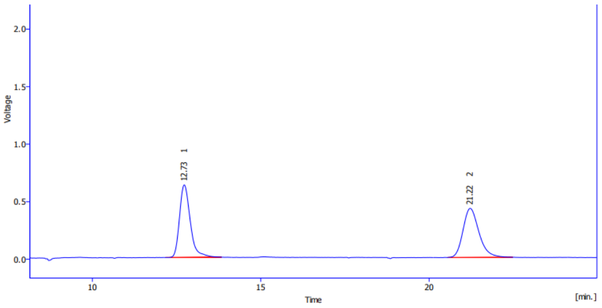

Result Table (Uncal - C:\CLARITY\TOM\TDD-160 TEST)

	Reten. Time [min]	Area [mV.s]	Height [mV]	Area [%]	Height [%]	W05 [min]
1	12.728	14009.417	630.059	49.9	59.6	0.34
2	21.216	14051.254	426.643	50.1	40.4	0.50
	Total	28060.671	1056.702	100.0	100.0	

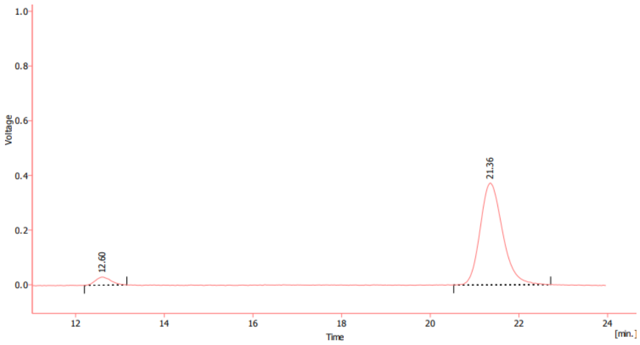

Result Table (Uncal - C:\Clarity\TOM\TDD-256 isolated)

	Reten. Time [min]	Area [mV.s]	Height [mV]	Area [%]	Height [%]	W05 [min]
1	12.604	712.872	29.577	5.2	7.4	0.39
2	21.356	13098.111	372.285	94.8	92.6	0.54
	Total	13810.983	401.862	100.0	100.0	

(−)-bis(2,6-xylenol)[12,13] (−)-3a

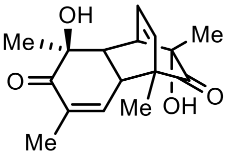

The enantioselective reaction was achieved according to general procedure F, using catalyst *ent*-**6a**. The crude mixture was purified by flash column chromatography (hexanes-EtOAc), to obtain the product as a white solid (44.5 mg, 78% yield, 94:6 e.r.). Analytical data was identical to that described for (+)-bis(2,6-xylenol).

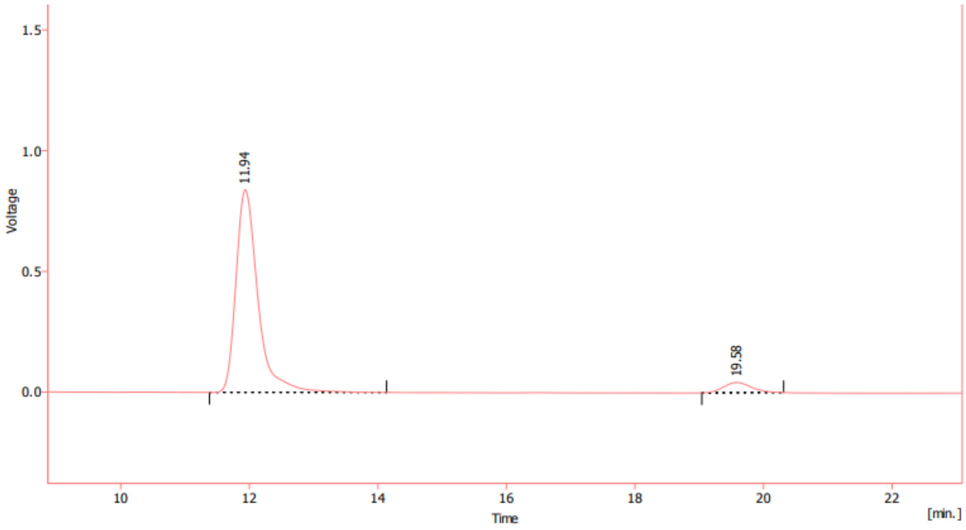

Result Table (Uncal - C:\Clarity\TOM\TDD-555c)

	Reten. Time [min]	Area [mV.s]	Height [mV]	Area [%]	Height [%]	W05 [min]
1	11.936	19977.465	839.697	94.0	95.2	0.34
2	19.584	1275.975	42.308	6.0	4.8	0.48
	Total	21253.440	882.005	100.0	100.0	

8,10-dihydroxy-2,4,4a,6,8,10-hexamethyl-4,4a,8,8a-tetrahydro-1,4-ethanonaphthalene-7,9(1H)-dione[12] (3b)

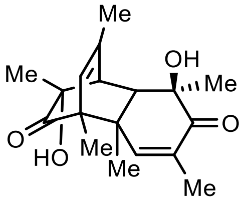

Prepared using general procedure E from 2,4,6-trimethylphenol (55.8 mg, 0.41 mmol). The crude product was purified by automated flash column chromatography (0 → 50% EtOAc in hexane) to yield the title compound as a white solid (41 mg, 66%). Chiral HPLC was achieved with Chiralpak IC column (75:25 Hexane:IPA) t_R = 8.3 min (enantiomer 1), 9.7 min (enantiomer 2). Flow rate 0.75 mL min^{-1}.
The enantioselective reaction was achieved according to general procedure F. The crude mixture was purified by flash column chromatography (hexanes-EtOAc), to obtain the product as a white solid (49 mg, 79% yield, 95:5 e.r.).

mp: 166-168 ºC.
^{1}H NMR (400 MHz, CDCl$_3$): δ 6.02 (s, 1H), 5.08 – 5.00 (m, 1H), 3.93 (s, 1H), 3.14 (t, *J* = 2.0 Hz, 1H), 2.80 (d, *J* = 1.9 Hz, 1H), 2.31 (s, 1H), 1.83 (d, *J* = 1.4 Hz, 3H), 1.70 (d, *J* = 1.6 Hz, 3H), 1.36 (s, 3H), 1.24 (s x2, 6H), 1.16 (s, 3H).
^{13}C NMR (101 MHz, CDCl$_3$): δ 214.26, 202.61, 145.39, 145.23, 133.22, 127.73, 73.87, 72.39, 57.88, 48.86, 48.77, 45.50, 32.59, 25.34, 23.33, 21.59, 16.39, 12.58.
LCMS (ESI$^+$): *m/z* calcd. for $C_{18}H_{24}O_4$ 305.2; found 305.0 [M+H].

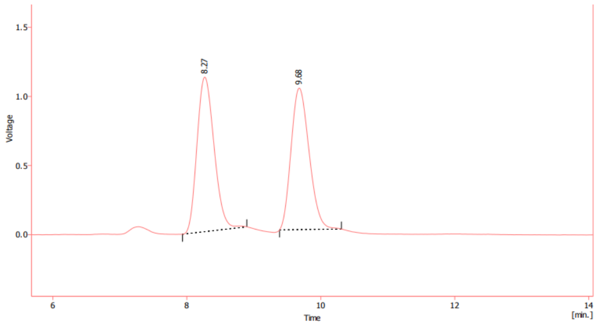

Result Table (Uncal - C:\Clarity\TOM\TDD-179c (rac))

	Reten. Time [min]	Area [mV.s]	Height [mV]	Area [%]	Height [%]	W05 [min]
1	8.268	19828.031	1117.743	50.8	52.2	0.28
2	9.676	19210.188	1023.659	49.2	47.8	0.30
	Total	39038.219	2141.402	100.0	100.0	

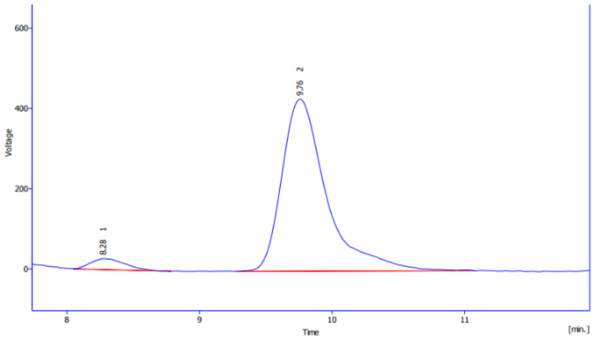

Result Table (Uncal - C:\CLARITY\TOM\TDD-259 ISO (TRIME)B)

	Reten. Time [min]	Area [mV.s]	Height [mV]	Area [%]	Height [%]	W05 [min]
1	8.276	499.739	27.294	4.8	6.0	0.29
2	9.760	10020.866	428.859	95.2	94.0	0.33
	Total	10520.606	456.153	100.0	100.0	

3,5,8,10-tetraethyl-3,10-dihydroxytricyclo[6.2.2.0^{2,7}]dodeca-5,11-diene-4,9-dione (3c)

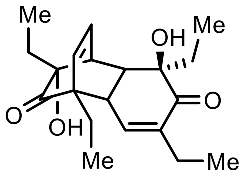

The racemic material was prepared using general procedure E from 2,6-diethylphenol (0.41 mmol). Prep. TLC was performed on the crude product using 7:3 hexanes:EtOAc to obtain a racemic sample of the title compound. Chiral HPLC was achieved with Chiralpak IC column (75:25 Hexane:IPA) t_R = 8.5 min (enantiomer 1), 12 min (enantiomer 2). Flow rate 0.75 mL min^{-1}

The enantioselective reaction was achieved according to general procedure F. The crude mixture was purified by flash column chromatography (hexanes-EtOAc), to obtain the product as a white solid (53.5 mg, 79% yield, 98:2 e.r.).

mp: 90-92 ºC.

IR (cm^{-1}): 3434, 2970, 2932, 1727, 1672.

^{1}H NMR (400 MHz, CDCl$_3$): δ 6.26 (dd, *J* = 8.2, 6.8 Hz, 1H), 6.19 (d, *J* = 4.3 Hz, 1H), 5.58 (dd, *J* = 8.3, 1.3 Hz, 1H), 4.00 (s, 1H), 3.38 (dt, *J* = 6.8, 1.7 Hz, 1H), 3.13 (dd, *J* = 8.2, 1.8 Hz, 1H), 2.96 (dd, *J* = 8.2, 4.3 Hz, 1H), 2.44 – 2.10 (m, 3H), 1.95 – 1.68 (m, 2H), 1.62 (q, *J* = 7.4 Hz, 2H), 1.53 – 1.32 (m, 2H), 1.05 – 0.94 (m, 6H), 0.87 (t, *J* = 7.5 Hz, 3H), 0.66 (t, *J* = 7.4 Hz, 3H).

^{13}C NMR (126 MHz, CDCl$_3$): δ 215.98, 203.02, 141.61, 137.89, 134.31, 132.23, 76.90, 76.31, 58.48, 44.44, 42.28, 41.46, 37.07, 29.92, 23.29, 22.42, 12.79, 9.54, 7.38, 7.05.

HRMS (ESI$^+$): *m/z* calcd. for $C_{20}H_{28}O_4Na$ 355.1880; found 355.1879 [M+Na].

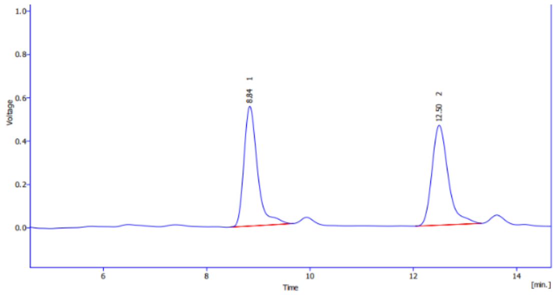

Result Table (Uncal - C:\CLARITY\TOM\TDD-381 RAC)

	Reten. Time [min]	Area [mV.s]	Height [mV]	Area [%]	Height [%]	W05 [min]
1	8.836	10054.879	552.586	50.0	54.5	0.27
2	12.500	10045.693	460.632	50.0	45.5	0.32
	Total	20100.572	1013.218	100.0	100.0	

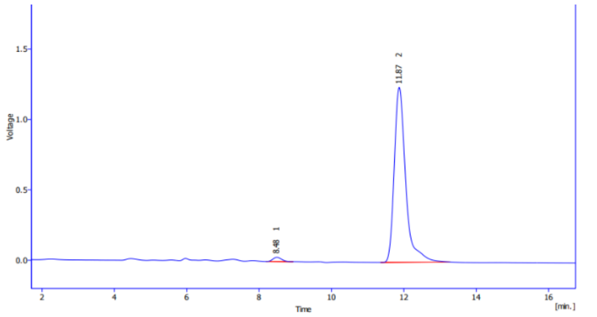

Result Table (Uncal - C:\CLARITY\TOM\TDD-380B)

	Reten. Time [min]	Area [mV.s]	Height [mV]	Area [%]	Height [%]	W05 [min]
1	8.484	502.153	29.894	1.8	2.3	0.27
2	11.872	27300.118	1242.802	98.2	97.7	0.32
	Total	27802.271	1272.697	100.0	100.0	

3,10-Dihydroxy-3,5,6,8,10,12-hexamethyltricyclo[6.2.2.02,7]dodeca-5,11-diene-4,9-dione[12] (3d)

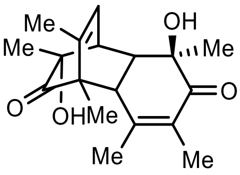

The racemic material was prepared according to procedure E, from 2,3,6-trimethylphenol (55.8 mg, 0.41 mmol). The crude mixture was purified by automated flash column chromatography (0 → 80% EtOAc in hexane) to yield the title compound as an off-white solid (42.6 mg, 68%). Chiral HPLC was achieved with Chiralpak IC column (75:25 Hexane:IPA) t_R = 12 min (enantiomer 1), 25 min (enantiomer 2). Flow rate 0.75 mL min^{-1}.

The enantioselective reaction was achieved according to general procedure F. The crude mixture was purified by flash column chromatography (hexanes-EtOAc), to obtain the product as a white solid (45 mg, 72% yield, 97:3 e.r.).

mp: 179-181 ºC.

^{1}H NMR (500 MHz, CDCl$_3$): δ 5.97 (dd, J = 6.9, 1.4 Hz, 1H), 4.13 (s, 1H), 3.34 (dd, J = 6.9, 2.7 Hz, 1H), 2.99 (dd, J = 8.6, 2.6 Hz, 1H), 2.88 (d, J = 8.6 Hz, 1H), 2.28 (s, 1H), 1.99 (d, J = 0.8 Hz, 3H), 1.83 – 1.80 (m, 3H), 1.45 (d, J = 1.6 Hz, 3H), 1.24 (s, 3H), 1.23 (s, 3H), 1.18 (s, 3H).

^{13}C NMR (126 MHz, CDCl$_3$): δ 213.90, 202.95, 148.43, 137.09, 132.45, 131.21, 73.82, 72.11, 56.55, 49.12, 44.30, 41.40, 32.20, 26.37, 23.75, 18.80, 15.27, 13.66.

LCMS (ESI$^+$): *m/z* calcd. for $C_{18}H_{24}O_4$ 305.2; found 305.0 [M+H].

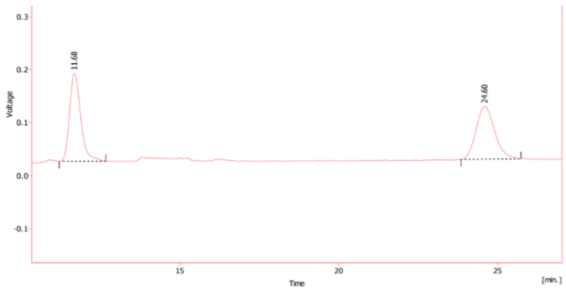

Result Table (Uncal - C:\Clarity\TOM\TDD-209 rac)

	Reten. Time [min]	Area [mV.s]	Height [mV]	Area [%]	Height [%]	W05 [min]
1	11.684	4071.167	166.059	50.2	62.5	0.37
2	24.600	4038.162	99.595	49.8	37.5	0.63
	Total	8109.329	265.654	100.0	100.0	

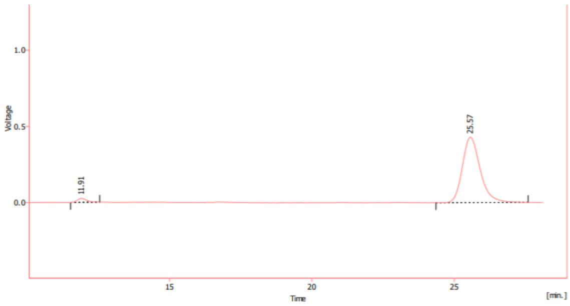

Result Table (Uncal - C:\Clarity\TOM\TDD-261)

	Reten. Time [min]	Area [mV.s]	Height [mV]	Area [%]	Height [%]	W05 [min]
1	11.912	585.014	25.492	3.0	5.6	0.36
2	25.568	18702.431	430.492	97.0	94.4	0.66
	Total	19287.446	455.985	100.0	100.0	

(3R)-3,10-dibenzyl-3,10-dihydroxy-5,6,8,12-tetramethyltricyclo[6.2.2.0^{2,7}]dodeca-5,11-diene-4,9-dione (3e)

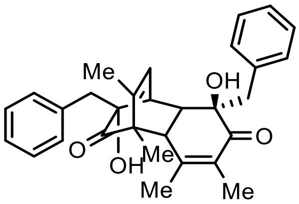

The racemic material was prepared using general procedure E from 2-benzyl-5,6-dimethyl phenol (0.28 mmol). Prep. TLC was performed on the crude product using 2:1 hexanes:EtOAc to obtain a racemic sample of the title compound. Chiral HPLC was achieved with Chiralpak IC column (60:40 Hexane:IPA) t_R = 10 min (enantiomer 1), ca. 28 min (enantiomer 2). Flow rate 0.75 mL min^{-1}.
The enantioselective reaction was achieved according to general procedure F (0.37 mmol). The crude mixture was purified by flash column chromatography (hexanes-EtOAc), to obtain the product as a white solid (61.7 mg, 73% yield, 97:3 e.r.).

mp: 64-65 ºC
IR (cm^{-1}): 3446, 3027, 2918, 2851, 1720, 1670.
^{1}H NMR (400 MHz, CDCl$_3$): δ 7.35 – 7.27 (m, 3H), 7.18 – 7.13 (m, 5H), 6.94 – 6.89 (m, 2H), 5.95 (dd, *J* = 6.9, 1.5 Hz, 1H), 4.10 (s, 1H), 3.25 (dd, *J* = 6.9, 2.4 Hz, 1H), 3.05 (dd, *J* = 8.5, 2.4 Hz, 1H), 2.96 (d, *J* = 8.5 Hz, 1H), 2.83 (d, *J* = 3.0 Hz, 1H), 2.80 (d, *J* = 3.4 Hz, 1H), 2.71 (d, *J* = 13.9 Hz, 1H), 2.64 (d, *J* = 13.4 Hz, 1H), 2.22 (s, 1H), 1.99 (d, *J* = 0.9 Hz, 3H), 1.67 – 1.65 (m, 3H), 1.45 (d, *J* = 1.6 Hz, 3H), 1.31 (s, 3H).
^{13}C NMR (126 MHz, CDCl$_3$): δ 213.26, 200.72, 148.66, 137.21, 135.75, 135.54, 132.02, 131.77, 130.50, 129.96, 128.58, 128.13, 127.23, 126.98, 77.27, 74.57, 57.08, 51.38, 50.63, 43.68, 41.58, 40.78, 23.76, 18.92, 15.56, 13.62.
HRMS (ESI$^+$): *m/z* calcd. for $C_{30}H_{32}O_4Na$ 479.2192; found 479.2193 [M+Na].

Note: *The second enantiomer has a very broad peak in the HPLC chromatogram. Alternative chiral stationary phases and eluents were unsuccessful in separating the enantiomers.*

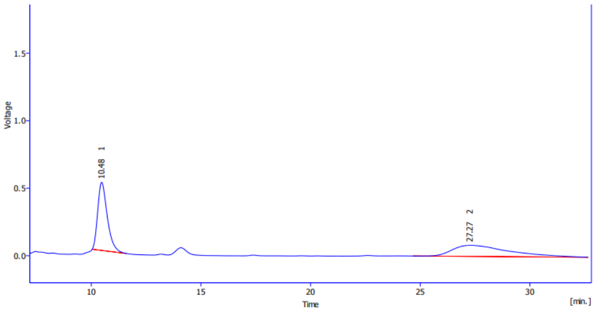

Result Table (Uncal - C:\CLARITY\TOM\TDD-474 RAC C)

	Reten. Time [min]	Area [mV.s]	Height [mV]	Area [%]	Height [%]	W05 [min]
1	10.476	15315.946	504.899	50.9	86.0	0.46
2	27.272	14796.665	82.396	49.1	14.0	2.60
	Total	30112.611	587.295	100.0	100.0	

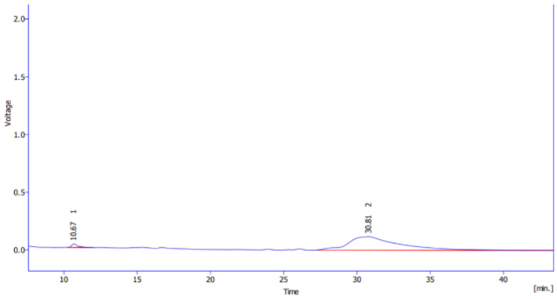

Result Table (Uncal - C:\Clarity\TOM\TDD-471 IC-60-40)

	Reten. Time [min]	Area [mV.s]	Height [mV]	Area [%]	Height [%]	W05 [min]
1	10.672	955.590	31.080	3.2	20.9	0.41
2	30.808	29062.778	117.912	96.8	79.1	3.26
	Total	30018.368	148.992	100.0	100.0	

(3R)-3,10-dihydroxy-5,6,8,12-tetramethyl-3,10-bis[(4-methylphenyl)methyl]tricyclo[6.2.2.0^{2,7}]dodeca-5,11-diene-4,9-dione (3f)

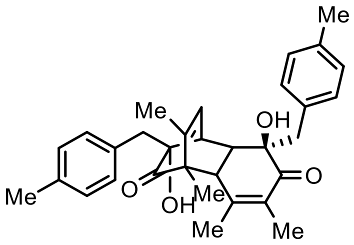

The racemic material was prepared using general procedure E. Prep. TLC was performed on the crude product using 2:1 hexanes:EtOAc to obtain a racemic sample of the title compound. Chiral HPLC was achieved with Chiralpak IC column (75:25 Hexane:IPA) t_R = 19 min (enantiomer 1), 36.5 min (enantiomer 2). Flow rate 0.75 mL min^{-1}.

The enantioselective reaction was achieved according to general procedure F (0.26 mmol). The crude mixture was purified by flash column chromatography (hexanes-EtOAc), to obtain the product as a white solid (45 mg, 70% yield, 96:4 e.r.).

mp: 148-149 ºC

IR (cm^{-1}): 3388, 3018, 2939, 2923, 1717, 1667.

^{1}H NMR (400 MHz, CDCl$_3$): δ 7.12 (d, *J* = 7.9 Hz, 2H), 7.04 (d, *J* = 8.0 Hz, 2H), 6.98 (d, *J* = 7.8 Hz, 2H), 6.79 (d, *J* = 7.9 Hz, 2H), 5.97 (dd, *J* = 6.9, 1.3 Hz, 1H), 4.08 (s, 1H), 3.24 (dd, *J* = 6.9, 2.4 Hz, 1H), 3.03 (dd, *J* = 8.5, 2.3 Hz, 1H), 2.95 (d, *J* = 8.5 Hz, 1H), 2.77 (d, *J* = 13.7 Hz, 2H), 2.66 (d, *J* = 13.9 Hz, 1H), 2.60 (d, *J* = 13.5 Hz, 1H), 2.33 (s, 3H), 2.26 (s, 3H), 2.18 (s, 1H), 1.99 (s, 3H), 1.67 (s, 3H), 1.46 (d, *J* = 1.4 Hz, 3H), 1.30 (s, 3H).

^{13}C NMR (126 MHz, CDCl$_3$): δ 213.31, 200.86, 148.60, 137.17, 136.81, 136.44, 132.60, 132.30, 131.97, 131.84, 130.35, 129.81, 129.32, 128.85, 77.34, 74.51, 57.09, 51.01, 50.63, 43.21, 41.58, 40.73, 23.76, 21.23, 21.19, 18.93, 15.58, 13.63.

HRMS (ESI$^+$): *m/z* calcd. for $C_{32}H_{36}O_4Na$ 507.2506; found 507.2506 [M+Na].

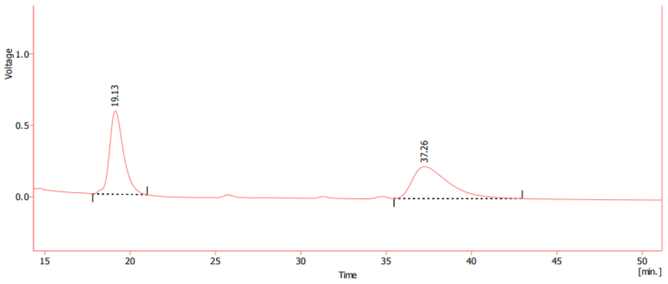

Result Table (Uncal - C:\Clarity\TOM\TDD-478 RAC)

	Reten. Time [min]	Area [mV.s]	Height [mV]	Area [%]	Height [%]	W05 [min]
1	19.132	33217.795	582.749	50.2	72.3	0.84
2	37.260	32892.953	223.089	49.8	27.7	2.22
	Total	66110.748	805.839	100.0	100.0	

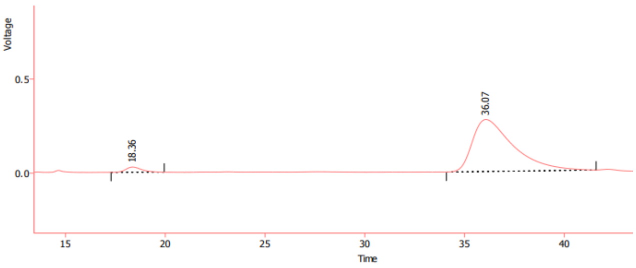

Result Table (Uncal - C:\Clarity\TOM\TDD-476a)

	Reten. Time [min]	Area [mV.s]	Height [mV]	Area [%]	Height [%]	W05 [min]
1	18.356	1532.614	27.743	3.8	9.1	0.84
2	36.072	39329.956	276.403	96.2	90.9	2.11
	Total	40862.570	304.145	100.0	100.0	

(3R)-3,10-bis[(4-fluorophenyl)methyl]-3,10-dihydroxy-5,6,8,12-tetramethyltricyclo[6.2.2.0^{2,7}]dodeca-5,11-diene-4,9-dione (3g)

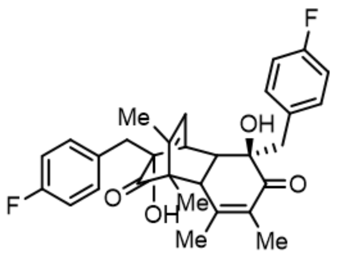

The racemic material was prepared using general procedure E. Prep. TLC was performed on the crude product using 2:1 hexanes:EtOAc to obtain a racemic sample of the title compound. Chiral HPLC was achieved with Chiralpak IC column (75:25 Hexane:IPA) t_R = 10 min (enantiomer 1), 27.5 min (enantiomer 2). Flow rate 0.75 mL min^{-1}.

The enantioselective reaction was achieved according to general procedure F (0.26 mmol). The crude mixture was purified by flash column chromatography (hexanes-EtOAc), to obtain the product as a white solid (61 mg, 98:2 e.r.). The product was found to be inseparable from a catalyst decomposition product (~10% by ^{1}H NMR) by chromatography. To further purify the title compound, the product was recrystallised in a screw cap vial, using 4:1 hexane-EtOAc. The crystals were isolated by careful decantation by pipette, before drying *in vacuo*. This afforded the title compound as colourless crystals, in 69% overall yield (73% mass recovery from crystallisation). The enantiopurity of the recrystallised material was unchanged.

mp: 126-128 ºC

IR (cm^{-1}): 3416, 3054, 2916, 1717, 1667.

^{1}H NMR (400 MHz, CDCl$_3$): δ 7.14 – 7.09 (m, 2H), 7.02 – 6.97 (m, 2H), 6.89 – 6.85 (m, 4H), 5.92 (dd, *J* = 6.9, 1.4 Hz, 1H), 4.07 (s, 1H), 3.20 (dd, *J* = 6.9, 2.3 Hz, 1H), 3.01 (dd, *J* = 8.6, 2.2 Hz, 1H), 2.96 (d, *J* = 8.5 Hz, 1H), 2.79 (d, *J* = 9.5 Hz, 1H), 2.76 (d, *J* = 10.0 Hz, 1H), 2.66 (d, *J* = 14.1 Hz, 1H), 2.60 (d, *J* = 13.6 Hz, 1H), 2.20 (s, 1H), 2.03 (s, 3H), 1.67 (s, 3H), 1.46 (d, *J* = 1.5 Hz, 3H), 1.31 (s, 3H).

^{13}C NMR (126 MHz, CDCl$_3$): δ 213.32, 200.61, 163.47, 163.26, 161.03, 160.82, 148.67, 137.40, 131.93, 131.85, 131.50, 131.39, 131.31, 131.24, 115.48, 115.27, 115.09, 114.88, 74.52, 57.09, 50.61, 50.34, 42.82, 41.42, 40.71, 23.80, 18.93, 15.52, 13.61.

^{19}F NMR (376 MHz, CDCl$_3$): δ -115.70 – -115.79 (m), -115.80 – -115.90 (m).

HRMS (ESI$^+$): *m/z* calcd. for $C_{30}H_{30}F_2O_4Na$ 515.2004; found 515.2004 [M+Na].

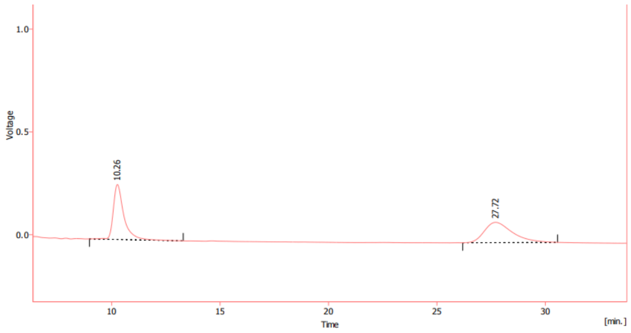

Result Table (Uncal - C:\Clarity\TOM\TDD-485)

	Reten. Time [min]	Area [mV.s]	Height [mV]	Area [%]	Height [%]	W05 [min]
1	10.264	9067.052	267.232	50.3	73.0	0.47
2	27.720	8944.924	98.776	49.7	27.0	1.36
	Total	18011.976	366.007	100.0	100.0	

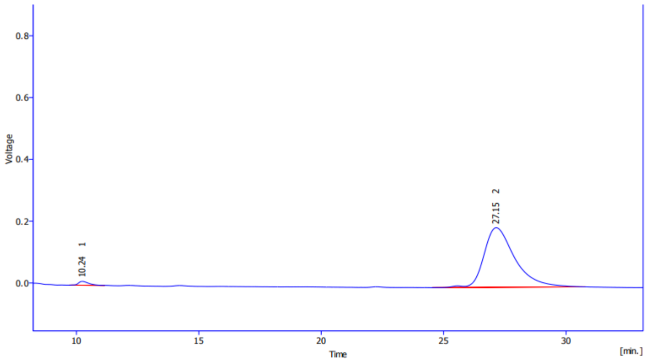

Result Table (Uncal - C:\CLARITY\TOM\COPY OF TDD-484B)

	Reten. Time [min]	Area [mV.s]	Height [mV]	Area [%]	Height [%]	W05 [min]
1	10.240	370.343	12.591	2.1	6.1	0.44
2	27.148	16939.173	192.931	97.9	93.9	1.28
	Total	17309.515	205.522	100.0	100.0	

3,10-dihydroxy-3,10-diisopropyl-6,12-dimethyltricyclo[6.2.2.0^{2,7}]dodeca-5,11-diene-4,9-dione[12] **(3h)**

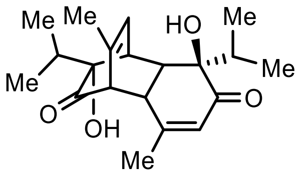

The racemic material was prepared using general procedure E from 2-isopropyl-5-methylphenol (0.41 mmol). Chiral HPLC was achieved with Chiralpak IC column (95:5 Hexane:IPA) t_R = 19 min (enantiomer 1), 23 min (enantiomer 2). Flow rate 0.75 mL min^{-1}

The enantioselective reaction was achieved according to a modified version of general procedure F, in which the reaction was performed at room temperature (20 ºC). The crude mixture was purified by flash column chromatography (hexanes-EtOAc), to obtain the product as a white solid (47.1 mg, 69% yield, >99:1 e.r).

mp: 163-165 ºC.

^{1}H NMR (500 MHz, CDCl$_3$): δ 6.00 – 5.99 (m, 1H), 5.86 – 5.83 (m, 1H), 3.79 (d, *J* = 3.8 Hz, 1H), 3.31 (dd, *J* = 6.8, 1.8 Hz, 1H), 3.27 (dd, *J* = 8.2, 1.5 Hz, 1H), 3.18 (dd, *J* = 2.7, 1.9 Hz, 1H), 3.09 (dd, *J* = 8.2, 2.7 Hz, 1H), 2.23 (s, 1H), 1.97 (s, 3H), 1.78 (hept, *J* = 6.8 Hz, 1H), 1.63 – 1.57 (m, 4H), 0.98 (d, *J* = 6.7 Hz, 3H), 0.86 (d, *J* = 6.9 Hz, 3H), 0.84 (d, *J* = 6.8 Hz, 3H), 0.59 (d, *J* = 6.7 Hz, 3H).

^{13}C NMR (126 MHz, CDCl$_3$): δ 215.01, 201.95, 156.06, 135.96, 126.81, 125.58, 78.37, 77.99, 57.41, 47.41, 42.08, 37.48, 37.41, 32.66, 22.27, 21.52, 16.89, 16.79, 16.45, 16.22.

LCMS (ESI$^+$): *m/z* calcd. for $C_{20}H_{29}O_4$ 333.2; found 333.0 [M+H].

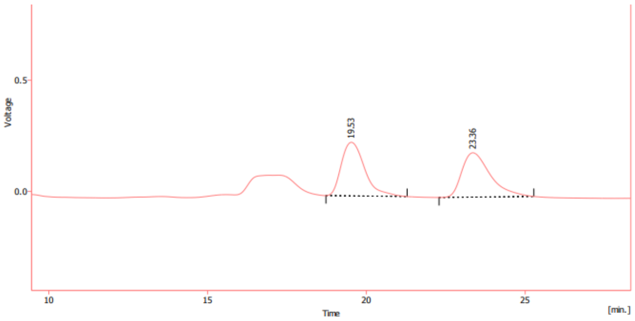

Result Table (Uncal - C:\Clarity\TOM\TDD-317 RAC c)

	Reten. Time [min]	Area [mV.s]	Height [mV]	Area [%]	Height [%]	W05 [min]
1	19.528	12335.359	241.207	49.8	54.7	0.78
2	23.356	12446.823	199.594	50.2	45.3	0.94
	Total	24782.182	440.801	100.0	100.0	

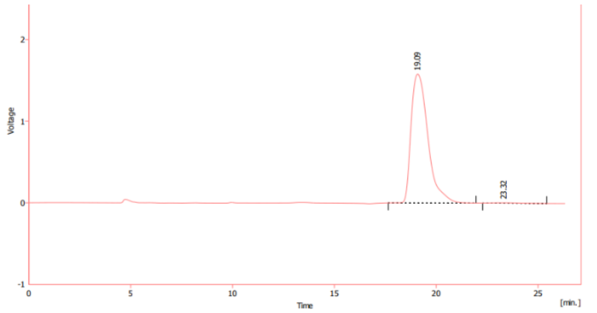

Result Table (Uncal - C:\Clarity\TOM\TDD-321 d)

	Reten. Time [min]	Area [mV.s]	Height [mV]	Area [%]	Height [%]	W05 [min]
1	19.092	90735.598	1578.401	99.6	99.7	0.88
2	23.324	399.159	4.744	0.4	0.3	1.36
	Total	91134.757	1583.145	100.0	100.0	

3,10-dihydroxy-3,6,10,12-tetramethyltricyclo[6.2.2.0^{2,7}]dodeca-5,11-diene-4,9-dione[12] (3k)

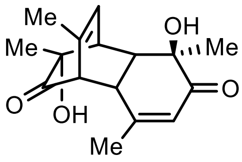

The racemic material was prepared using general procedure E from 2,5-dimethylphenol (0.41 mmol). Prep. TLC was performed on the crude product using 1:1 hexanes:EtOAc to obtain a racemic sample of the title compound. Chiral HPLC was achieved with Chiralpak IC column (75:25 Hexane:IPA) t_R = 13 min (enantiomer 1), 26 min (enantiomer 2). Flow rate 0.75 mL min^{-1}

The enantioselective reaction was achieved according to a modified version of general procedure F, in which the reaction was performed at room temperature (20 ºC). The crude mixture was purified by flash column chromatography (hexanes-EtOAc), to obtain the product as a white solid (22.5 mg, 40% yield, 93:7 e.r.).

mp: 188-190 ºC.

^{1}H NMR (500 MHz, $CDCl_3$): δ 6.02 (s, 1H), 5.89 – 5.85 (dt, *J* = 6.7 Hz, 1.63 Hz, 1H), 3.99 (s, 1H), 3.32 (dd, *J* = 6.8, 1.9 Hz, 1H), 3.18 – 3.14 (m, 3H), 2.23 (s, 1H), 2.00 (d, *J* = 1.3 Hz, 3H), 1.62 (d, *J* = 1.7 Hz, 3H), 1.30 (s, 3H), 1.25 (s, 3H).

^{13}C NMR (126 MHz, $CDCl_3$): δ 212.91, 201.55, 156.47, 136.68, 128.50, 125.06, 73.30, 73.17, 57.11, 44.92, 44.43, 41.31, 32.15, 26.08, 22.57, 21.62.

LCMS (ESI$^+$): *m/z* calcd. for $C_{16}H_{20}O_4$ 277.1 found 276.9 [M+H].

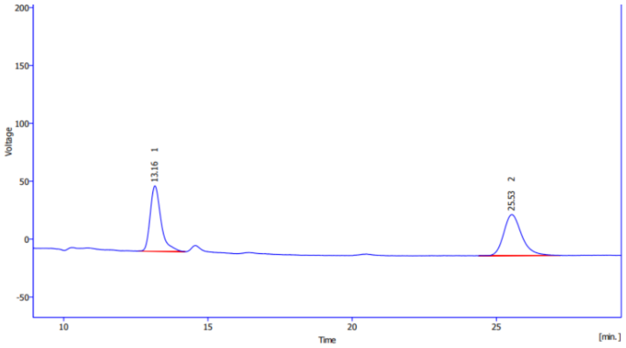

Result Table (Uncal - C:\CLARITY\TOM\TDD-371 RAC)

	Reten. Time [min]	Area [mV.s]	Height [mV]	Area [%]	Height [%]	W05 [min]
1	13.160	1473.995	56.564	48.9	61.5	0.38
2	25.528	1537.688	35.422	51.1	38.5	0.65
	Total	3011.683	91.986	100.0	100.0	

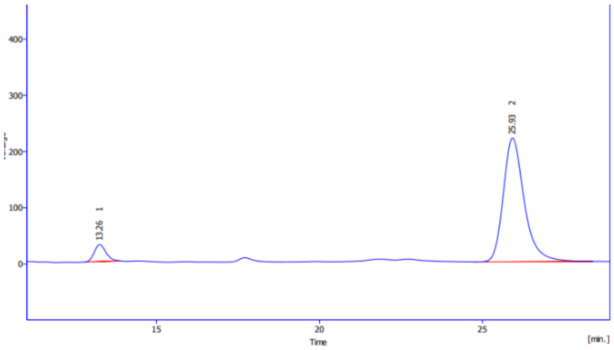

Result Table (Uncal - C:\CLARITY\TOM\TDD-354 REDO)

	Reten. Time [min]	Area [mV.s]	Height [mV]	Area [%]	Height [%]	W05 [min]
1	13.256	749.786	30.101	7.1	12.0	0.39
2	25.928	9816.403	219.950	92.9	88.0	0.66
	Total	10566.189	250.051	100.0	100.0	

(+)-biscarvacrol (+)-3i[12,14]

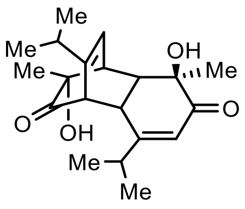

The racemic material was prepared using general procedure E from 2-methyl-5-isopropylphenol (61.5 mg, 0.41 mmol). The crude mixture was purified by automated flash column chromatography (0 → 80% EtOAc in hexane) to yield the title compound as an off-white solid (40.3 mg, 59%). Chiral HPLC was achieved with Chiralpak IC column (75:25 Hexane:IPA) t_R = 8 min (enantiomer 1), 14 min (enantiomer 2). Flow rate 0.75 mL min^{-1}

The enantioselective reaction was achieved according to general procedure F. The crude mixture was purified by flash column chromatography (hexanes-EtOAc), to obtain the product as a white solid (41.8 mg, 61% yield, 99:1 e.r.).

mp: 132-134 ºC.

^{1}H NMR (500 MHz, $CDCl_3$): δ 5.97 (s, 1H), 5.86 (dt, *J* = 6.9, 1.5 Hz, 1H), 4.04 (s, 1H), 3.36 (dd, *J* = 6.9, 2.4 Hz, 1H), 3.23 (dd, *J* = 8.6, 2.0 Hz, 1H), 3.16 (t, *J* = 1.9 Hz, 1H), 3.12 (dd, *J* = 8.7, 2.4 Hz, 1H), 2.49 (hept, *J* = 7.4 Hz, 1H), 2.32 (s, 1H), 1.91 – 1.80 (m, 1H), 1.25 (s, 3H), 1.23 (s, 3H), 1.14 (d, *J* = 7.0 Hz, 3H), 1.12 (s, 3H), 0.90 (d, *J* = 6.7 Hz, 3H), 0.86 (d, *J* = 6.9 Hz, 3H).

^{13}C NMR (126 MHz, $CDCl_3$): δ 212.45, 202.10, 166.66, 145.86, 126.27, 120.16, 73.64, 73.11, 56.01, 44.80, 42.24, 41.10, 33.45, 33.07, 32.44, 26.05, 23.13, 20.92, 20.25, 19.42.

LCMS (ESI$^+$): *m/z* calcd. for $C_{20}H_{29}O_4$ 333.2; found 333.1 [M+H].

$[\alpha]_D^{25}$ = + 50.9º (c = 0.11, $CHCl_3$).

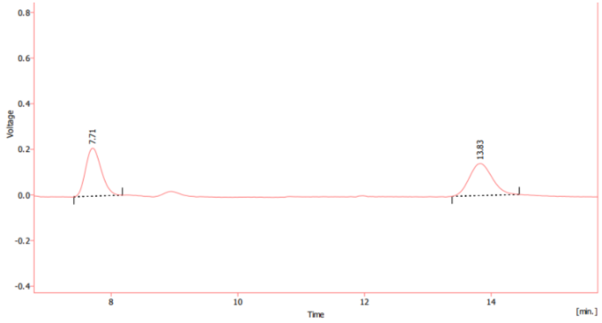

Result Table (Uncal - C:\Clarity\TOM\TDD-191b)

	Reten. Time [min]	Area [mV.s]	Height [mV]	Area [%]	Height [%]	W05 [min]
1	7.708	3602.321	210.139	50.5	60.0	0.27
2	13.832	3523.955	140.210	49.5	40.0	0.40
	Total	7126.276	350.349	100.0	100.0	

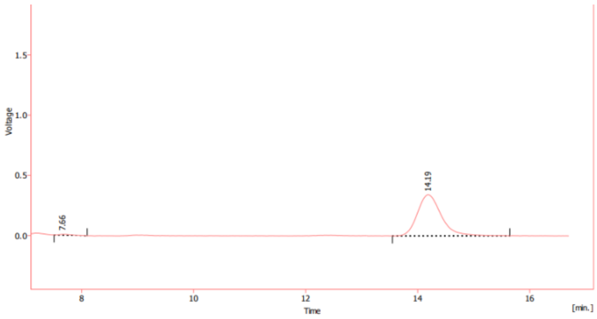

Result Table (Uncal - C:\Clarity\TOM\TDD-262b)

	Reten. Time [min]	Area [mV.s]	Height [mV]	Area [%]	Height [%]	W05 [min]
1	7.664	118.949	7.002	1.2	2.0	0.21
2	14.188	10086.624	341.816	98.8	98.0	0.44
	Total	10205.573	348.818	100.0	100.0	

(−)-biscarvacrol (−)-3i[12,14]

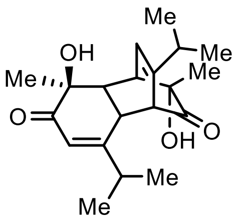

The enantioselective reaction was achieved according to general procedure F, using catalyst *ent*-**6a**. The crude mixture was purified by flash column chromatography (hexanes-EtOAc), to obtain the product as a white solid (45.6 mg, 66% yield, 97.5:2.5 e.r.). Analytical data was identical to that described for (+)-biscarvacrol.

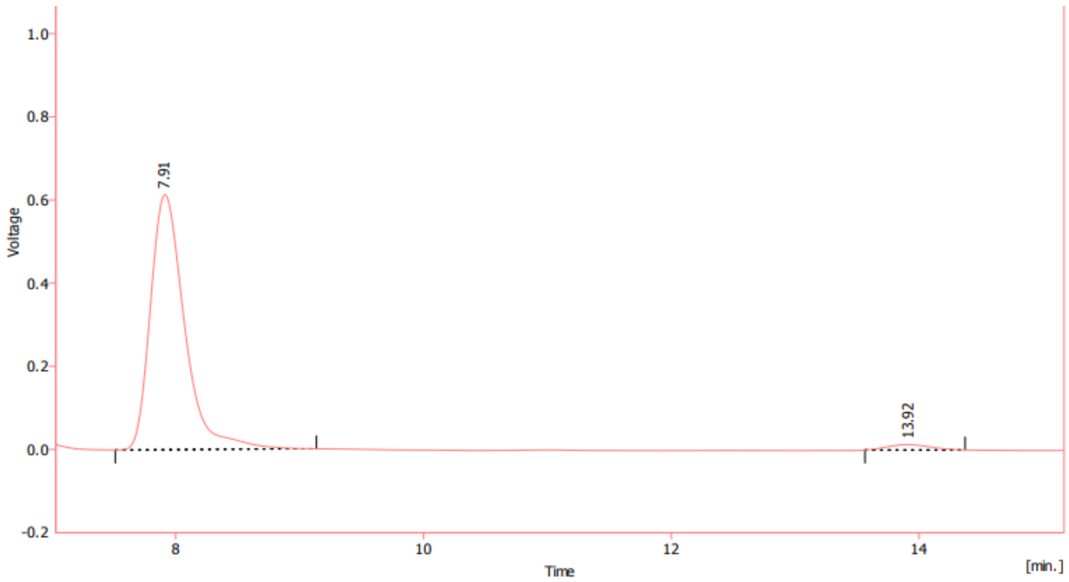

Result Table (Uncal - C:\Clarity\TOM\TDD-557B)

	Reten. Time [min]	Area [mV.s]	Height [mV]	Area [%]	Height [%]	W05 [min]
1	7.912	11775.706	613.753	97.5	97.9	0.29
2	13.916	302.888	12.944	2.5	2.1	0.38
	Total	12078.594	626.697	100.0	100.0	

3,10-Dihydroxy-6,12-dimethoxy-3,10,-dimethyltricyclo[6.2.2.0^{2,7}]dodeca-5,11-diene-4,9-dione[15] **(3j)**

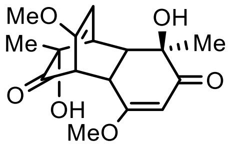

The racemic material was prepared using general procedure E from 2-methyl-5-methoxyphenol (0.41 mmol). Preparative TLC (1:1 CH_2Cl_2:EtOAc) was performed on the crude reaction mixture after thermolysis at 70 ºC for 1 h, in order to obtain a racemic sample. Chiral HPLC was achieved with Chiralpak IC column (75:25 Hexane:IPA) t_R = 15 min (enantiomer 1), 35 min (enantiomer 2). Flow rate 0.75 mL min^{-1}

The enantioselective reaction was achieved according to a modified version of general procedure F, in which the tertiary amine catalyst **8** was employed (10 mol%). Conversion of the SM was confirmed by TLC (3:1 hexane:EtOAc). LCMS analysis could identify the major product as the non-dimerized *o*-quinol, and so the neat crude reaction mixture was heated to 70 ºC for 1 h.[15] The desired product could then be identified by LCMS, and the crude mixture was subsequently purified by flash column chromatography (CH_2Cl_2-EtOAc)* to afford the product as a white solid (33.4 mg, 53% yield, 98:2 e.r.).

**other chromatography eluents were found to contaminate the product with residual iminium catalyst.*

mp: 209-211 ºC (decomp.)

^{1}H NMR (400 MHz, CDCl$_3$): δ 5.43 (s, 1H), 4.84 (dd, *J* = 7.5, 2.4 Hz, 1H), 4.11 (s, 1H), 3.69 (s, 3H), 3.43 (s, 3H), 3.36 (dd, *J* = 7.5, 2.0 Hz, 1H), 3.33 (t, *J* = 2.6 Hz, 1H), 3.25 (dd, *J* = 8.8, 2.7 Hz, 1H), 3.16 (dd, *J* = 8.8, 2.0 Hz, 1H), 1.33 (s, 3H), 1.30 (s, 3H).

^{13}C NMR (101 MHz, CDCl$_3$): δ 209.99, 200.39, 172.31, 154.61, 100.15, 99.85, 72.69, 72.11, 56.56, 56.30, 55.34, 43.53, 42.39, 40.56, 32.60, 25.72.

LCMS (ESI$^+$): *m/z* calcd. for $C_{16}H_{21}O_6$ 309.1 found 309.0 [M+H].

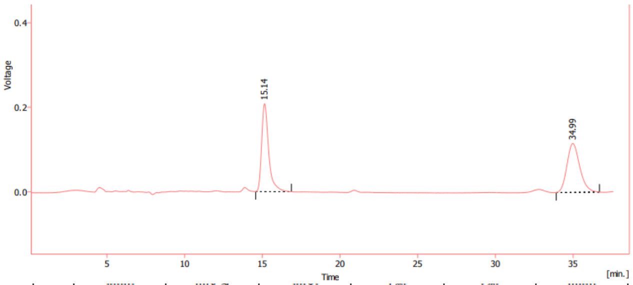

	[illegible]	[illegible]	[illegible]	[illegible]	[illegible]	[illegible]
1	15.144	6555.778	208.018	49.8	64.3	0.46
2	34.988	6614.726	115.543	50.2	35.7	0.88
	Total	13170.504	323.562	100.0	100.0	

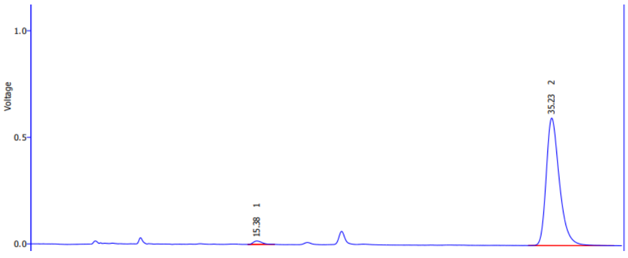

Result Table (Uncal - C:\CLARITY\TOM\TDD-391B)

	Reten. Time [min]	Area [mV.s]	Height [mV]	Area [%]	Height [%]	W05 [min]
1	15.376	654.666	15.982	1.7	2.6	0.64
2	35.228	36893.812	597.992	98.3	97.4	0.92
	Total	37548.477	613.973	100.0	100.0	

5,8-di-tert-butyl-3,10-dihydroxy-3,10-dimethyltricyclo[6.2.2.$0^{2,7}$]dodeca-5,11-diene-4,9-dione (3l)

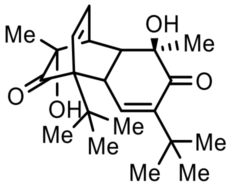

The racemic material was prepared using general procedure E from 2-*tert*-butyl-6-methyl-phenol (0.41 mmol). Prep. TLC was performed on the crude product using 1:1 hexanes:EtOAc to obtain a racemic sample of the title compound. Chiral HPLC was achieved with Chiralpak IC column (75:25 Hexane:IPA) t_R = 7.5 min (enantiomer 1), 23 min (enantiomer 2). Flow rate 0.75 mL min^{-1}

The enantioselective reaction was achieved according to general procedure F. The crude mixture was purified by flash column chromatography (hexanes-EtOAc), to obtain the product as a pale yellow solid (37 mg, 50% yield, 92.5:7.5 e.r.).

mp: 109-111 ºC.

IR (cm^{-1}): 3521, 3445, 2964, 2963, 1713, 1684.

^{1}H NMR (500 MHz, $CDCl_3$): δ 6.35 – 6.29 (m, 2H), 5.92 (dd, J = 8.7, 0.7 Hz, 1H), 4.20 (br s, 1H), 3.34 (dt, J = 6.9, 1.6 Hz, 1H), 3.24 (ddd, J = 8.3, 5.3, 0.7 Hz, 1H), 3.04 (dd, J = 8.3, 1.8 Hz, 1H), 2.61 (br s, 1H), 1.26 – 0.97 (m, 24H).

^{13}C NMR (126 MHz, $CDCl_3$): 217.41, 203.81, 145.83, 135.91, 135.64, 129.64, 74.86, 74.65, 63.36, 43.90, 43.23, 42.85, 35.07, 33.57, 31.21, 29.24, 28.70, 25.10.

HRMS (ESI^+): *m/z* calcd. for $C_{22}O_4H_{32}Na$ 383.2193; found 383.2191 [M+Na].

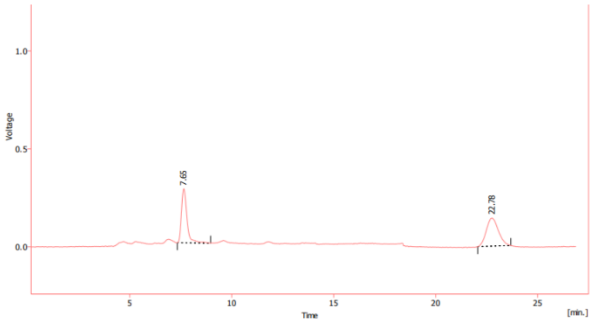

Result Table (Uncal - C:\Clarity\TOM\TDD-349 rac b)

	Reten. Time [min]	Area [mV.s]	Height [mV]	Area [%]	Height [%]	W05 [min]
1	7.652	5223.895	275.678	47.1	65.8	0.28
2	22.776	5874.033	143.006	52.9	34.2	0.65
	Total	11097.928	418.684	100.0	100.0	

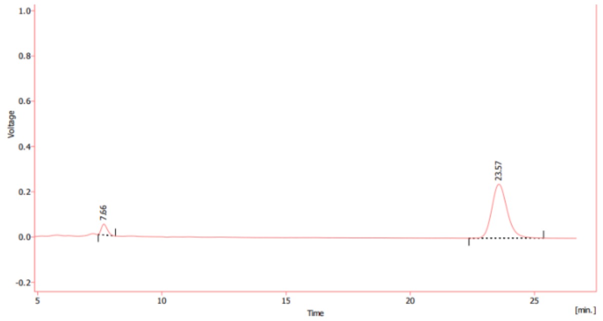

Result Table (Uncal - C:\Clarity\TOM\TDD-364)

	Reten. Time [min]	Area [mV.s]	Height [mV]	Area [%]	Height [%]	W05 [min]
1	7.664	803.453	48.064	7.3	16.8	0.26
2	23.568	10243.464	238.494	92.7	83.2	0.66
	Total	11046.917	286.558	100.0	100.0	

Dearomatization of 2-methyl-6-benzylphenol

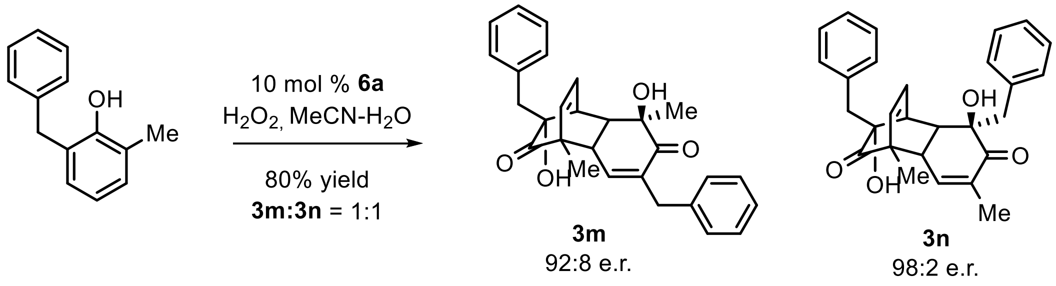

The racemic material was prepared using general procedure E from 2-methyl-6-benzylphenol (0.41 mmol). The mixture of bicyclo[2.2.2]octenone products was isolated by column chromatography (0 → 60% EtOAc in hexane). Preparative TLC was then performed (9:1 CH_2Cl_2/EtOAc) to separate the two products in order to obtain HPLC samples. Chiral HPLC was achieved with Chiralpak IC column (75:25 Hexane:IPA), flow rate 0.75 mL min^{-1}.

Product **3m**: t_R = 12.5 min (enantiomer 1), 15.0 min (enantiomer 2).

Product **3n**: t_R = 14.0 min (enantiomer 1), 16.0 min (enantiomer 2).

The enantioselective reaction was achieved according to general procedure F, where the crude mixture was purified by flash column chromatography (hexanes:EtOAc) to obtain a mixture of the two products as a white solid (69.9 mg, 80% yield, 1:1 isomeric ratio). The two products were then separated by a further chromatographic purification (0 → 5% EtOAc in CH_2Cl_2). The characterisation of hetero-dimer **3m** was in agreement with literature.[16]

(3R)-5,10-dibenzyl-3,10-dihydroxy-3,8-dimethyltricyclo[6.2.2.0^{2,7}]dodeca-5,11-diene-4,9-dione[16] **(3m)**

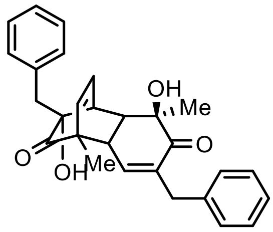

mp: 130-132 ºC.

^{1}H NMR (400 MHz, CDCl$_3$): δ 7.34 – 7.26 (m, 4H), 7.24 – 7.18 (m, 1H), 7.17 – 7.10 (m, 4H), 6.33 (dd, *J* = 8.0, 6.8 Hz, 1H), 6.13 (d, *J* = 4.4 Hz, 1H), 5.54 (dd, *J* = 8.1, 1.2 Hz, 1H), 3.85 (s, 1H), 3.58 (d, *J* = 15.3 Hz, 1H), 3.52 (d, *J* = 15.3 Hz, 2H), 3.22 (dt, *J* = 6.7, 1.7 Hz, 1H), 3.12 (dd, *J* = 8.3, 1.9 Hz, 1H), 2.88 (dd, *J* = 8.3, 4.5 Hz, 1H), 2.78 (d, *J* = 14.0 Hz, 1H), 2.72 (d, *J* = 13.9 Hz, 1H), 2.25 (s, 1H), 1.27 (s, 3H), 1.13 (s, 3H).

^{13}C NMR (101 MHz, CDCl$_3$): δ 213.58, 202.20, 140.03, 138.45, 135.30, 135.00, 133.50, 130.49, 129.08, 128.69, 128.59, 127.30, 126.71, 75.25, 73.82, 54.19, 44.80, 43.33, 42.93, 42.03, 36.42, 31.41, 15.79.

LCMS (ESI$^+$): *m/z* calcd. for $C_{28}H_{29}O_4$ 429.2 found 429.1 [M+H].

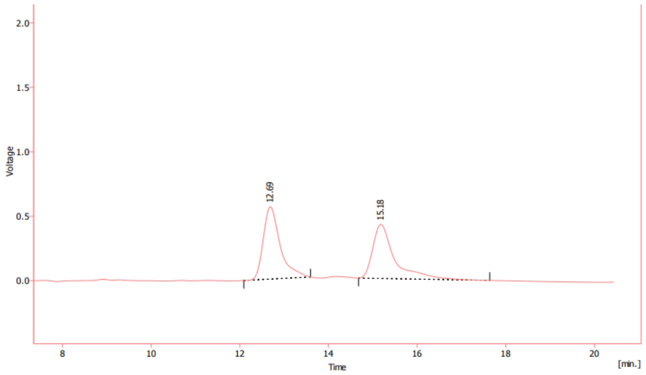

Result Table (Uncal - C:\Clarity\TOM\TDD-468het(2))

	Reten. Time [min]	Area [mV.s]	Height [mV]	Area [%]	Height [%]	W05 [min]
1	12.688	14864.447	560.528	50.8	57.2	0.38
2	15.184	14378.853	419.762	49.2	42.8	0.44
	Total	29243.300	980.289	100.0	100.0	

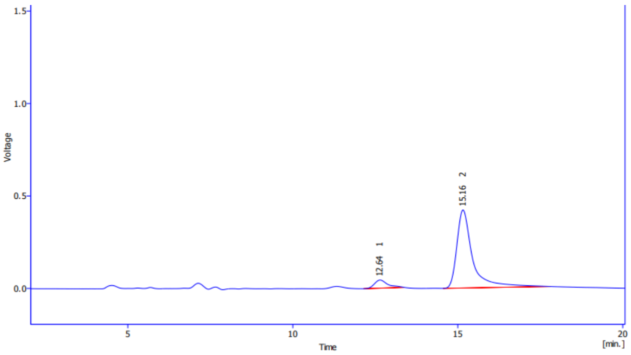

Result Table (Uncal - C:\CLARITY\TOM\TDD-462B")

	Reten. Time [min]	Area [mV.s]	Height [mV]	Area [%]	Height [%]	W05 [min]
1	12.636	1224.757	43.987	8.0	9.4	0.40
2	15.156	14053.102	422.070	92.0	90.6	0.44
	Total	15277.859	466.057	100.0	100.0	

(3R)-3,10-dibenzyl-3,10-dihydroxy-5,8-dimethyltricyclo[6.2.2.0^{2,7}]dodeca-5,11-diene-4,9-dione (3n)

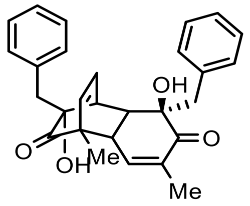

mp: 67-69 ºC

IR (cm^{-1}): 3455, 2923, 2852, 1722, 1680.

^{1}H NMR (400 MHz, CDCl$_3$): δ 7.33 – 7.25 (m, 3H), 7.18 – 7.13 (m, 5H), 6.98 – 6.94 (m, 2H), 6.36 (dd, *J* = 4.2, 1.4 Hz, 1H), 6.31 (dd, *J* = 8.0, 6.8 Hz, 1H), 5.58 (dd, *J* = 8.1, 1.1 Hz, 1H), 3.87 (s, 1H), 3.29 (dd, *J* = 8.2, 1.8 Hz, 1H), 3.24 (dt, *J* = 6.8, 1.7 Hz, 1H), 3.03 – 2.98 (m, 1H), 2.93 – 2.83 (m, 2H), 2.81 – 2.70 (m, 2H), 2.28 (s, 1H), 1.74 (t, *J* = 1.5 Hz, 3H), 1.42 (s, 3H).

^{13}C NMR (126 MHz, CDCl$_3$): δ 213.88, 201.51, 139.18, 136.79, 135.32, 135.30, 135.02, 133.61, 130.51, 129.95, 128.60, 128.21, 127.32, 127.16, 77.40, 75.46, 54.26, 50.72, 45.23, 43.35, 42.57, 41.94, 16.38, 15.94.

HRMS (ESI$^+$): *m/z* calcd. for $C_{28}H_{28}O_4$ 451.1880; found 451.1880 [M+Na].

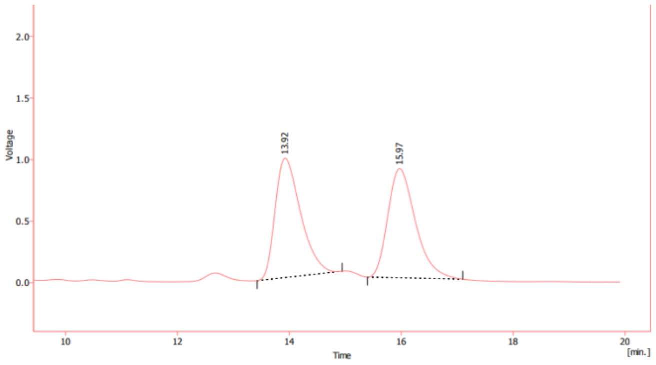

Result Table (Uncal - C:\Clarity\TOM\TDD-468homo(2))

	Reten. Time [min]	Area [mV.s]	Height [mV]	Area [%]	Height [%]	W05 [min]
1	13.924	30681.131	968.712	50.1	52.2	0.50
2	15.972	30521.282	886.616	49.9	47.8	0.53
	Total	61202.413	1855.328	100.0	100.0	

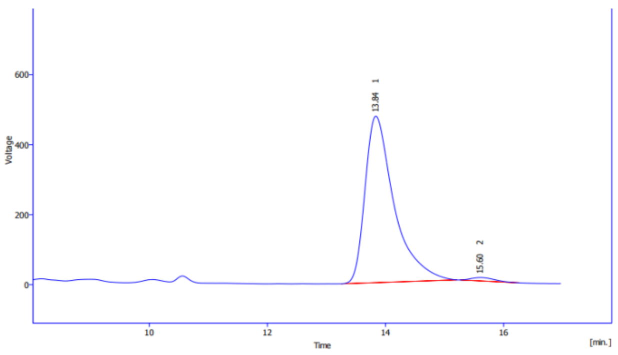

Result Table (Uncal - C:\CLARITY\TOM\TDD-462A')

	Reten. Time [min]	Area [mV.s]	Height [mV]	Area [%]	Height [%]	W05 [min]
1	13.836	16285.551	475.935	98.4	98.0	0.49
2	15.604	266.362	9.764	1.6	2.0	0.42
	Total	16551.913	485.699	100.0	100.0	

Synthesis of *ent*-bis(2,6-xylenol) using Amine pre-catalyst 8

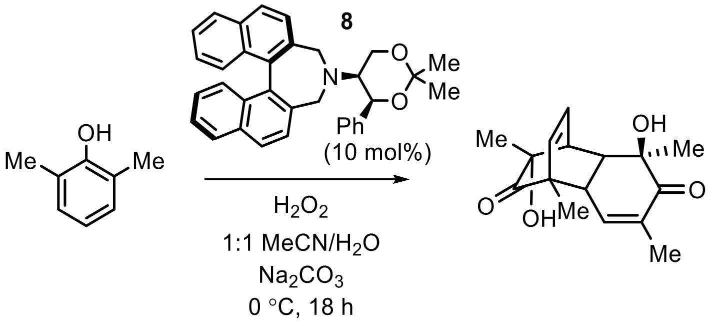

The reaction was carried out in an identical manner to general procedure F, employing amine **8** instead of **6a**. To a stirred mixture of the phenol (0.41 mmol), amine **8** (10 mol%), and Na_2CO_3 (2.46 mmol) in 1:1 MeCN-H_2O (2.5 mL) at 0 ºC, H_2O_2 (126 µL, 30% aq. soln) was added. The resulting mixture was then stirred overnight at 0 ºC, before quenching with $Na_2S_2O_3$. The reaction was then diluted with CH_2Cl_2 and extracted with CH_2Cl_2 (2 x 15 mL). The combined organics were then dried over $MgSO_4$, filtered, and concentrated *in vacuo*. Purification was achieved using flash column chromatography (0 → 80% EtOAc in hexane) to afford the product as a white solid (43 mg, 75% yield, 95:5 e.r.). Spectral data was identical to that when using general procedure E or F.

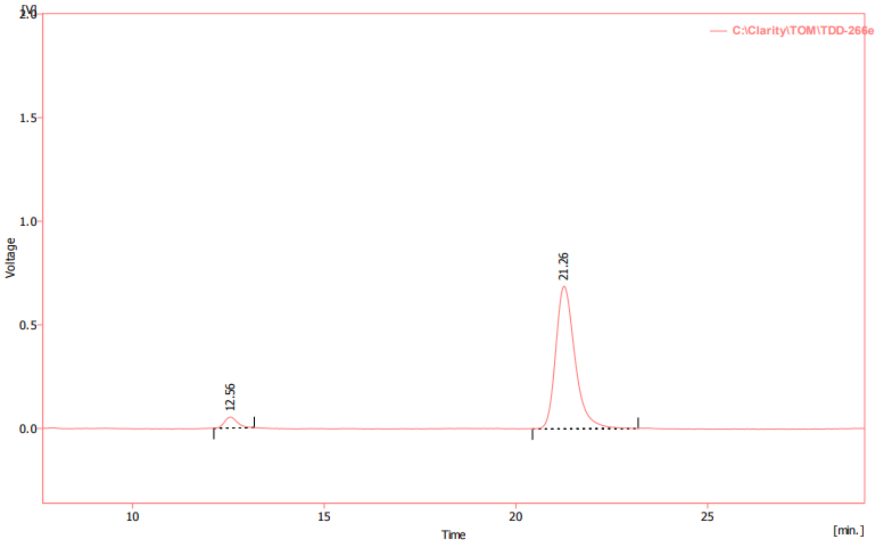

Result Table (Uncal - C:\Clarity\TOM\TDD-266e)

	Reten. Time [min]	Area [mV.s]	Height [mV]	Area [%]	Height [%]	W05 [min]
1	12.556	1221.657	52.413	4.8	7.1	0.36
2	21.256	23967.718	688.150	95.2	92.9	0.52
	Total	25189.375	740.564	100.0	100.0	

Gram Scale Synthesis of *ent*-bis(2,6-xylenol) with reduced loading

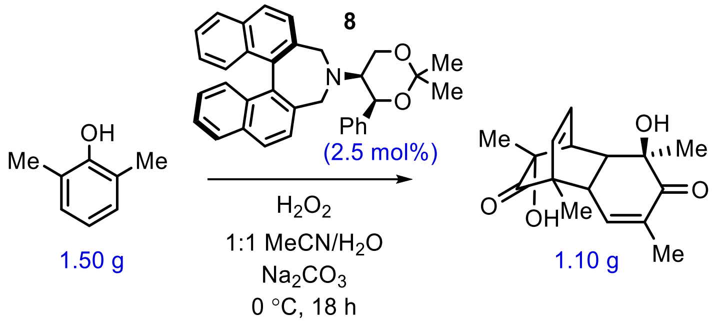

Na_2CO_3 (6.5 g, 61.3 mmol) was added to a stirred solution of 2,6-dimethylphenol (1.5 g, 12.3 mmol), and the amine pre-catalyst **8** (148 mg, 0.31 mmol) in 1:1 MeCN-H_2O (75 mL). The resulting suspension was cooled to 0 ºC, before adding H_2O_2 (3.78 mL, 30% aq. solution) dropwise, over approximately 15 minutes. The reaction mixture was then stirred rapidly overnight at 0 ºC, before quenching with $Na_2S_2O_3$. MeCN was then removed *in vacuo*, before extracting with CH_2Cl_2 (3 x 100 mL). The combined organics were then dried over $MgSO_4$, filtered, and concentrated to afford the crude product. Purification was achieved using flash column chromatography (0 → 50% EtOAc in hexane), to afford the product as a white solid (1.1 g, 65%, 92.5:7.5 e.r.). Spectral data was identical to that reported from the standard procedure.

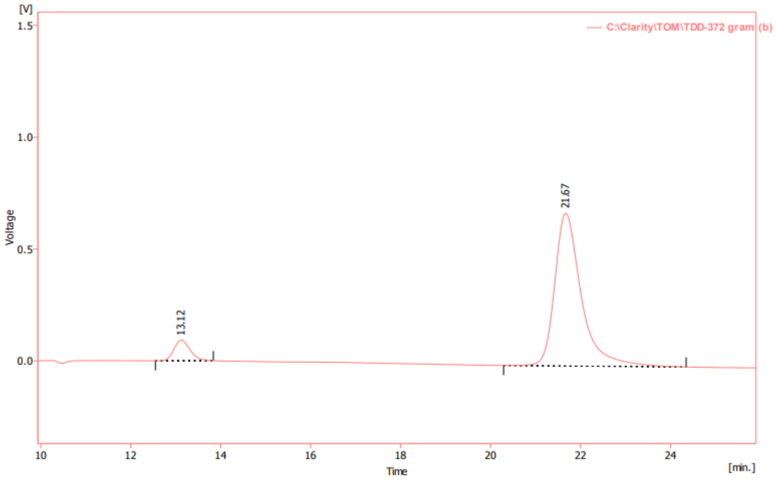

Result Table (Uncal - C:\Clarity\TOM\TDD-372 gram (b))

	Reten. Time [min]	Area [mV.s]	Height [mV]	Area [%]	Height [%]	W05 [min]
1	13.124	2291.110	91.696	7.5	11.8	0.38
2	21.672	28155.955	682.488	92.5	88.2	0.59
	Total	30447.065	774.184	100.0	100.0	

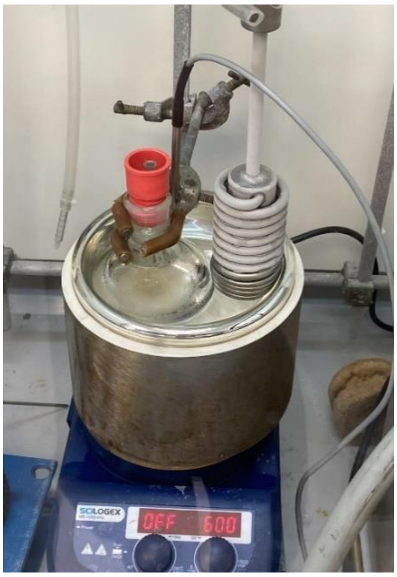

Figure S5. Set-up of the gram-scale dearomatization.

Retro-[4+2]/[4+2] reactions of *ent*-bis(2,6-xylenol)

6-(4-chlorophenyl)-3-hydroxy-1,3-dimethylbicyclo[2.2.2]octa-5,7-dien-2-one (9)

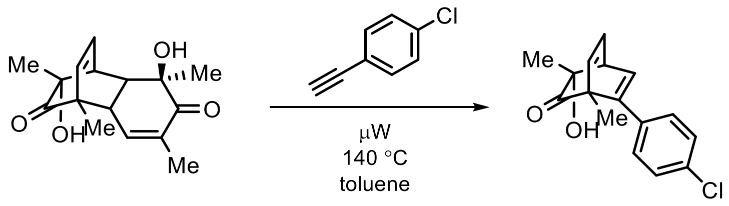

ent-bis(2,6-xylenol) (30 mg, 0.11 mmol, 92.5:7.5 e.r.), 4-chlorophenyl acetylene (218 mg, 1.6 mmol) and dry toluene (2 mL) were added to a microwave vial at room temperature (20 ºC). The mixture was then purged with argon, before placing in the microwave reactor. The reaction mixture was irradiated for a total of 4 h, maintaining a temperature of 140 ºC. The reaction was then cooled, before removing toluene *in vacuo*. Purification was then achieved using flash column chromatography (0 → 60% EtOAc in hexane) to afford the product as a colourless oil (59 mg, 98% yield, 92:8 e.r.). Chiral HPLC was achieved with Chiralpak IC column (75:25 Hexane:IPA) t_R = 6.8 min (enantiomer 1), 7.6 min (enantiomer 2). Flow rate 0.75 mL min^{-1}.

IR (cm^{-1}): 3461, 2975, 2933, 1721.
^{1}H NMR (400 MHz, CDCl$_3$): δ 7.29 – 7.22 (m, 2H), 7.02 – 6.97 (m, 2H), 6.61 – 6.53 (m, 1H), 6.44 (d, *J* = 6.2 Hz, 1H), 6.16 (dd, *J* = 7.2, 1.9 Hz, 1H), 3.83 (td, *J* = 6.3, 1.9 Hz, 1H), 1.36 (s, 3H), 1.34 (s, 3H).
^{13}C NMR (101 MHz, CDCl$_3$): δ 206.37, 144.20, 135.90, 135.72, 135.45, 134.06, 133.61, 129.99, 128.32, 69.60, 58.51, 48.44, 27.07, 14.58.
HRMS (ESI$^+$): *m/z* calcd. for $C_{16}H_{15}ClO_2Na$ 295.1305; found 295.1304 [M+Na].

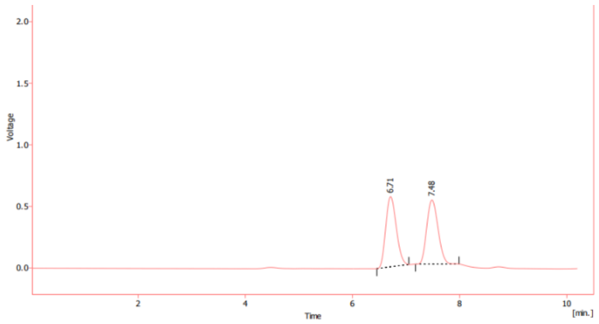

Result Table (Uncal - C:\Clarity\TOM\TDD-431)

	Reten. Time [min]	Area [mV.s]	Height [mV]	Area [%]	Height [%]	W05 [min]
1	6.848	10352.808	807.105	51.1	52.0	0.21
2	7.636	9923.279	746.201	48.9	48.0	0.22
	Total	20276.087	1553.306	100.0	100.0	

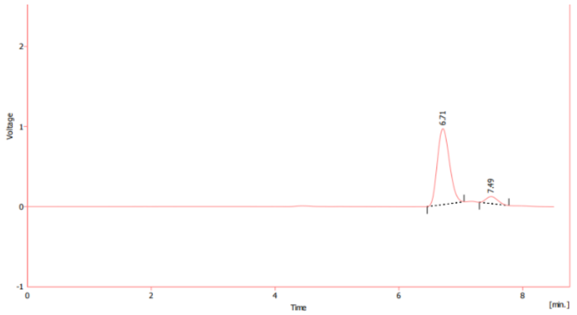

Result Table (Uncal - C:\Clarity\TOM\TDD-426b)

	Reten. Time [min]	Area [mV.s]	Height [mV]	Area [%]	Height [%]	W05 [min]
1	6.712	12887.478	946.915	91.9	91.6	0.22
2	7.488	1134.873	86.821	8.1	8.4	0.21
	Total	14022.351	1033.735	100.0	100.0	

1-hydroxy-1,3,6,7-tetramethyl-1,2,4a,5,8,8a-hexahydronaphthalen-2-one (10)

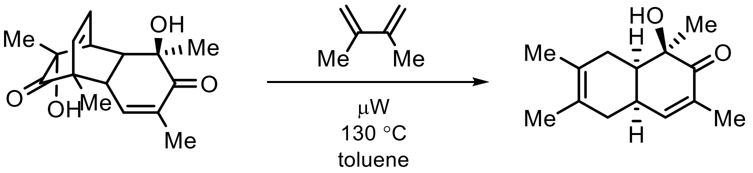

ent-bis(2,6-xylenol) (30 mg, 0.11 mmol, 92.5 : 7.5 e.r.), 1,3-dimethyl butadiene (0.18 mL, 1.6 mmol) and dry toluene (2 mL) were added to a microwave vial at room temperature (20 ºC). The mixture was then purged with argon, before placing in the microwave reactor. The reaction mixture was irradiated for a total of 4 h, maintaining a temperature of 130 ºC. The reaction was then cooled, before removing toluene *in vacuo*. Purification was then achieved using flash column chromatography (0 → 40% EtOAc in hexane) to afford the product as a colourless oil (45 mg, 93%, 91:9 e.r.). Chiral HPLC was achieved with Chiralpak IC column (75:25 Hexane:IPA) t_R = 8.5 min (enantiomer 1), 13 min (enantiomer 2). Flow rate 0.75 mL min^{-1}.

IR (cm^{-1}): 3498, 2975, 2920, 2894, 1671.

^{1}H NMR (400 MHz, $CDCl_3$): δ 6.36 – 6.33 (m, 1H), 3.61 (s, 1H), 3.00 – 2.85 (m, 1H), 2.48 – 2.37 (m, 1H), 2.34 – 2.24 (m, 1H), 2.08 (dd, *J* = 18.0, 6.7 Hz, 1H), 1.97 (d, *J* = 17.3 Hz, 1H), 1.79 (dd, *J* = 2.7, 1.4 Hz, 3H), 1.63 (s, 3H), 1.53 (s, 3H), 1.36 (s, 3H).

^{13}C NMR (101 MHz, $CDCl_3$): δ 203.49, 150.77, 132.53, 125.55, 123.75, 77.07, 43.94, 37.60, 33.95, 28.84, 24.52, 19.13, 19.08, 15.75.

HRMS (ESI$^+$): *m/z* calcd. for $C_{14}H_{20}O_2Na$ 243.1356; found 243.1356 [M+Na].

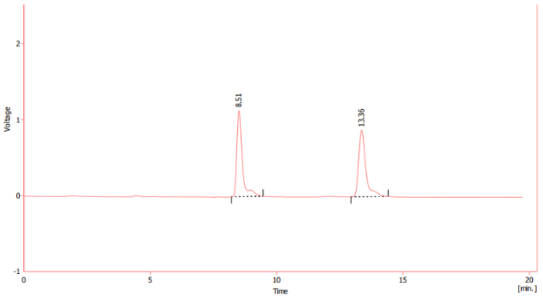

Result Table (Uncal - C:\Clarity\TOM\TDD-425b)

	Reten. Time [min]	Area [mV.s]	Height [mV]	Area [%]	Height [%]	W05 [min]
1	8.512	17176.992	1130.916	50.0	56.3	0.21
2	13.364	17153.187	878.069	50.0	43.7	0.28
	Total	34330.179	2008.985	100.0	100.0	

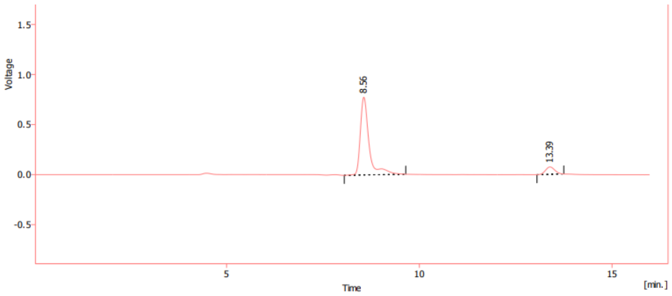

Result Table (Uncal - C:\Clarity\TOM\TDD-420b)

	Reten. Time [min]	Area [mV.s]	Height [mV]	Area [%]	Height [%]	W05 [min]
1	8.560	12175.495	777.024	90.9	91.3	0.22
2	13.388	1221.534	73.672	9.1	8.7	0.27
	Total	13397.028	850.695	100.0	100.0	

3-hydroxy-7-(4-methoxyphenyl)-1,3-dimethylbicyclo[2.2.2]oct-5-en-2-one (11)

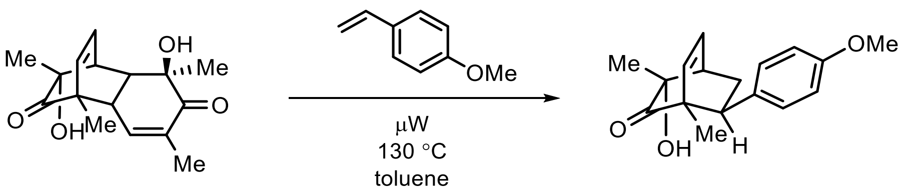

ent-bis(2,6-xylenol) (30 mg, 0.11 mmol, 92.5 : 7.5 e.r.), 4-methoxy styrene (0.14 mL, 1.10 mmol) and dry toluene (2 mL) were added to a microwave vial at room temperature (20 ºC). The mixture was then purged with argon, before placing in the microwave reactor. The reaction mixture was irradiated for 1 h, maintaining a temperature of 130 ºC. The reaction was then cooled, before removing toluene *in vacuo*. Purification was then achieved using flash column chromatography (0 → 40% EtOAc in hexane) to afford the product as a white solid, in quantitative yield (*ca.* 10:1 regiomeric ratio of inseparable isomers, 91.5:8.5 e.r.). Chiral HPLC was achieved with Chiralpak IC column (75:25 Hexane:IPA) t_R = 7.6 min (enantiomer 1), 9 min (enantiomer 2). Flow rate 0.75 mL min^{-1}.

mp: 117 ºC.
IR (cm^{-1}): 3426, 2962, 2930, 1721, 1612.
^{1}H NMR (400 MHz, $CDCl_3$): δ 7.08 – 7.02 (m, 2H), 6.81 – 6.75 (m, 2H), 6.63 (dd, *J* = 8.1, 6.9 Hz, 1H), 5.80 – 5.76 (m, 1H), 3.78 (s, 3H), 3.01 – 2.95 (m, 1H), 2.94 – 2.78 (m, 2H), 2.46 (s, 1H), 1.64 – 1.54 (m, 1H), 1.32 (s, 3H), 0.89 (s, 3H).
^{13}C NMR (101 MHz, $CDCl_3$): δ 214.16, 158.61, 136.66, 135.38, 131.63, 129.78, 113.66, 72.31, 55.36, 53.06, 45.61, 43.78, 32.30, 26.32, 15.88.
HRMS (ESI$^+$): *m/z* calcd. for $C_{17}H_{20}O_3Na$ 295.1305; found 295.1304 [M+Na].

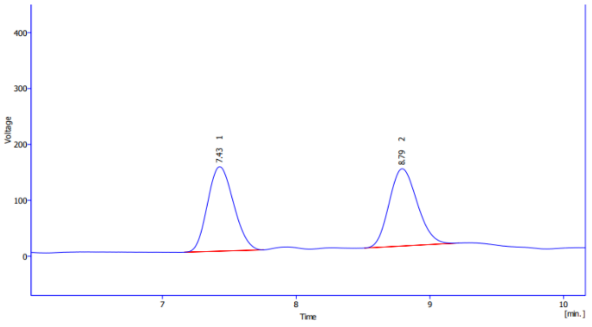

Result Table (Uncal - C:\CLARITY\TOM\TDD-417)

	Reten. Time [min]	Area [mV.s]	Height [mV]	Area [%]	Height [%]	W05 [min]
1	7.428	2044.689	151.187	50.4	52.3	0.22
2	8.792	2011.904	138.128	49.6	47.7	0.24
	Total	4056.593	289.314	100.0	100.0	

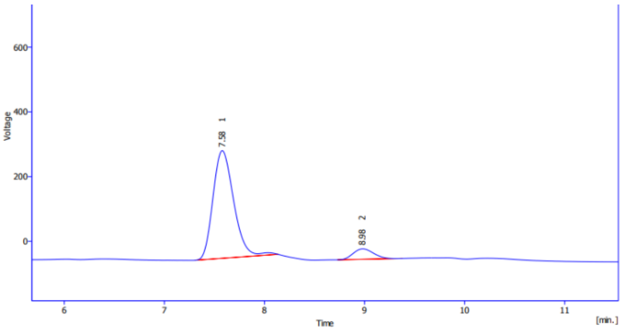

Result Table (Uncal - C:\CLARITY\TOM\TDD-415)

	Reten. Time [min]	Area [mV.s]	Height [mV]	Area [%]	Height [%]	W05 [min]
1	7.580	4782.049	332.587	91.3	91.2	0.22
2	8.980	457.532	32.225	8.7	8.8	0.23
	Total	5239.581	364.812	100.0	100.0	

Unsuccessful substrates

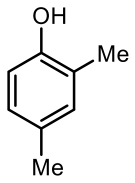

low yielding
para-dearomatization

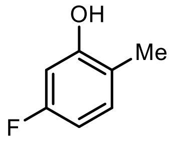

no conversion

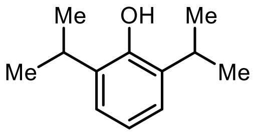

no conversion

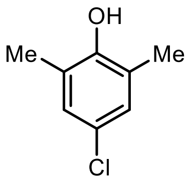

messy/complex mixture

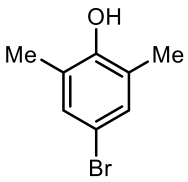

messy/complex mixture

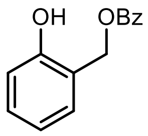

messy/complex mixture

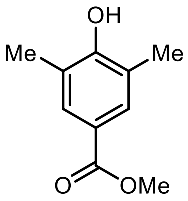

undesired, unidentified product

Mechanistic Studies

i) Direct HRMS observation of the oxaziridinium ion

Na_2CO_3 (1 mg) followed by Oxone (6 mg) was added to a solution of Binapthylazepinium catalyst **6a** (2 mg) in 1:1 CH_2Cl_2-H_2O (1 mL), at room temperature (20 ºC). The solution was vigorously mixed for around 30 seconds, and then the two layers were allowed to partition. A sample was then taken from the organic layer, diluted with MeOH, and immediately analysed by ESI-HRMS. This allowed for the observation of m/z=500.2220, which corresponds to the oxaziridinium ion **12**. Isotopic patterns agreed well with the predicted spectrum.

(a)

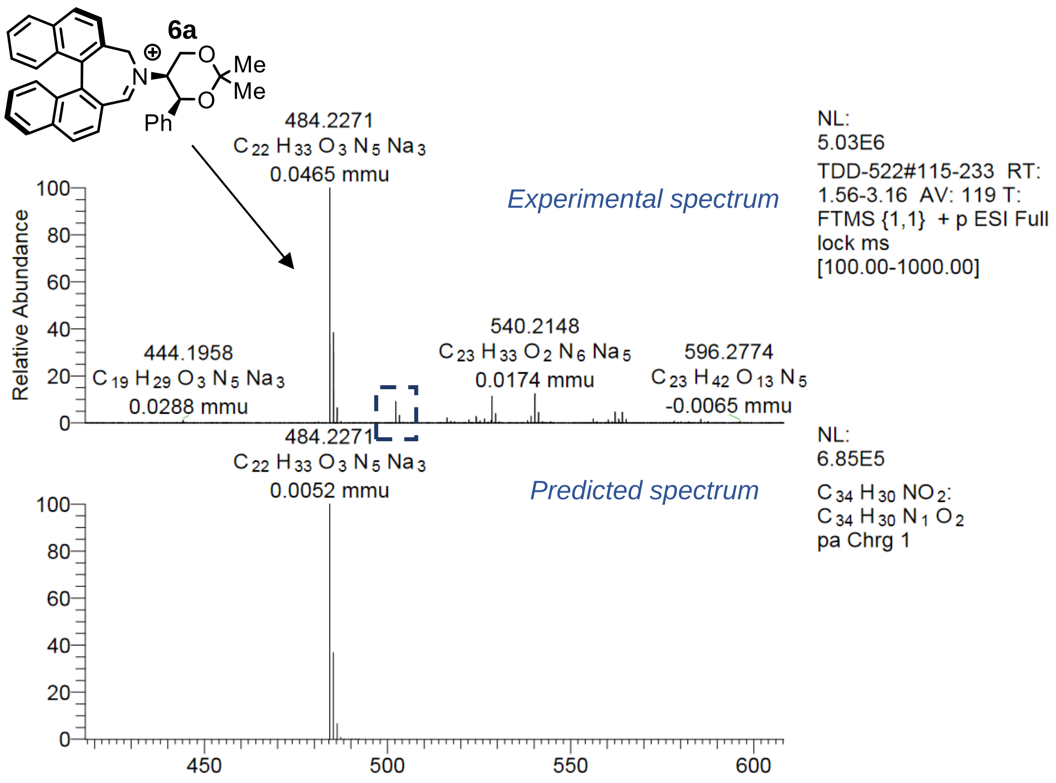

(b)

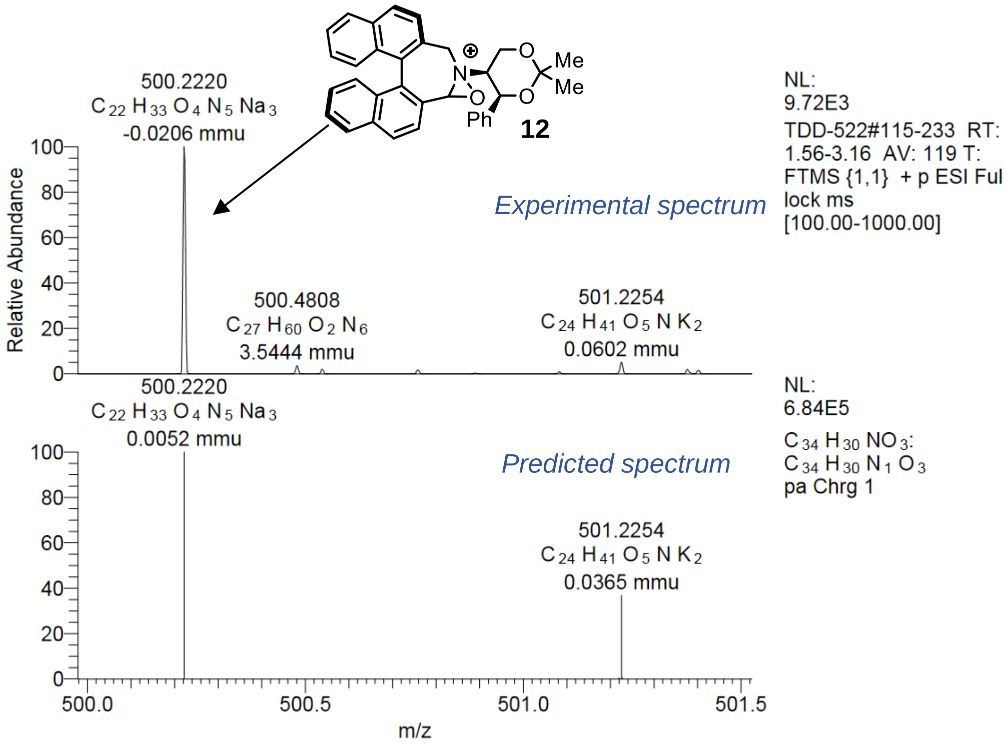

Figure S6. Direct HRMS analysis of oxidant + catalyst mixture, (a) iminium ion (b) zoomed in view of the spectrum to show oxaziridinium **12** (b). Both species show predicted m/z values as well as isotopologue patterns.

ii) Stoichiometric dearomatization reaction using an isolated oxaziridinium salt

3,4-Dihydroisoquinoline[17] (S12)

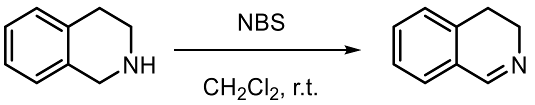

NBS (1.47 g, 8.3 mmol), was added to 1,2,3,4-tetrahydroisoquinoline (0.94 mL, 7.5 mmol) in CH_2Cl_2 (40 mL), at 0 °C. The resulting solution was then allowed to warm to room temperature (20 ºC), and stirred for a further 1 h. 30% NaOH (10 mL) was then added, and the resulting mixture was stirred for 1 h. The organic layer was then separated, before acidifying with 10% HCl (2 x 20 mL). The acidic aqueous extracts were then basified using aqueous NH_3, before extracting with CH_2Cl_2 (3 x 30 mL). The combined organic extracts were then dried over $MgSO_4$, filtered, and concentrated *in vacuo*. Purification of the crude product was achieved by flash column chromatography (100% EtOAc) to afford the title compound as a pale-yellow oil (752 mg, 76%).

^{1}H NMR (400 MHz, $CDCl_3$): δ 8.31 (t, *J* = 2.2 Hz, 1H), 7.32 (td, *J* = 7.2, 1.9 Hz, 1H), 7.29 – 7.21 (m, 2H), 7.12 (dd, *J* = 7.3, 0.4 Hz, 1H), 3.74 (ddd, *J* = 8.0, 6.3, 2.2 Hz, 2H), 2.75 – 2.68 (m, 2H).
^{13}C NMR (101 MHz, $CDCl_3$): δ 160.39, 136.40, 131.10, 128.59, 127.48, 127.25, 127.14, 47.47, 25.10.
LCMS (ESI$^+$): *m/z* calcd. for C_9H_9N 132.1; found 132.0 [M+H].

3,4-dihydroisoquinoline 1,2-oxide[18] (S13)

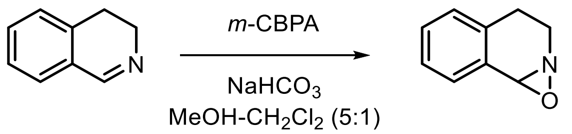

To a stirred solution of 3,4-dihydroisoquinoline (650 mg, 5.0 mmol) in $MeOH-CH_2Cl_2$ (5:1 v/v, 120 mL), *m*-CPBA (1.34 g, ~70%, 5.4 mmol) was slowly added at 0 °C. $NaHCO_3$ (416 mg, 5.0 mmol) was then added, before allowing the reaction mixture to warm to room temperature (20 ºC). The reaction was stirred at r.t. for 20 h, before diluting with CH_2Cl_2 (50 mL). The organics were washed with sat. $Na_2S_2O_3$ solution (2 x 30 mL), followed by sat. $NaHCO_3$ (30 mL), and finally with brine (30 mL). The organic layer was then separated, dried over $MgSO_4$, filtered, and concentrated *in vacuo*. The crude product was then purified by flash column chromatography (100% EtOAc) to afford the oxaziridine as a pale-yellow oil (252 mg, 35%).

^{1}H NMR (400 MHz, $CDCl_3$): δ 7.51 (dd, *J* = 7.4, 1.2 Hz, 1H), 7.36 (td, *J* = 7.5, 1.4 Hz, 1H), 7.30 (t, *J* = 7.4 Hz, 1H), 7.13 (d, *J* = 7.4 Hz, 1H), 4.94 (s, 1H), 3.93 – 3.82 (m, 1H), 2.95 – 2.80 (m, 2H), 2.54 – 2.44 (m, 1H).
^{13}C NMR (101 MHz, $CDCl_3$): δ 135.38, 130.13, 129.88, 129.24, 127.97, 126.78, 74.44, 48.77, 23.82.
LCMS (ESI$^+$): *m/z* calcd. for C_9H_9NO 148.1; found 148.0 [M+H].

2-Methyl-4,8b-dihydro-3H-oxazireno[3,2-a]isoquinolin-2-ium tetrafluoroborate[19] (14)

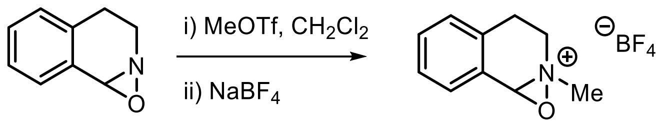

To a stirred solution of oxaziridine **S13** (92.5 mg, 0.63 mmol) in dry CH_2Cl_2 (5 mL), MeOTf (0.11 mL, 0.97 mmol) was added dropwise, at 0 °C. The reaction was then stirred at 0 °C for a further 30 min, before warming to room temperature (20 ºC). $NaBF_4$ (75 mg, 0.68 mmol), in the minimum volume of acetone, was then slowly added, before stirring at r.t. for 15 min. Solvents were then removed *in vacuo*, and the resulting solid was triturated in Et_2O, to afford the oxaziridinium salt as an off-white solid (127 mg, 81%). The oxaziridinium salt was found to partially decompose into the corresponding iminium salt (~ 18% by ^{1}H NMR), which can be identified by both LC-MS and ^{1}H NMR.

^{1}H NMR (400 MHz, CD_3CN): δ 7.83 (d, *J* = 7.6 Hz, 1H), 7.64 (t, *J* = 7.6 Hz, 1H), 7.50 (t, *J* = 7.4 Hz, 1H), 7.38 (d, *J* = 7.6 Hz, 1H), 6.19 (s, 1H), 4.35 (dd, *J* = 13.3, 6.2 Hz, 1H), 4.35 (dd, *J* = 13.3, 6.2 Hz, 1H), 3.88 (tt, *J* = 10.0, 5.0 Hz, 1H), 3.76 (s, 3H), 3.15 (ddd, *J* = 18.6, 12.5, 6.2 Hz, 1H), 3.02 (dd, *J* = 16.9, 4.7 Hz, 1H).
^{13}C NMR (101 MHz, CD_3CN): δ 134.64, 134.31, 133.29, 129.91, 129.08, 122.61, 84.95, 54.19, 51.15, 24.96.
LCMS (ESI$^+$): *m/z* calcd. for $C_{10}H_{12}NO^+$ 162.1; found 162.0 [M$^+$]

Stoichiometric dearomatization using 14 to form 3a

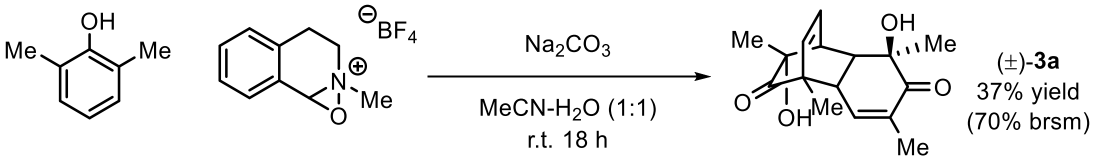

To a stirred solution of 2,6-dimethylphenol (45 mg, 0.34 mmol) and Na_2CO_3 (109 mg, 1.03 mmol) in H_2O (1.25 mL), a solution of freshly prepared oxaziridinium tetrafluoroborate **14** (115 mg, ~82%, 0.38 mmol) in MeCN (1.25 mL) was added dropwise, at 0 °C. The reaction was then warmed to r.t., and stirred for 20 h. The reaction was then quenched with $Na_2S_2O_3$, before diluting with CH_2Cl_2 (10 mL), and extracting with CH_2Cl_2 (2 x 10 mL). The combined organic extracts were then dried over $MgSO_4$, filtered, and concentrated *in vacuo*. The crude product was then purified by automated flash column chromatography (0 → 80% EtOAc) to afford **3a** as a white solid (17.5 mg, 37% yield, 70% brsm). Analytical data was identical to that reported from general procedure E or F.

X-ray Crystallography

The crystal structures of **3a** and **3g** were solved and refined routinely and further details are presented in Tables S3 – S6 and in the deposited cif files. CCDC 2152267 & 2152268 contain the supplementary crystallographic data for this paper. These data can be obtained free of charge from The Cambridge Crystallographic Data Centre via www.ccdc.cam.ac.uk/structures.

For 3a: This is a redetermination of the structure published in the CSD with REFCODE: EHMNAP10.[20] That structure, determined in 1973, had an *R* factor of 6.6% and was measured at room temperature. This re-determination is of significantly superior quality with an *R* factor of 3.3% and was measured at 100 K.

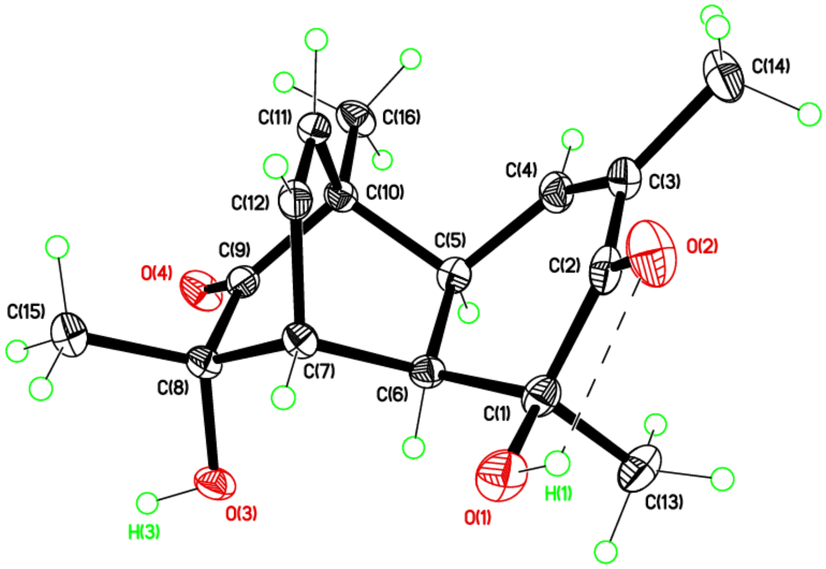

Figure S7. Displacement ellipsoid plot of **3a** at the 50% probability level showing an intramolecular S(5) H-bond.[21]

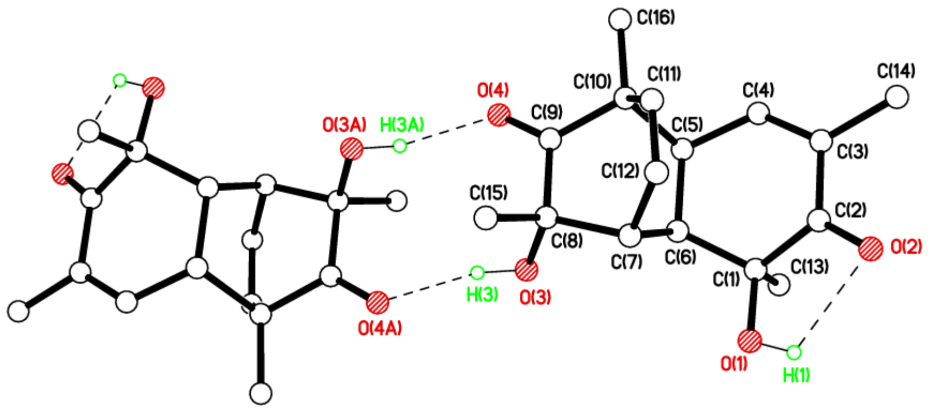

Figure S8. Hydrogen bonded centro-symmetric dimer pairs in the crystal structure of **3a** with S(5) and $R^2_2(10)$ graph set motifs. Most H atoms omitted for clarity.

Table S3

*Experimental details for **3a***

Crystal data	
Chemical formula	$C_{16}H_{20}O_4$
M_r	276.32
Crystal system, space group	Monoclinic, $P2_1/n$
Temperature (K)	100
a, *b*, *c* (Å)	8.36115 (6), 11.87163 (7), 14.50532 (11)
β (°)	104.3438 (7)
V (Å^3)	1394.92 (2)
Z	4
Radiation type	Cu $K\alpha$
μ (mm^{-1})	0.77
Crystal size (mm^3)	0.36 × 0.28 × 0.12
Data collection	
Diffractometer	Rigaku 007HF equipped with Varimax confocal mirrors and an AFC11 goniometer and HyPix 6000 detector
Absorption correction	Multi-scan *CrysAlis PRO* 1.171.41.105a (Rigaku Oxford Diffraction, 2021). Empirical absorption correction using spherical harmonics, implemented in SCALE3 ABSPACK scaling algorithm.
T_{min}, T_{max}	0.881, 1.000
No. of measured, independent and observed [$I > 2\sigma(I)$] reflections	18199, 2495, 2469
R_{int}	0.014
$(\sin \theta/\lambda)_{max}$ (Å^{-1})	0.602
Refinement	
$R[F^2 > 2\sigma(F^2)]$, $wR(F^2)$, S	0.033, 0.077, 1.03
No. of reflections	2495
No. of parameters	261
H-atom treatment	All H-atom parameters refined
$\Delta\rangle_{max}$, $\Delta\rangle_{min}$ (e Å^{-3})	0.28, -0.17

Computer programs: *CrysAlis PRO* 1.171.41.105a (Rigaku OD, 2021), SHELXT-2018/2 (Sheldrick, 2015), *SHELXL2018*/3 (Sheldrick, 2018), Bruker *SHELXTL*.

Table S4

*Hydrogen-bond geometry (Å, º) for (**3a**)*

D—H···*A*	*D*—H	H···*A*	*D*···*A*	*D*—H···*A*
O1—H1···O2	0.82 (3)	2.23 (3)	2.6621 (13)	114 (2)
C6—H6···O3	0.976 (13)	2.279 (13)	2.7915 (13)	111.7 (9)
O3—H3···O4^{i}	0.889 (19)	1.912 (19)	2.7838 (11)	166.3 (16)

Symmetry code: (i) -*x*+1, -*y*, -*z*+1.

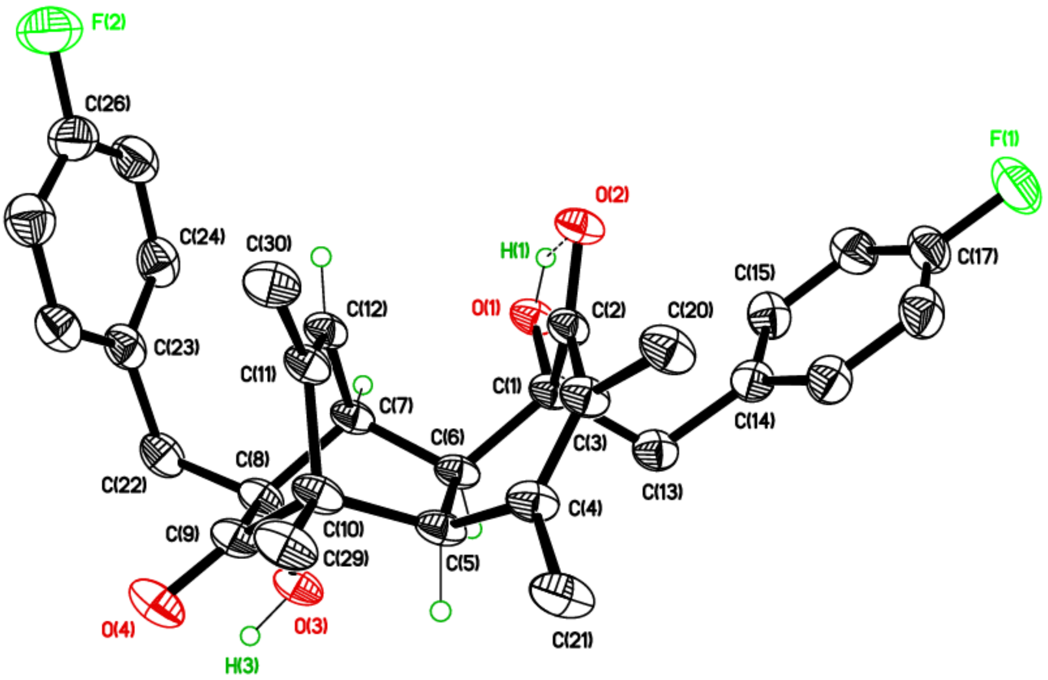

Figure S9. Displacement ellipsoid plot of **3g** at the 50% probability level showing an intramolecular S(5) H-bond. Most H atoms omitted for clarity.

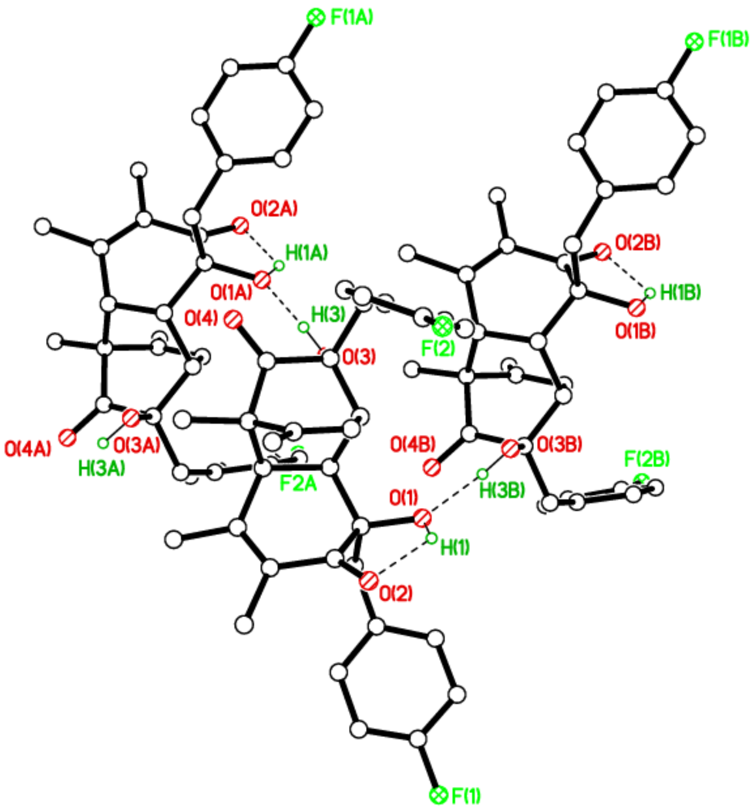

Figure S10. H-bonded, C(7) zig-zag chains and S(5) motifs in the crystal structure of **3g**. Most H atoms omitted for clarity.

For **3g**: The key point is that the absolute structure was reliably determined.

Table S5

*Experimental details for **3g***

Crystal data	
Chemical formula	$C_{30}H_{30}F_2O_4$
M_r	492.54
Crystal system, space group	Monoclinic, $P2_1$
Temperature (K)	100
a, *b*, *c* (Å)	11.67041 (19), 8.92920 (13), 12.4767 (2)
β (°)	108.8488 (18)
V (Å^3)	1230.44 (4)
Z	2
Radiation type	Cu *K*α
μ (mm^{-1})	0.80
Crystal size (mm^3)	0.17 × 0.09 × 0.03

Data collection	
Diffractometer	Rigaku 007HF equipped with Varimax confocal mirrors and an AFC11 quarter χ goniometer and HyPix 6000 detector
Absorption correction	Analytical. *CrysAlis PRO* 1.171.41.122a (Rigaku Oxford Diffraction, 2021) Analytical numeric absorption correction using a multifaceted crystal model based on expressions derived by R.C. Clark & J.S. Reid. (Clark, R. C. & Reid, J. S. (1995). Acta Cryst. A51, 887-897) Empirical absorption correction using spherical harmonics, implemented in SCALE3 ABSPACK scaling algorithm.
T_{min}, T_{max}	0.955, 0.990
No. of measured, independent and observed [$I > 2\sigma(I)$] reflections	52195, 4624, 4415
R_{int}	0.036
$(\sin \theta/\lambda)_{max}$ (Å^{-1})	0.612
Refinement	
$R[F^2 > 2\sigma(F^2)]$, $wR(F^2)$, S	0.027, 0.071, 1.06
No. of reflections	4624
No. of parameters	337
No. of restraints	1
H-atom treatment	H atoms treated by a mixture of independent and constrained refinement
$\Delta\rangle_{max}$, $\Delta\rangle_{min}$ (e Å^{-3})	0.12, -0.12
Absolute structure	Flack x determined using 1952 quotients [(I+)-(I-)]/[(I+)+(I-)] (Parsons, Flack and Wagner, Acta Cryst. B69 (2013) 249-259).
Absolute structure parameter	-0.02 (6)

Computer programs: *CrysAlis PRO* 1.171.41.122a (Rigaku OD, 2021), SHELXT-2018/2 (Sheldrick, 2015), *SHELXL2018*/3 (Sheldrick, 2018), Bruker *SHELXTL*.

Table S6

*Hydrogen-bond geometry (Å, º) for (**3g**)*

D—H···*A*	*D*—H	H···*A*	*D*···*A*	*D*—H···*A*
O3—H3···O1^{i}	0.88 (3)	1.98 (3)	2.7954 (19)	153 (3)
O1—H1···O2	0.84 (3)	2.04 (3)	2.5877 (18)	122 (3)
C13—H13*B*···O4^{ii}	0.99	2.57	3.476 (2)	151
C18—H18···F2^{iii}	0.95	2.58	3.402 (3)	145
C27—H27···F1^{iii}	0.95	2.57	3.429 (3)	151

Symmetry codes: (i) -*x*, *y*+1/2, -*z*+1; (ii) -*x*, *y*-1/2, -*z*+1; (iii) -*x*+1, *y*+1/2, -*z*+1.

NMR Spectra

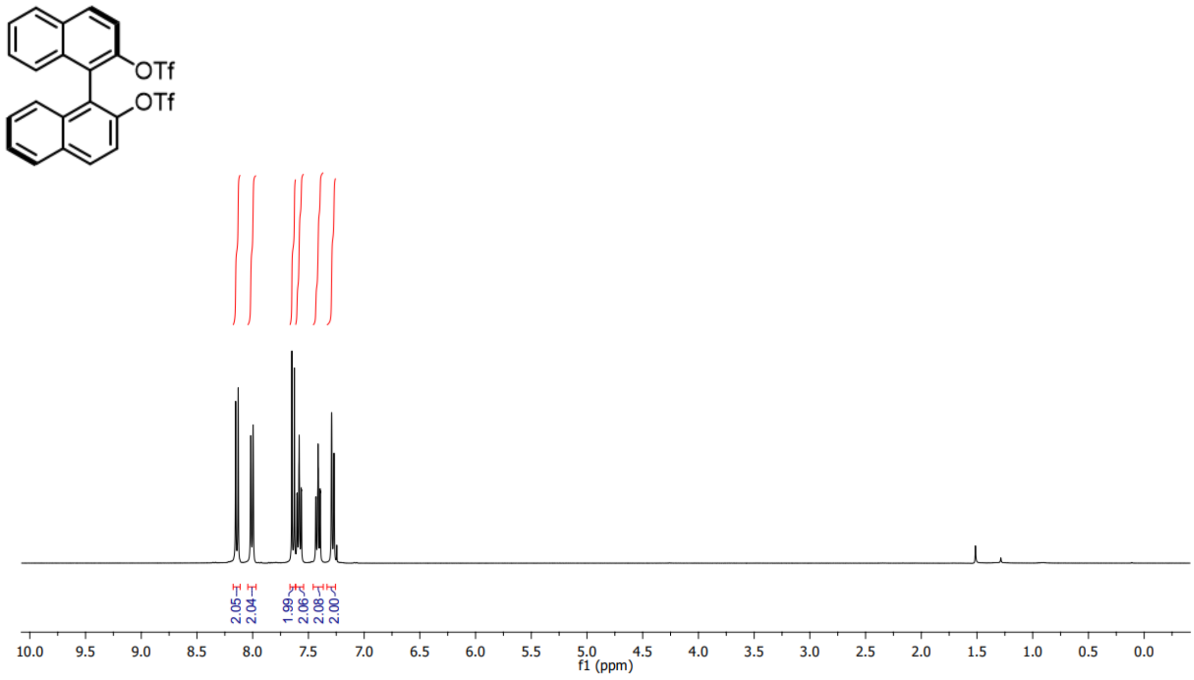
OTf
OTf
2.05
2.04
1.99
2.06
2.08
2.00
10.0 9.5 9.0 8.5 8.0 7.5 7.0 6.5 6.0 5.5 5.0 4.5 4.0 3.5 3.0 2.5 2.0 1.5 1.0 0.5 0.0
f1 (ppm)

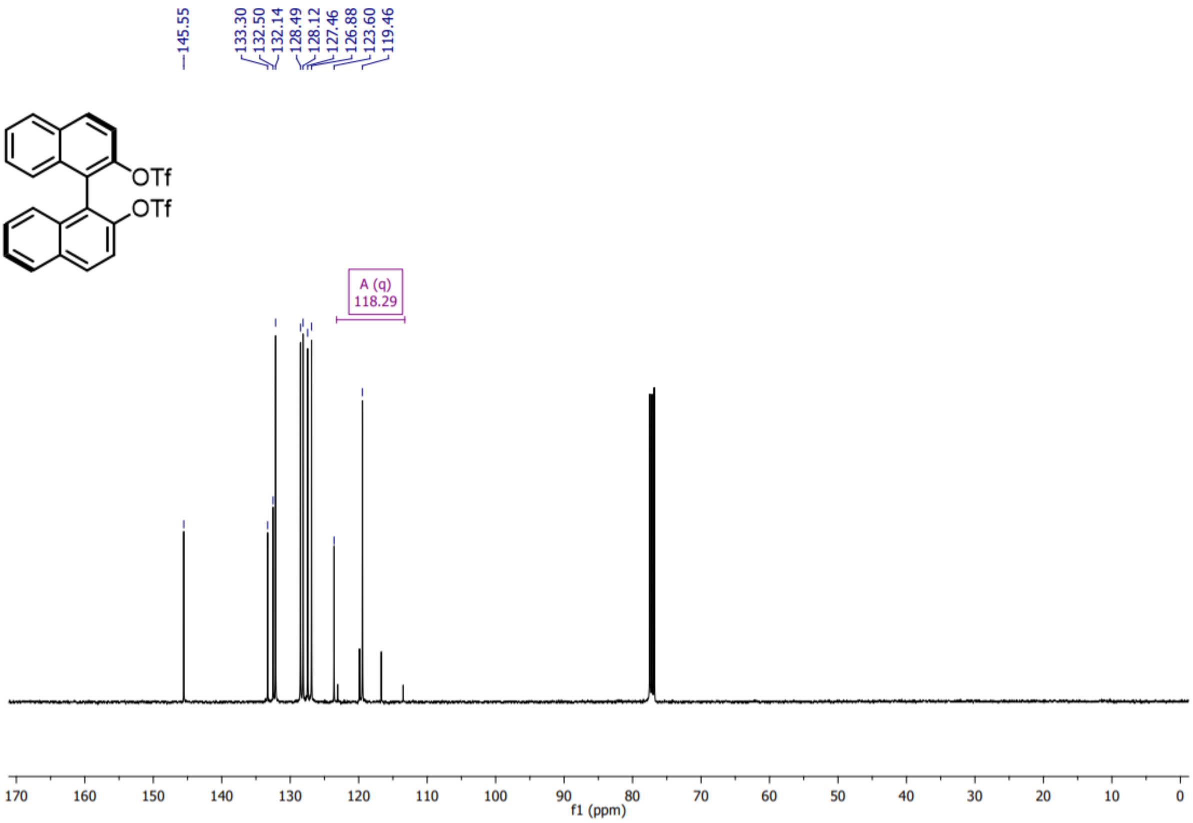
145.55
133.30
132.50
132.14
128.49
128.12
127.46
126.88
123.60
119.46
OTf
OTf
A (q)
118.29
170 160 150 140 130 120 110 100 90 80 70 60 50 40 30 20 10 0
f1 (ppm)

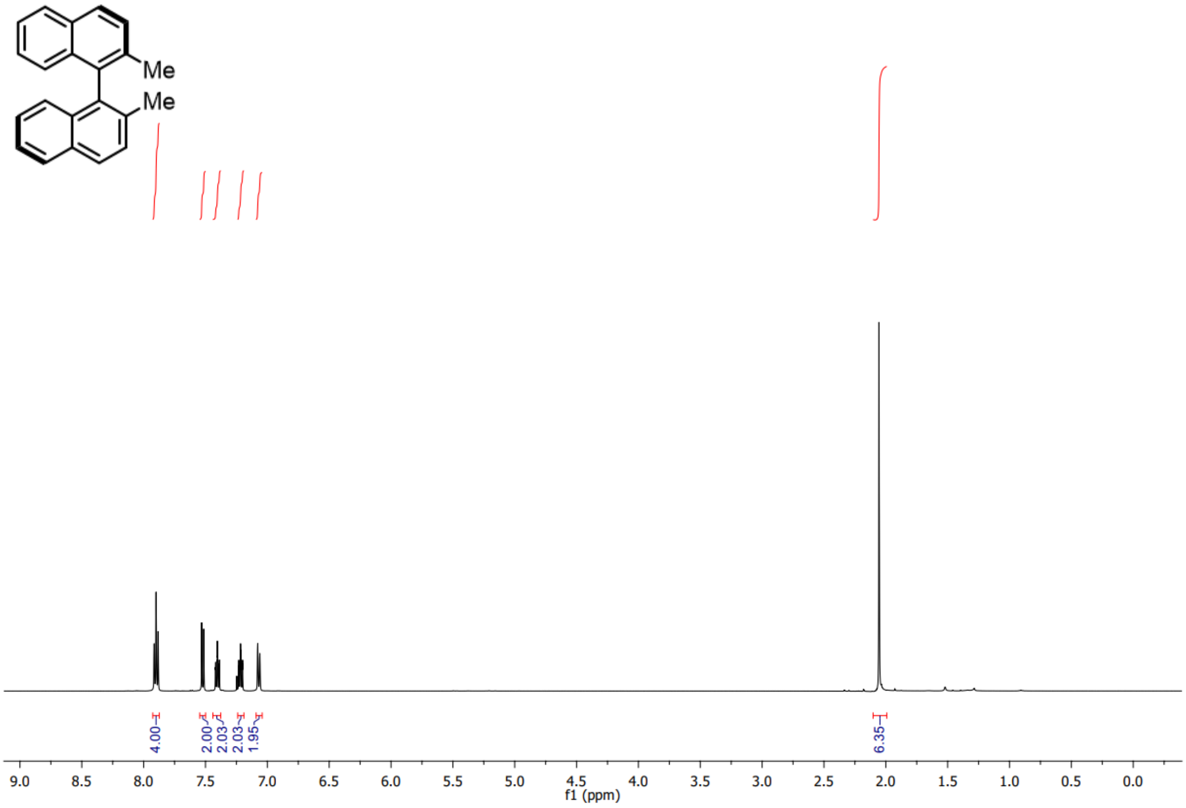
Me
Me
4.00
2.00
2.03
2.03
1.95
6.35
9.0
8.5
8.0
7.5
7.0
6.5
6.0
5.5
5.0
4.5
4.0
3.5
3.0
2.5
2.0
1.5
1.0
0.5
0.0
f1 (ppm)

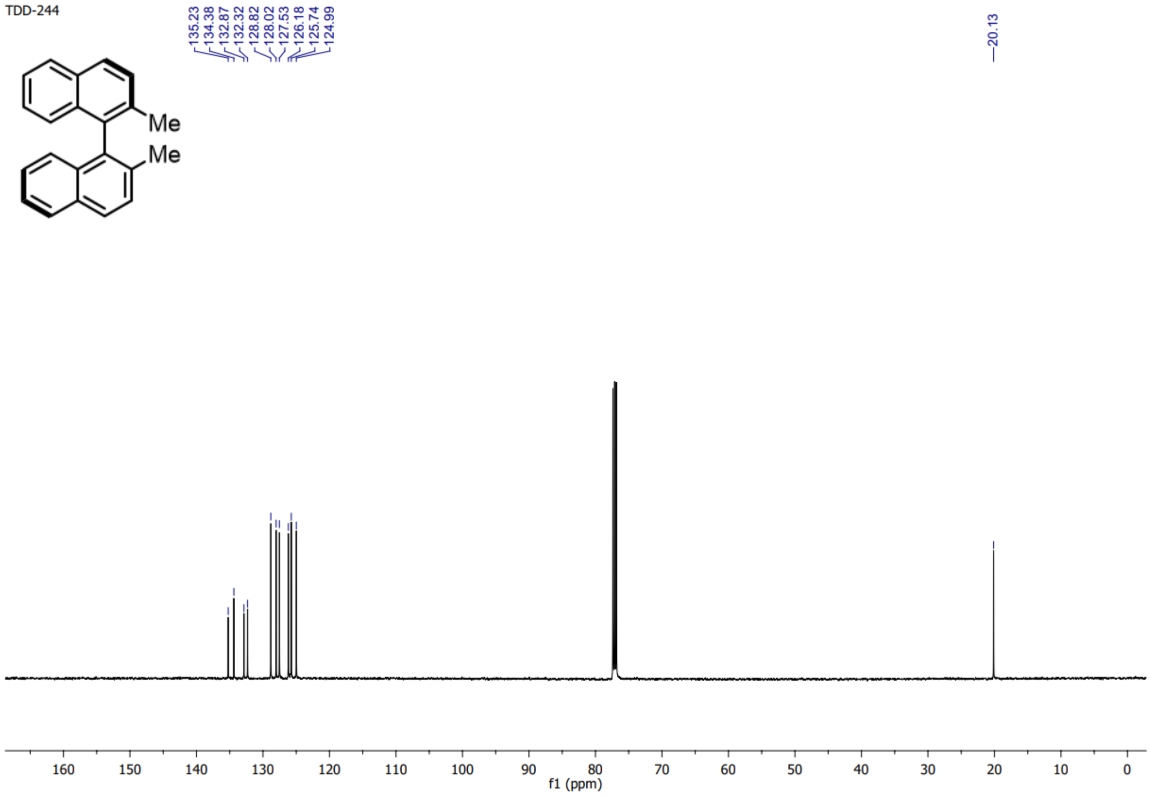
TDD-244
135.23
134.38
132.87
132.32
128.82
128.02
127.53
126.18
125.74
124.99
—20.13
Me
Me
160
150
140
130
120
110
100
90
80
70
60
50
40
30
20
10
0
f1 (ppm)

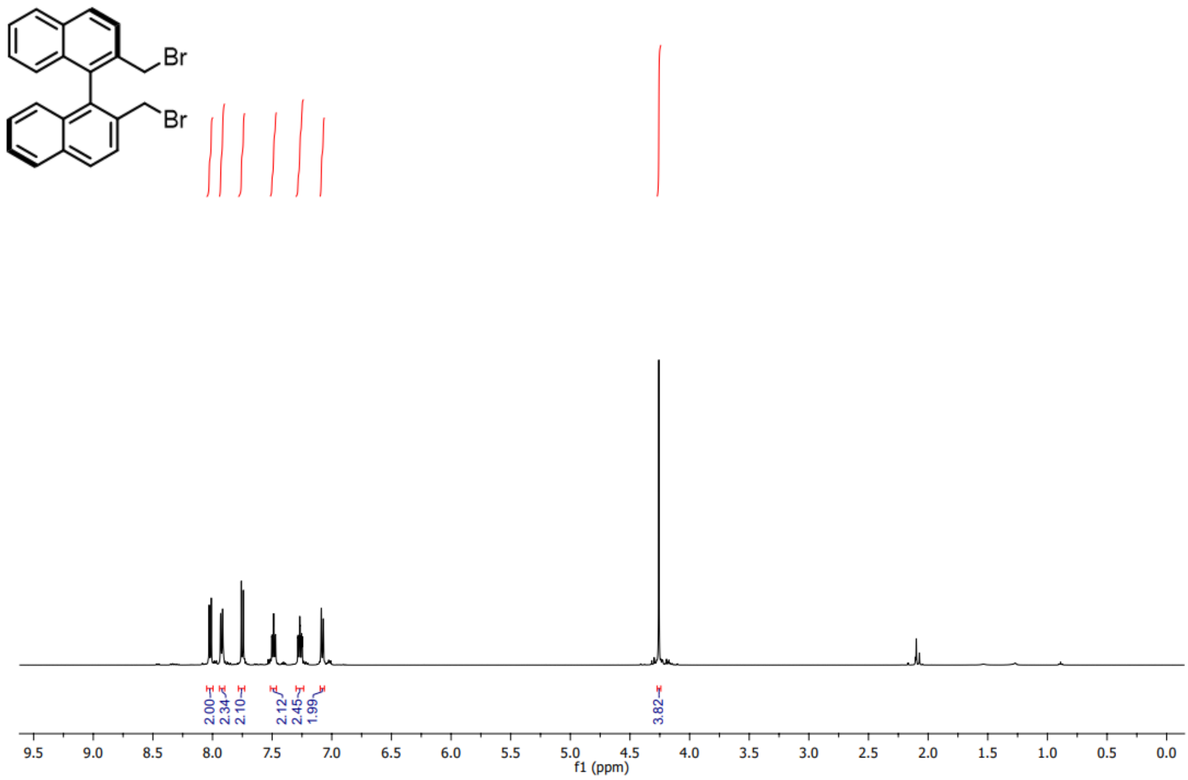
Br
Br
2.00
2.34
2.10
2.12
2.45
1.99
3.82
9.5 9.0 8.5 8.0 7.5 7.0 6.5 6.0 5.5 5.0 4.5 4.0 3.5 3.0 2.5 2.0 1.5 1.0 0.5 0.0
f1 (ppm)

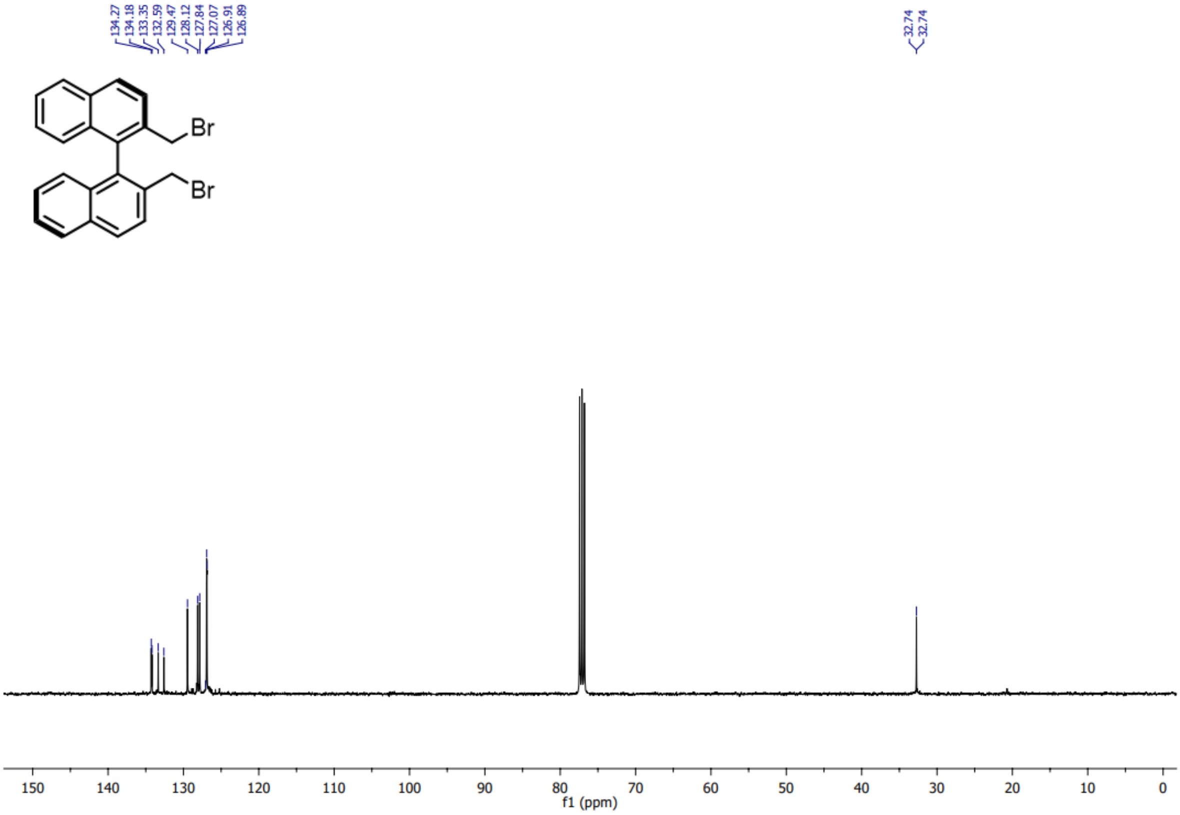
134.27
134.18
133.35
132.59
129.47
128.12
127.84
127.07
126.91
126.89
32.74
32.74
Br
Br
150 140 130 120 110 100 90 80 70 60 50 40 30 20 10 0
f1 (ppm)

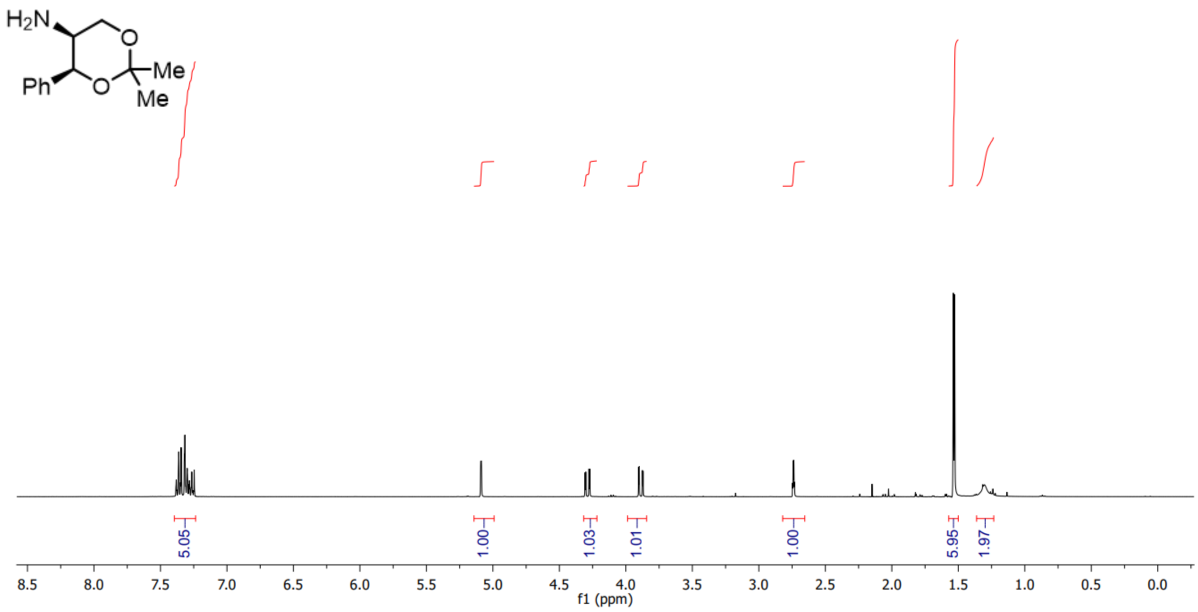
H2N
Ph
O
O
Me
Me
5.05
1.00
1.03
1.01
1.00
5.95
1.97
8.5
8.0
7.5
7.0
6.5
6.0
5.5
5.0
4.5
4.0
3.5
3.0
2.5
2.0
1.5
1.0
0.5
0.0
f1 (ppm)

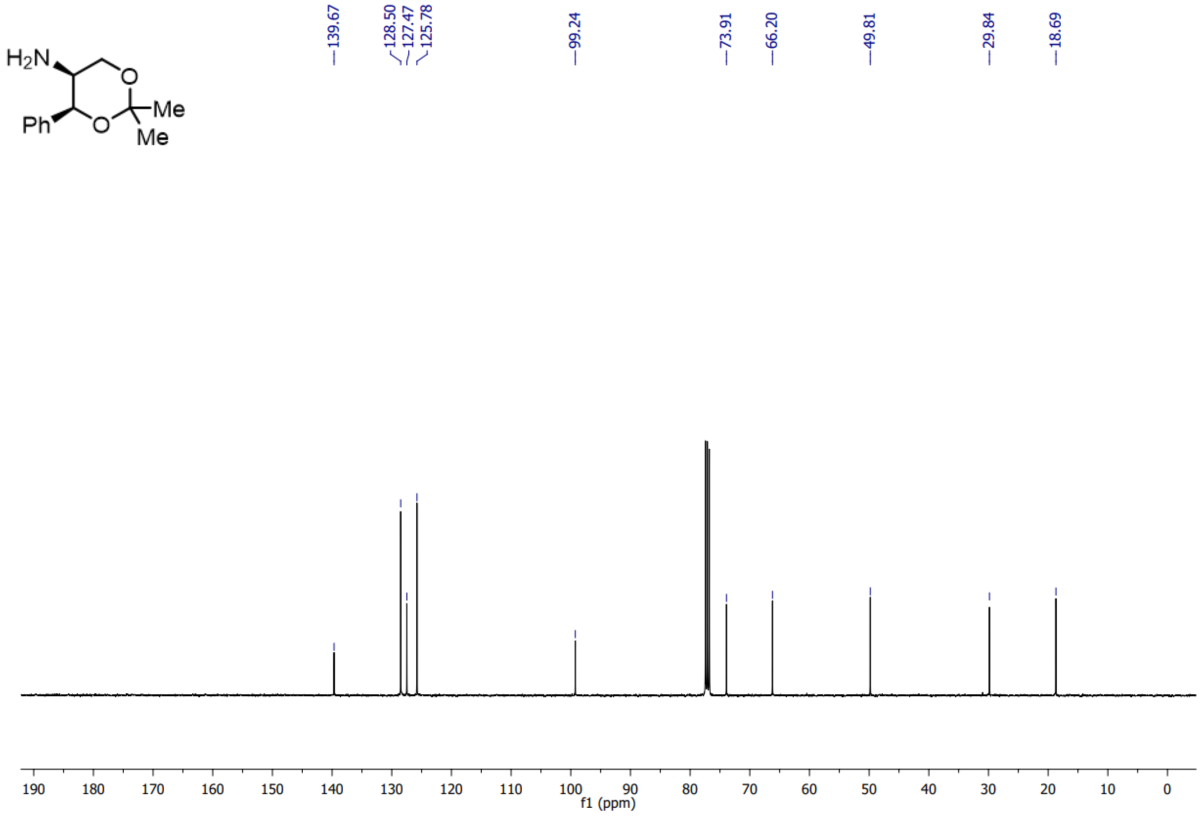
H2N
Ph
O
O
Me
Me
139.67
128.50
127.47
125.78
99.24
73.91
66.20
49.81
29.84
18.69
190
180
170
160
150
140
130
120
110
100
90
80
70
60
50
40
30
20
10
0
f1 (ppm)

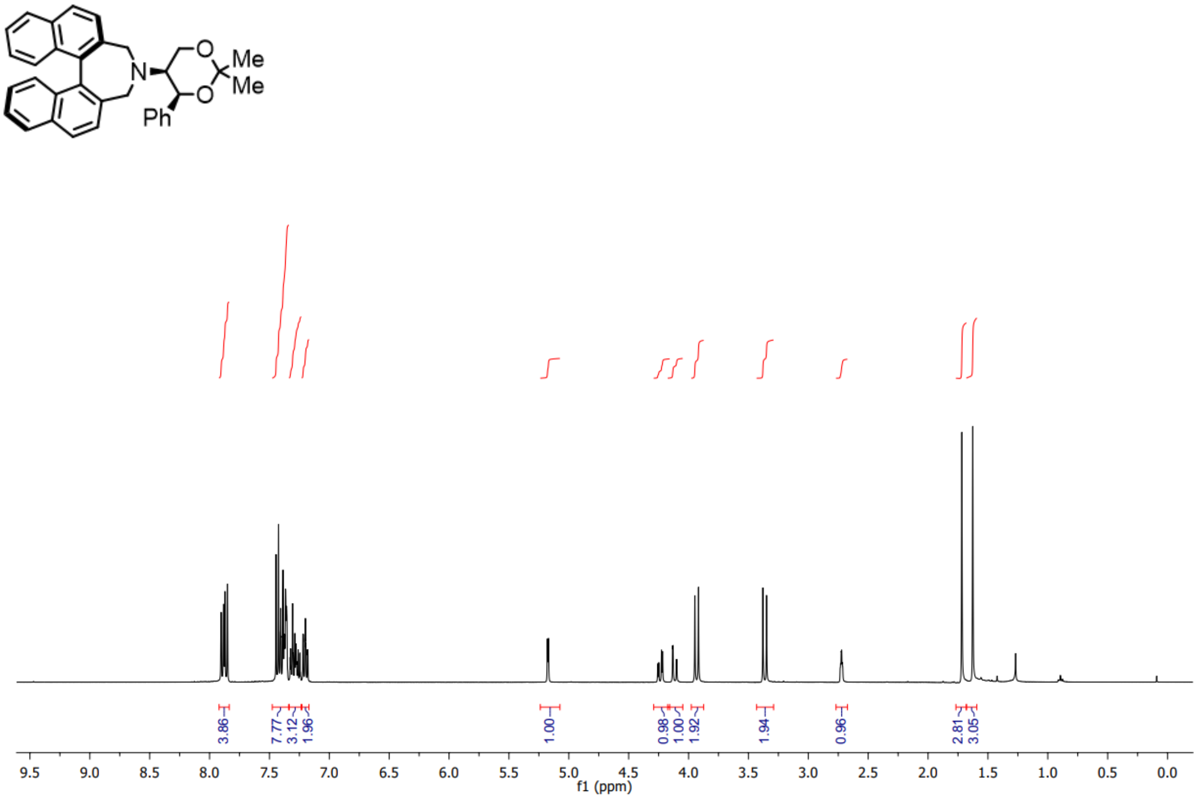
N
O
O
Me
Me
Ph
3.86
7.77
3.12
1.96
1.00
0.98
1.00
1.92
1.94
0.96
2.81
3.05
9.5 9.0 8.5 8.0 7.5 7.0 6.5 6.0 5.5 5.0 4.5 4.0 3.5 3.0 2.5 2.0 1.5 1.0 0.5 0.0
f1 (ppm)

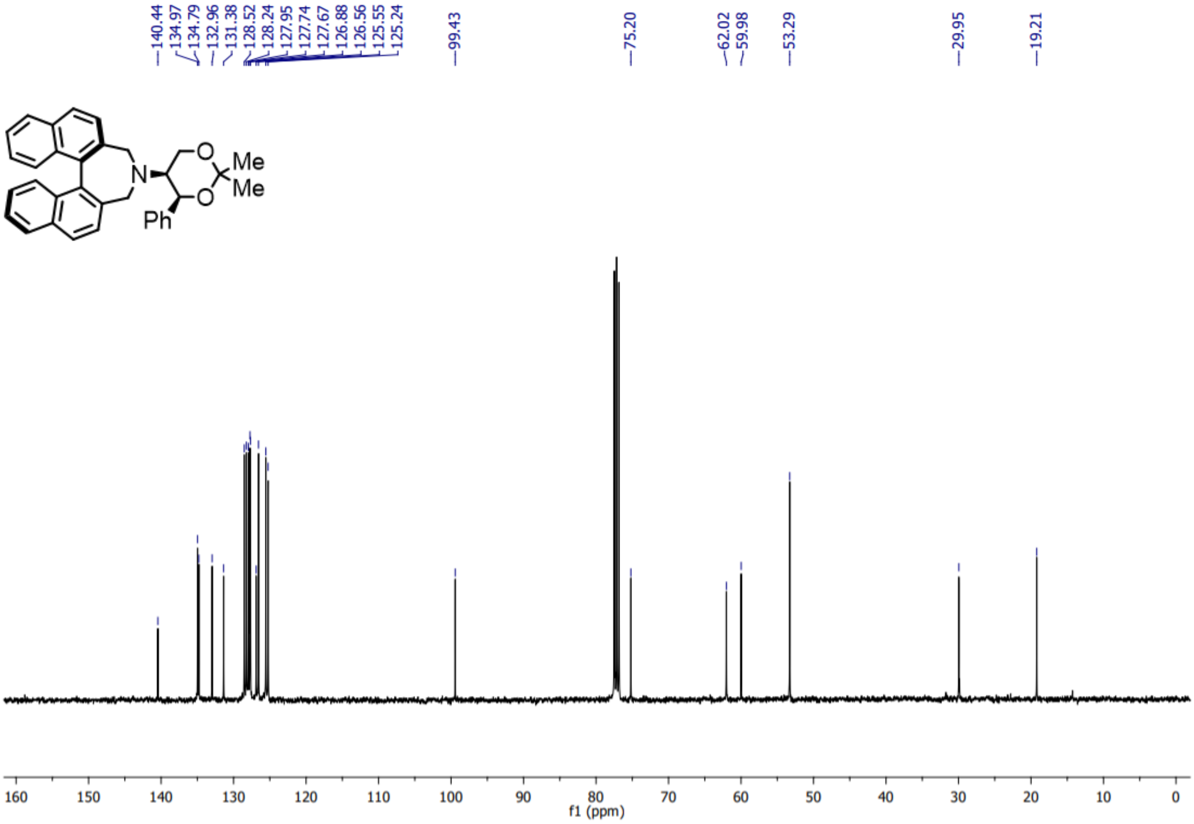
140.44
134.97
134.79
132.96
131.38
128.52
128.24
127.95
127.74
127.67
126.88
126.56
125.55
125.24
99.43
75.20
62.02
59.98
53.29
29.95
19.21
N
O
O
Me
Me
Ph
160 150 140 130 120 110 100 90 80 70 60 50 40 30 20 10 0
f1 (ppm)

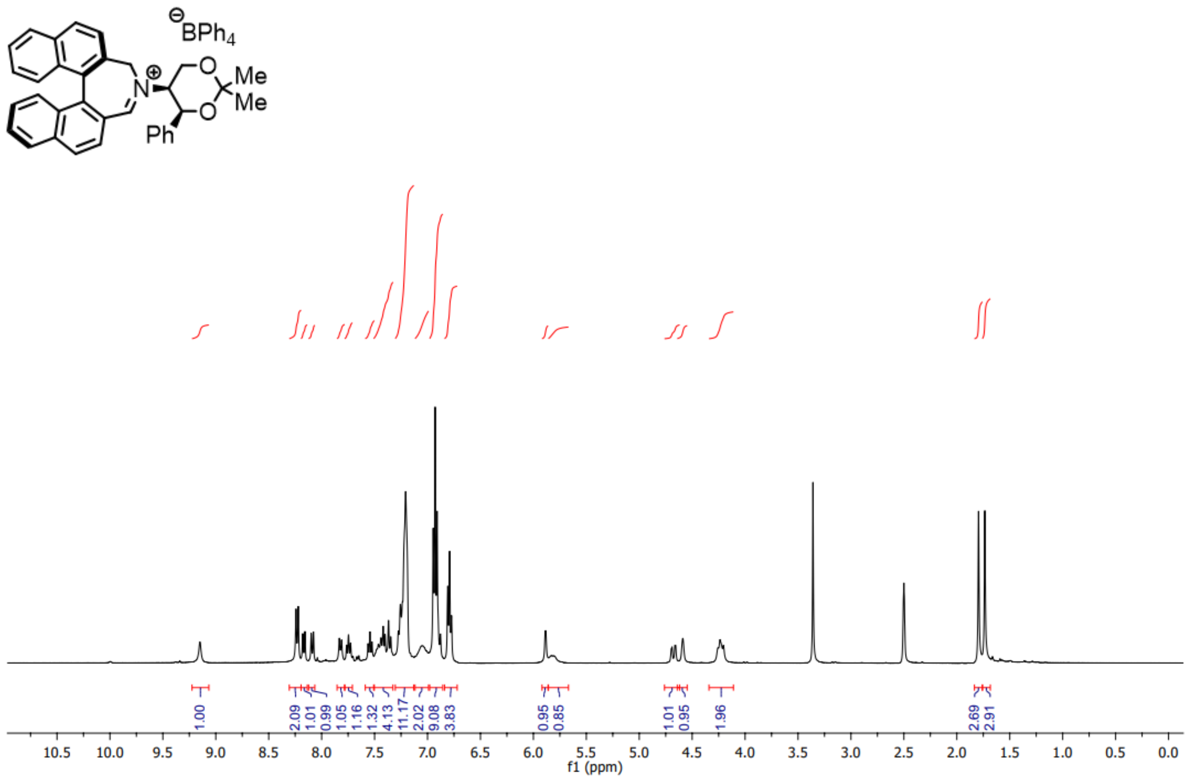
BPh4
Me
Me
Ph
1.00
2.09
1.01
0.99
1.05
1.16
1.32
4.13
11.17
2.02
9.08
3.83
0.95
0.85
1.01
0.95
1.96
2.69
2.91
f1 (ppm)

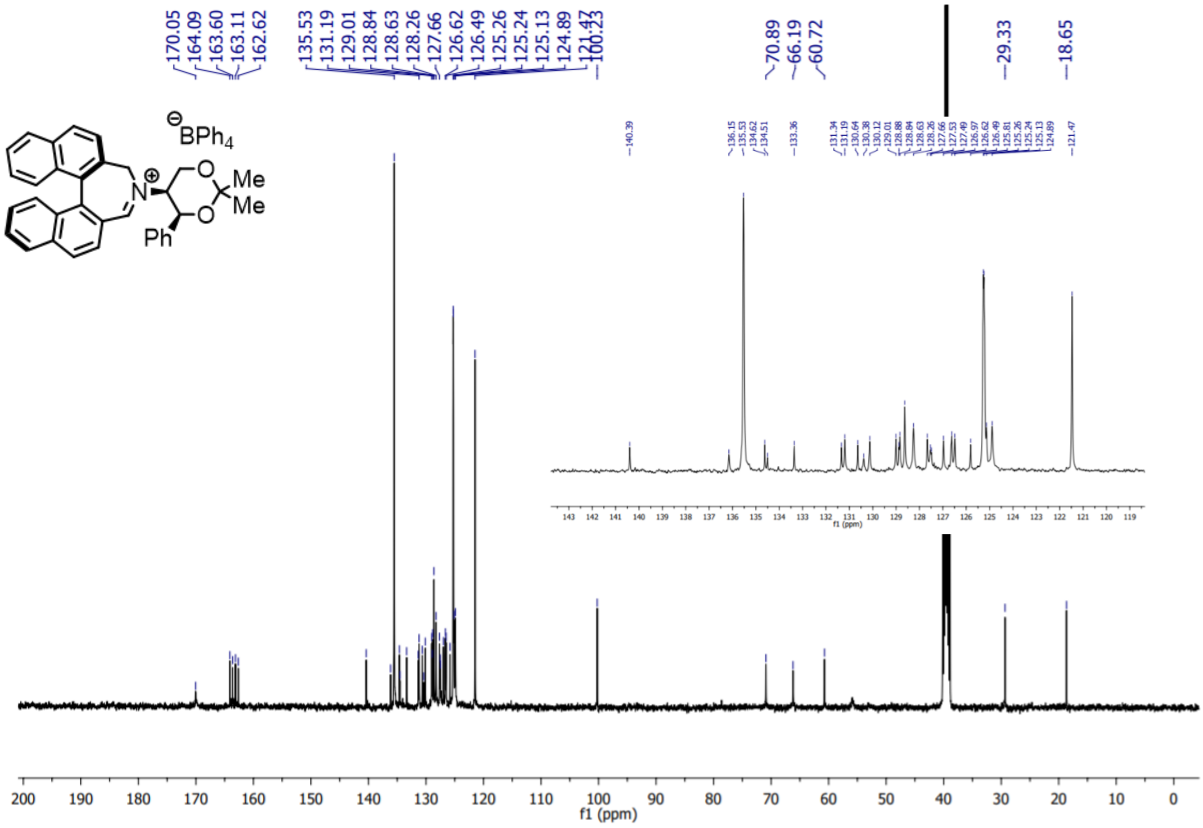
170.05
164.09
163.60
163.11
162.62
135.53
131.19
129.01
128.84
128.63
128.26
127.66
126.62
126.49
125.26
125.24
125.13
124.89
121.47
100.23
70.89
66.19
60.72
29.33
18.65
BPh4
Me
Me
Ph
f1 (ppm)

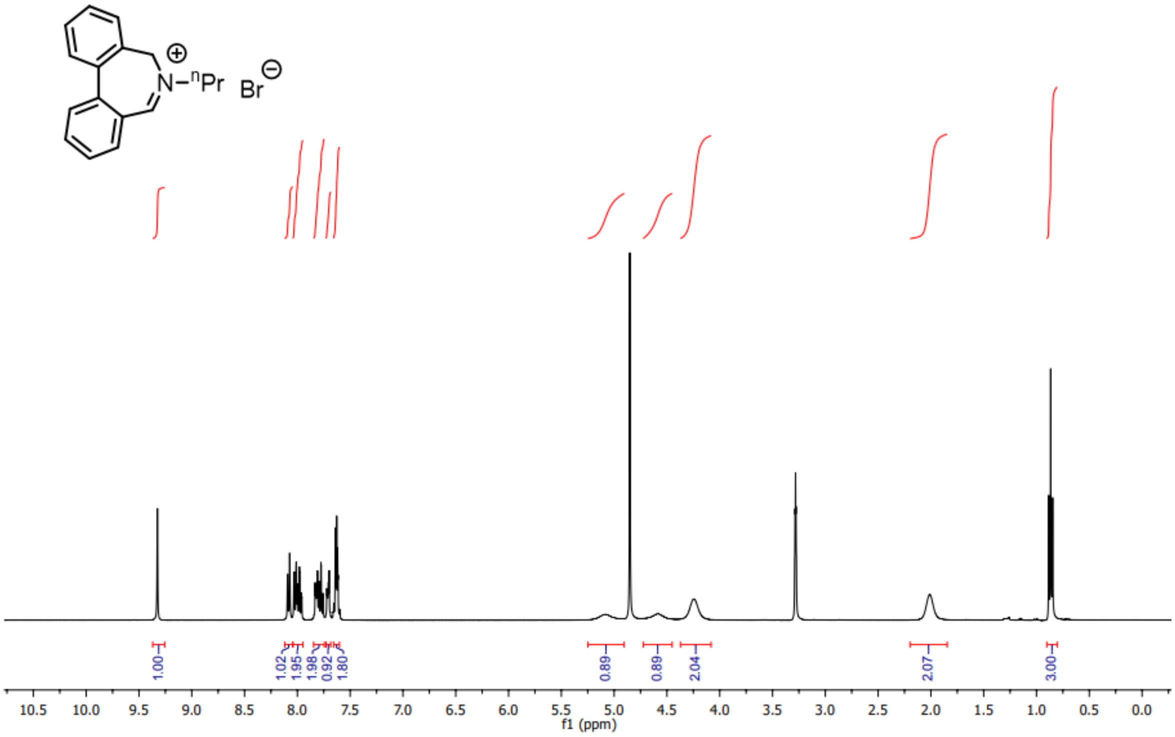
N–nPr Br
1.00
1.02
1.95
1.98
0.92
1.80
0.89
0.89
2.04
2.07
3.00
10.5 10.0 9.5 9.0 8.5 8.0 7.5 7.0 6.5 6.0 5.5 5.0 4.5 4.0 3.5 3.0 2.5 2.0 1.5 1.0 0.5 0.0
f1 (ppm)

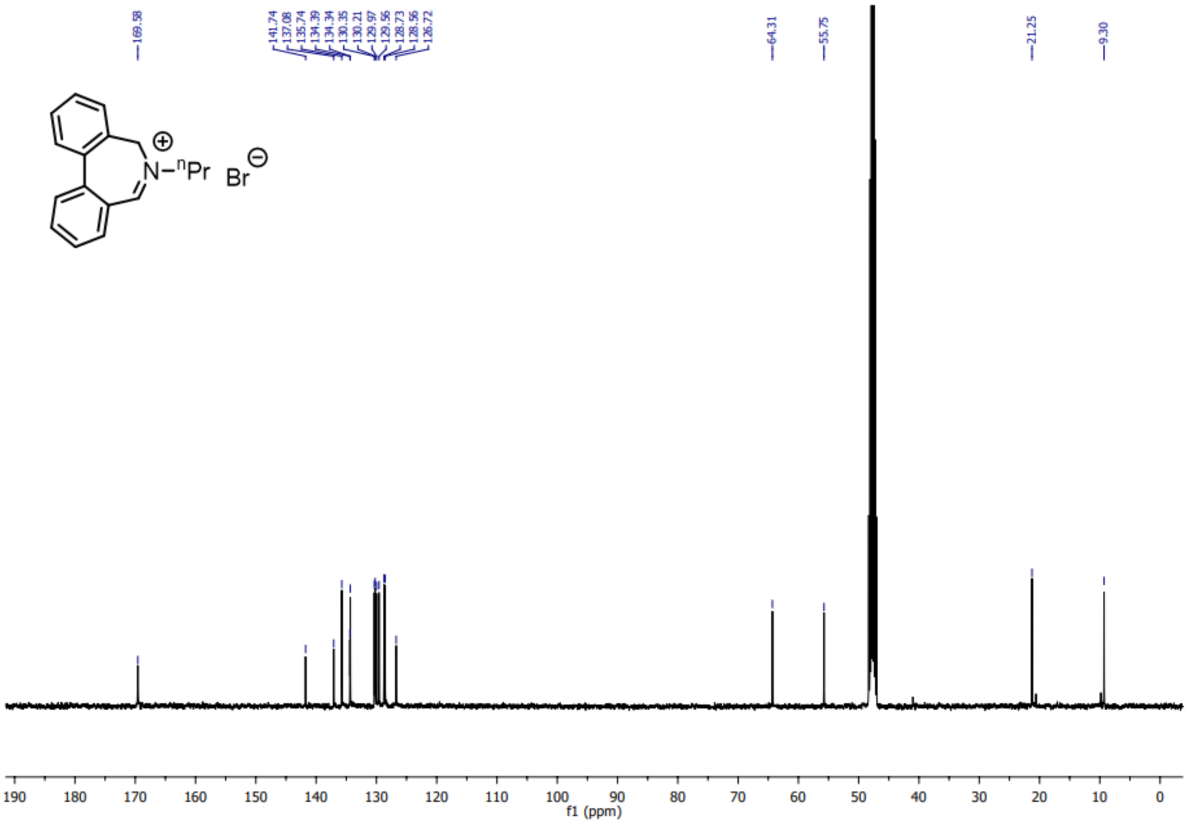
169.58
141.74
137.08
135.74
134.39
134.34
130.35
130.21
129.97
129.56
128.73
128.56
126.72
64.31
55.75
21.25
9.30
N–nPr Br
190 180 170 160 150 140 130 120 110 100 90 80 70 60 50 40 30 20 10 0
f1 (ppm)

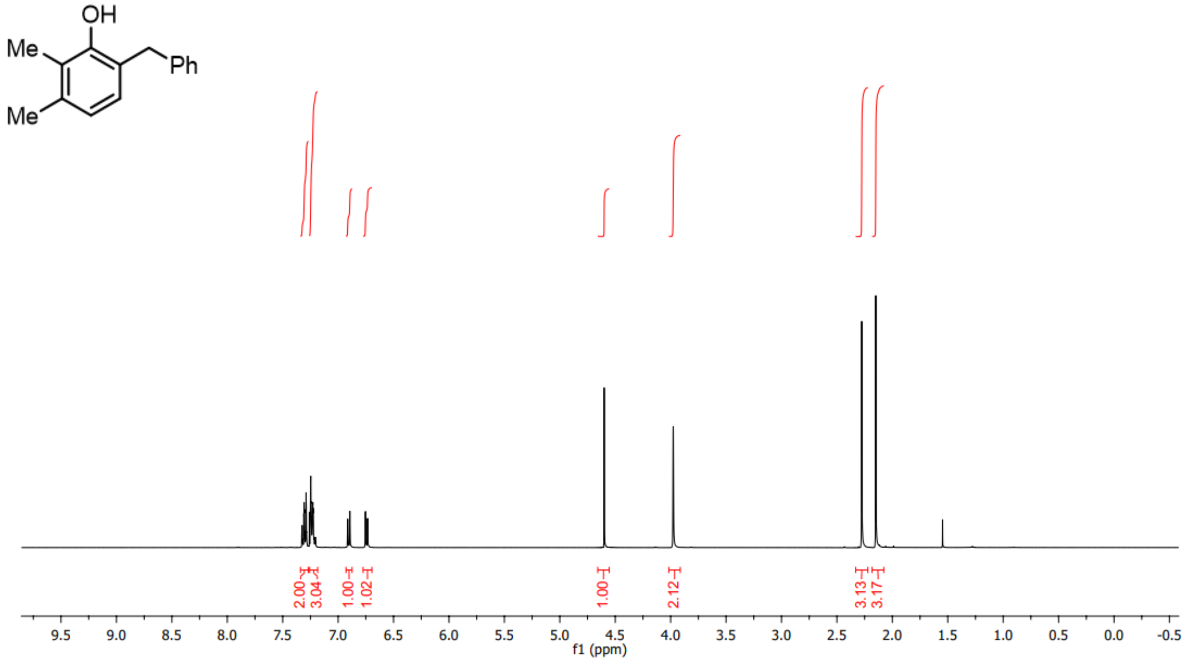
OH
Me
Me
Ph
2.00
3.04
1.00
1.02
1.00
2.12
3.13
3.17
9.5
9.0
8.5
8.0
7.5
7.0
6.5
6.0
5.5
5.0
4.5
4.0
3.5
3.0
2.5
2.0
1.5
1.0
0.5
0.0
-0.5
f1 (ppm)

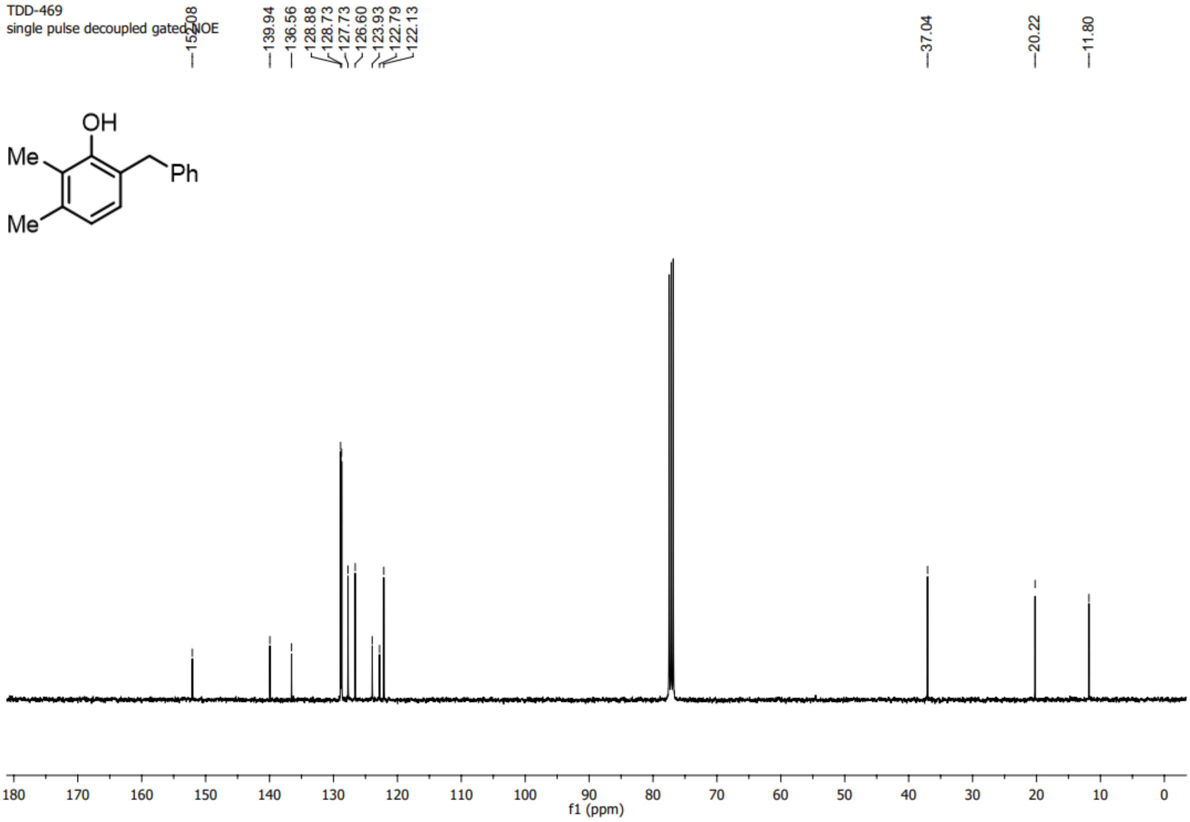
TDD-469
single pulse decoupled gated NOE
152.08
139.94
136.56
128.88
128.73
127.73
126.60
123.93
122.79
122.13
37.04
20.22
11.80
OH
Me
Me
Ph
180
170
160
150
140
130
120
110
100
90
80
70
60
50
40
30
20
10
0
f1 (ppm)

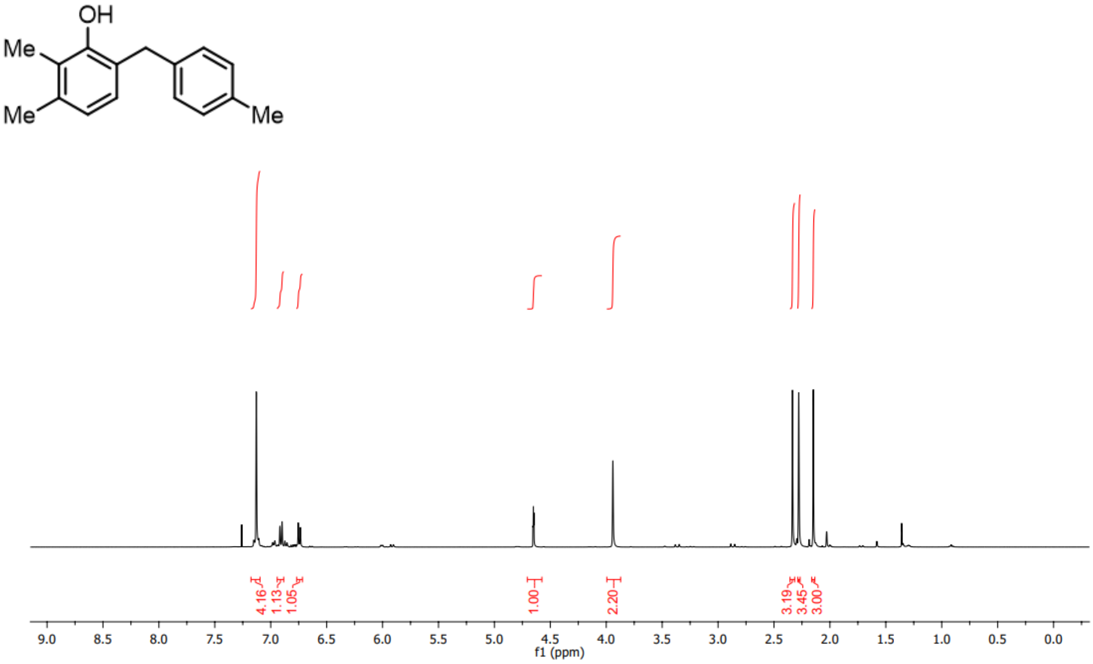
OH
Me
Me
Me
4.16
1.13
1.05
1.00
2.20
3.19
3.45
3.00
9.0 8.5 8.0 7.5 7.0 6.5 6.0 5.5 5.0 4.5 4.0 3.5 3.0 2.5 2.0 1.5 1.0 0.5 0.0
f1 (ppm)

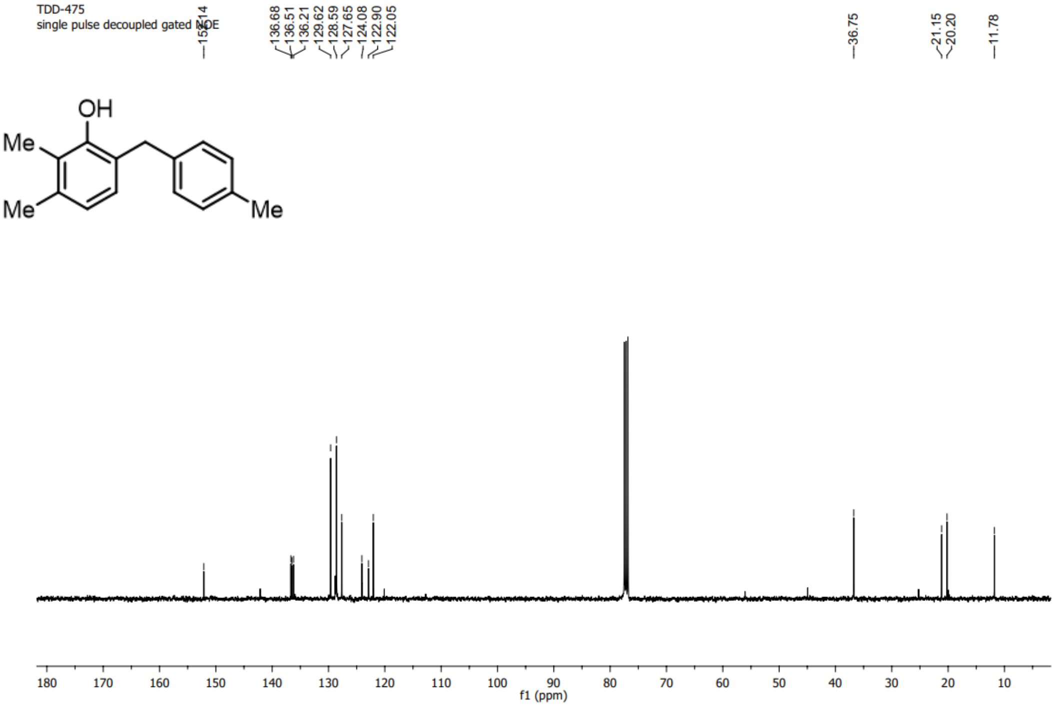
TDD-475
single pulse decoupled gated NOE
136.68
136.51
136.21
129.62
128.59
127.65
124.08
122.90
122.05
36.75
21.15
20.20
11.78
OH
Me
Me
Me
180 170 160 150 140 130 120 110 100 90 80 70 60 50 40 30 20 10
f1 (ppm)

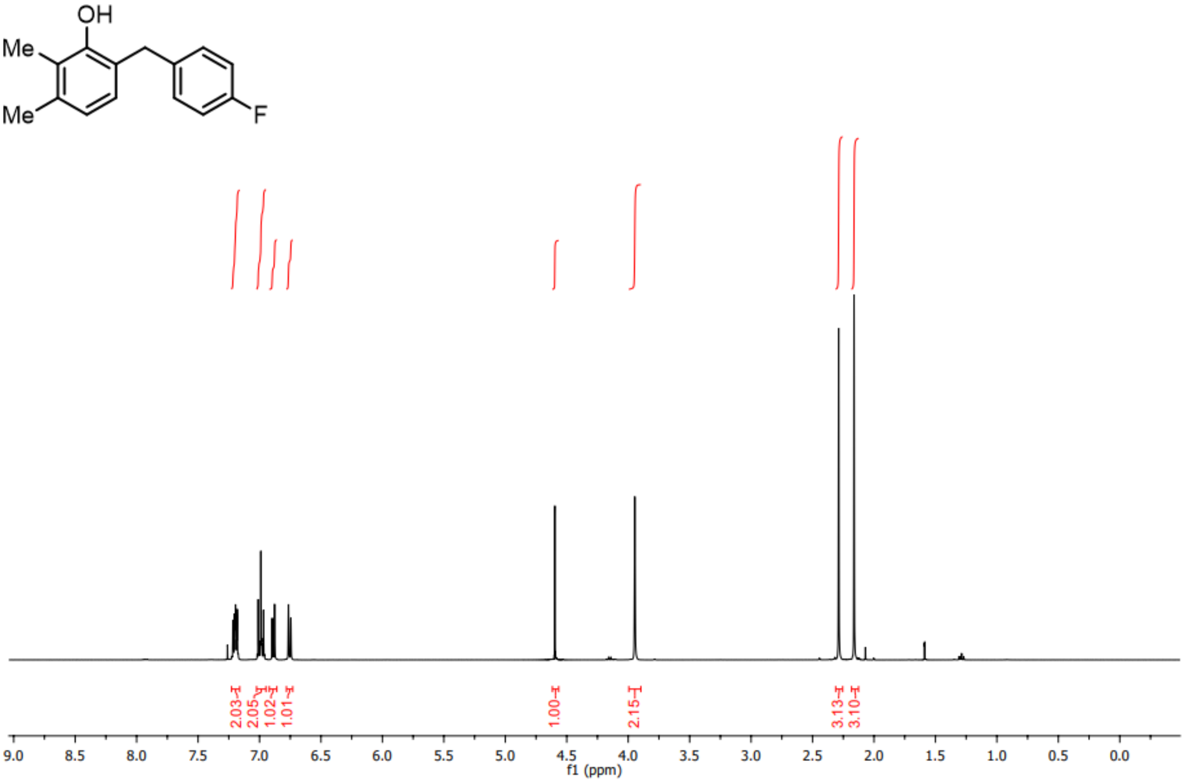
OH
Me
Me
F
2.03
2.05
1.02
1.01
1.00
2.15
3.13
3.10
9.0 8.5 8.0 7.5 7.0 6.5 6.0 5.5 5.0 4.5 4.0 3.5 3.0 2.5 2.0 1.5 1.0 0.5 0.0
f1 (ppm)

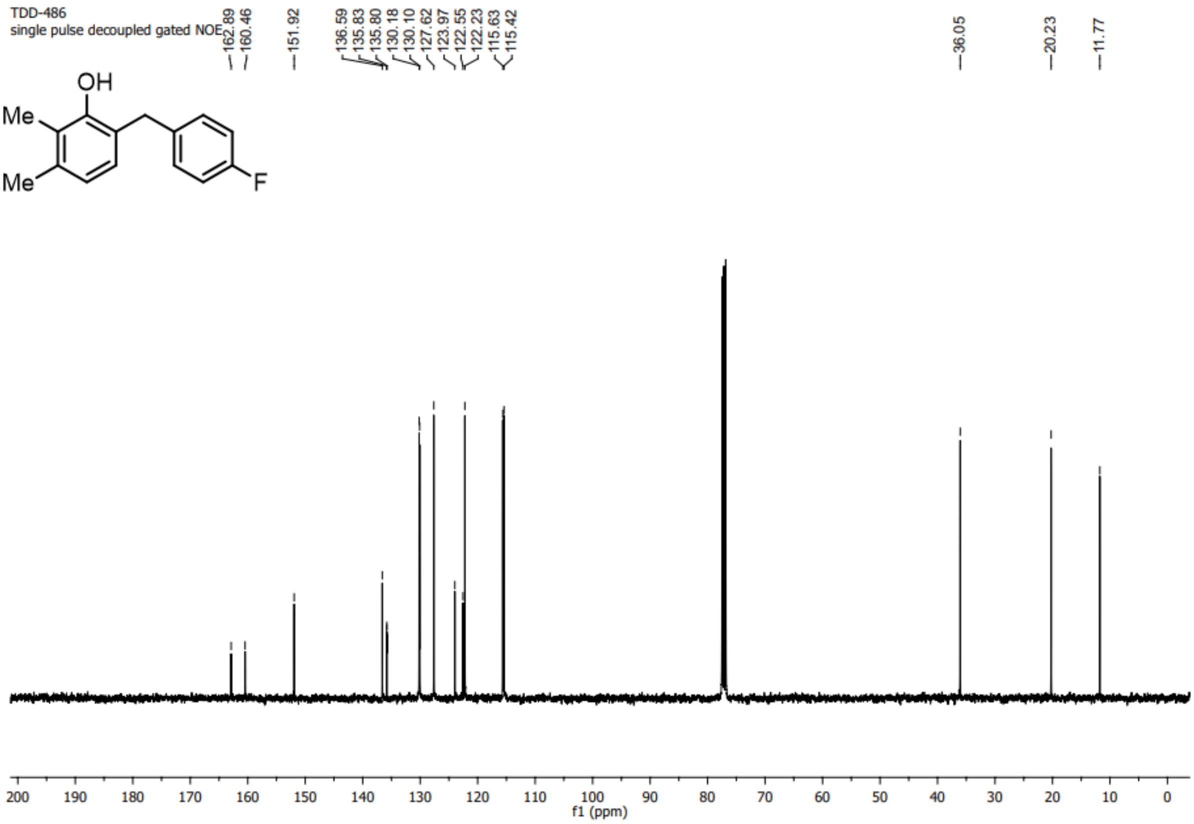
TDD-486
single pulse decoupled gated NOE
162.89
160.46
151.92
136.59
135.83
135.80
130.18
130.10
127.62
123.97
122.55
122.23
115.63
115.42
36.05
20.23
11.77
OH
Me
Me
F
200 190 180 170 160 150 140 130 120 110 100 90 80 70 60 50 40 30 20 10 0
f1 (ppm)

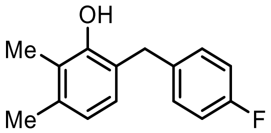

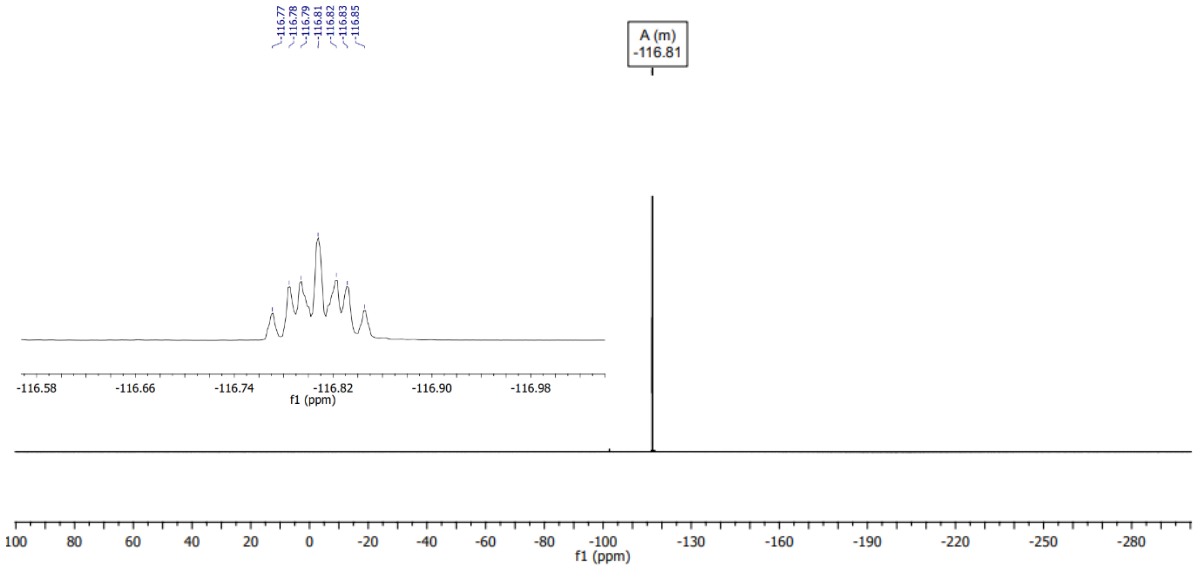
-116.77
-116.78
-116.79
-116.81
-116.82
-116.83
-116.85
A (m)
-116.81
-116.58
-116.66
-116.74
-116.82
-116.90
-116.98
f1 (ppm)
100
80
60
40
20
0
-20
-40
-60
-80
-100
-130
-160
-190
-220
-250
-280
f1 (ppm)

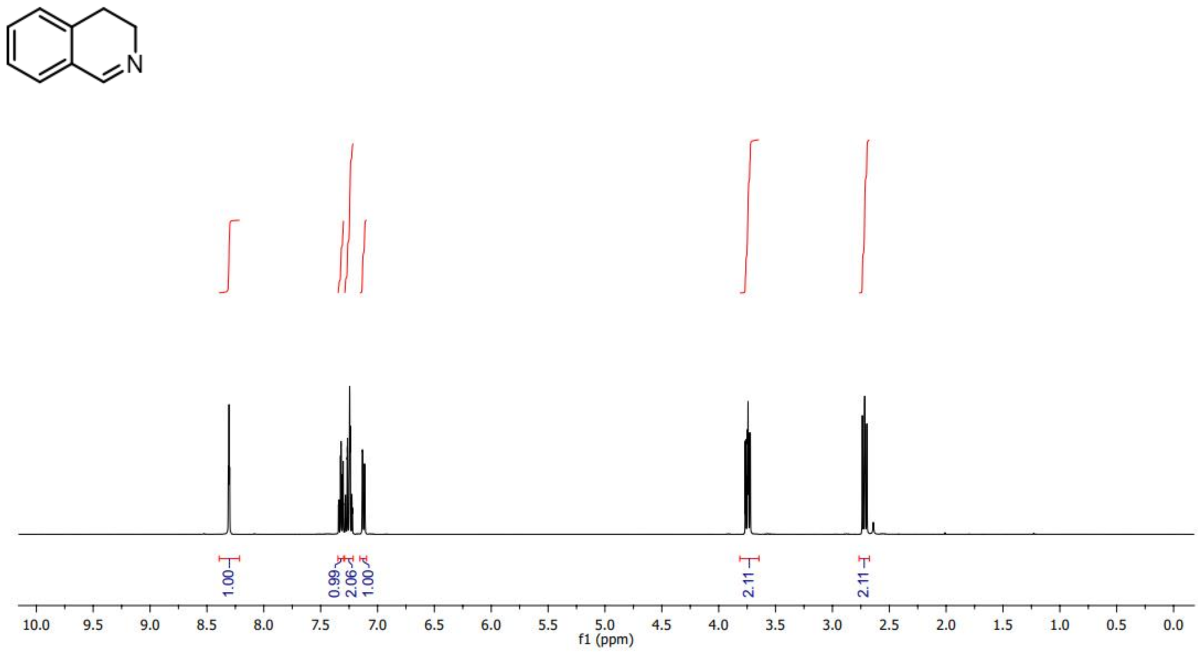
N
1.00
0.99
2.06
1.00
2.11
2.11
10.0 9.5 9.0 8.5 8.0 7.5 7.0 6.5 6.0 5.5 5.0 4.5 4.0 3.5 3.0 2.5 2.0 1.5 1.0 0.5 0.0
f1 (ppm)

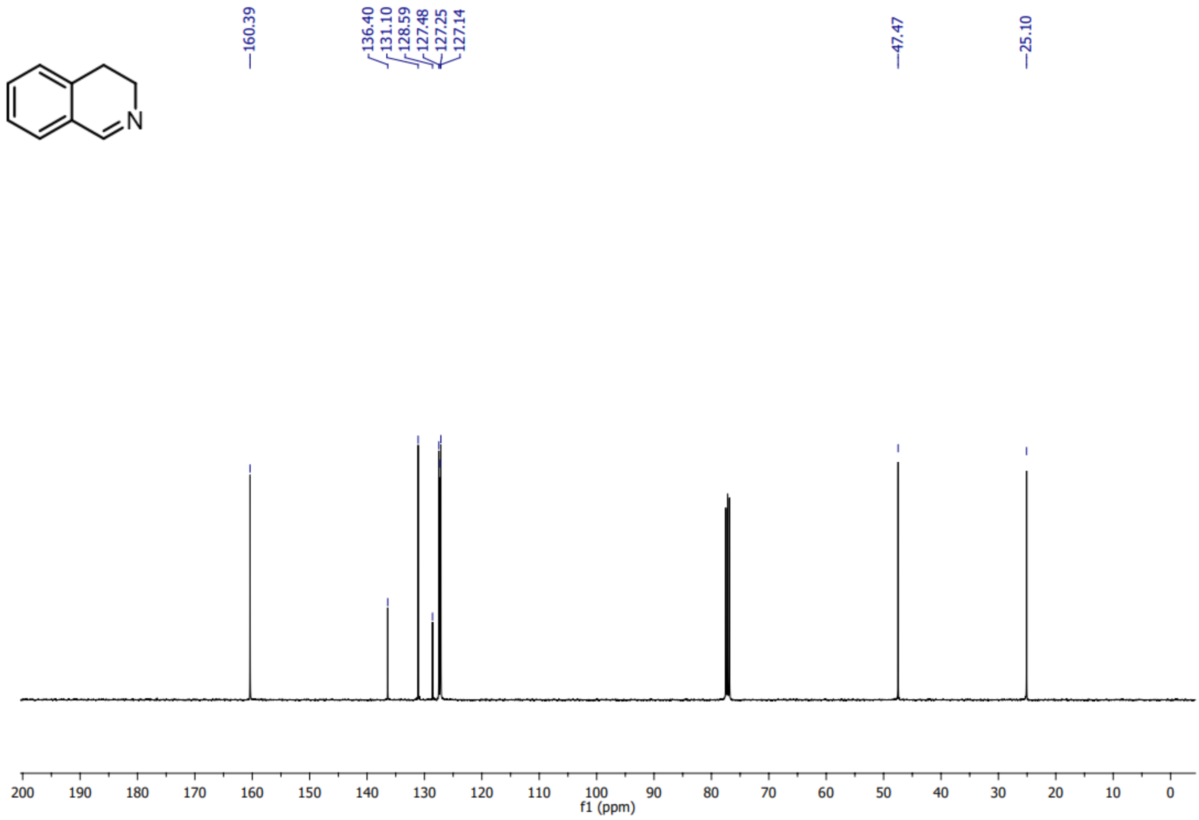
N
160.39
136.40
131.10
128.59
127.48
127.25
127.14
47.47
25.10
200 190 180 170 160 150 140 130 120 110 100 90 80 70 60 50 40 30 20 10 0
f1 (ppm)

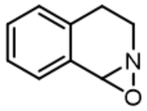
N
O

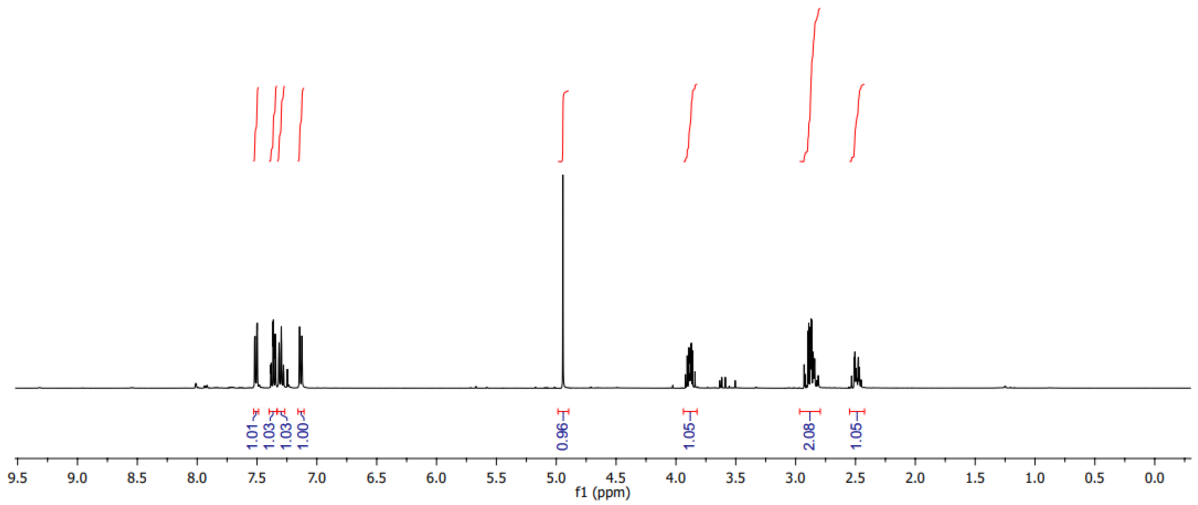
1.01
1.03
1.03
1.00
0.96
1.05
2.08
1.05
9.5 9.0 8.5 8.0 7.5 7.0 6.5 6.0 5.5 5.0 4.5 4.0 3.5 3.0 2.5 2.0 1.5 1.0 0.5 0.0
f1 (ppm)

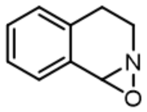
N
O

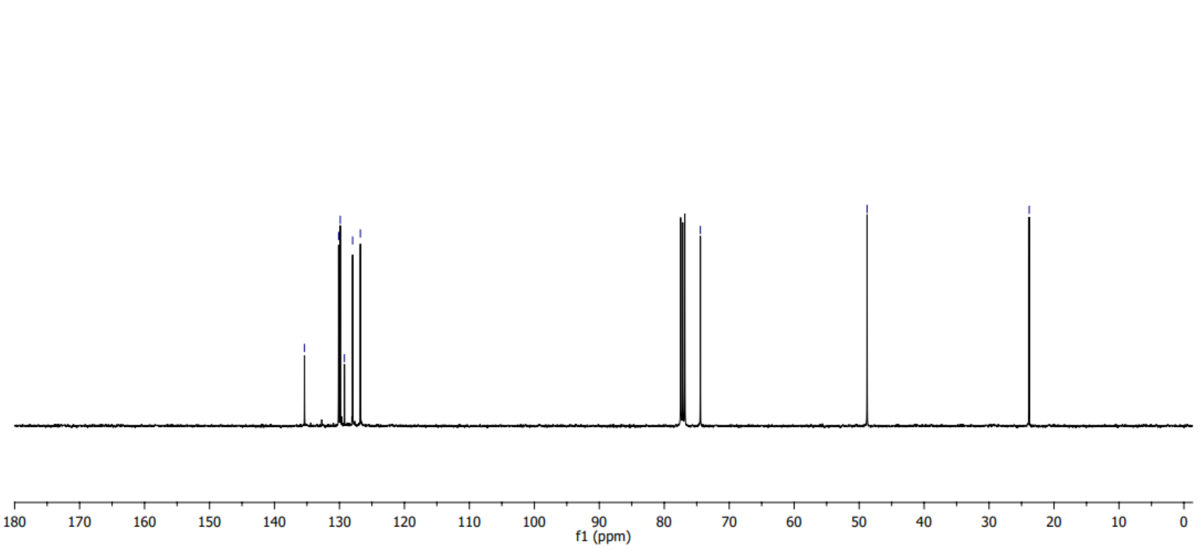
135.38
130.13
129.88
129.24
127.97
126.78
74.44
48.77
23.82
180 170 160 150 140 130 120 110 100 90 80 70 60 50 40 30 20 10 0
f1 (ppm)

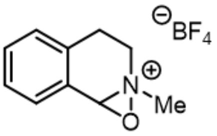

(~5.5:1 oxaziridinium : iminium)

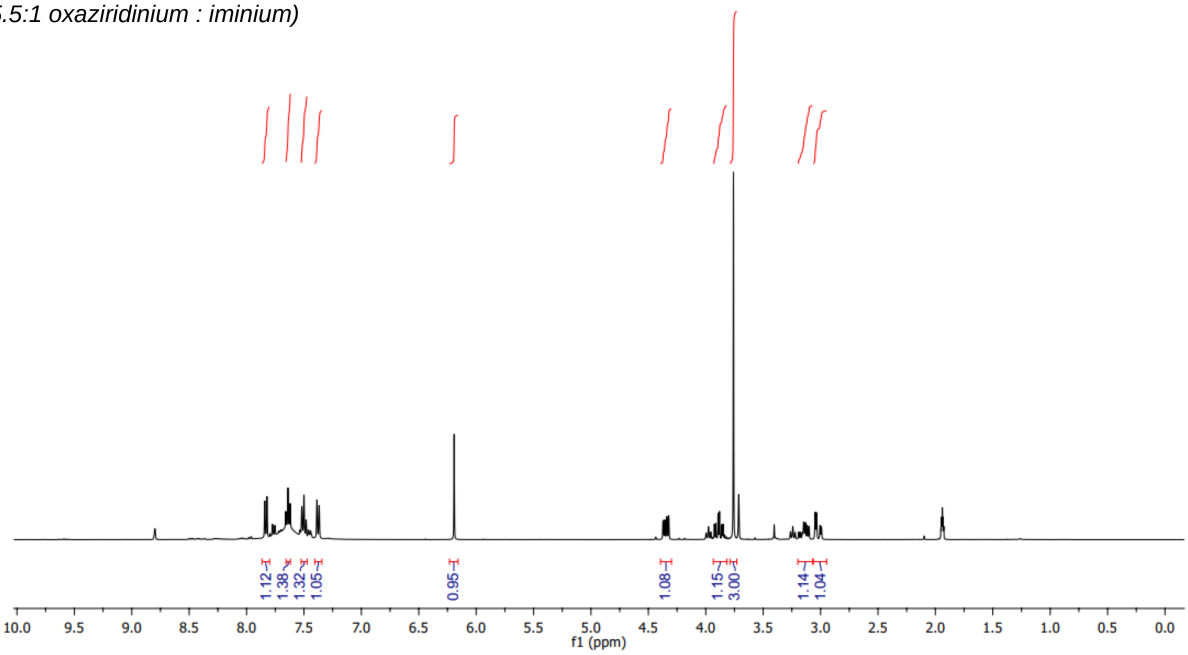

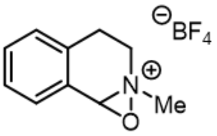

(~5.5:1 oxaziridinium : iminium)

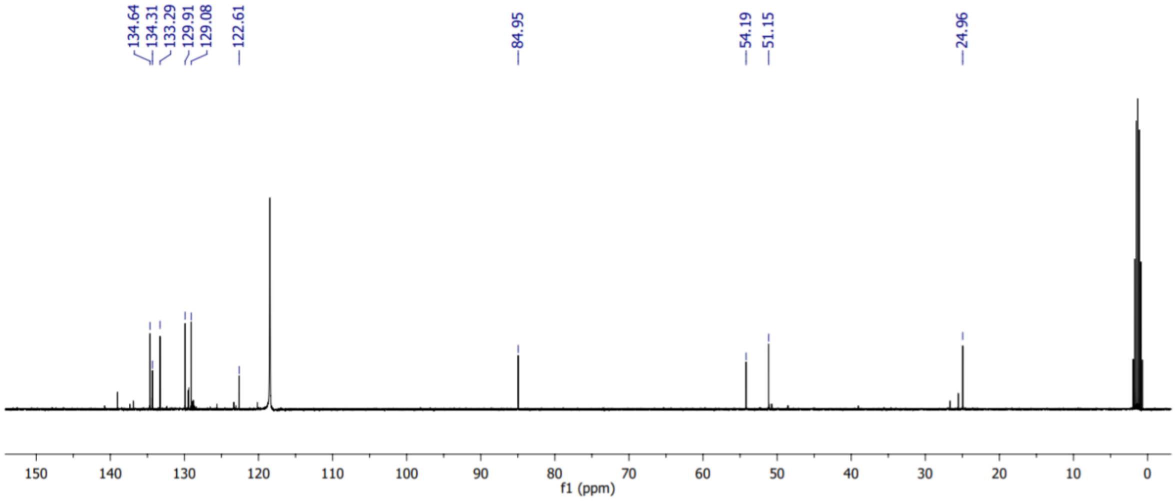

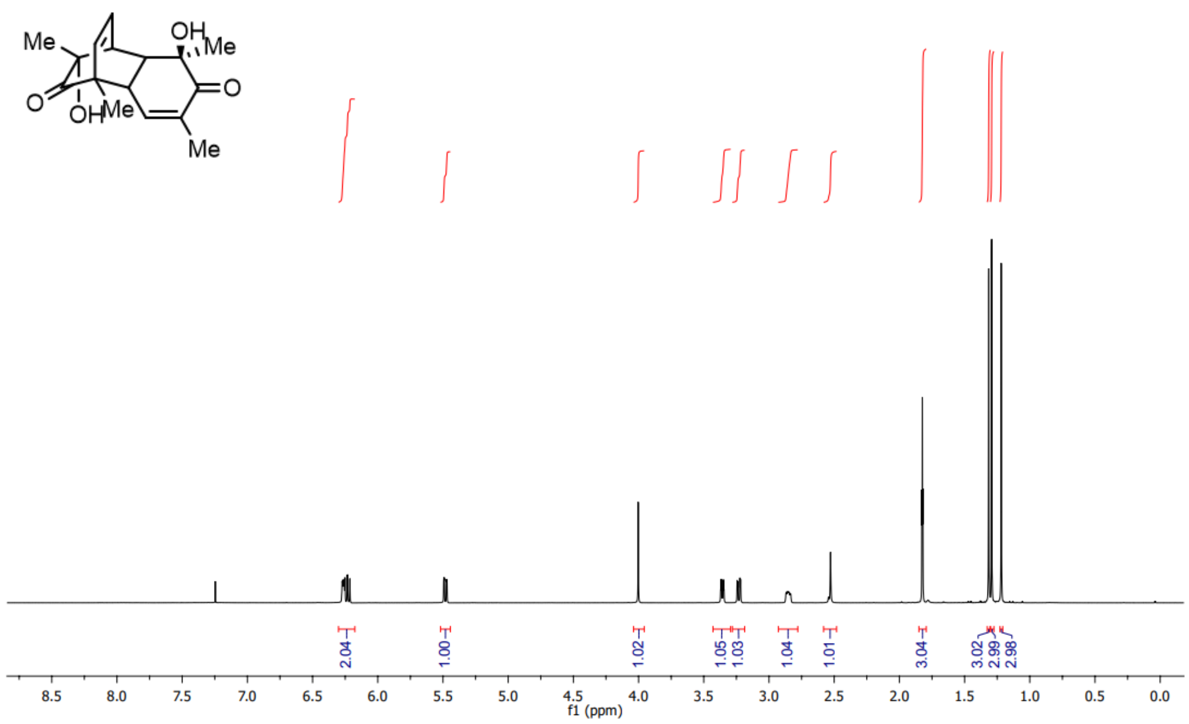
Me
OH
Me
O
O
OH
Me
Me
2.04
1.00
1.02
1.05
1.03
1.04
1.01
3.04
3.02
2.99
2.98
8.5 8.0 7.5 7.0 6.5 6.0 5.5 5.0 4.5 4.0 3.5 3.0 2.5 2.0 1.5 1.0 0.5 0.0
f1 (ppm)

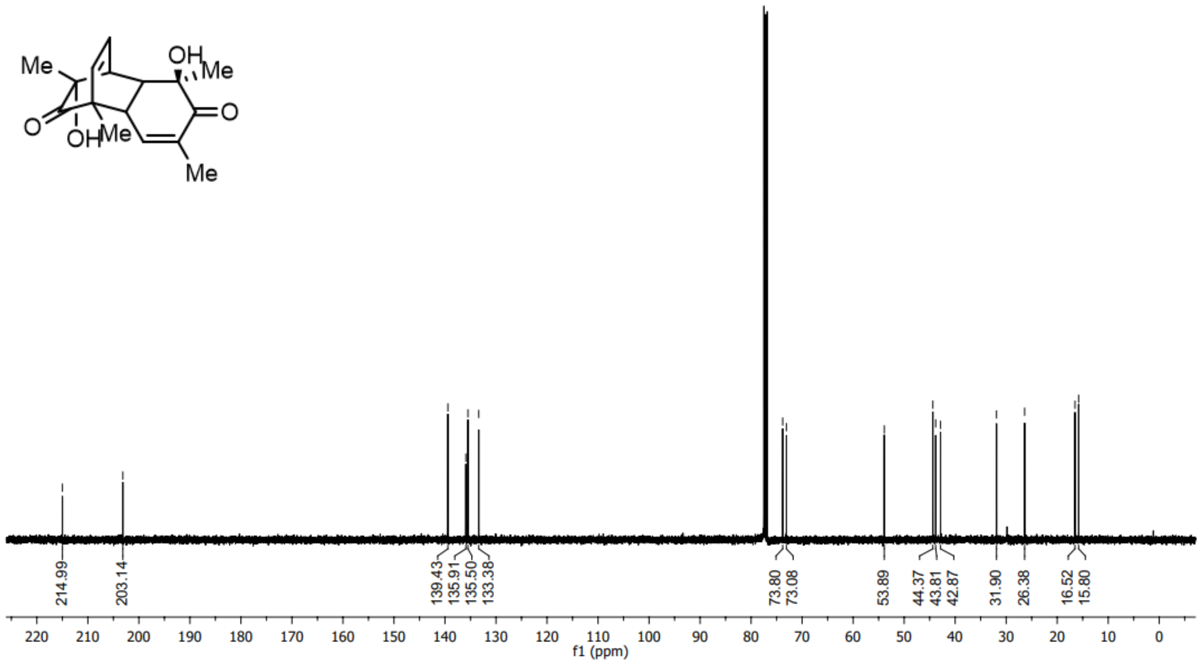
Me
OH
Me
O
O
OH
Me
Me
214.99
203.14
139.43
135.91
135.50
133.38
73.80
73.08
53.89
44.37
43.81
42.87
31.90
26.38
16.52
15.80
220 210 200 190 180 170 160 150 140 130 120 110 100 90 80 70 60 50 40 30 20 10 0
f1 (ppm)

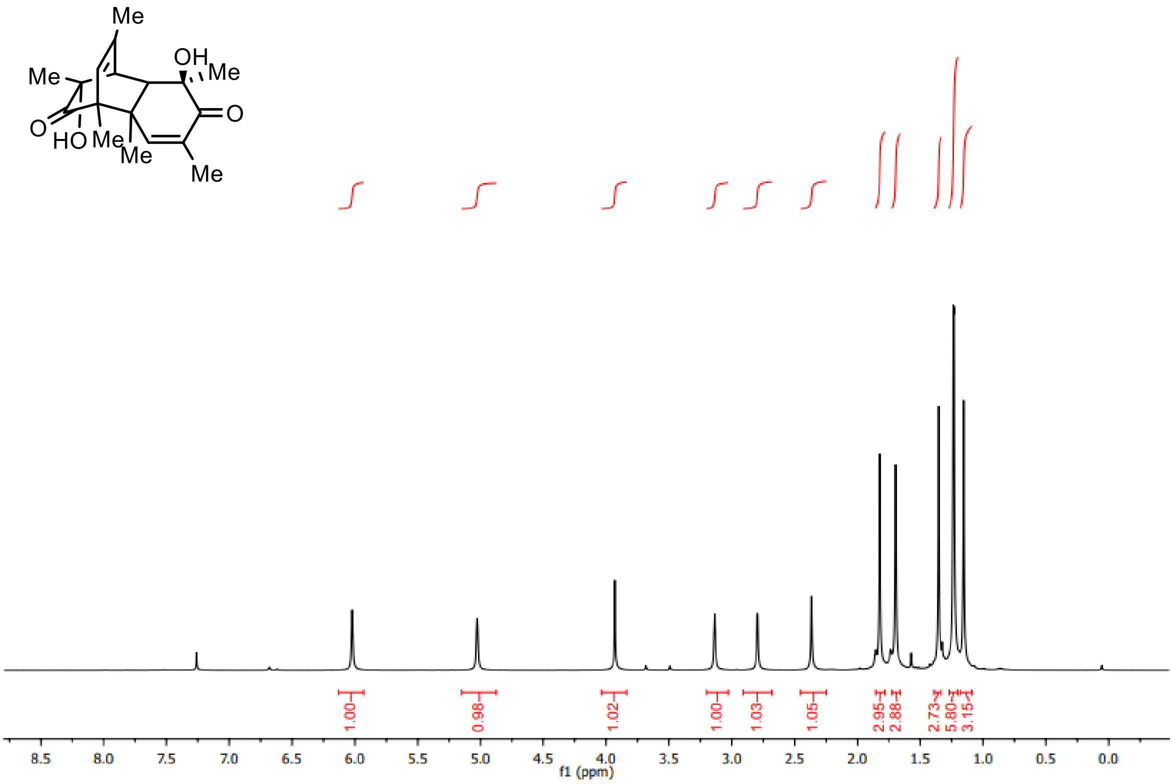
Me
OH
Me
Me
O
O
HO Me
Me
Me
1.00
0.98
1.02
1.00
1.03
1.05
2.95
2.88
2.73
5.80
3.15
f1 (ppm)

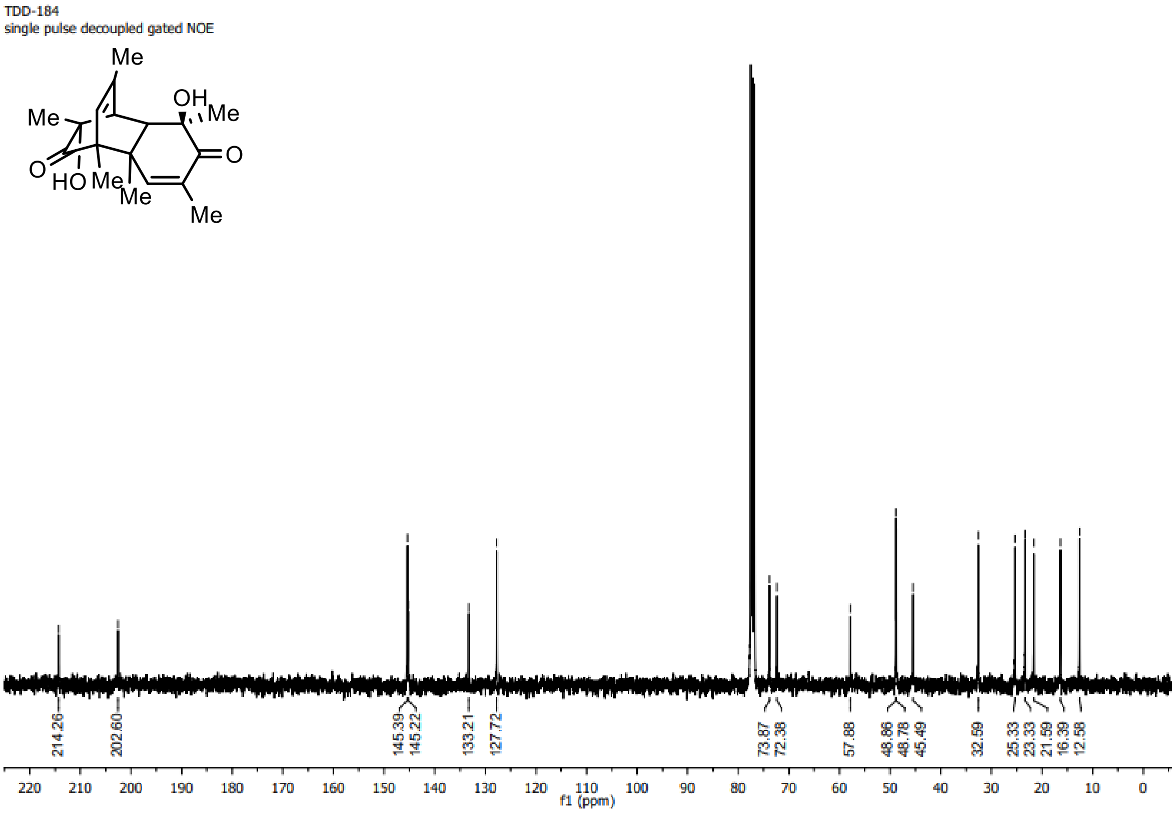
TDD-184
single pulse decoupled gated NOE
Me
OH
Me
Me
O
O
HO Me
Me
Me
214.26
202.60
145.39
145.22
133.21
127.72
73.87
72.38
57.88
48.86
48.78
45.49
32.59
25.33
23.33
21.59
16.39
12.58
f1 (ppm)

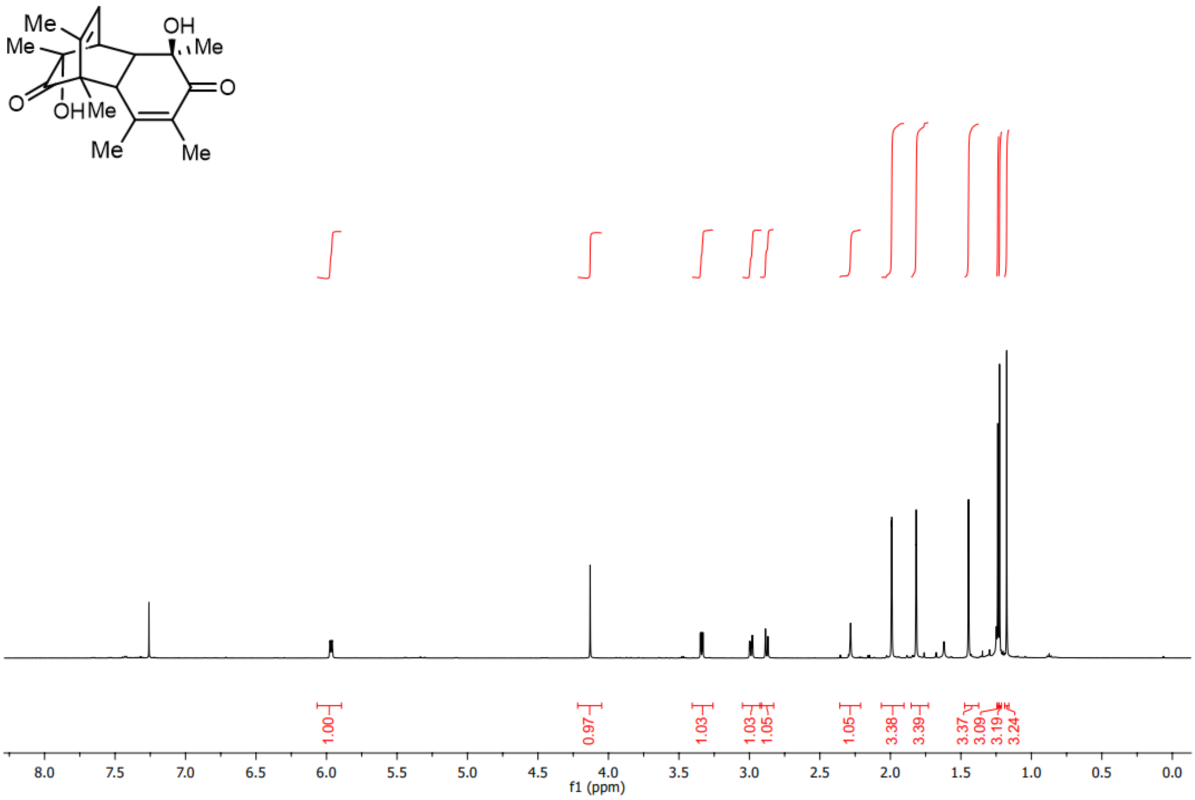
Me
Me
OH
Me
O
OH
Me
O
Me
Me
1.00
0.97
1.03
1.03
1.05
1.05
3.38
3.39
3.37
3.09
3.19
3.24
8.0
7.5
7.0
6.5
6.0
5.5
5.0
4.5
4.0
3.5
3.0
2.5
2.0
1.5
1.0
0.5
0.0
f1 (ppm)

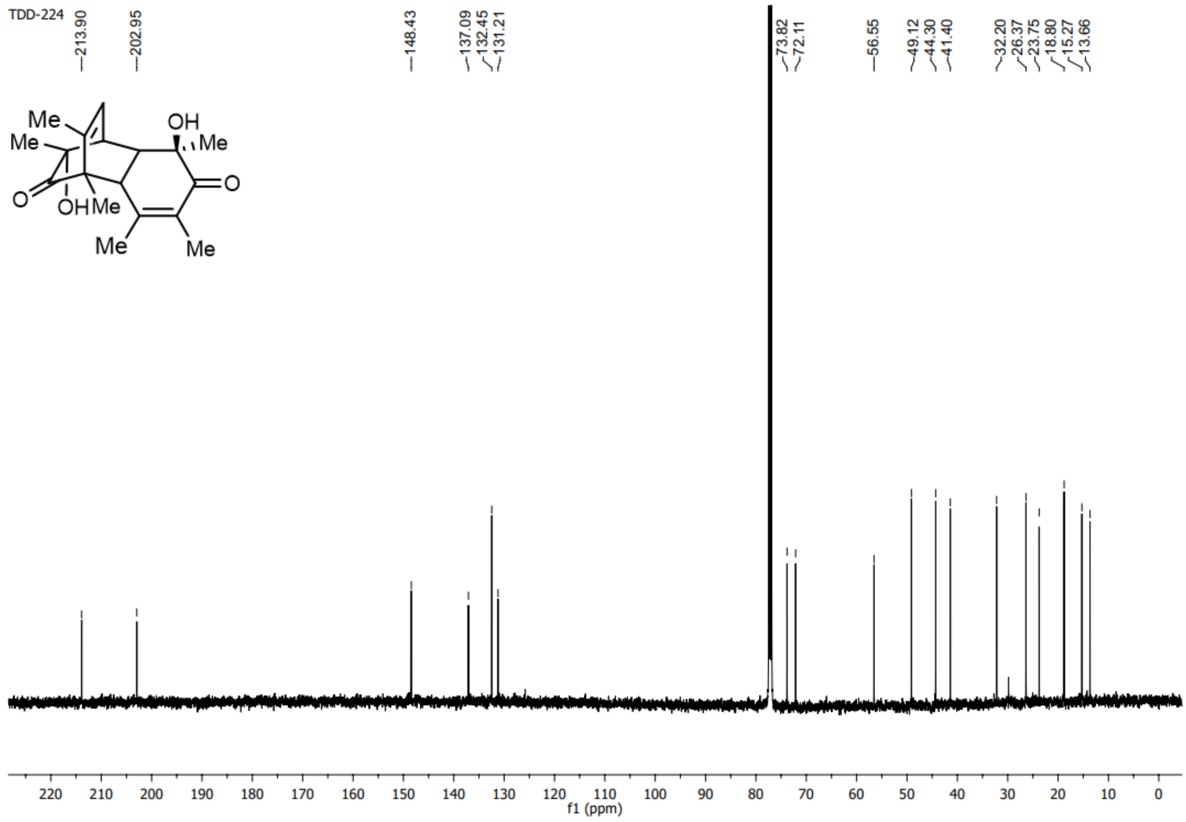
TDD-224
213.90
202.95
148.43
137.09
132.45
131.21
73.82
72.11
56.55
49.12
44.30
41.40
32.20
26.37
23.75
18.80
15.27
13.66
Me
Me
OH
Me
O
OH
Me
O
Me
Me
220
210
200
190
180
170
160
150
140
130
120
110
100
90
80
70
60
50
40
30
20
10
0
f1 (ppm)

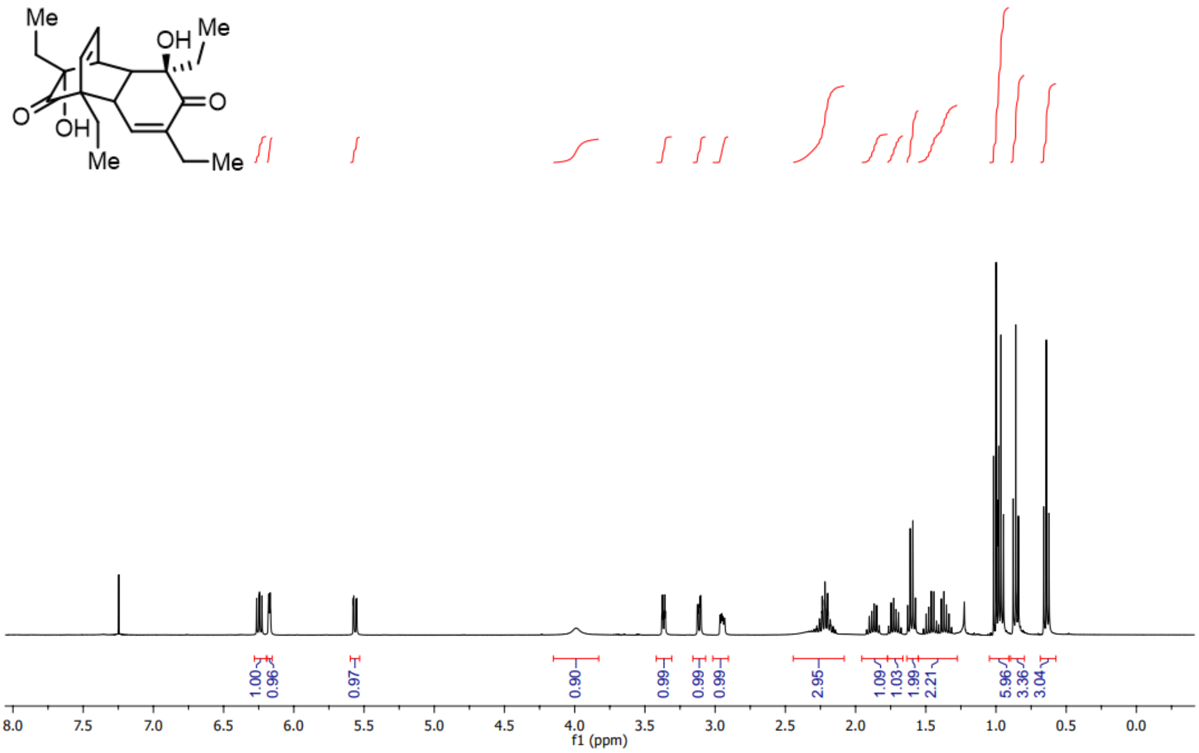
Me
OH
Me
O
OH
O
Me
Me
1.00
0.96
0.97
0.90
0.99
0.99
0.99
2.95
1.09
1.03
1.99
2.21
5.96
3.36
3.04
f1 (ppm)

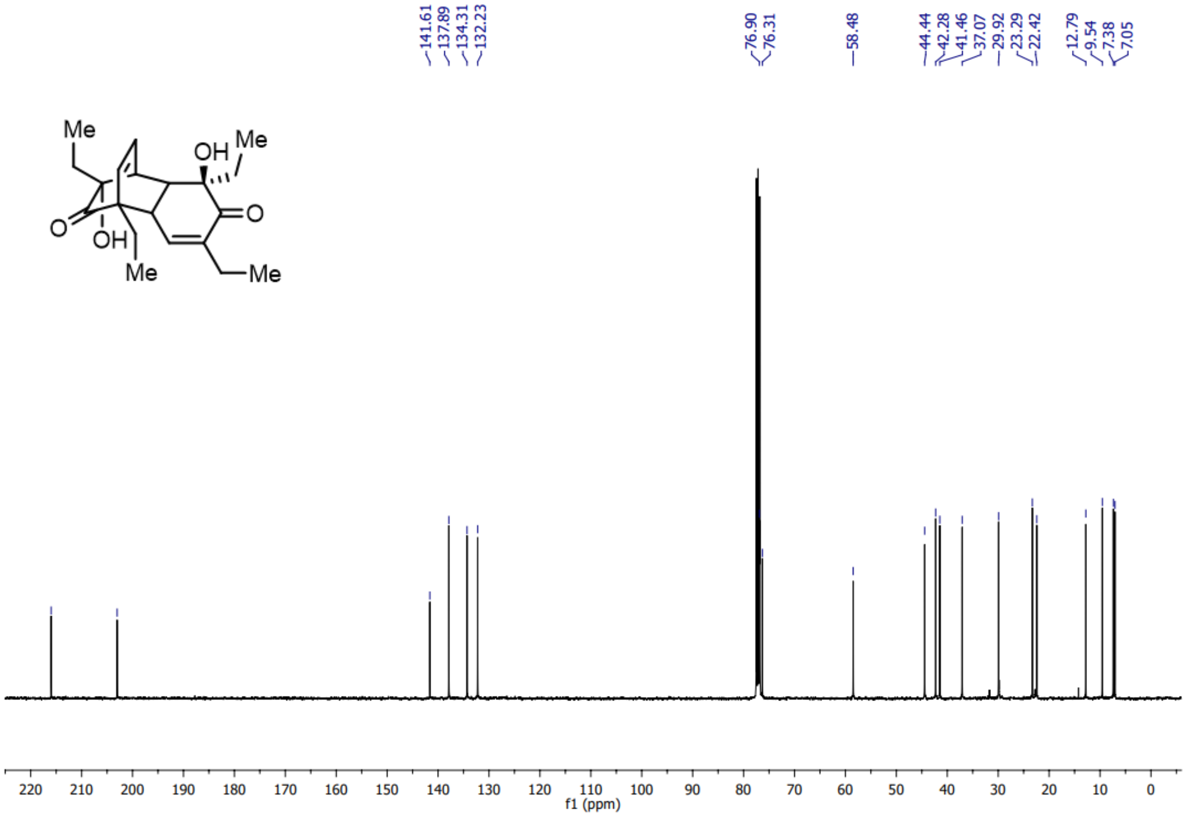
141.61
137.89
134.31
132.23
76.90
76.31
58.48
44.44
42.28
41.46
37.07
29.92
23.29
22.42
12.79
9.54
7.38
7.05
Me
OH
Me
O
OH
O
Me
Me
f1 (ppm)

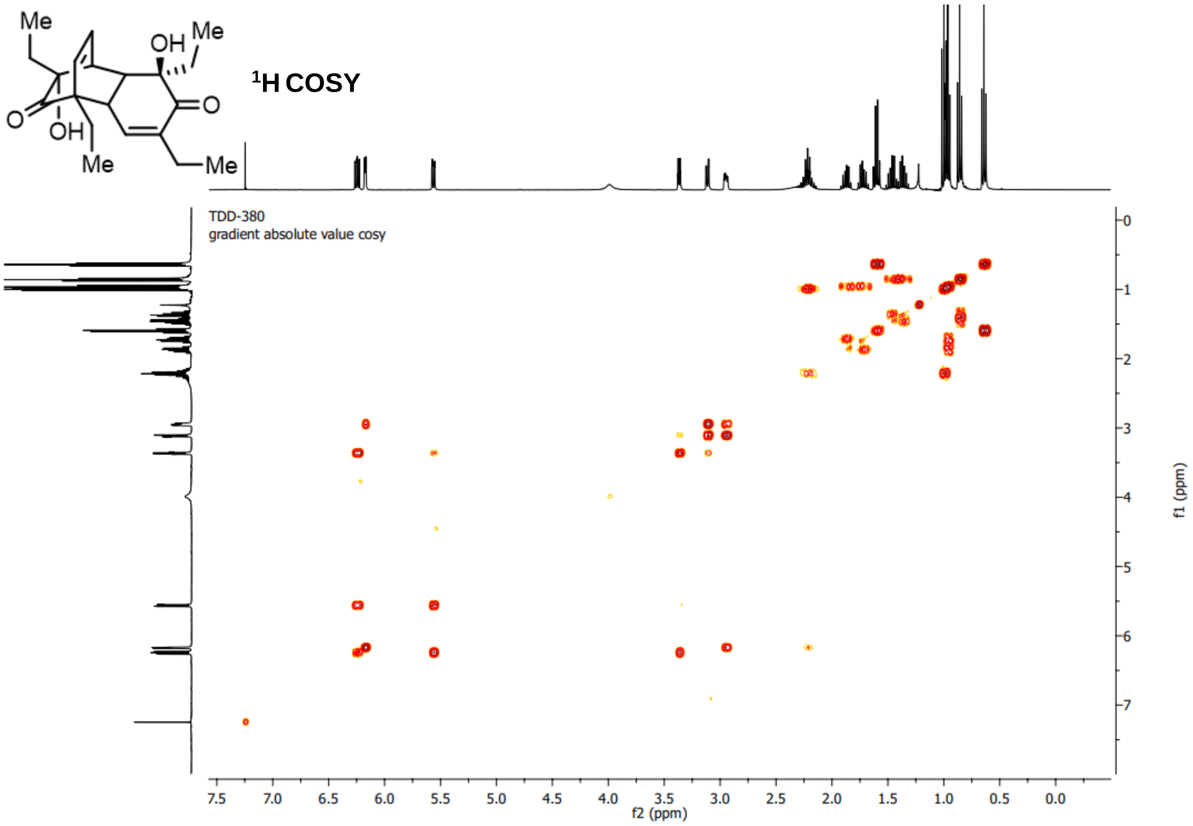

Me
OH
Me
O
OH
Me
O
Me
^{1}H COSY
TDD-380
gradient absolute value cosy
f2 (ppm)
f1 (ppm)

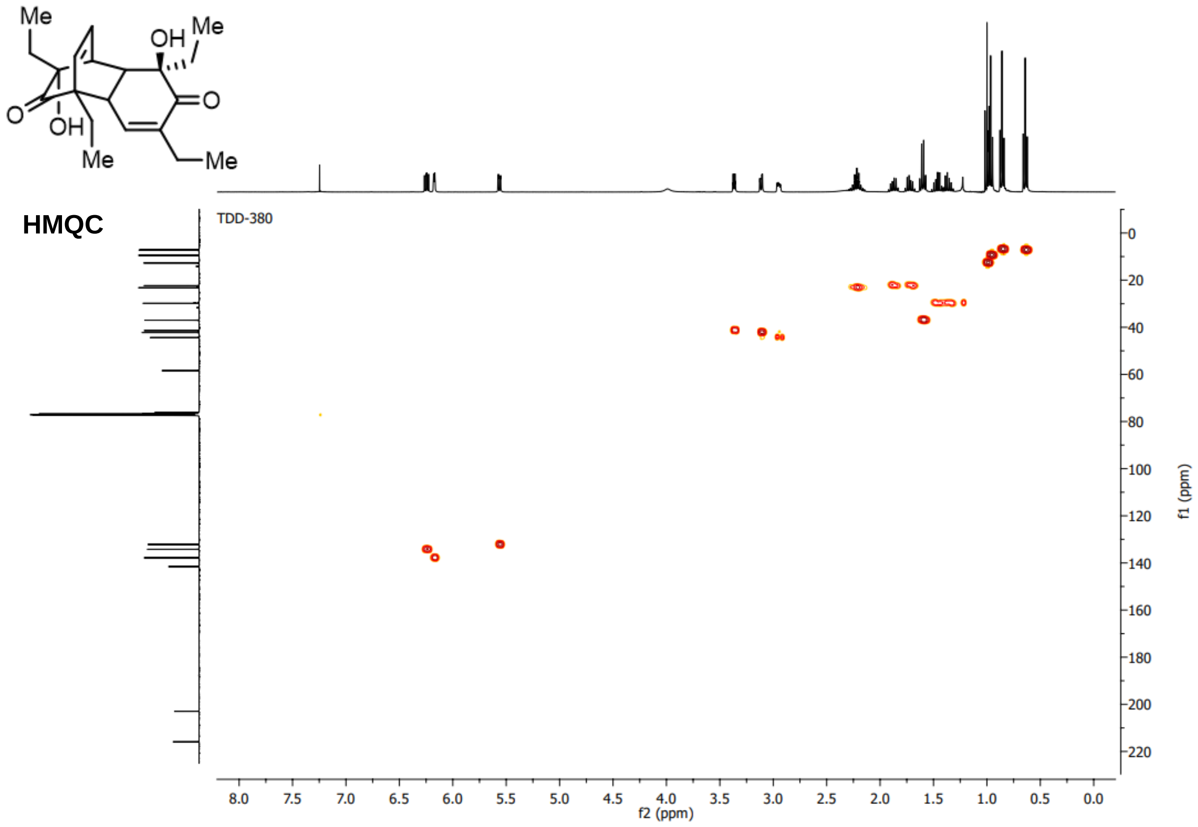

Me
OH
Me
O
OH
Me
O
Me
HMQC
TDD-380
f2 (ppm)
f1 (ppm)

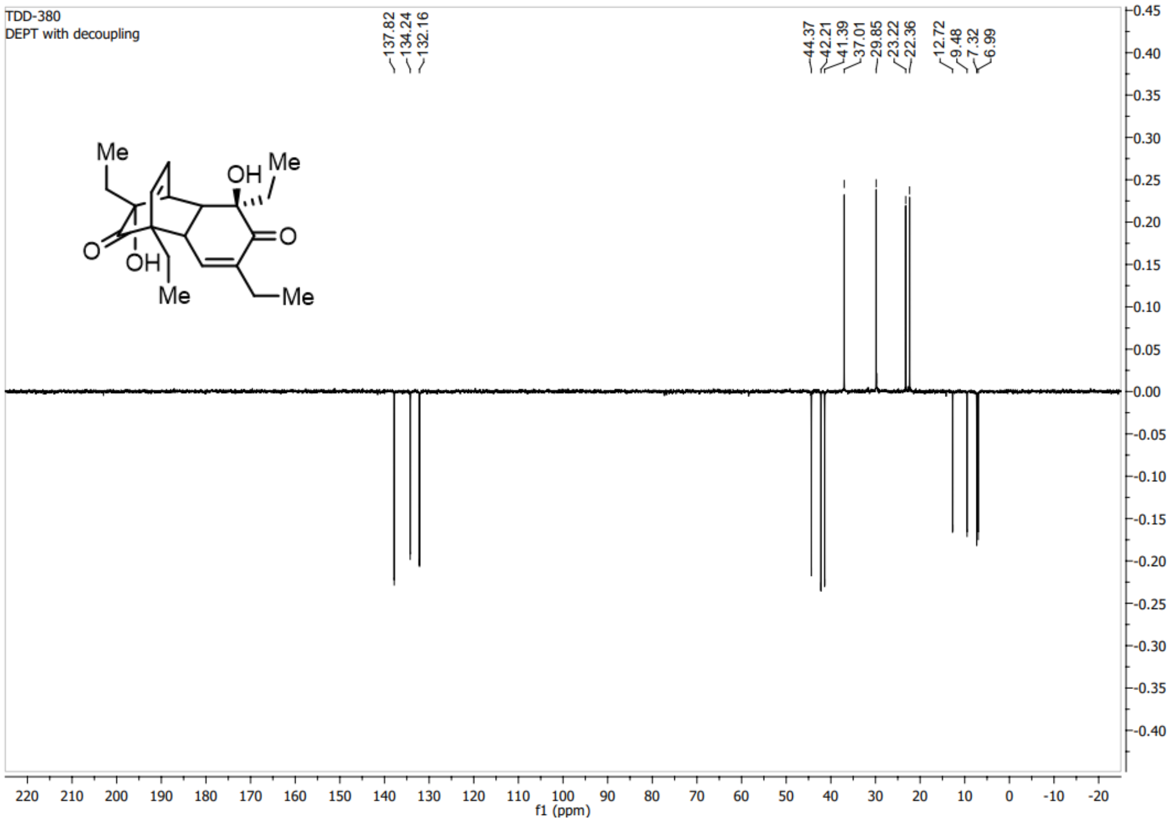
TDD-380
DEPT with decoupling
137.82
134.24
132.16
44.37
42.21
41.39
37.01
29.85
23.22
22.36
12.72
9.48
7.32
6.99
f1 (ppm)

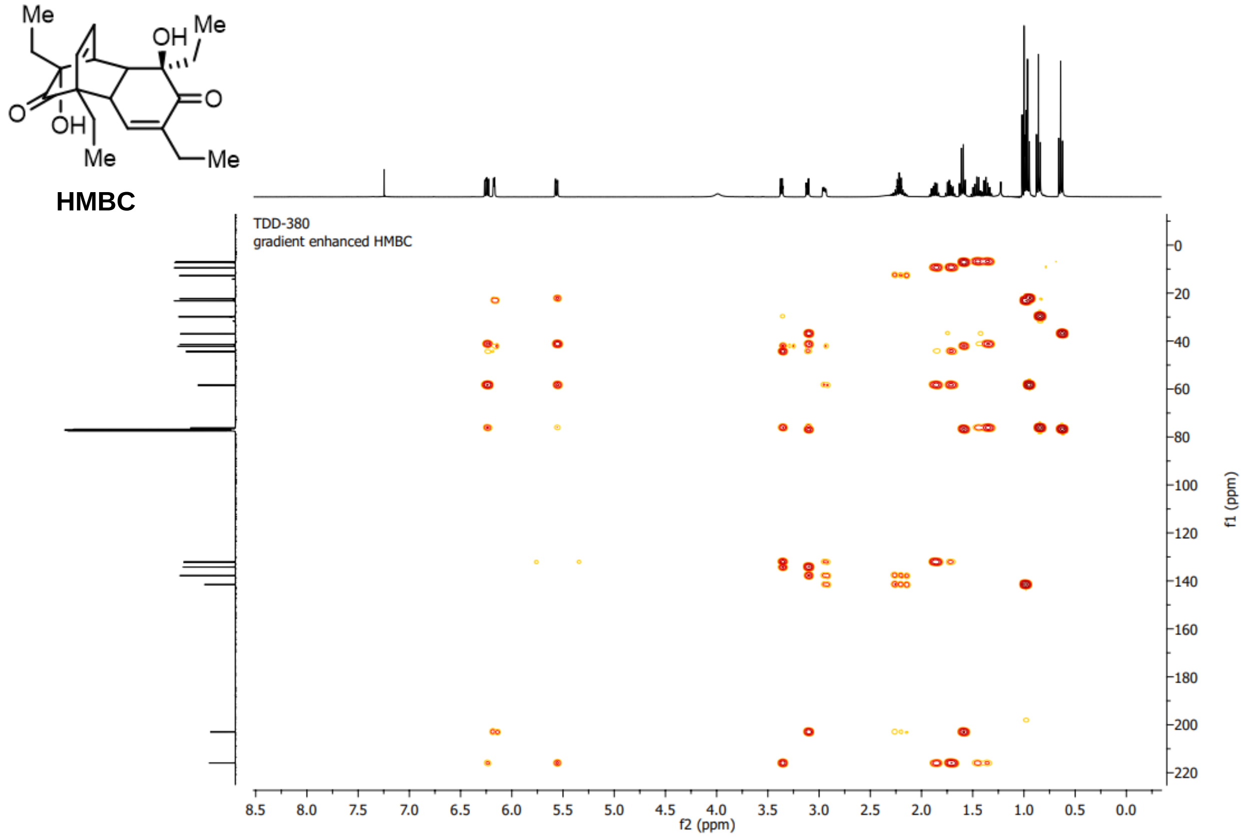
HMBC
TDD-380
gradient enhanced HMBC
f2 (ppm)
f1 (ppm)

key NOE enhancements

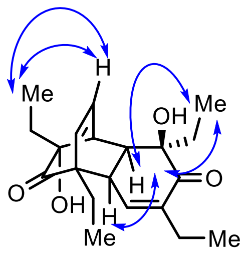

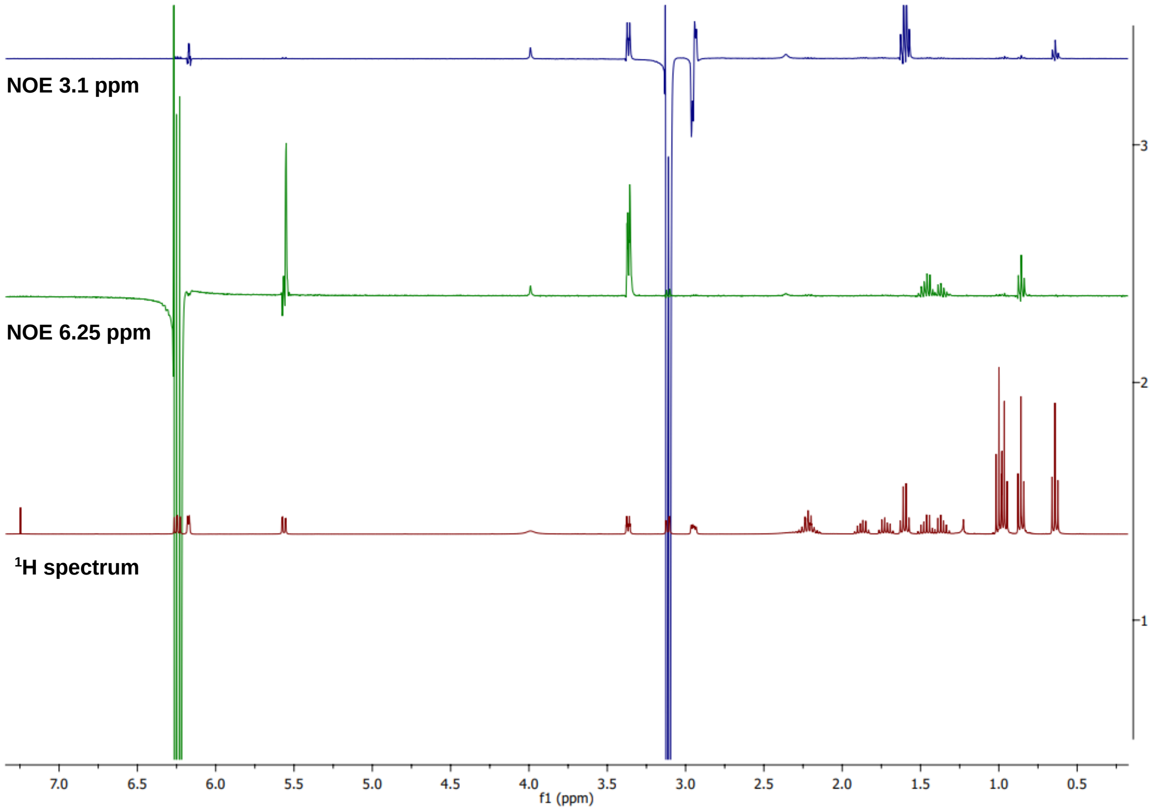

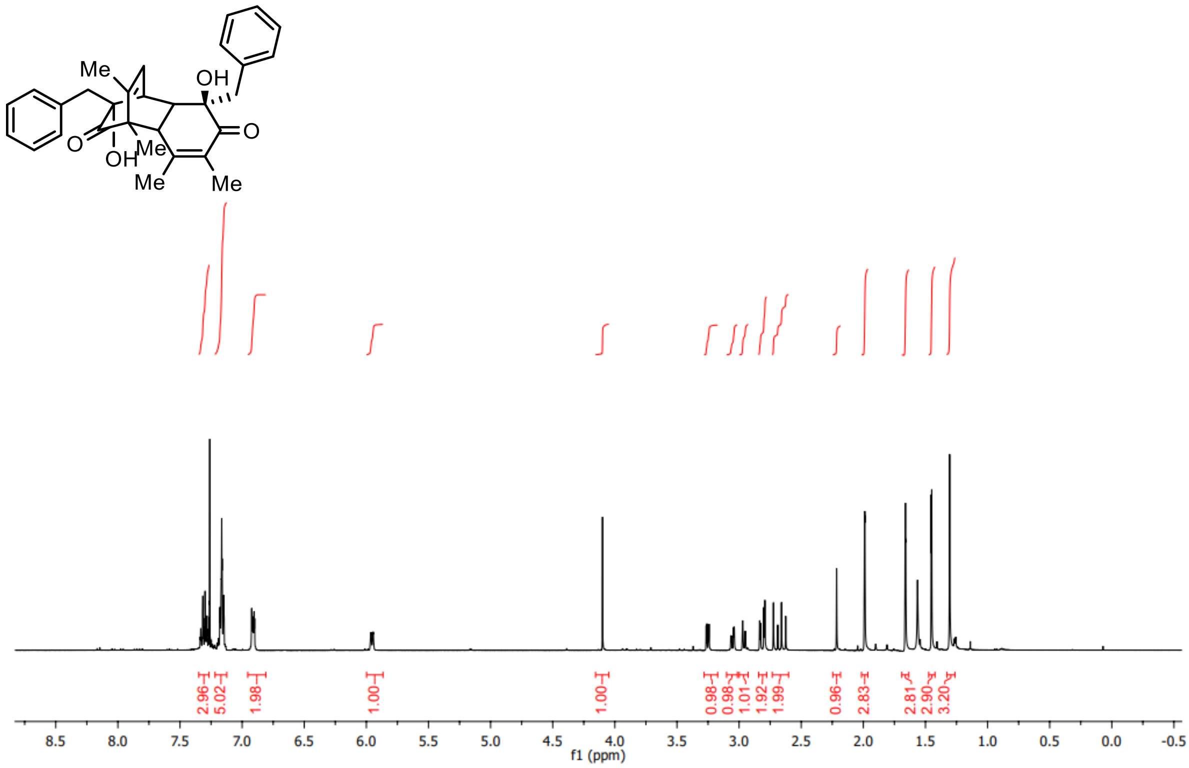

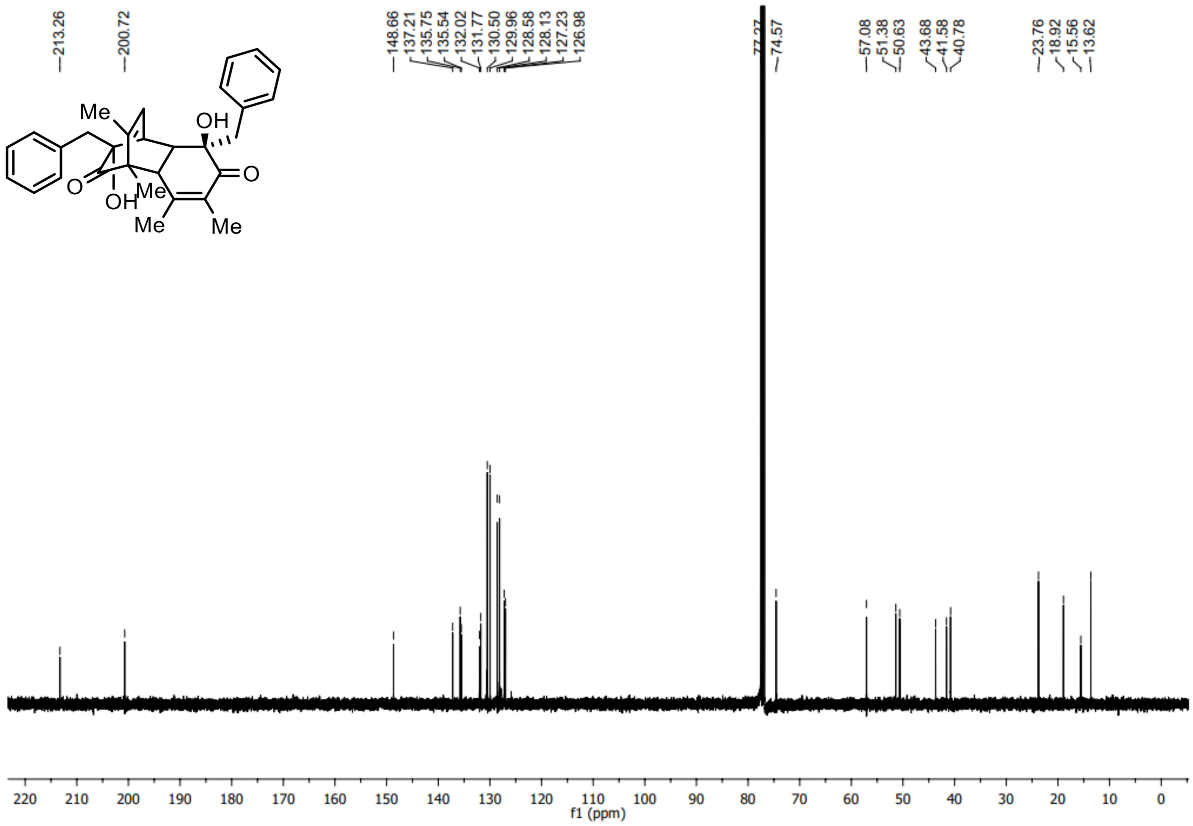

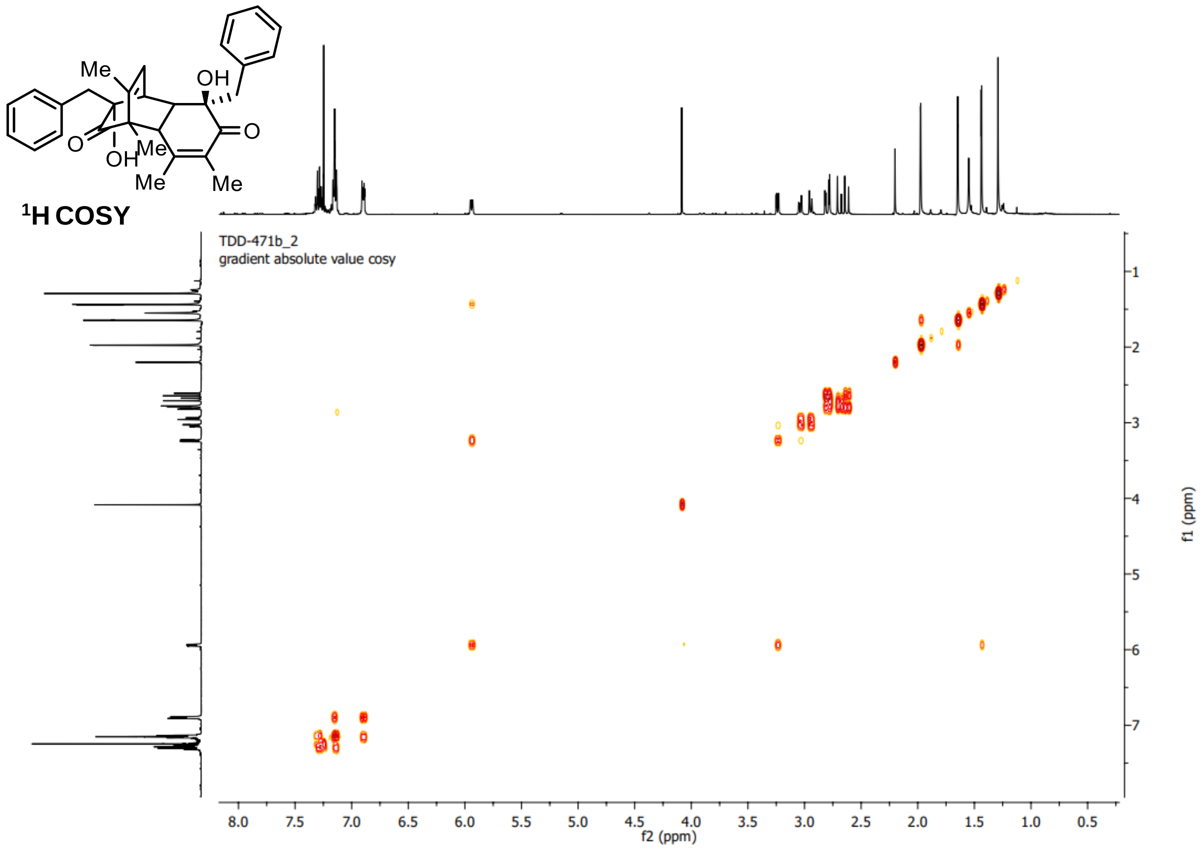
Me
OH
O
OH
Me
Me
Me
O
¹H COSY
TDD-471b_2
gradient absolute value cosy
f2 (ppm)
f1 (ppm)

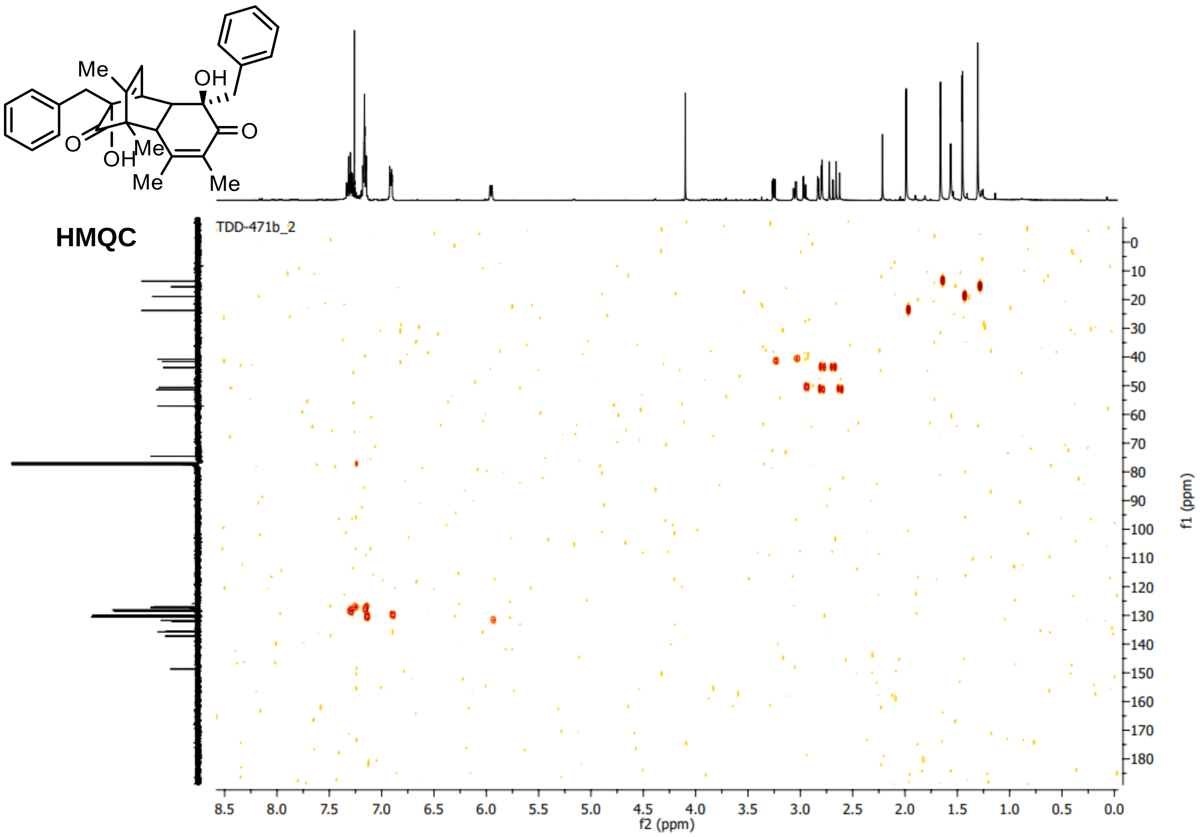
Me
OH
O
OH
Me
Me
Me
O
HMQC
TDD-471b_2
f2 (ppm)
f1 (ppm)

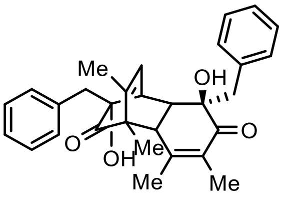
Me
OH
O
O
OH
Me
Me
Me

HMBC

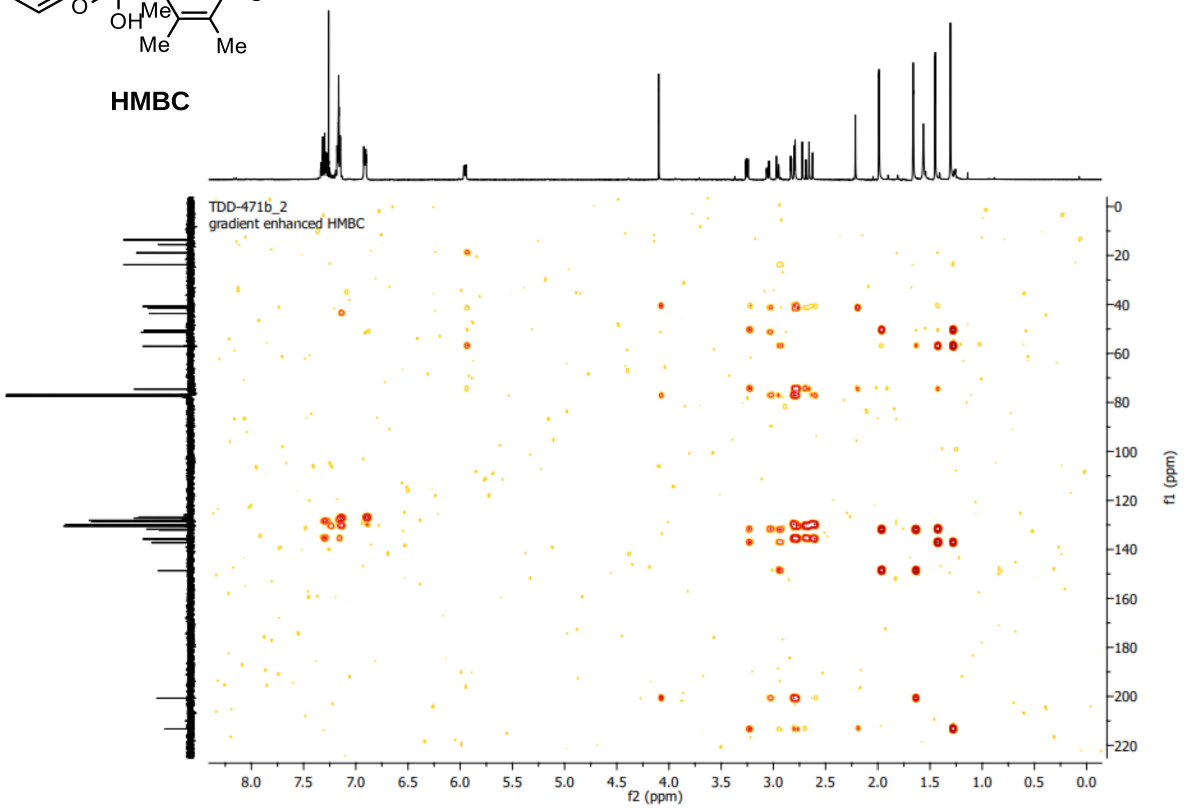
TDD-471b_2
gradient enhanced HMBC
f2 (ppm)
f1 (ppm)

key NOE enhancements

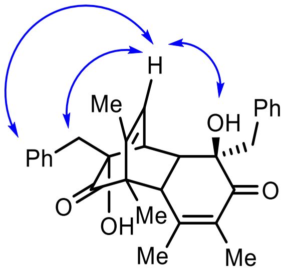

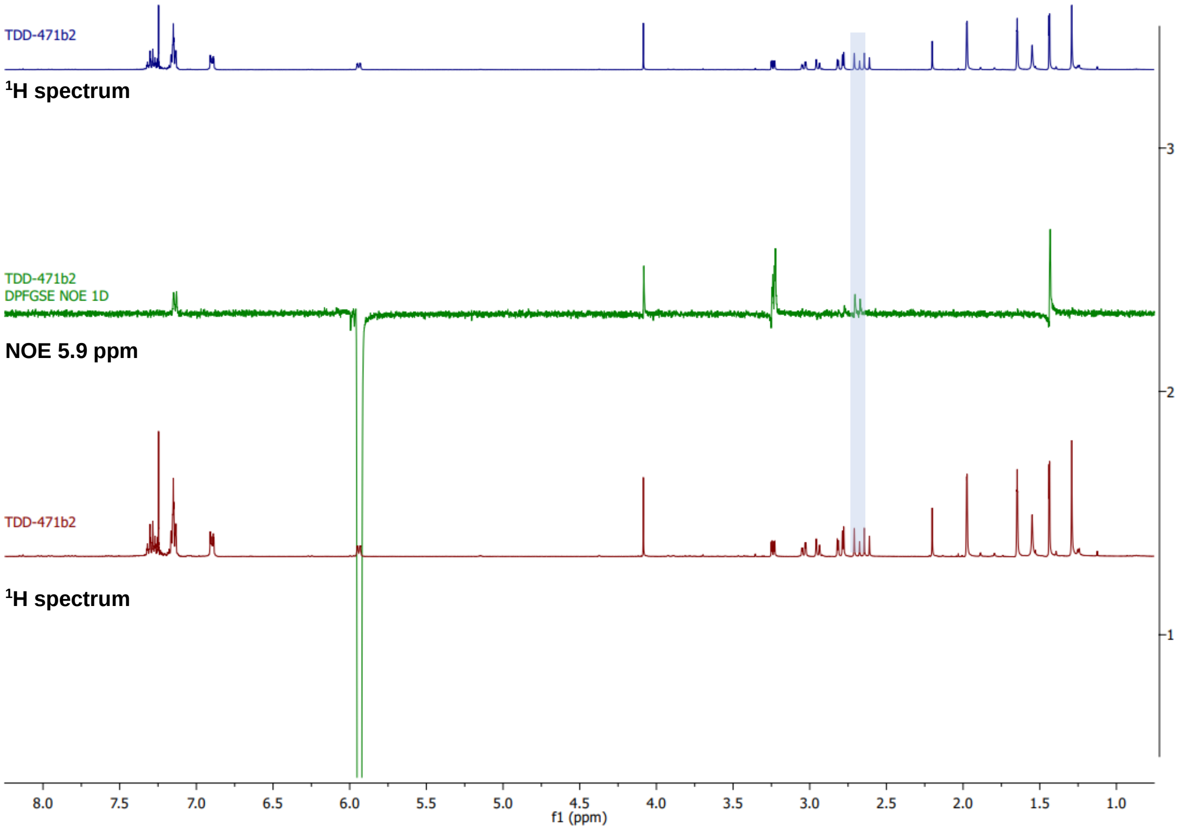

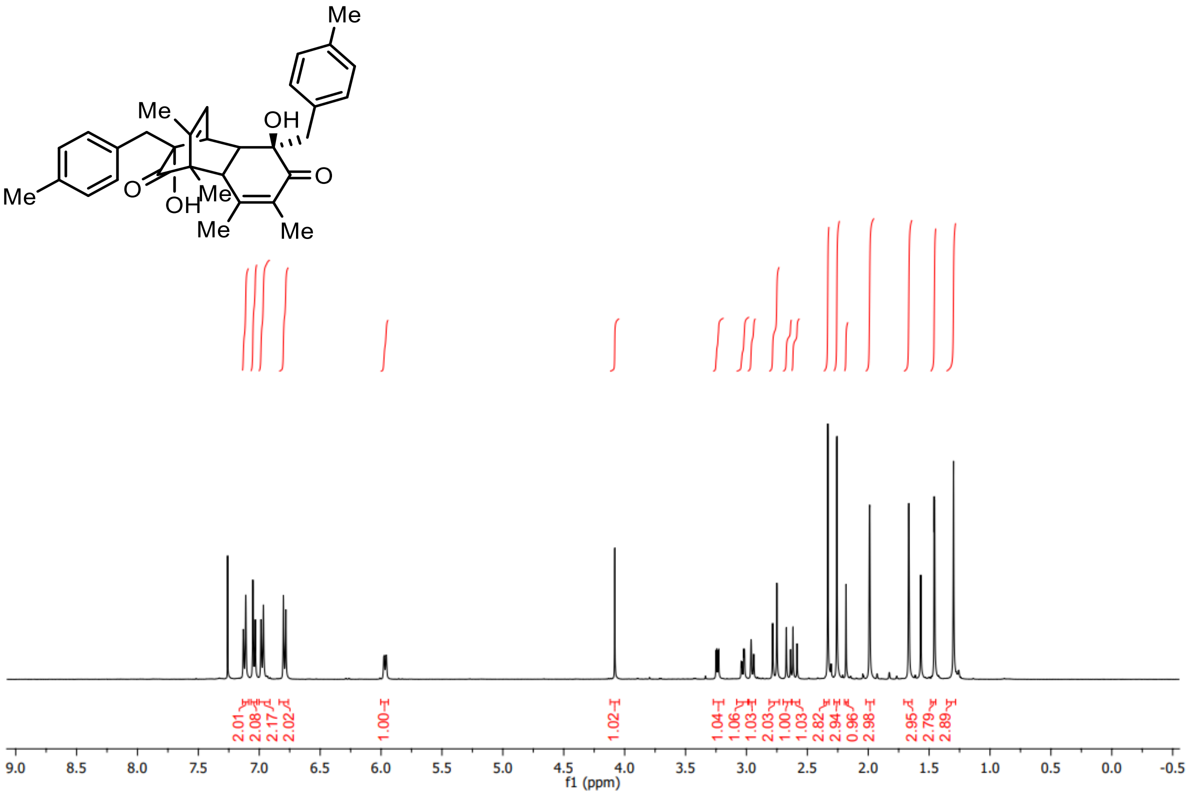

Me
OH
O
Me
O
OH
Me
Me
Me
Me
2.01
2.08
2.17
2.02
1.00
1.02
1.04
1.06
1.03
2.03
1.00
1.03
2.82
2.94
0.96
2.98
2.95
2.79
2.89
f1 (ppm)

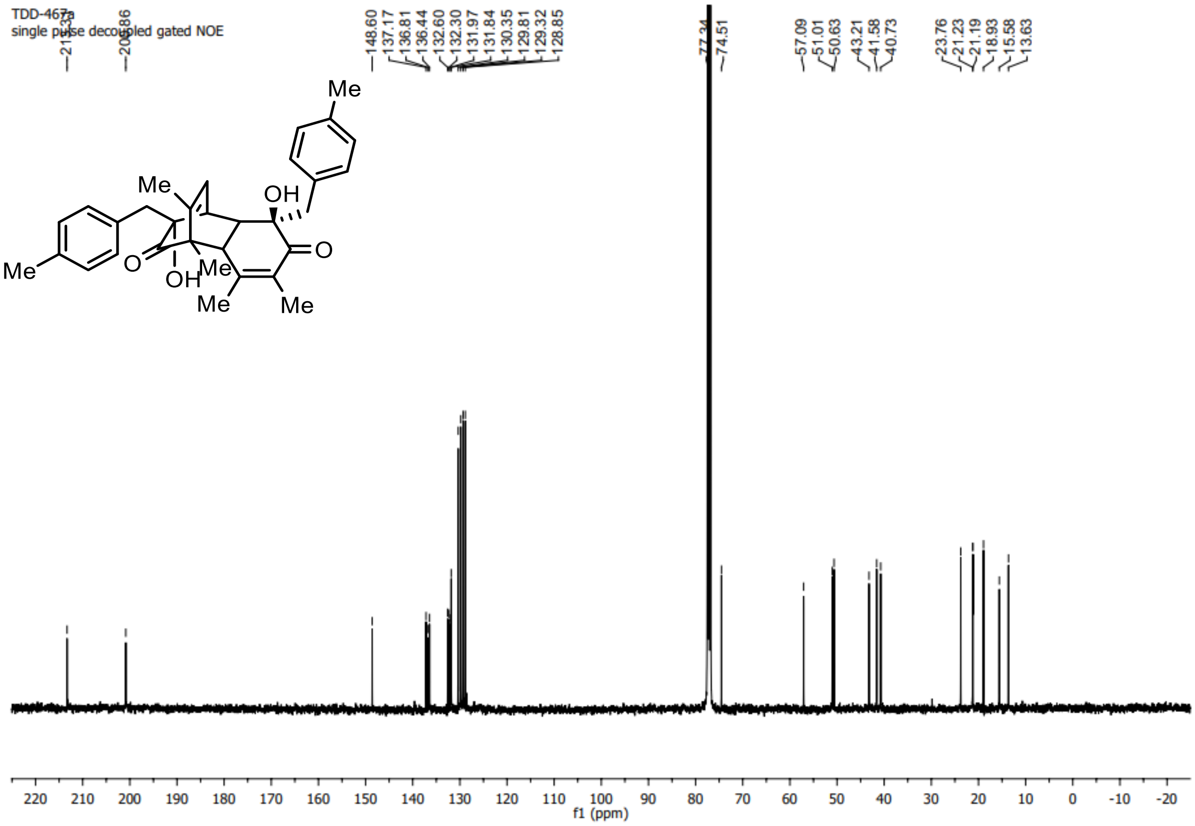

single pulse decoupled gated NOE
148.60
137.17
136.81
136.44
132.60
132.30
131.97
131.84
130.35
129.81
129.32
128.85
74.51
57.09
51.01
50.63
43.21
41.58
40.73
23.76
21.23
21.19
18.93
15.58
13.63
Me
OH
O
Me
O
OH
Me
Me
Me
Me
f1 (ppm)

^{1}H COSY

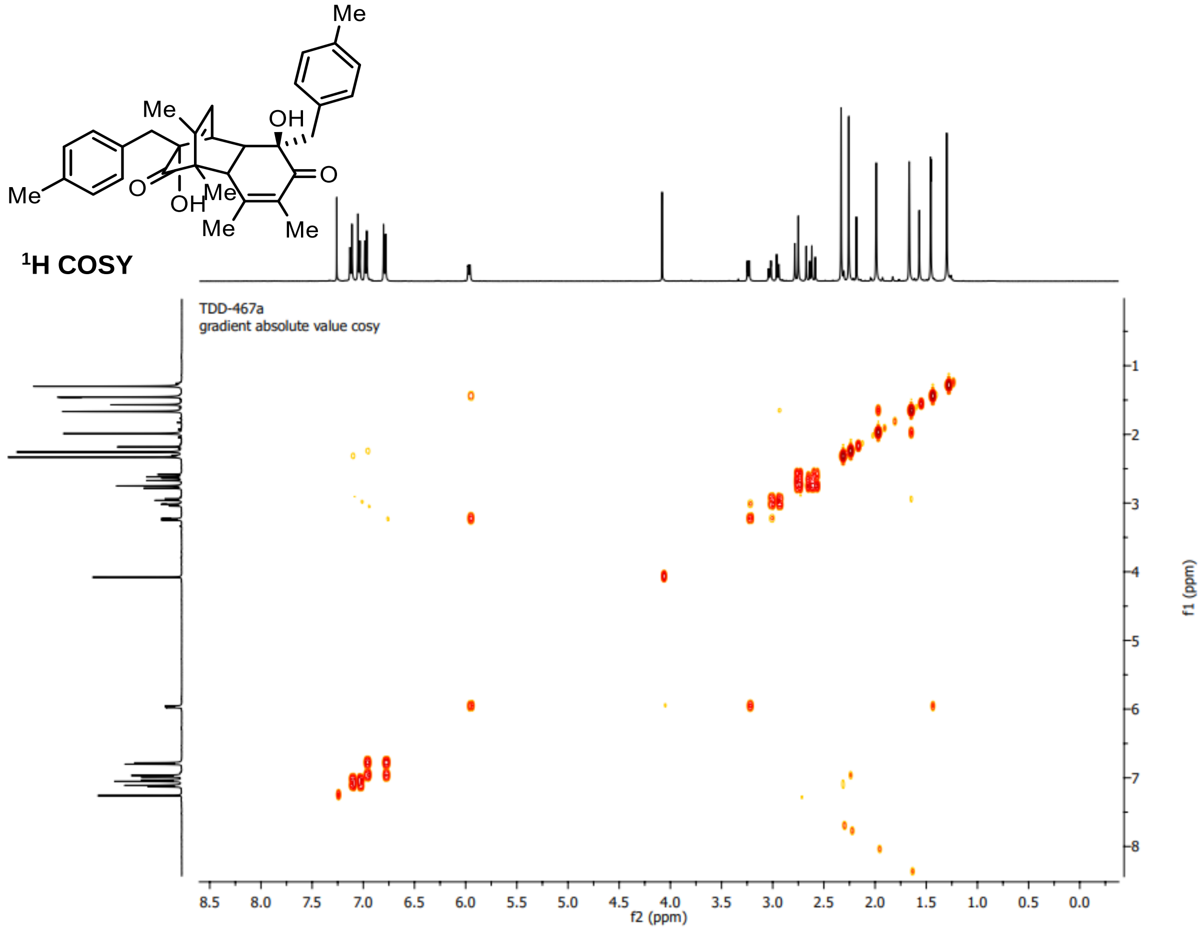
TDD-467a
gradient absolute value cosy
f2 (ppm)
f1 (ppm)

HMQC

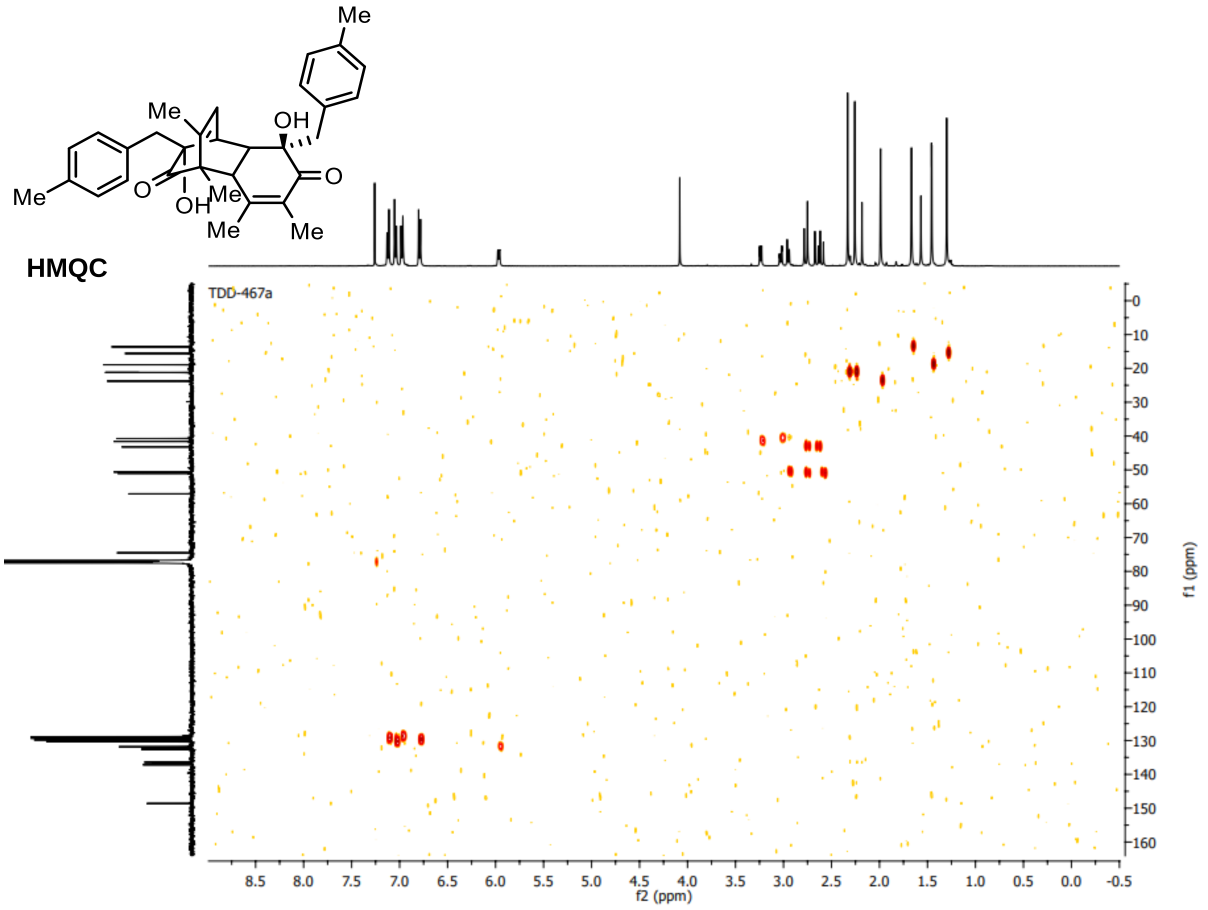
TDD-467a
f2 (ppm)
f1 (ppm)

HMBC

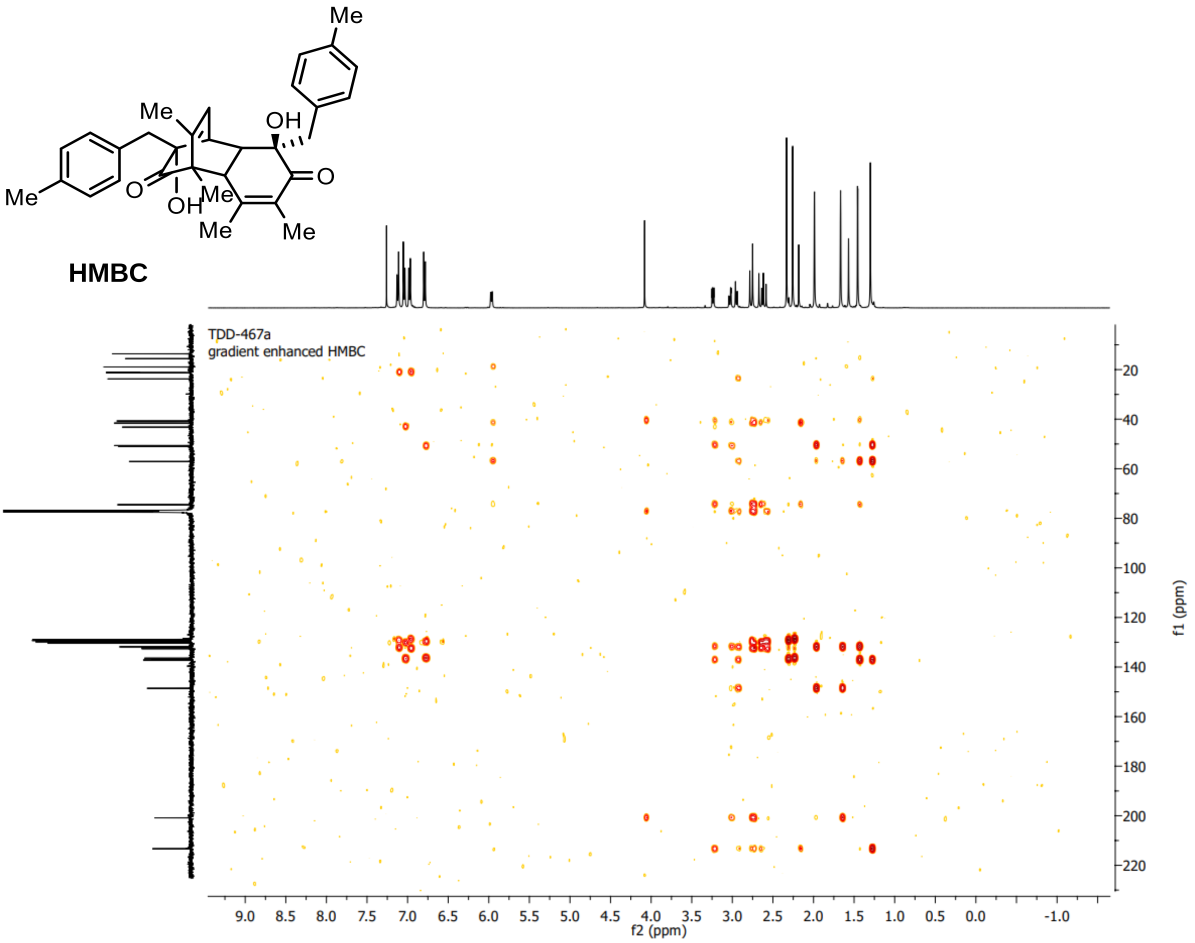
Me
OH
O
Me
O
OH
Me
Me
Me
Me
TDD-467a
gradient enhanced HMBC
f2 (ppm)
f1 (ppm)

key NOE enhancements

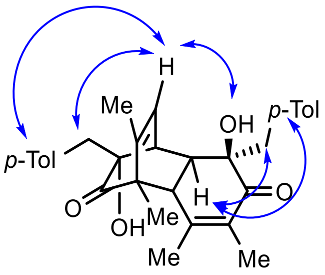

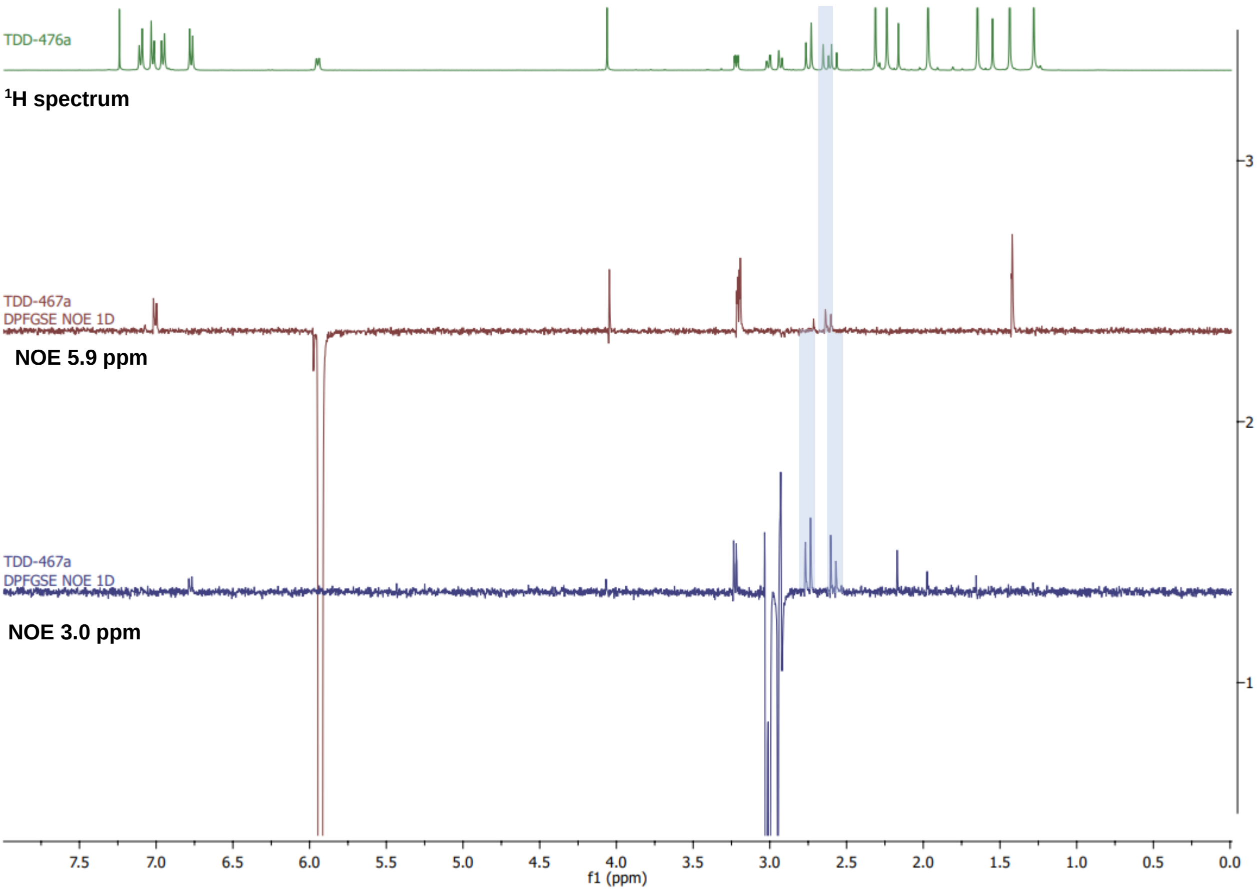

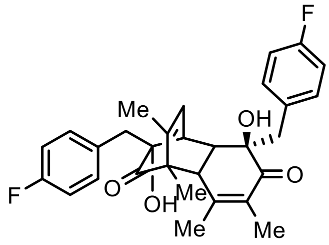
F
Me
OH
O
F
O
OH
Me
Me
Me

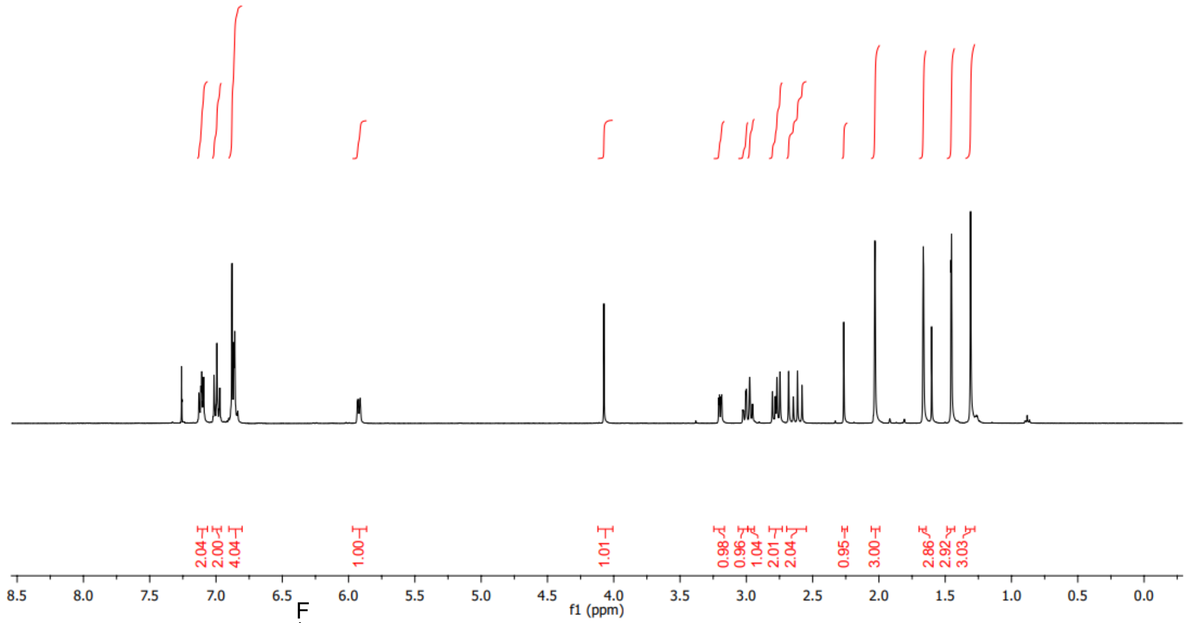
2.04
2.00
4.04
1.00
1.01
0.98
0.96
1.04
2.01
2.04
0.95
3.00
2.86
2.92
3.03
8.5
8.0
7.5
7.0
6.5
6.0
5.5
5.0
4.5
4.0
3.5
3.0
2.5
2.0
1.5
1.0
0.5
0.0
f1 (ppm)

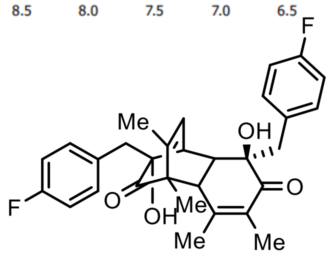
F
Me
OH
O
F
O
OH
Me
Me
Me

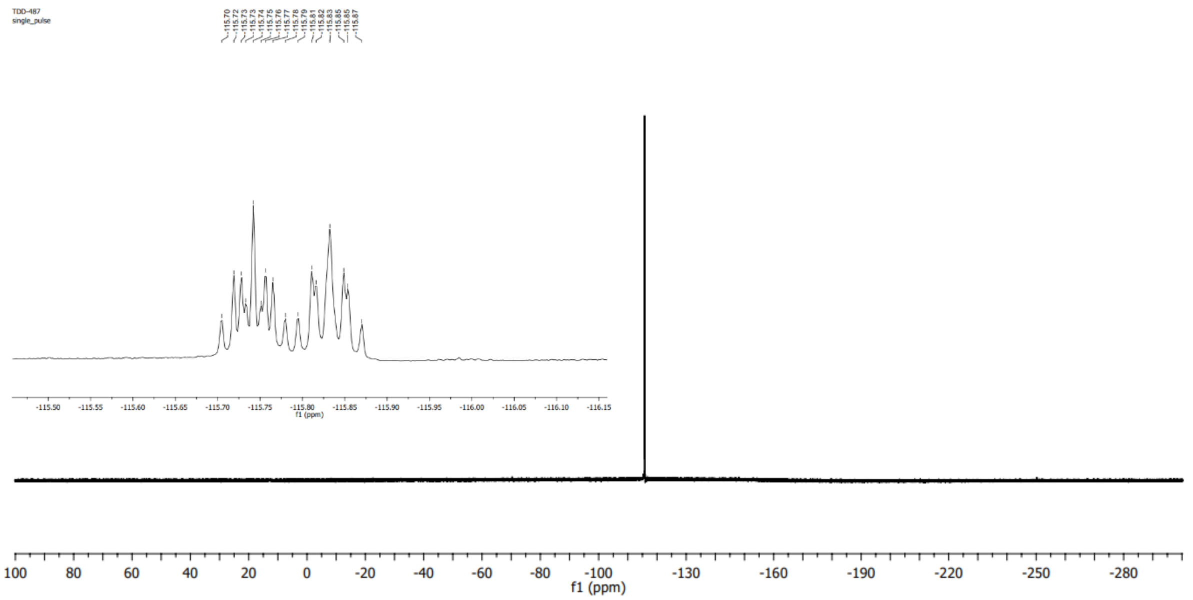
100
80
60
40
20
0
-20
-40
-60
-80
-100
-130
-160
-190
-220
-250
-280
f1 (ppm)

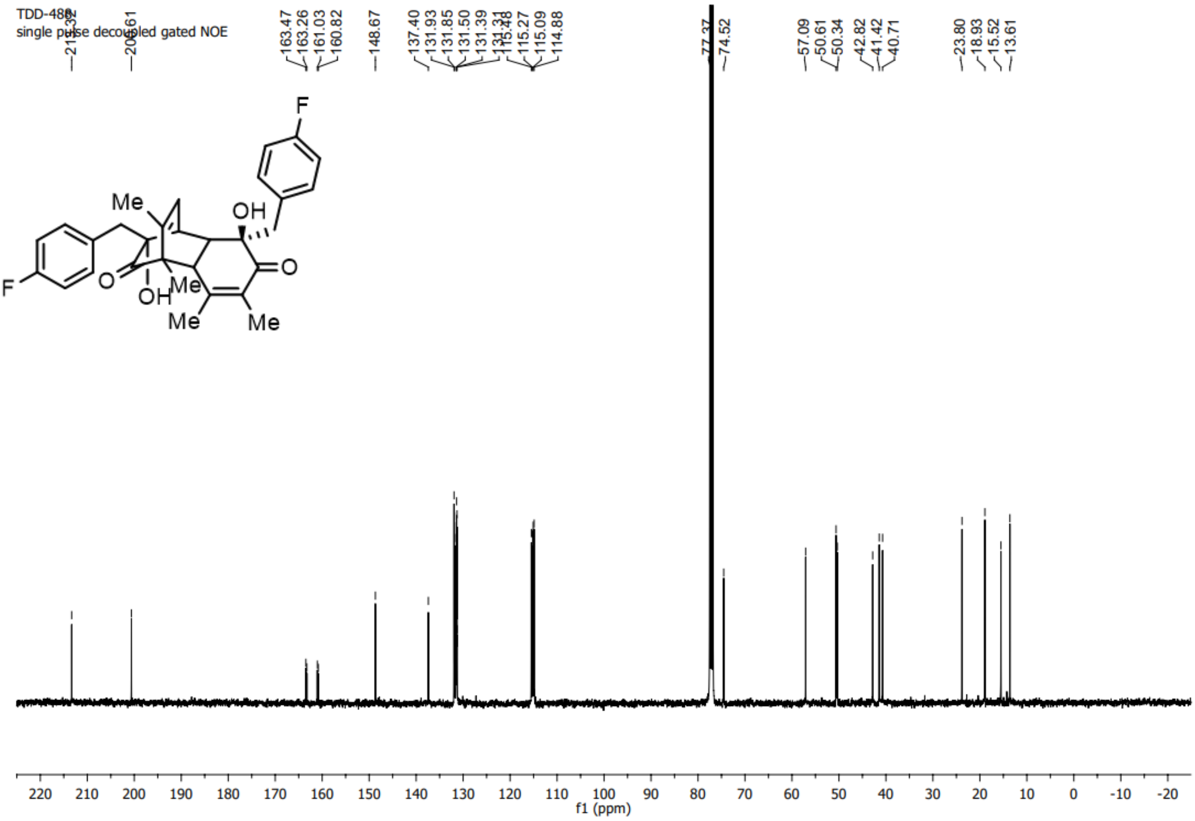

TDD-488
single pulse decoupled gated NOE
163.47
163.26
161.03
160.82
148.67
137.40
131.93
131.85
131.50
131.39
131.31
115.48
115.27
115.09
114.88
77.37
74.52
57.09
50.61
50.34
42.82
41.42
40.71
23.80
18.93
15.52
13.61
F
OH
Me
O
OH
Me
Me
Me
O
220 210 200 190 180 170 160 150 140 130 120 110 100 90 80 70 60 50 40 30 20 10 0 -10 -20
f1 (ppm)

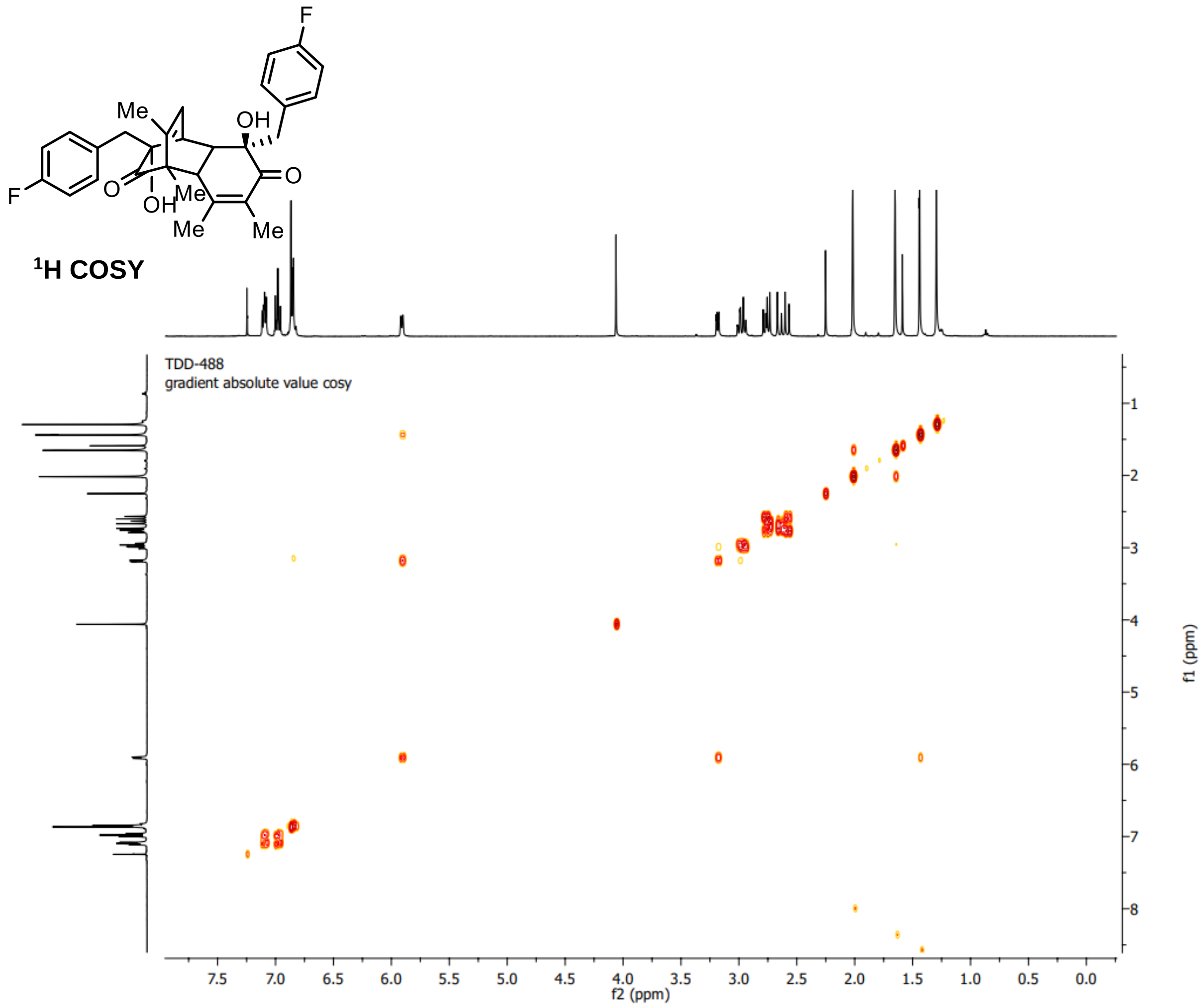

F
Me
OH
O
OH
Me
Me
Me
O
¹H COSY
TDD-488
gradient absolute value cosy
7.5 7.0 6.5 6.0 5.5 5.0 4.5 4.0 3.5 3.0 2.5 2.0 1.5 1.0 0.5 0.0
f2 (ppm)
1 2 3 4 5 6 7 8
f1 (ppm)

HMQC

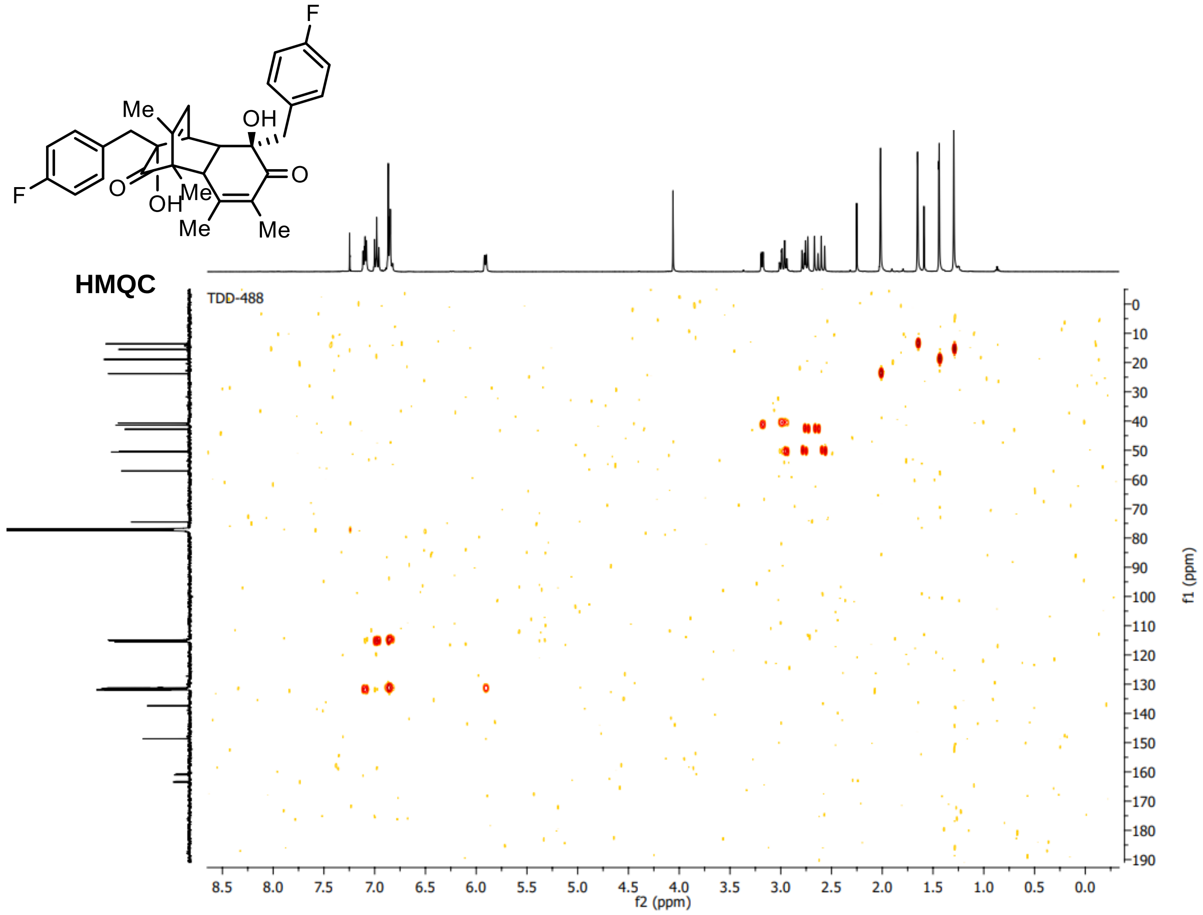

F
Me
OH
O
O
F
OH
Me
Me
Me
TDD-488
f2 (ppm)
f1 (ppm)

HMBC

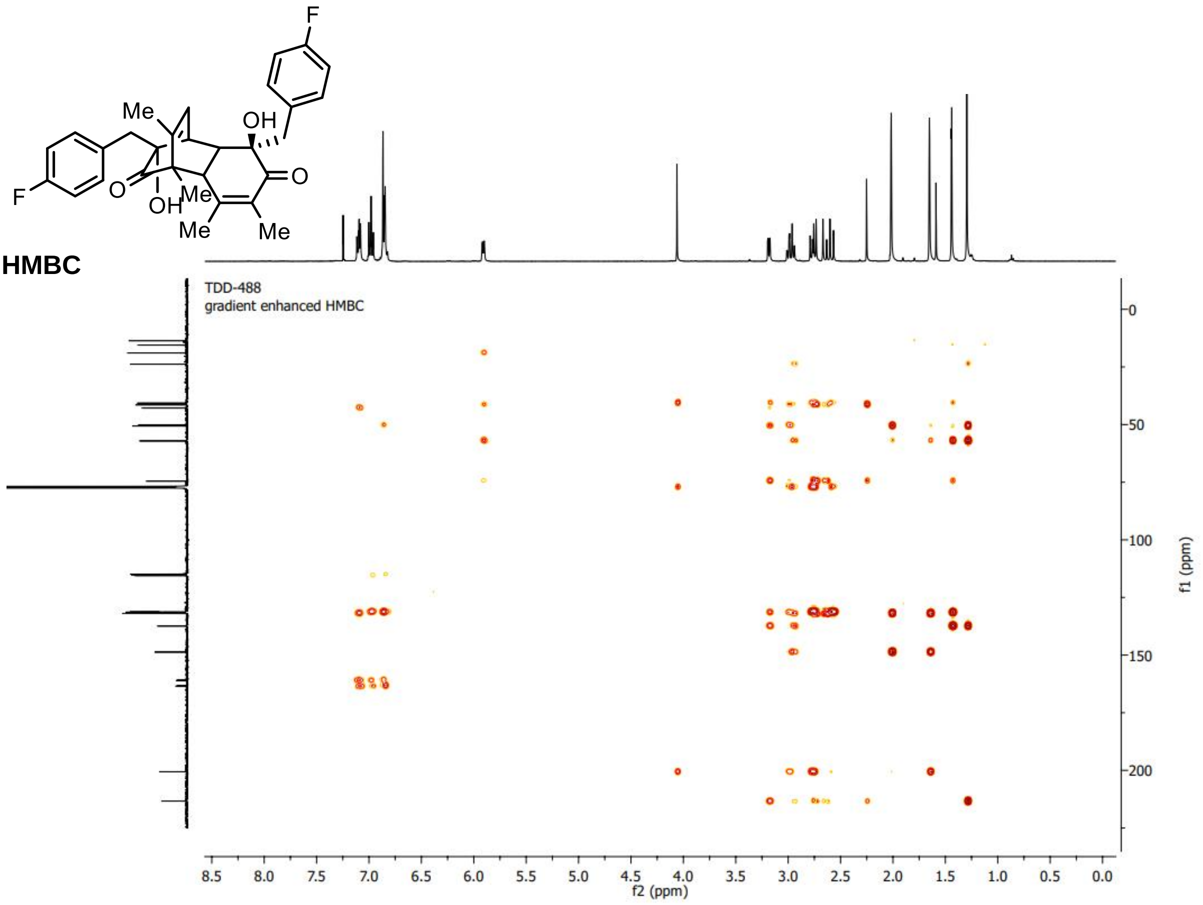

F
Me
OH
O
O
F
OH
Me
Me
Me
TDD-488
gradient enhanced HMBC
f2 (ppm)
f1 (ppm)

key NOE enhancements

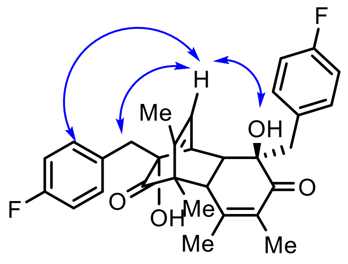

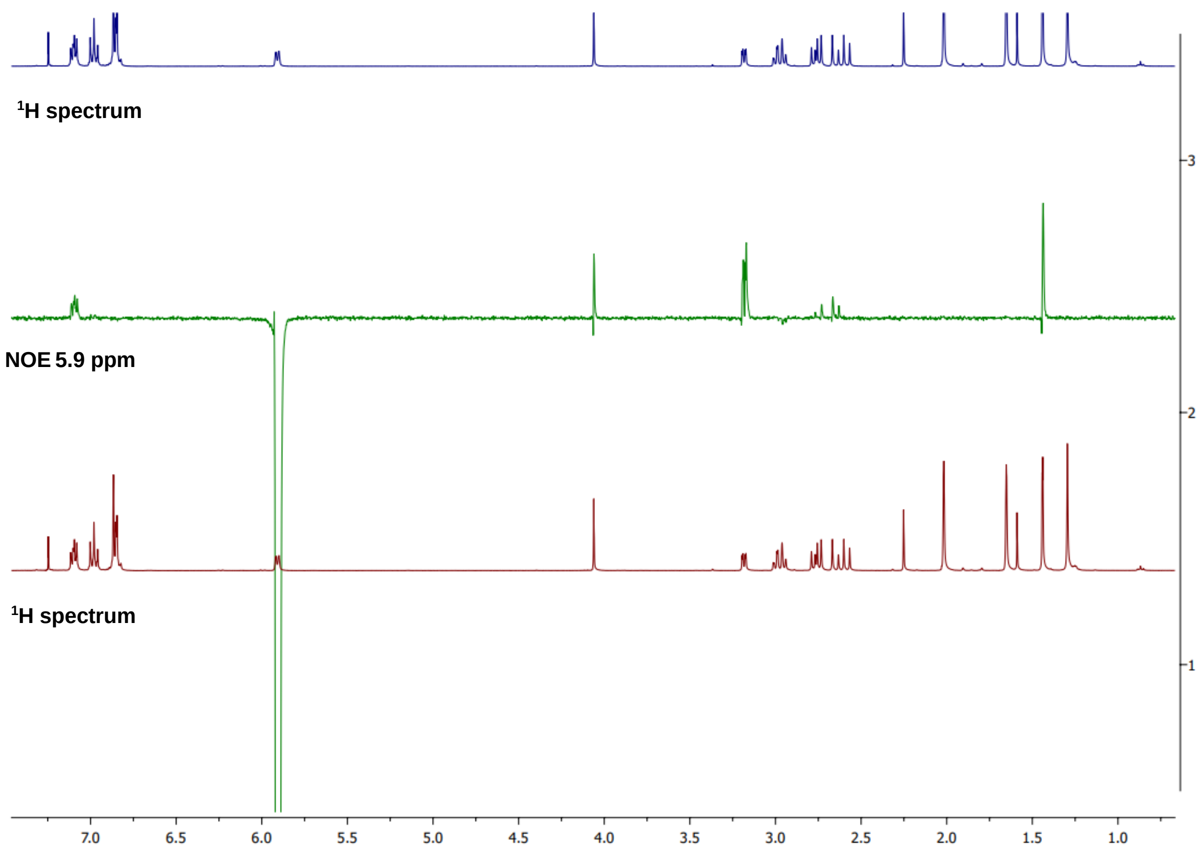

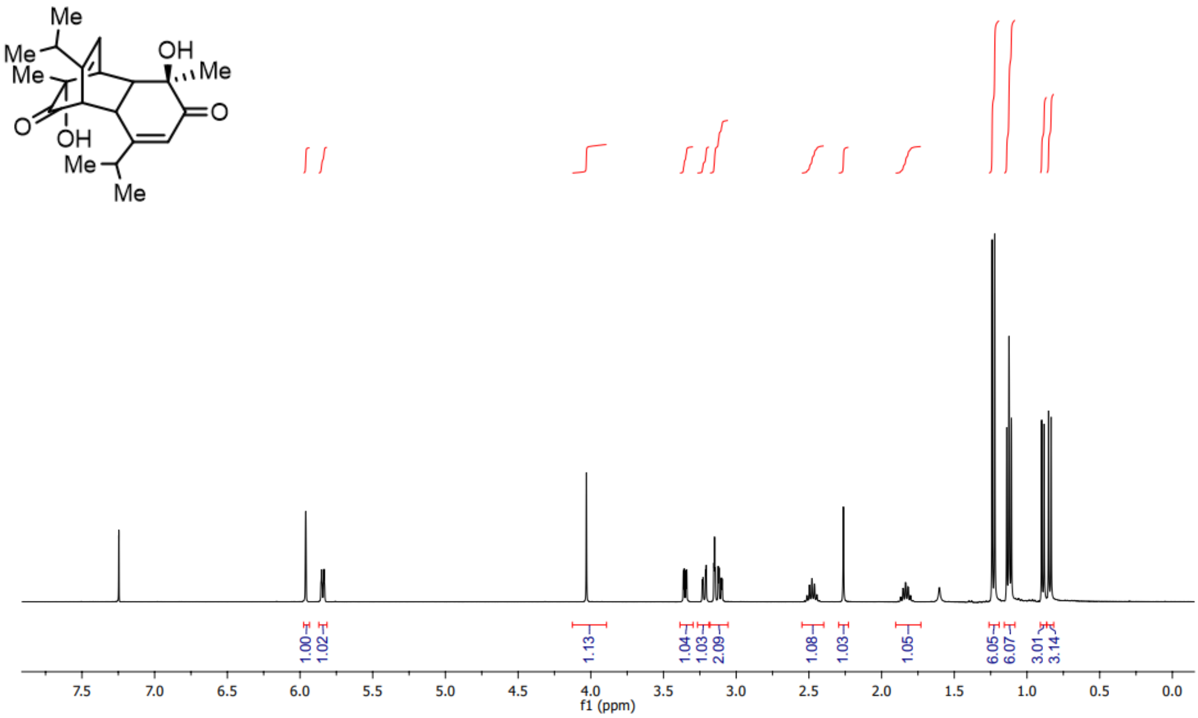

1.00
1.02
1.13
1.04
1.03
2.09
1.08
1.03
1.05
6.05
6.07
3.01
3.14
7.5
7.0
6.5
6.0
5.5
5.0
4.5
4.0
3.5
3.0
2.5
2.0
1.5
1.0
0.5
0.0
f1 (ppm)

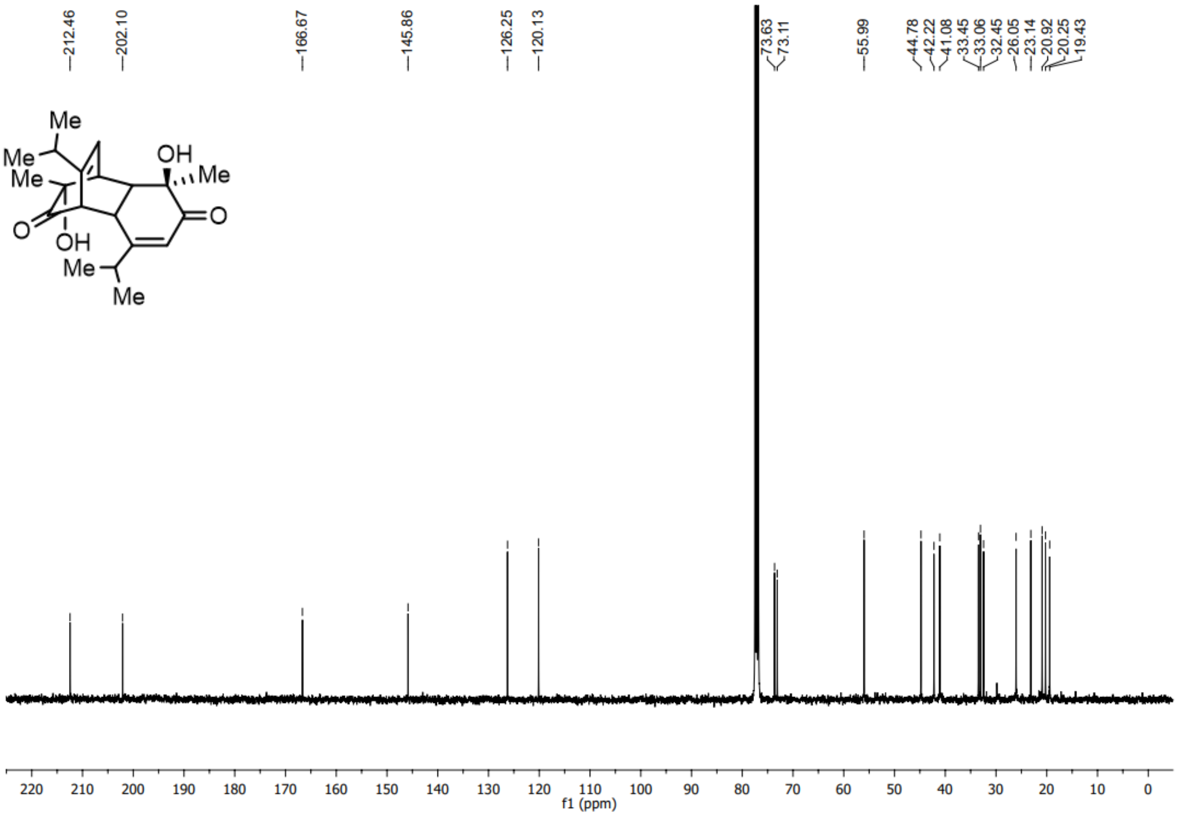

212.46
202.10
166.67
145.86
126.25
120.13
73.63
73.11
55.99
44.78
42.22
41.08
33.45
33.06
32.45
26.05
23.14
20.92
20.25
19.43
220
210
200
190
180
170
160
150
140
130
120
110
100
90
80
70
60
50
40
30
20
10
0
f1 (ppm)

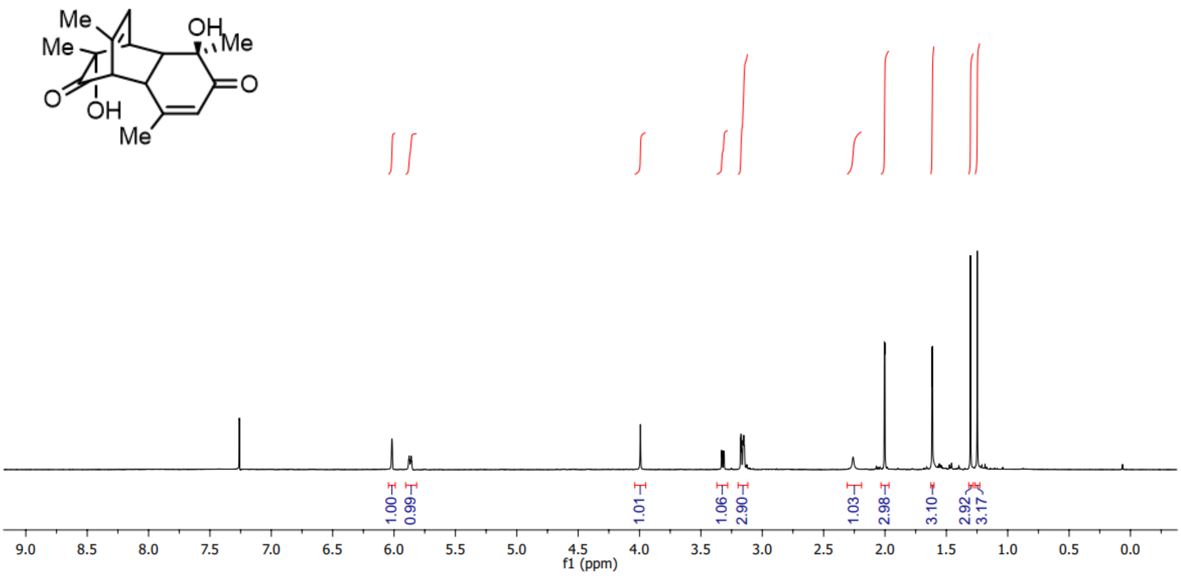
Me
Me
O
OH
Me
OH
Me
O
1.00
0.99
1.01
1.06
2.90
1.03
2.98
3.10
2.92
3.17
9.0 8.5 8.0 7.5 7.0 6.5 6.0 5.5 5.0 4.5 4.0 3.5 3.0 2.5 2.0 1.5 1.0 0.5 0.0
f1 (ppm)

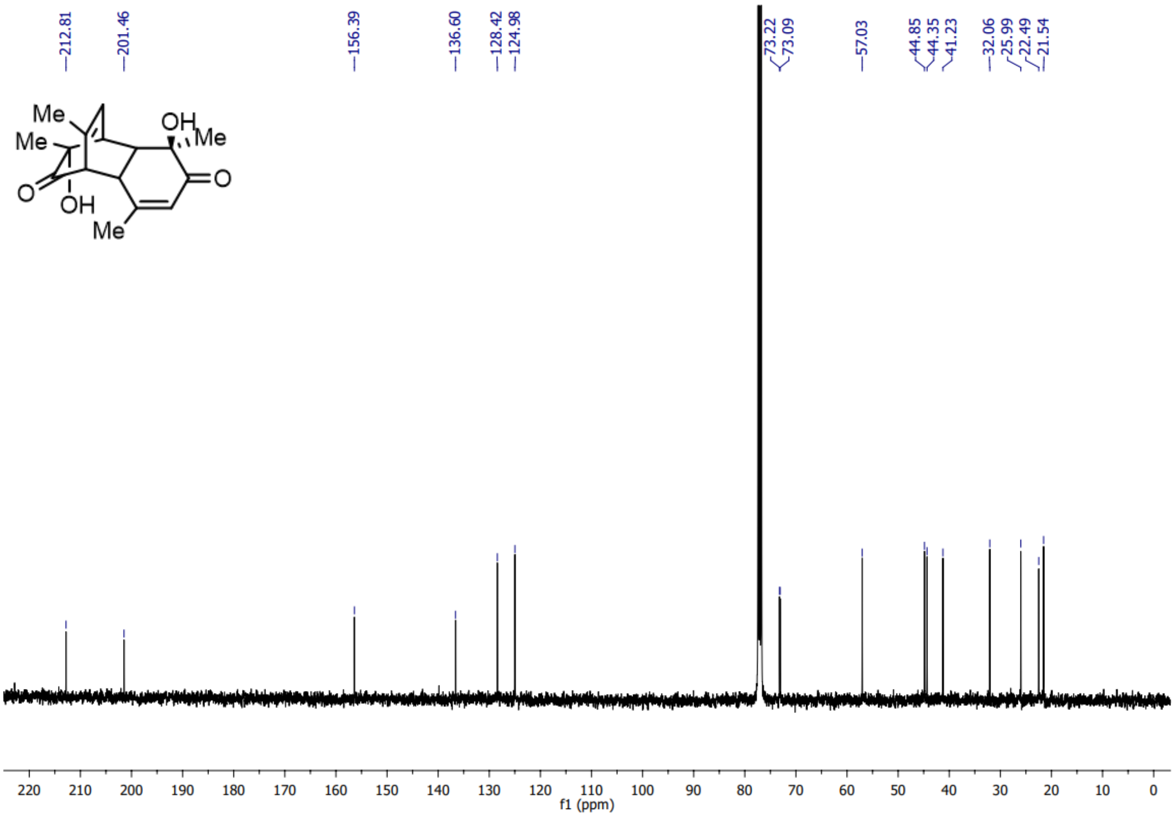
212.81
201.46
156.39
136.60
128.42
124.98
73.22
73.09
57.03
44.85
44.35
41.23
32.06
25.99
22.49
21.54
Me
Me
O
OH
Me
OH
Me
O
220 210 200 190 180 170 160 150 140 130 120 110 100 90 80 70 60 50 40 30 20 10 0
f1 (ppm)

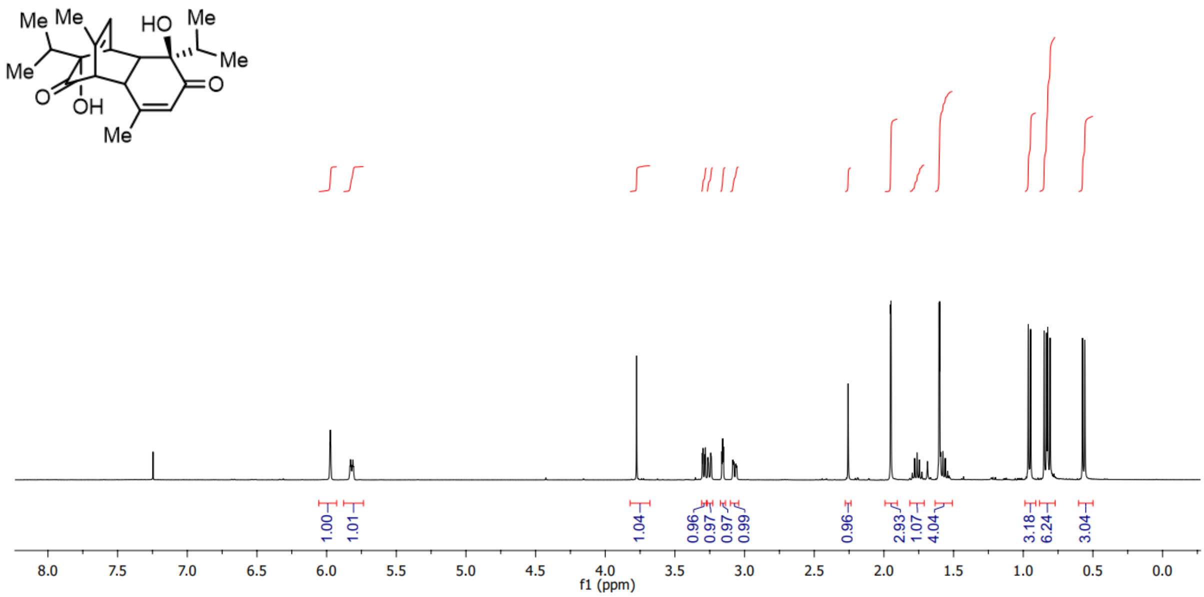
Me Me HO Me
Me
Me
O OH O
Me
1.00
1.01
1.04
0.96
0.97
0.97
0.99
0.96
2.93
1.07
4.04
3.18
6.24
3.04
8.0 7.5 7.0 6.5 6.0 5.5 5.0 4.5 4.0 3.5 3.0 2.5 2.0 1.5 1.0 0.5 0.0
f1 (ppm)

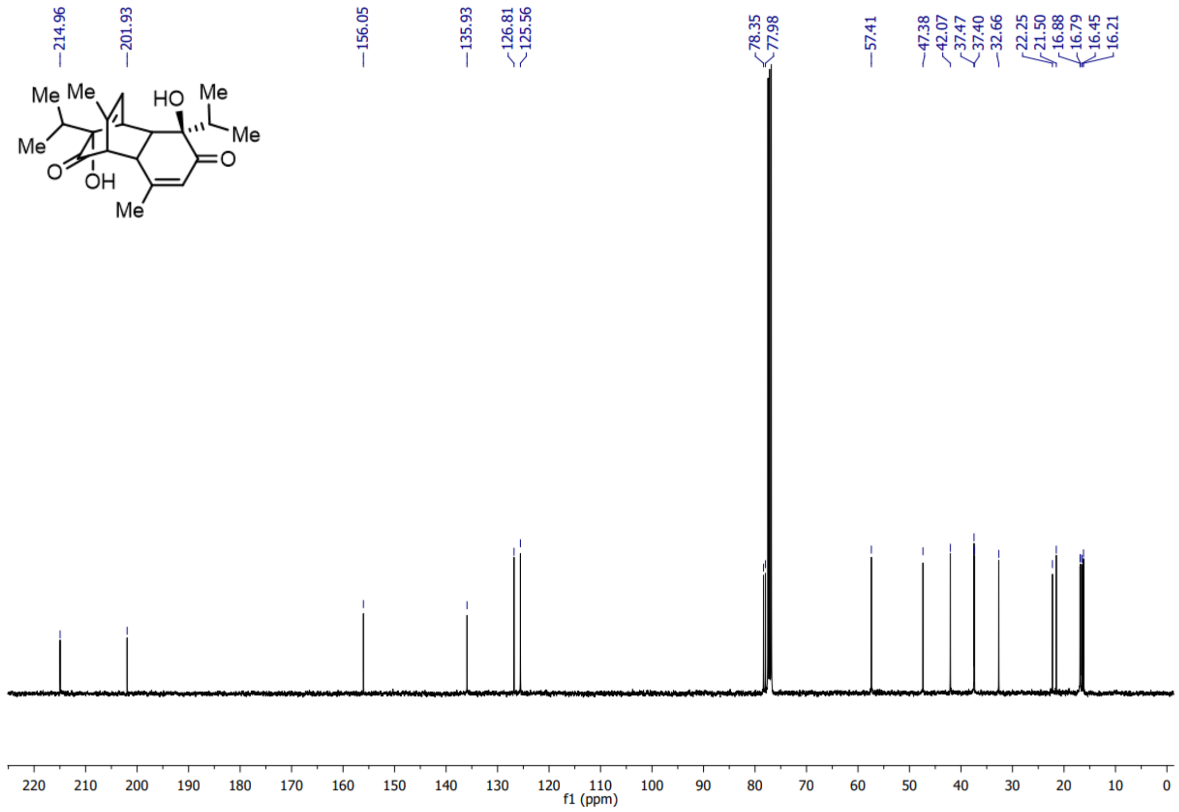
214.96
201.93
156.05
135.93
126.81
125.56
78.35
77.98
57.41
47.38
42.07
37.47
37.40
32.66
22.25
21.50
16.88
16.79
16.45
16.21
Me Me HO Me
Me
Me
O OH O
Me
220 210 200 190 180 170 160 150 140 130 120 110 100 90 80 70 60 50 40 30 20 10 0
f1 (ppm)

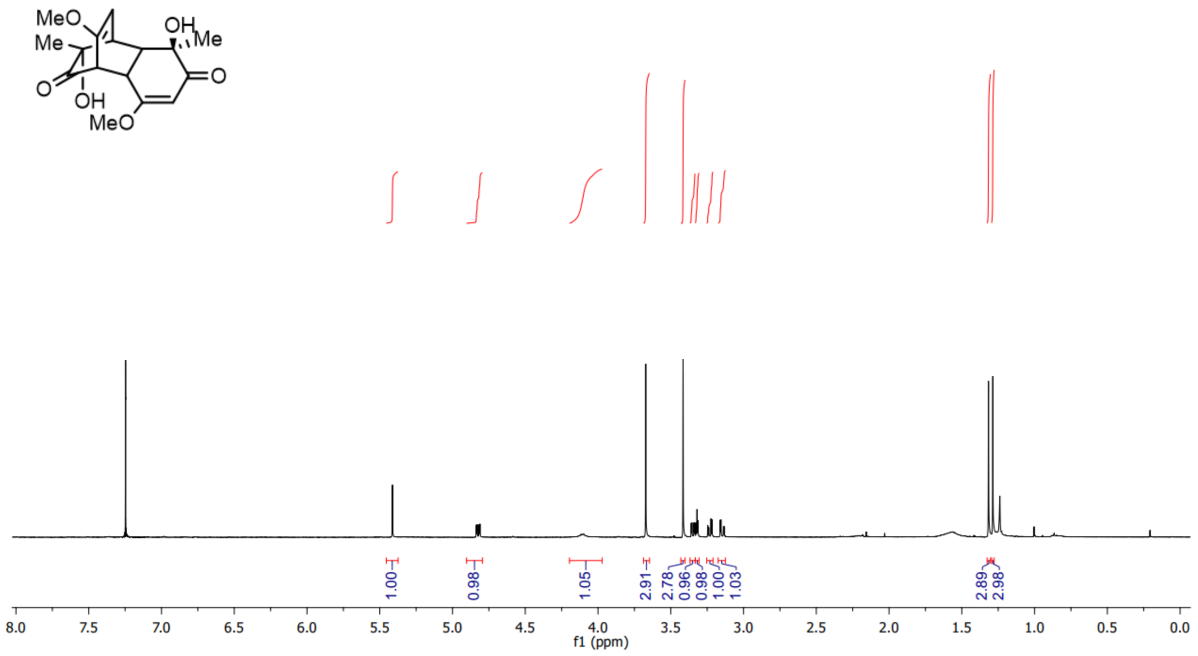
MeO
Me
OH
Me
O
O
OH
MeO
1.00
0.98
1.05
2.91
2.78
0.96
0.98
1.00
1.03
2.89
2.98
8.0 7.5 7.0 6.5 6.0 5.5 5.0 4.5 4.0 3.5 3.0 2.5 2.0 1.5 1.0 0.5 0.0
f1 (ppm)

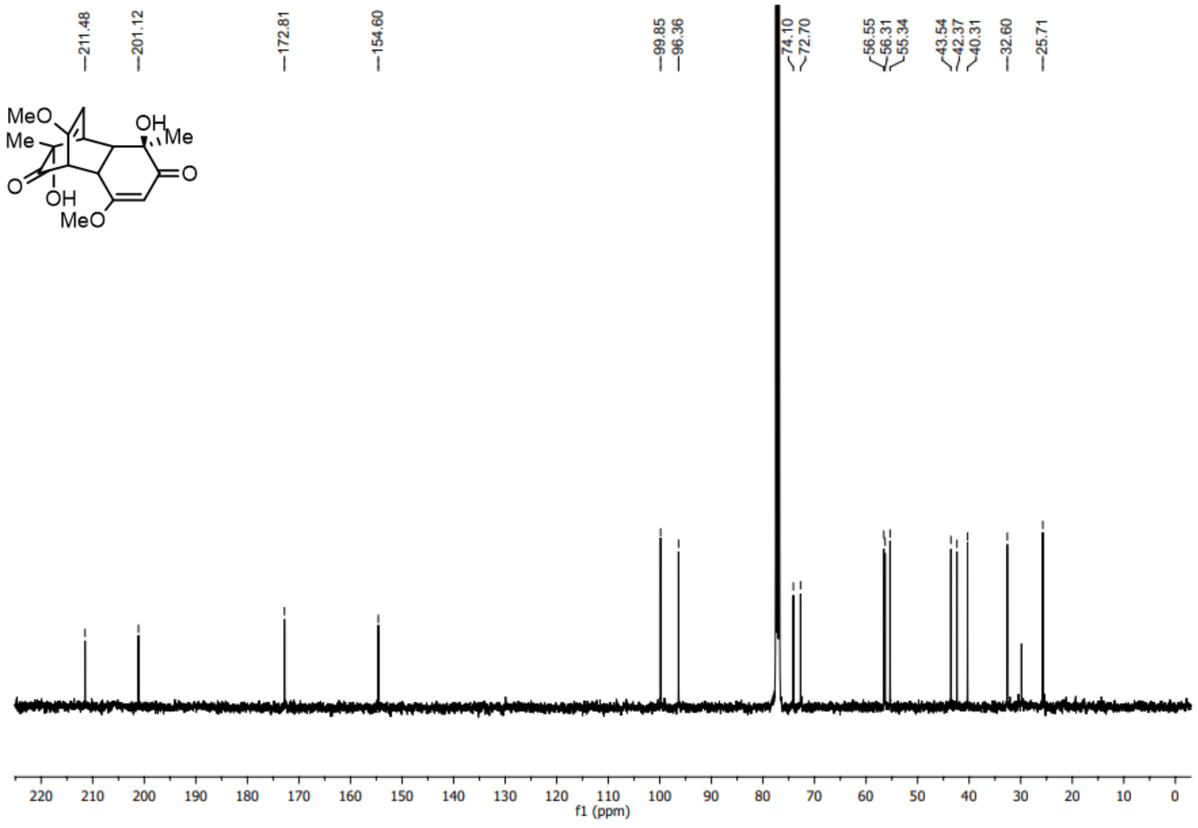
211.48
201.12
172.81
154.60
99.85
96.36
74.10
72.70
56.55
56.31
55.34
43.54
42.37
40.31
32.60
25.71
MeO
Me
OH
Me
O
O
OH
MeO
220 210 200 190 180 170 160 150 140 130 120 110 100 90 80 70 60 50 40 30 20 10 0
f1 (ppm)

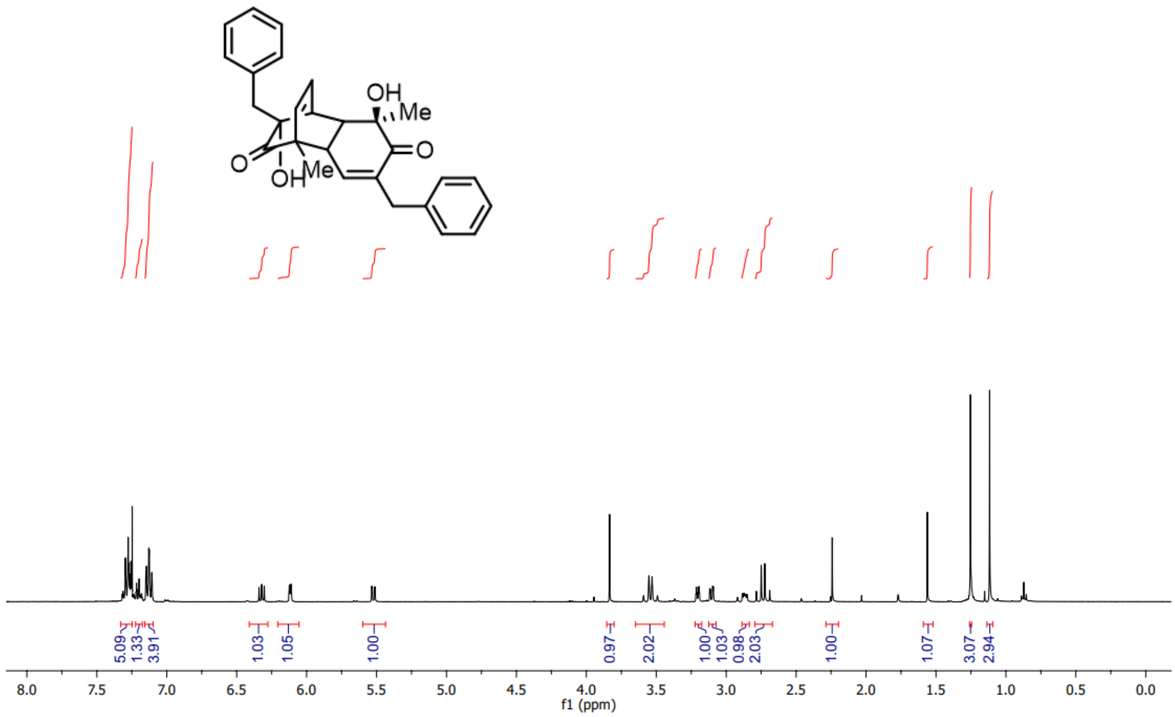
OH
Me
O
O
OH
Me
5.09
1.33
3.91
1.03
1.05
1.00
0.97
2.02
1.00
1.03
0.98
2.03
1.00
1.07
3.07
2.94
8.0
7.5
7.0
6.5
6.0
5.5
5.0
4.5
4.0
3.5
3.0
2.5
2.0
1.5
1.0
0.5
0.0
f1 (ppm)

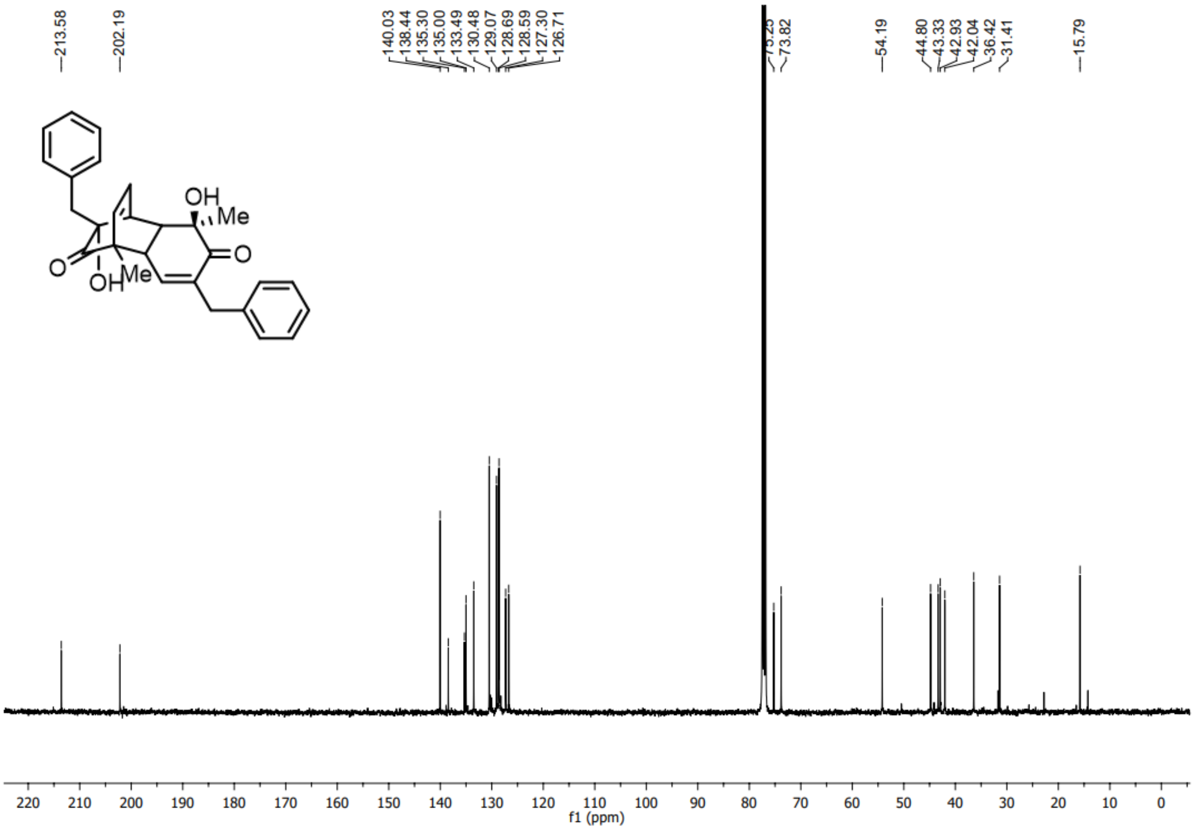
213.58
202.19
140.03
138.44
135.30
135.00
133.49
130.48
129.07
128.69
128.59
127.30
126.71
73.82
54.19
44.80
43.33
42.93
42.04
36.42
31.41
15.79
OH
Me
O
O
OH
Me
220
210
200
190
180
170
160
150
140
130
120
110
100
90
80
70
60
50
40
30
20
10
0
f1 (ppm)

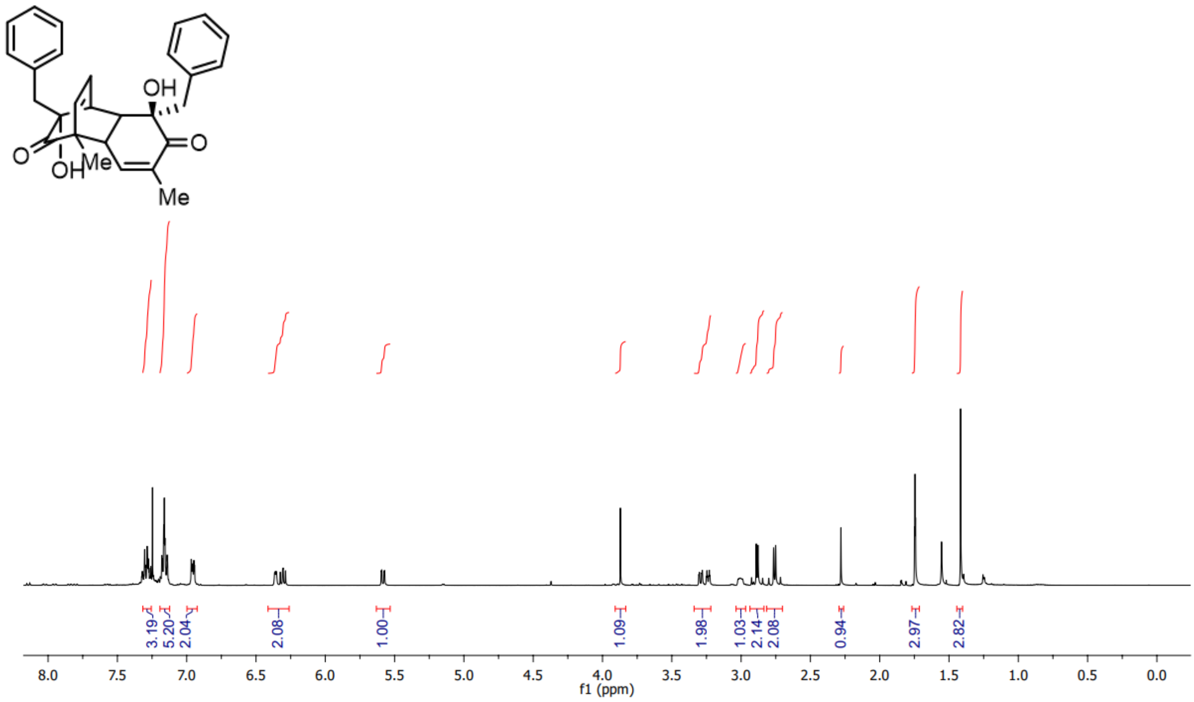
OH
O
OH
Me
O
Me
3.19
5.20
2.04
2.08
1.00
1.09
1.98
1.03
2.14
2.08
0.94
2.97
2.82
8.0 7.5 7.0 6.5 6.0 5.5 5.0 4.5 4.0 3.5 3.0 2.5 2.0 1.5 1.0 0.5 0.0
f1 (ppm)

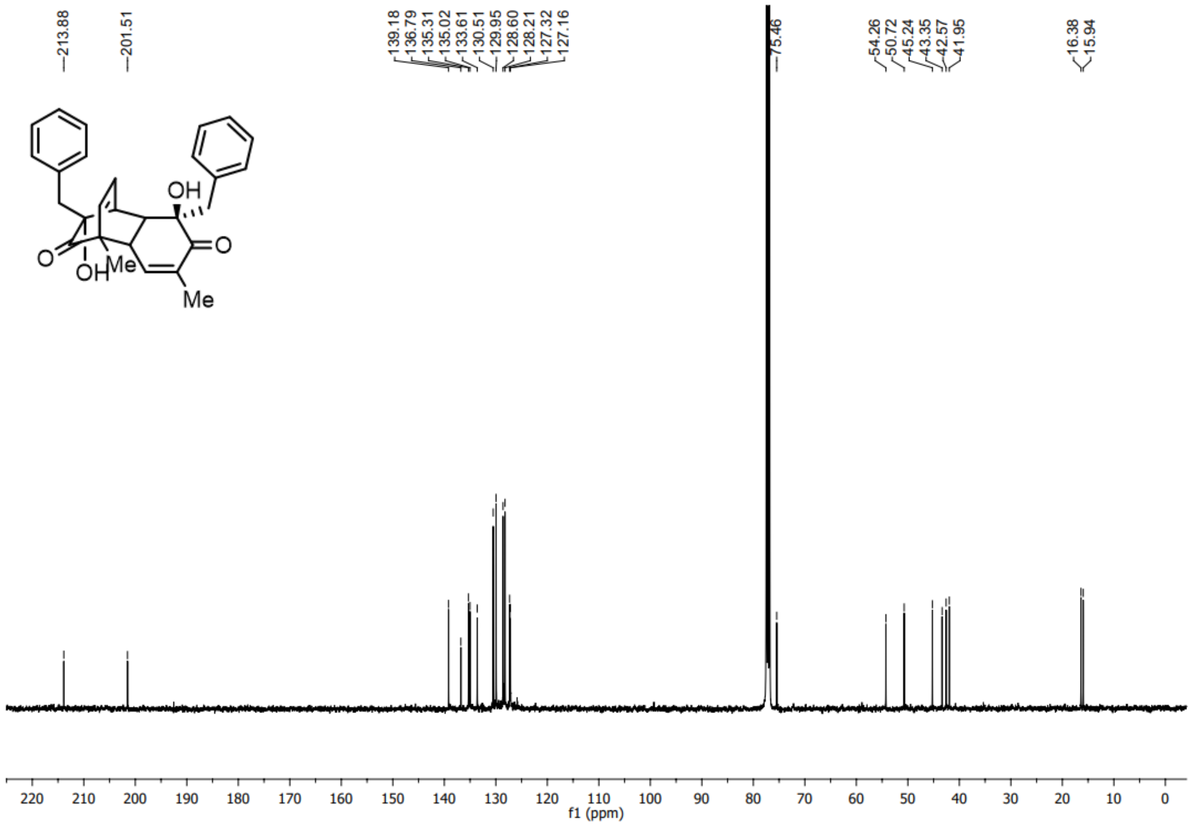
213.88
201.51
139.18
136.79
135.31
135.02
133.61
130.51
129.95
128.60
128.21
127.32
127.16
75.46
54.26
50.72
45.24
43.35
42.57
41.95
16.38
15.94
OH
O
OH
Me
O
Me
220 210 200 190 180 170 160 150 140 130 120 110 100 90 80 70 60 50 40 30 20 10 0
f1 (ppm)

^{1}H COSY

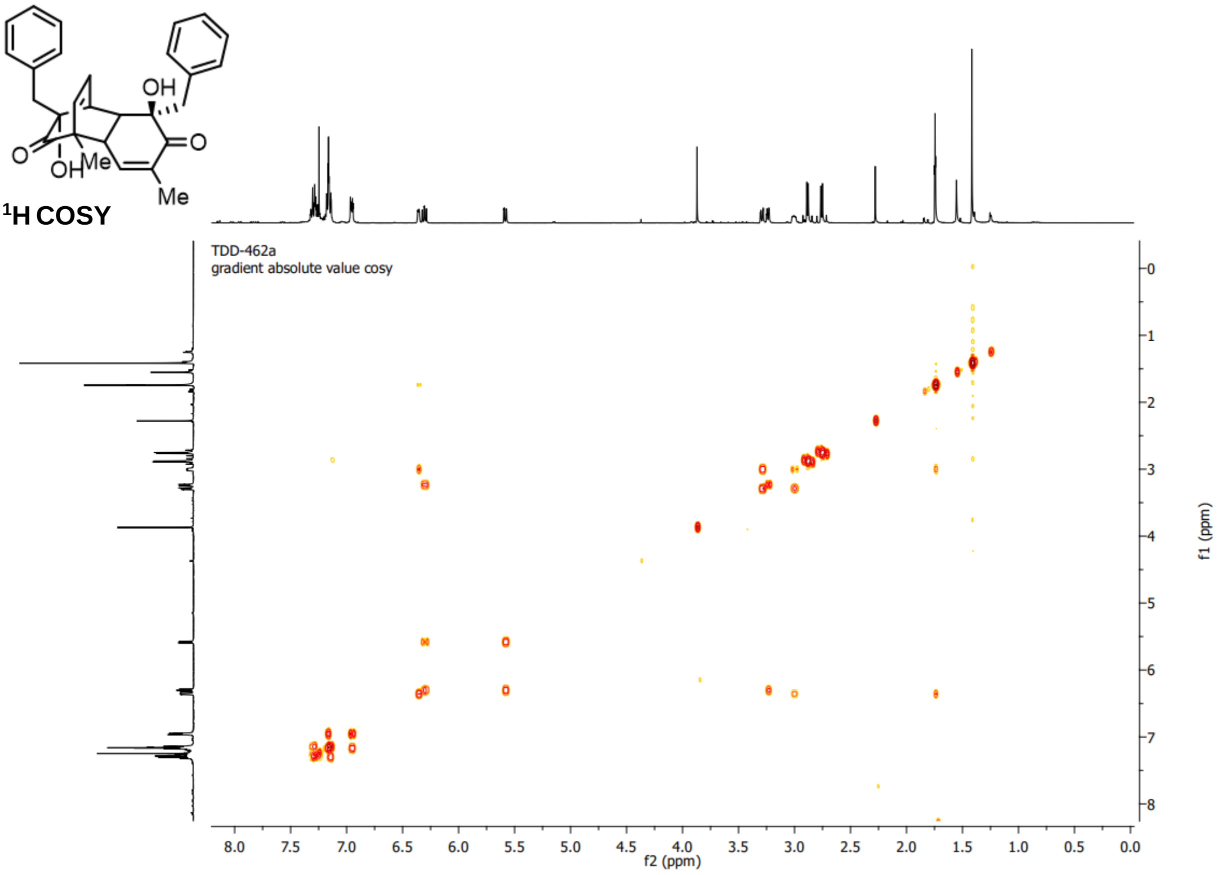
OH
O
O
OH
Me
Me
TDD-462a
gradient absolute value cosy
f2 (ppm)
f1 (ppm)

HMQC

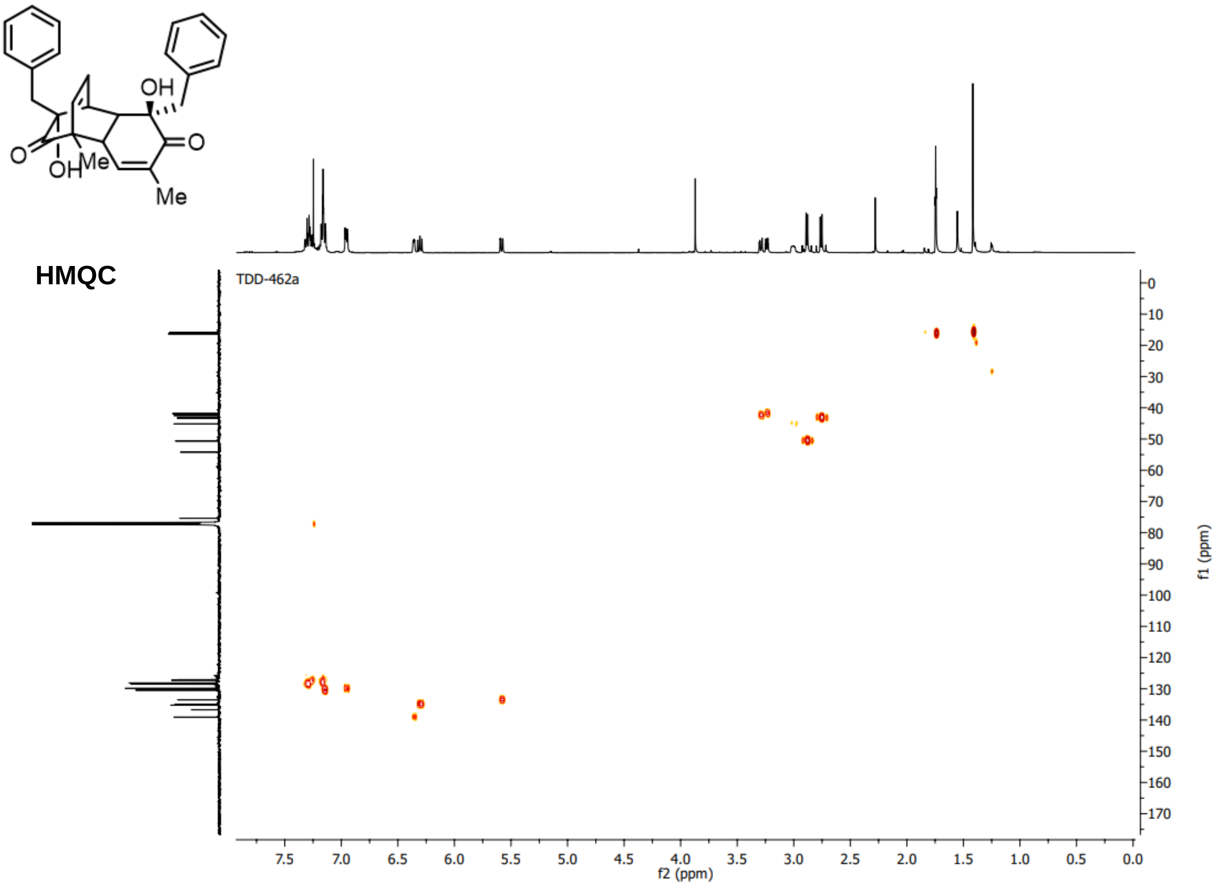
OH
O
O
OH
Me
Me
TDD-462a
f2 (ppm)
f1 (ppm)

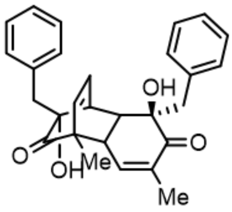

HMBC

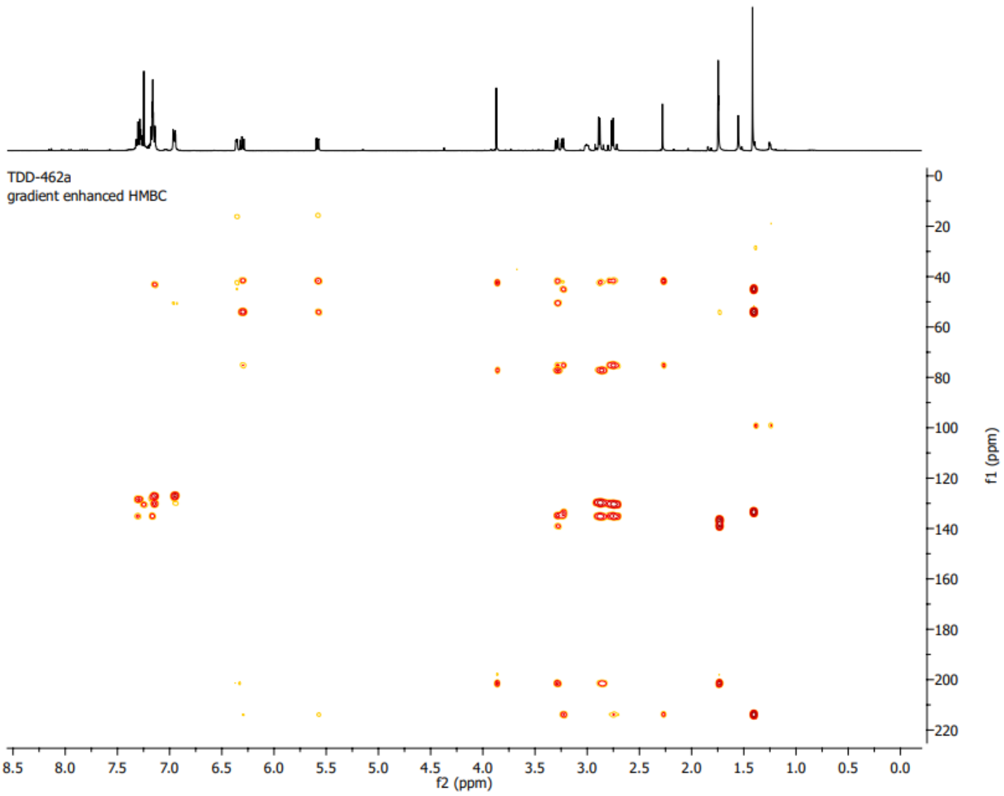

key NOE enhancements

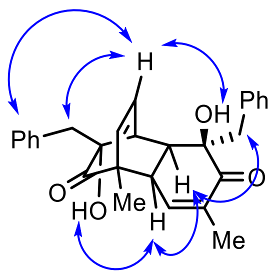

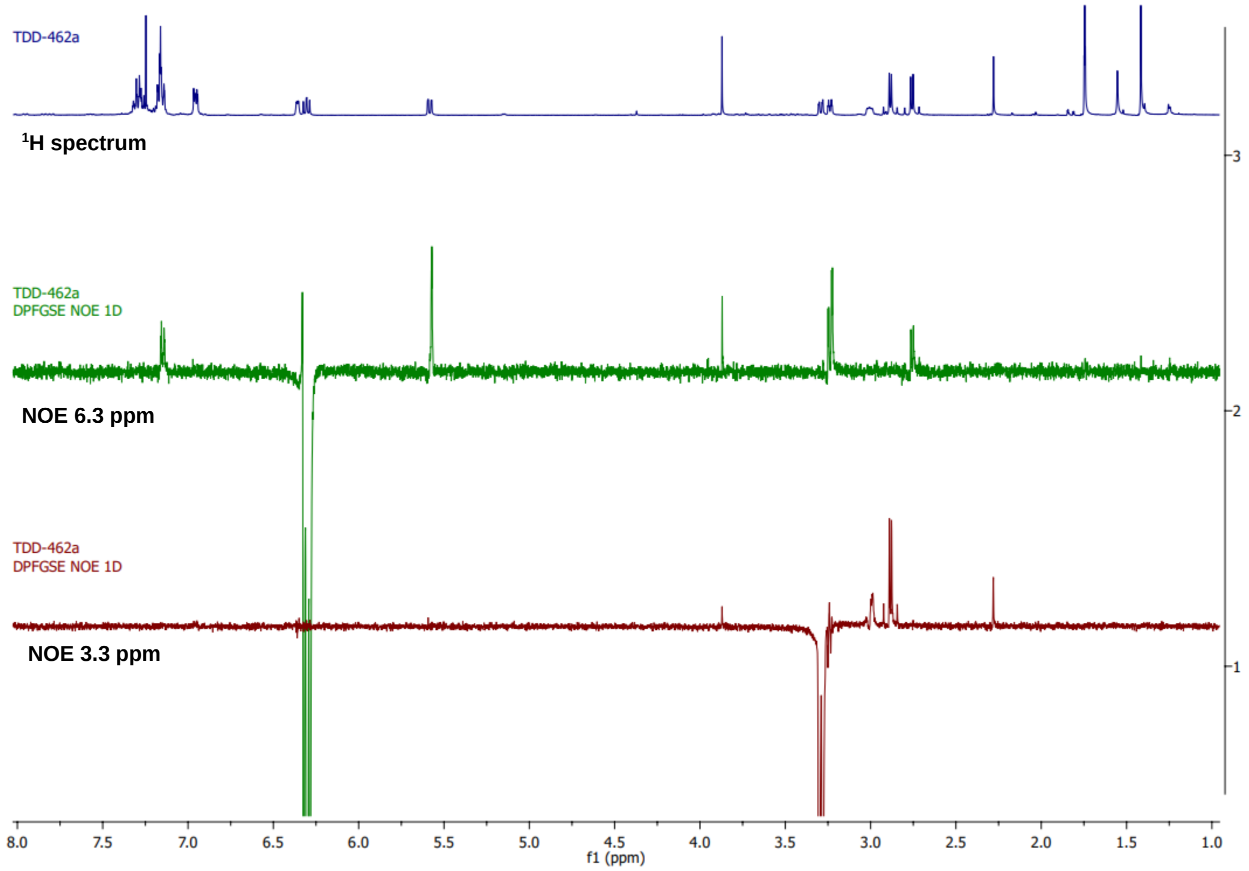

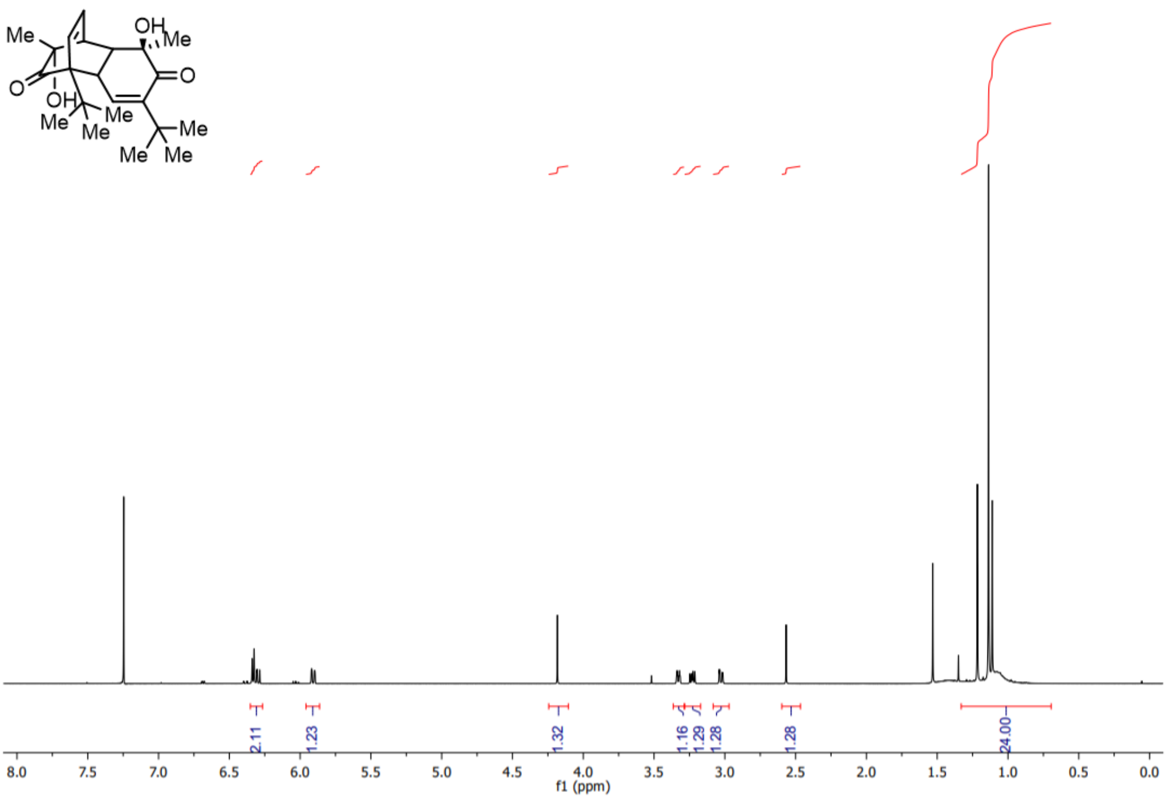
Me
OH
Me
O
O
OH
Me
Me
Me
Me
Me
Me
2.11
1.23
1.32
1.16
1.29
1.28
1.28
24.00
8.0 7.5 7.0 6.5 6.0 5.5 5.0 4.5 4.0 3.5 3.0 2.5 2.0 1.5 1.0 0.5 0.0
f1 (ppm)

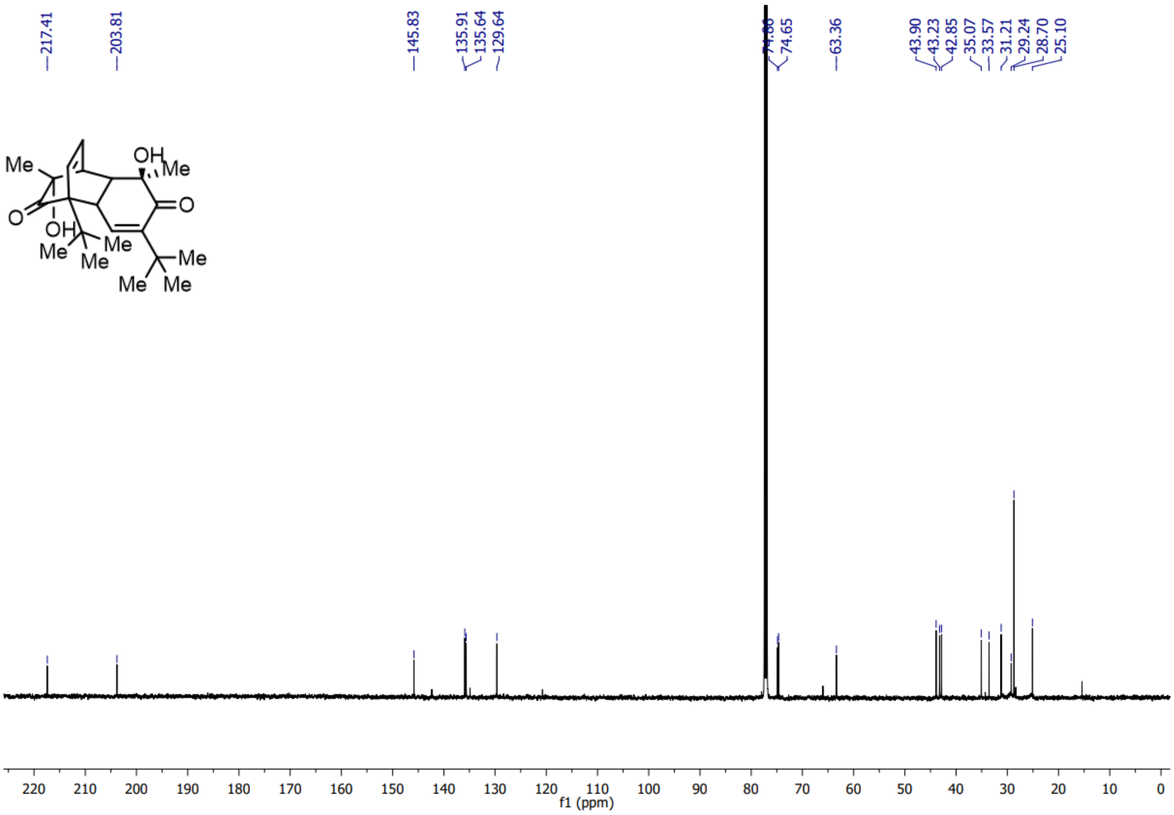
217.41
203.81
145.83
135.91
135.64
129.64
74.65
63.36
43.90
43.23
42.85
35.07
33.57
31.21
29.24
28.70
25.10
220 210 200 190 180 170 160 150 140 130 120 110 100 90 80 70 60 50 40 30 20 10 0
f1 (ppm)

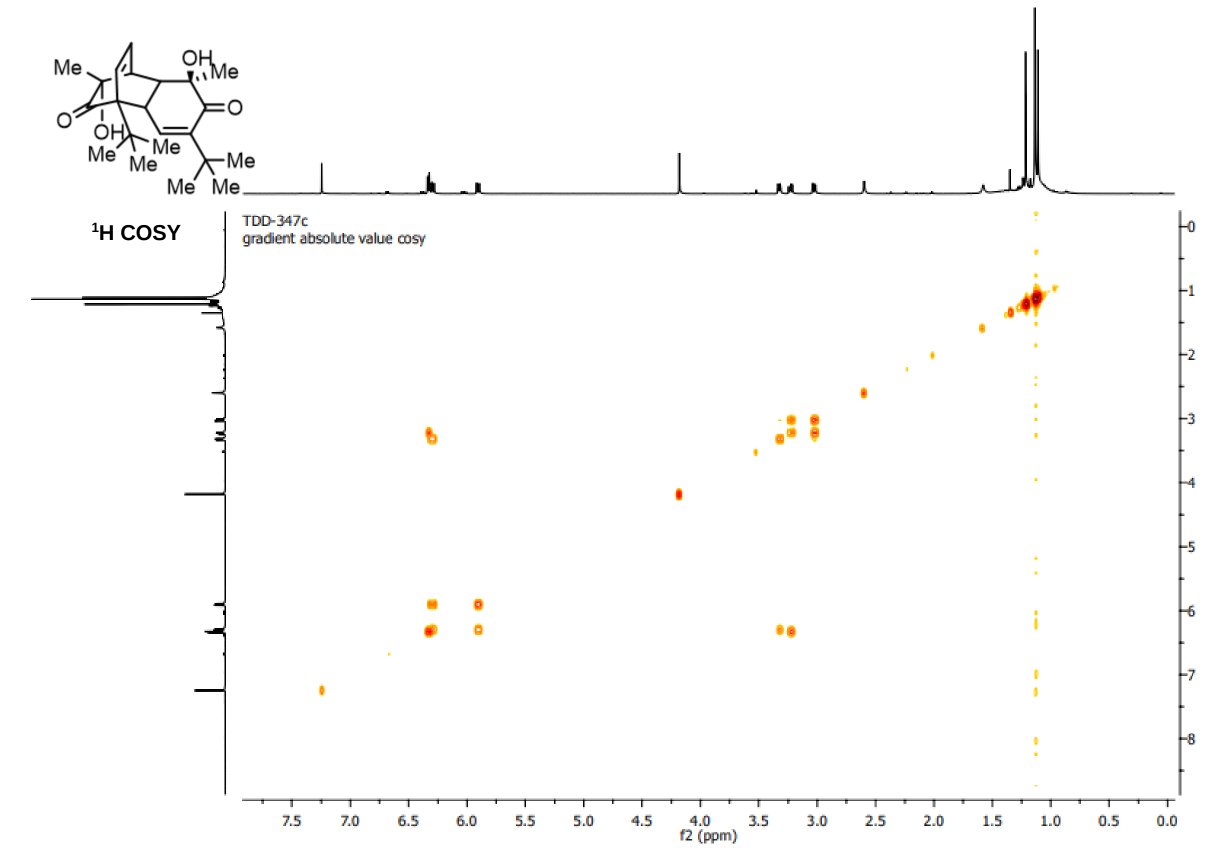
Me
OH
Me
O
OH
Me
Me
Me
O
Me
Me
Me
¹H COSY
TDD-347c
gradient absolute value cosy
f2 (ppm)

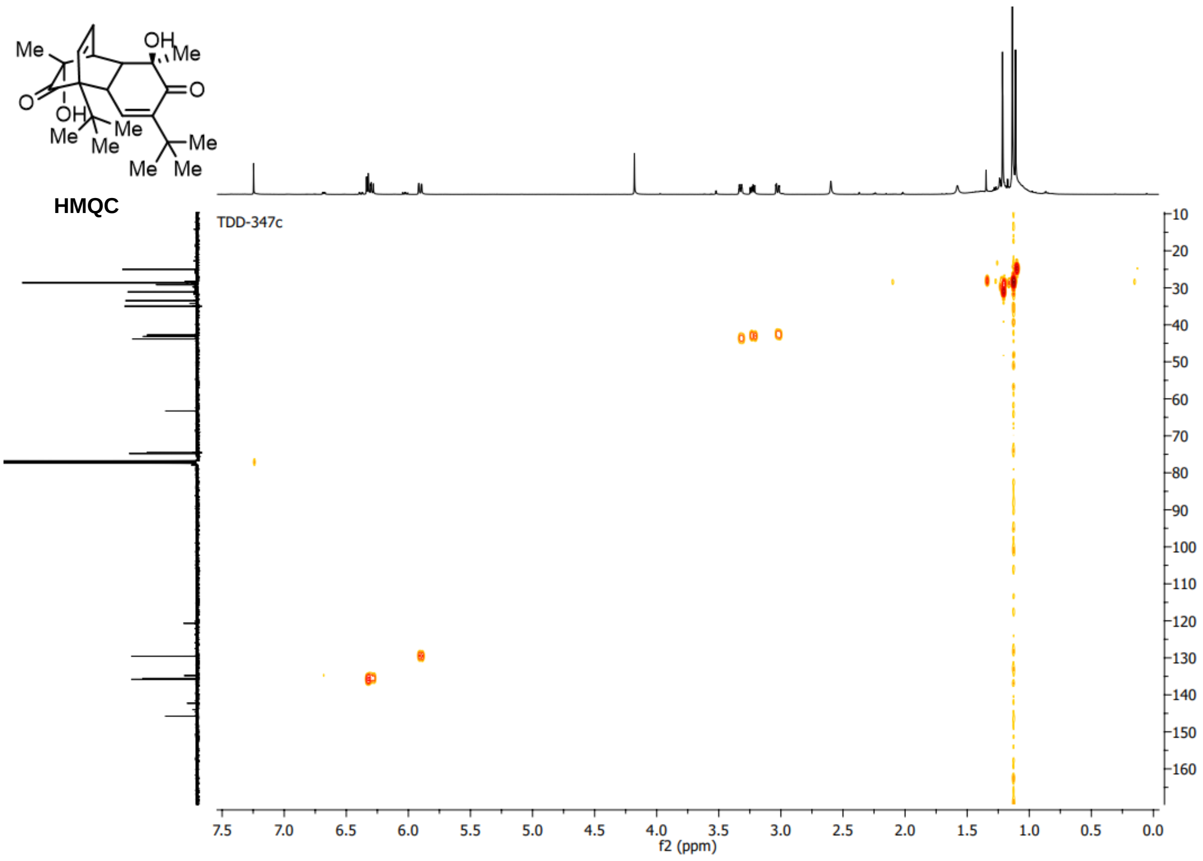
Me
OH
Me
O
OH
Me
Me
Me
O
Me
Me
Me
HMQC
TDD-347c
f2 (ppm)

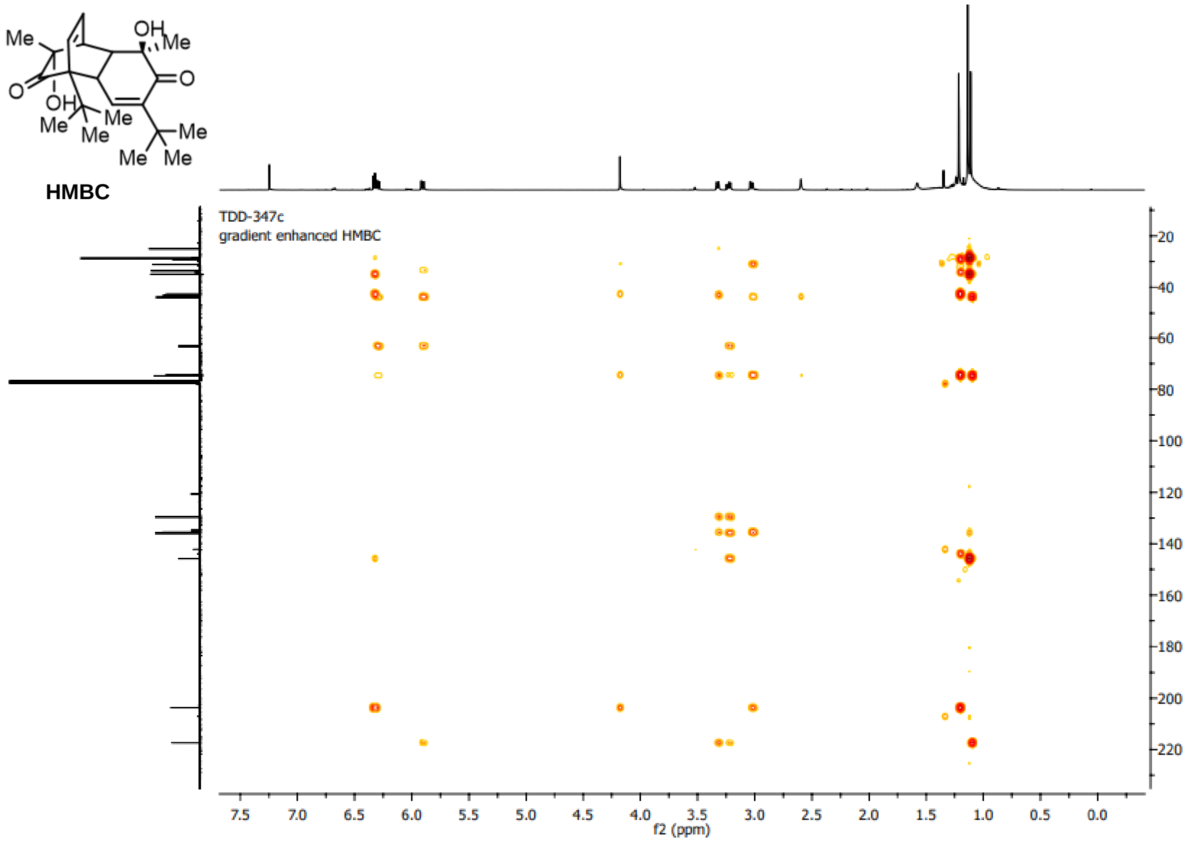
HMBC
TDD-347c
gradient enhanced HMBC
f2 (ppm)

key NOE enhancements

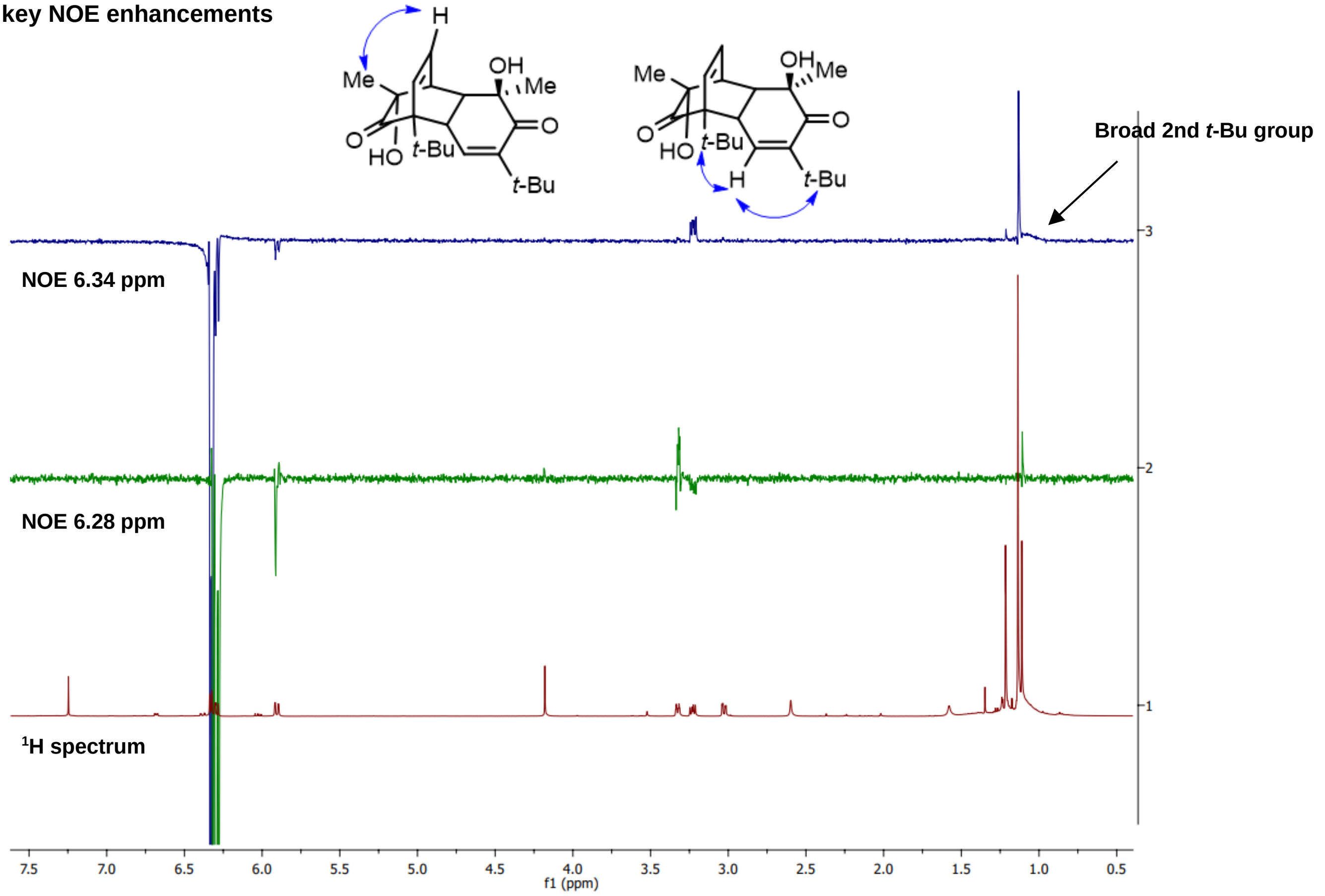

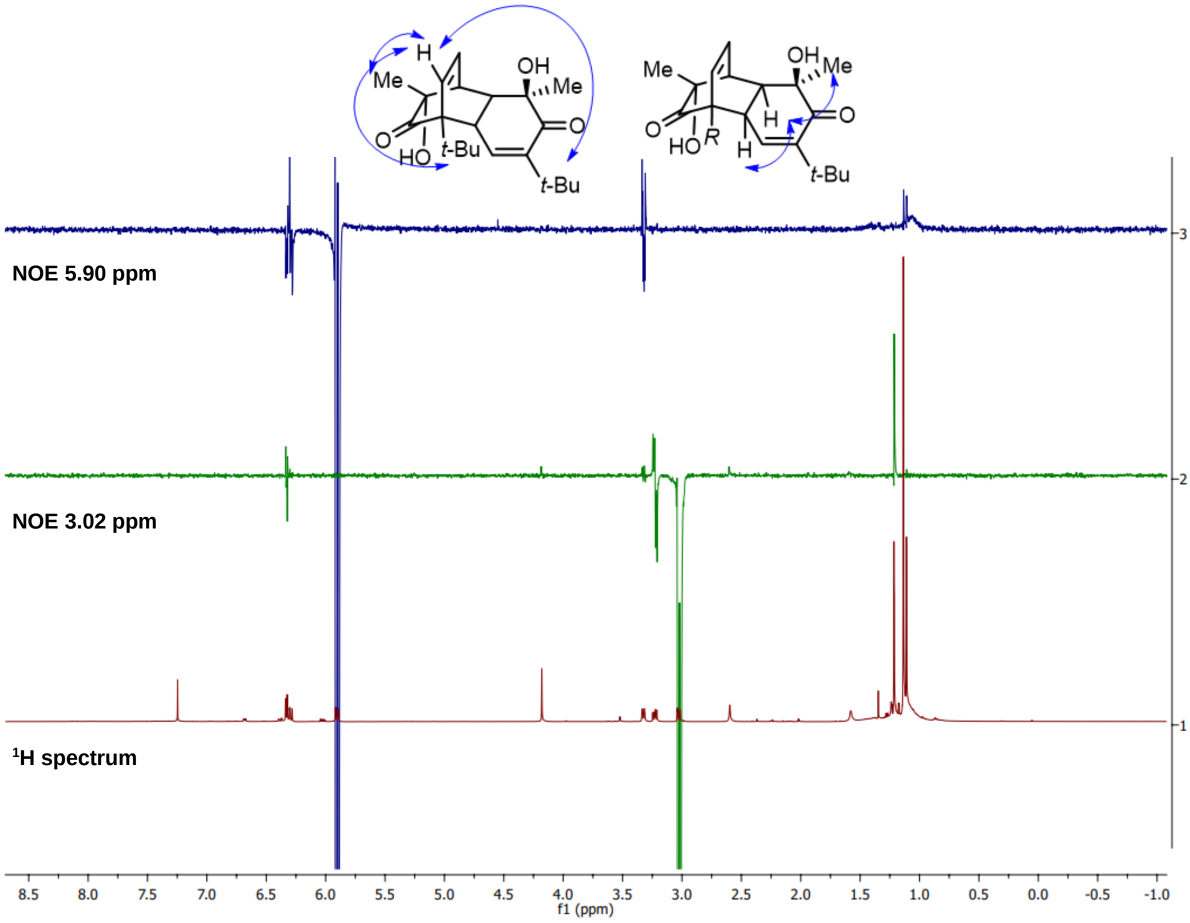

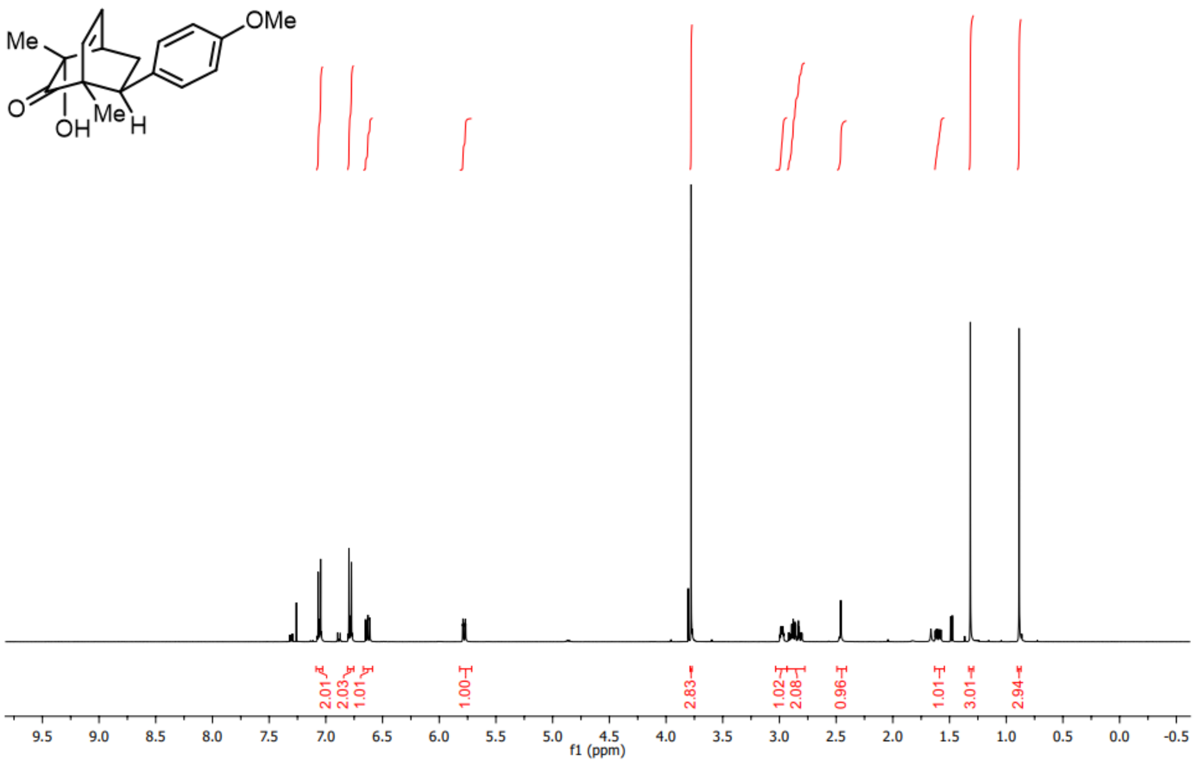
Me
OMe
O
OH
Me
H
2.01
2.03
1.01
1.00
2.83
1.02
2.08
0.96
1.01
3.01
2.94
9.5 9.0 8.5 8.0 7.5 7.0 6.5 6.0 5.5 5.0 4.5 4.0 3.5 3.0 2.5 2.0 1.5 1.0 0.5 0.0 -0.5
f1 (ppm)

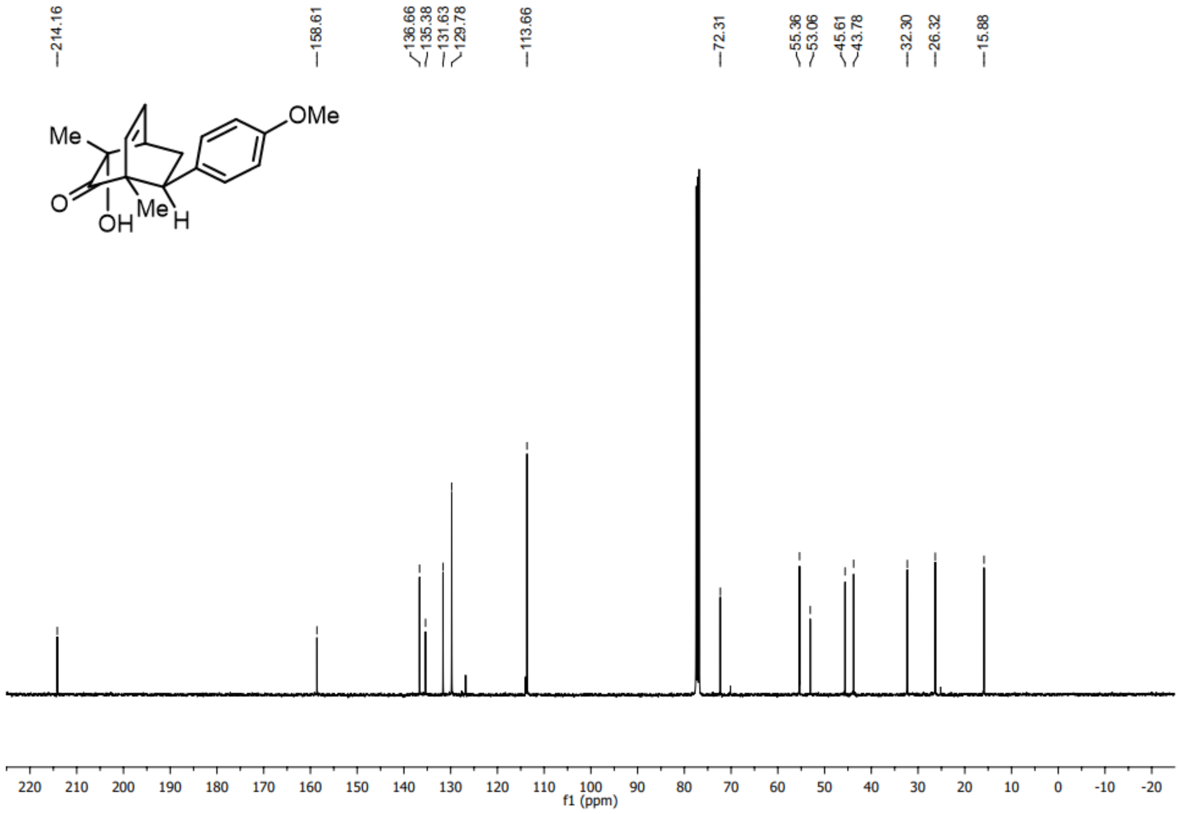
214.16
158.61
136.66
135.38
131.63
129.78
113.66
72.31
55.36
53.06
45.61
43.78
32.30
26.32
15.88
Me
OMe
O
OH
Me
H
220 210 200 190 180 170 160 150 140 130 120 110 100 90 80 70 60 50 40 30 20 10 0 -10 -20
f1 (ppm)

key NOE enhancements

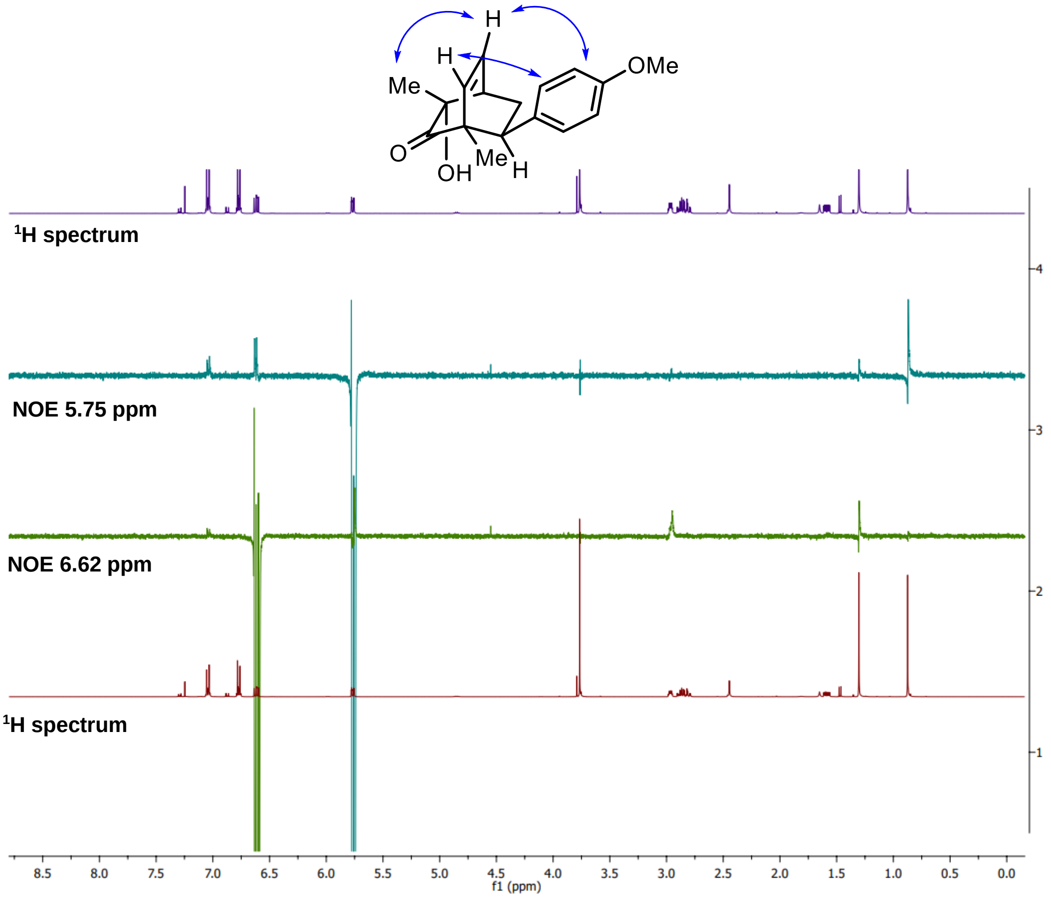

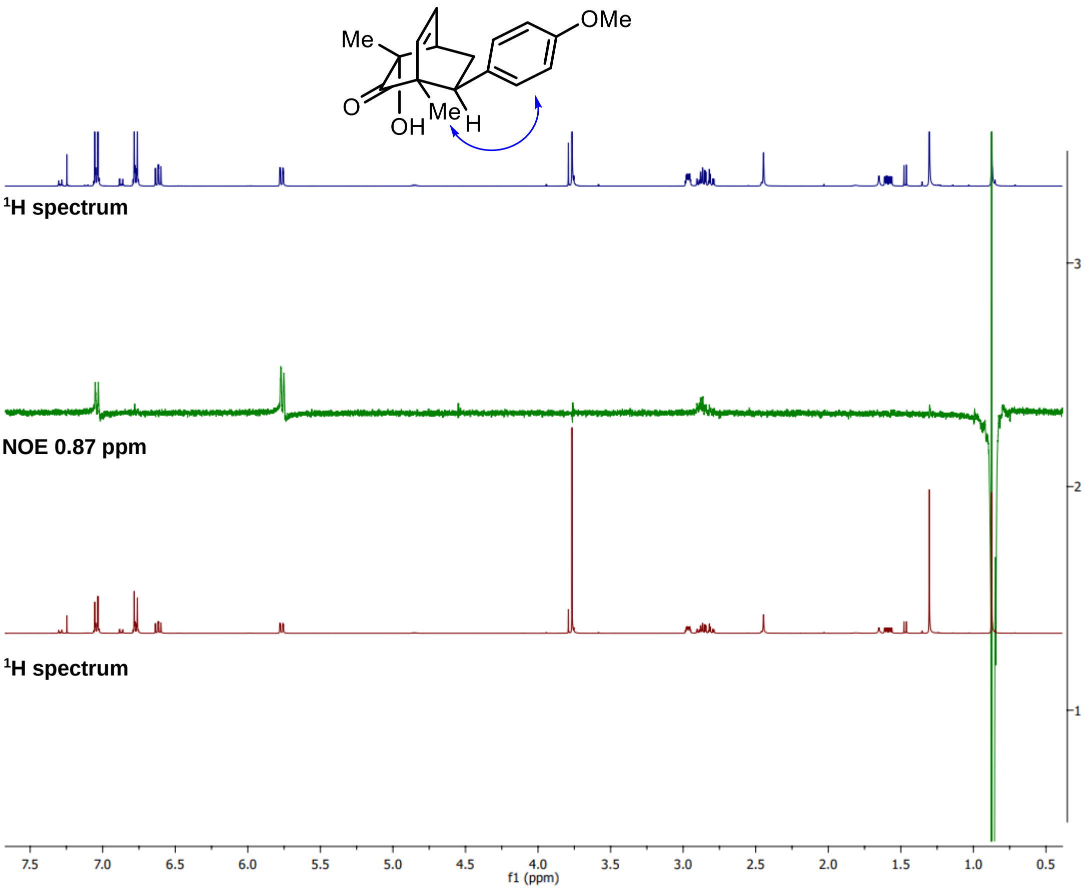

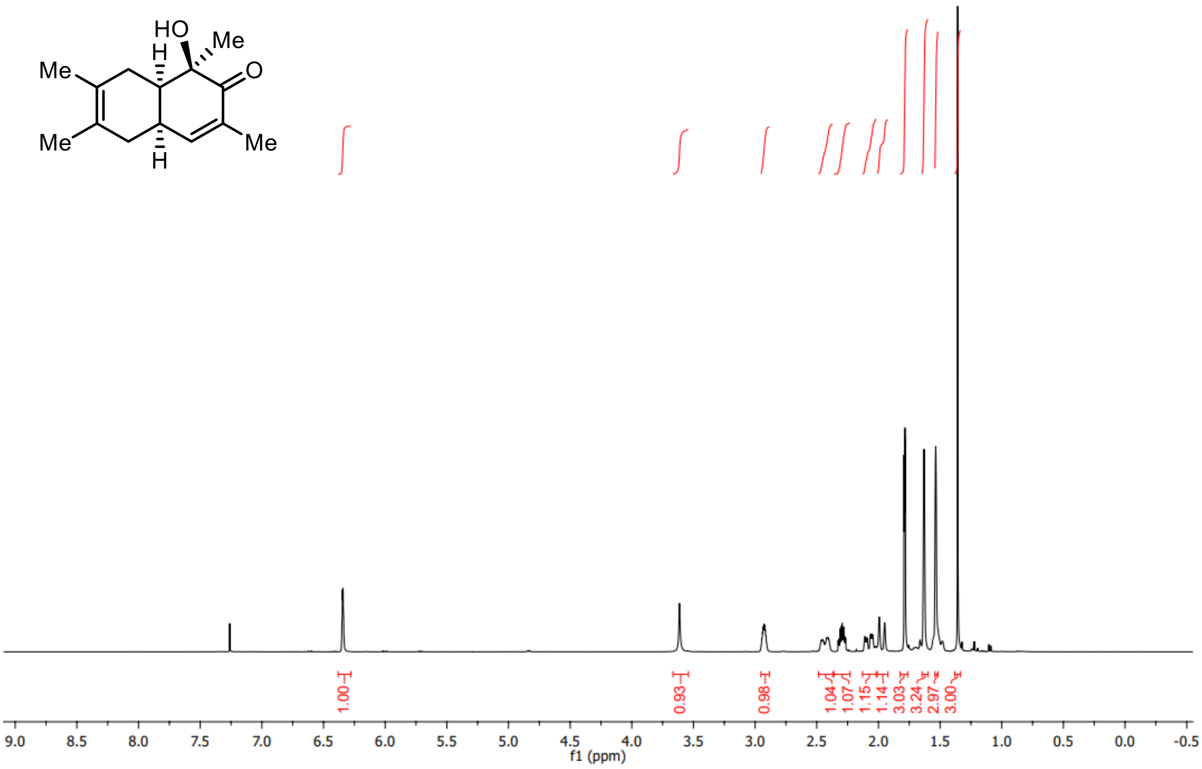
HO
H
Me
O
Me
Me
Me
H
1.00
0.93
0.98
1.04
1.07
1.15
1.14
3.03
3.24
2.97
3.00
9.0 8.5 8.0 7.5 7.0 6.5 6.0 5.5 5.0 4.5 4.0 3.5 3.0 2.5 2.0 1.5 1.0 0.5 0.0 -0.5
f1 (ppm)

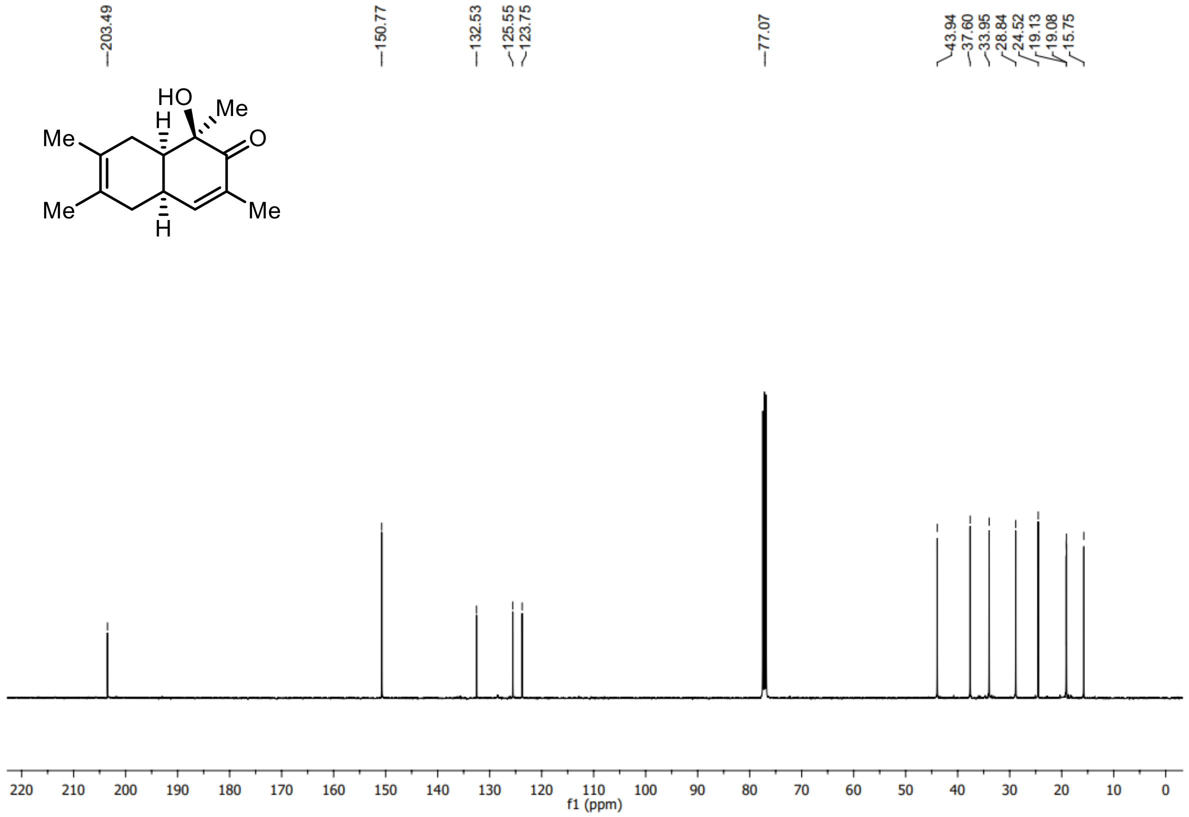
203.49
150.77
132.53
125.55
123.75
77.07
43.94
37.60
33.95
28.84
24.52
19.13
19.08
15.75
HO
H
Me
O
Me
Me
Me
H
220 210 200 190 180 170 160 150 140 130 120 110 100 90 80 70 60 50 40 30 20 10 0
f1 (ppm)

key NOE enhancements

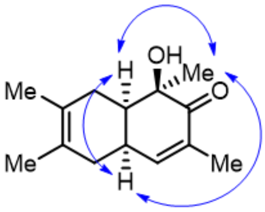

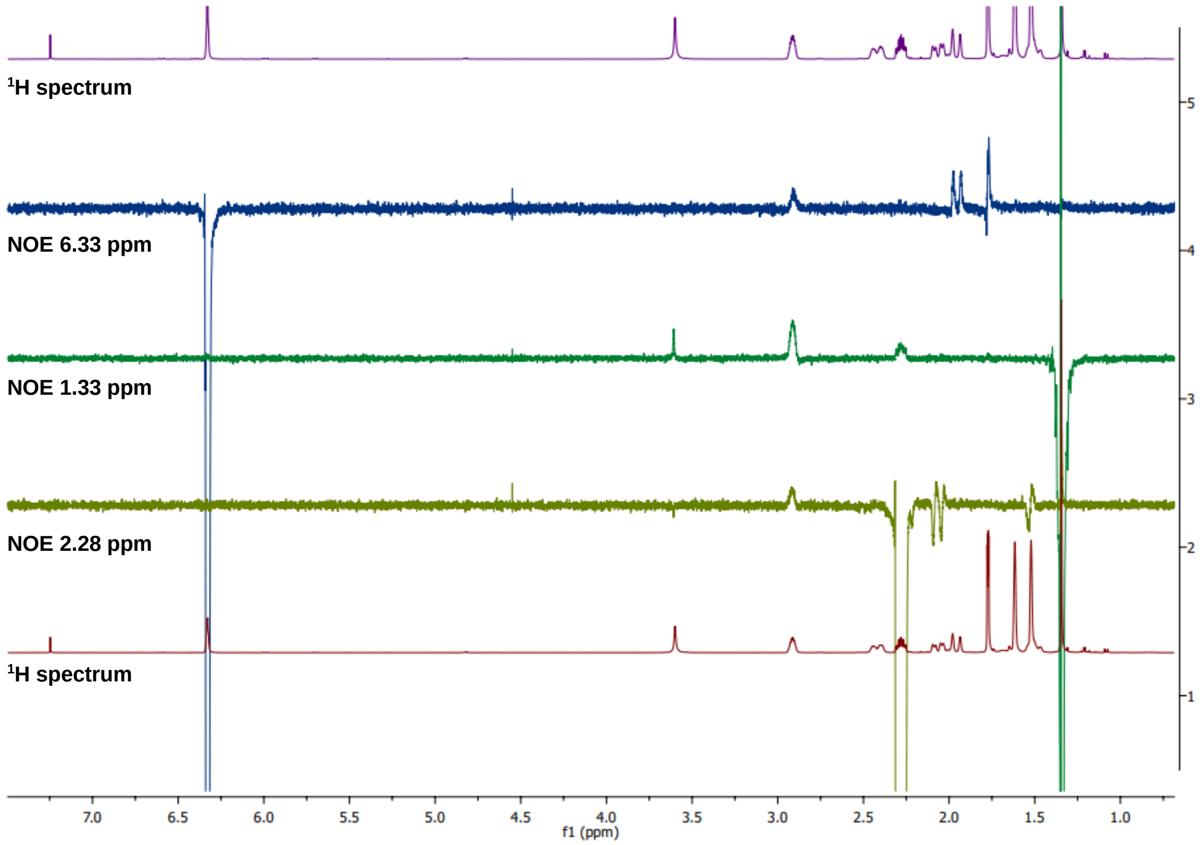

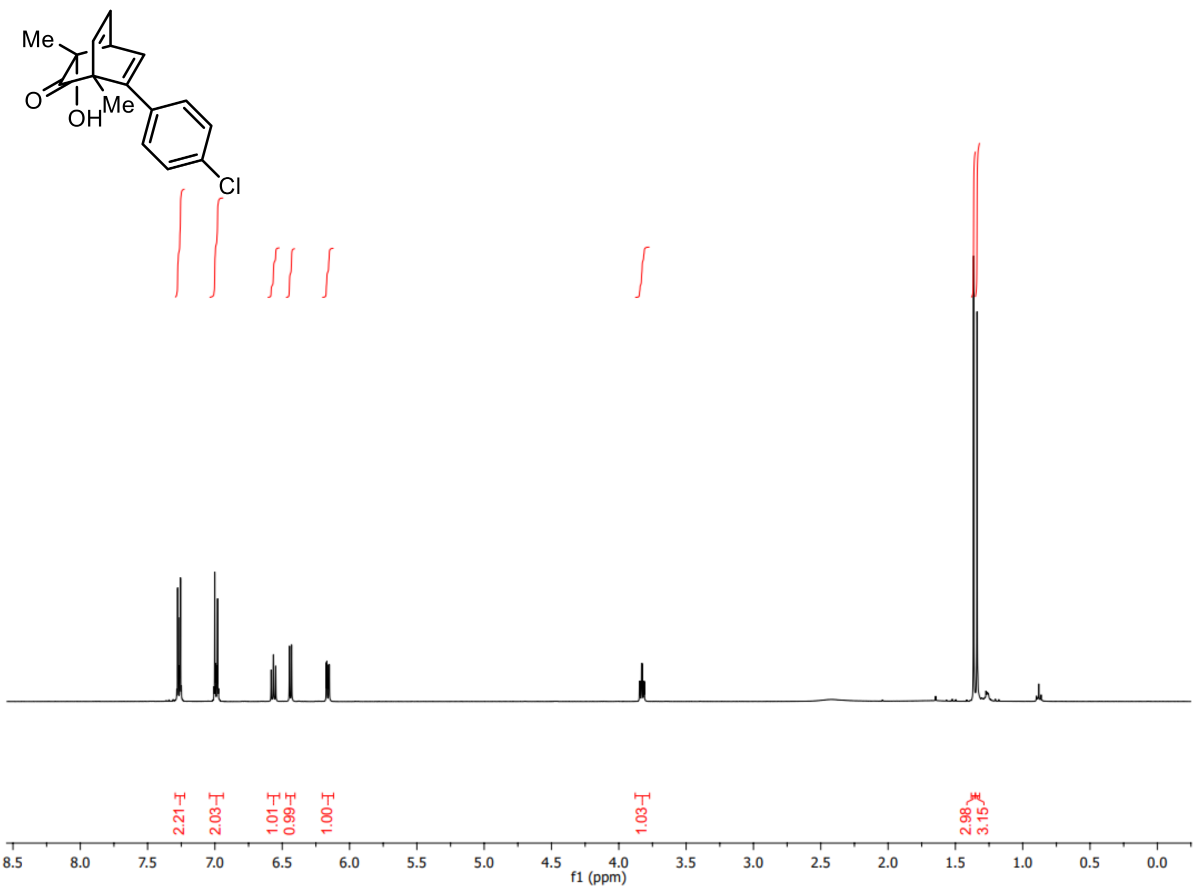
Me
O
OH
Me
Cl
2.21
2.03
1.01
0.99
1.00
1.03
2.98
3.15
f1 (ppm)

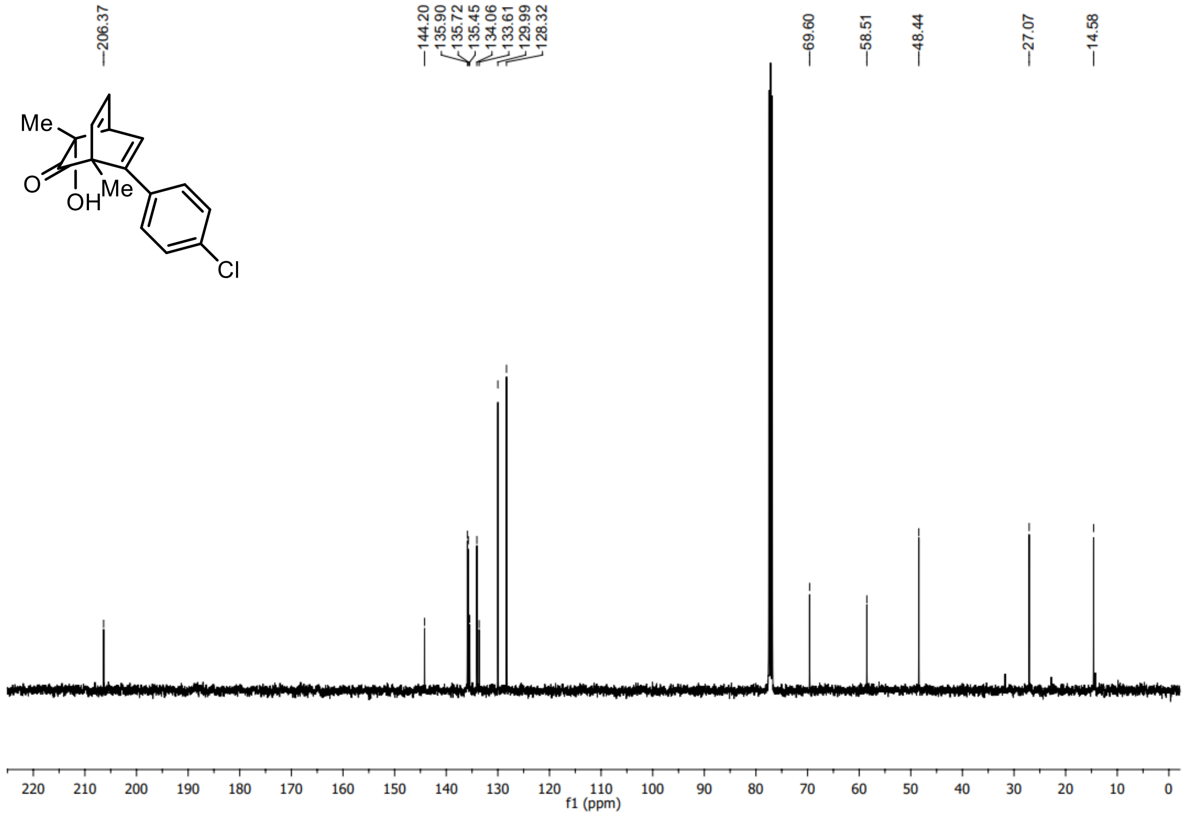
206.37
144.20
135.90
135.72
135.45
134.06
133.61
129.99
128.32
69.60
58.51
48.44
27.07
14.58
Me
O
OH
Me
Cl
f1 (ppm)

References

1. (a) Page, P. C. B.; Rassias, G. A.; Barros, D.; Ardakani, A.; Buckley, B.; Bethell, D.; Smith, T. A. D.; Slawin, A. M. Z. Functionalized Iminium Salt Systems for Catalytic Asymmetric Epoxidation. *J. Org. Chem.* **2001**, *66*, 6926–6931. (b) Bulman Page, P. C.; Buckley, B. R.; Rassias, G. A.; Blacker, A. J. New Chiral Iminium Salt Catalysts for Asymmetric Epoxidation. *European J. Org. Chem.* **2006**, *3*, 803–813.

2. Cervantes-Reyes, A.; Rominger, F.; Rudolph, M.; Hashmi, A. S. K. Gold(I) Complexes Stabilized by Nine- and Ten-Membered N-Heterocyclic Carbene Ligands. *Chem. - A Eur. J.* **2019**, *25*, 11745–11757.

3. Page, P. C. B.; Farah, M. M.; Buckley, B. R.; Chan, Y.; Blacker, A. J. Preparation of C 2-Symmetric Biaryl Bisiminium Salts and Their Use as Organocatalysts for Asymmetric Epoxidation. *Synlett* **2016**, *27*, 126–130.

4. Chen, P. A.; Setthakarn, K.; May, J. A. A Binaphthyl-Based Scaffold for a Chiral Dirhodium(II) Biscarboxylate Ligand with α-Quaternary Carbon Centers. *ACS Catal.* **2017**, *7* (9), 6155–6161.

5. Bulman Page, P. C.; Buckley, B. R.; Blacker, A. J. Iminium Salt Catalysts for Asymmetric Epoxidation: The First High Enantioselectivities. *Org. Lett.* **2004**, *6*, 1543–1546.

6. Gonçalves, M. H.; Martinez, A.; Grass, S.; Page, P. C. B.; Lacour, J. Enantioselective Olefin Epoxidation Using Homologous Amine and Iminium Catalysts-a Direct Comparison. *Tetrahedron Lett.* **2006**, *47*, 5297–5301.

7. Novikov, R.; Bernardinelli, G.; Lacour, J. Enantioselective Olefin Epoxidation Using Axially Chiral Biaryl Azepinium Salts as Catalysts. Rapid in-Situ Screening and Origin of the Stereocontrol. Adv. Synth. Catal. 2008, 350, 1113–1124.

8. Vachon, J.; Lauper, C.; Ditrich, K.; Lacour, J. Enantioselective Olefin Epoxidation Using Novel Biphenyl and Binaphthyl Azepines and Azepinium Salts. *Tetrahedron Asymmetry* **2006**, *17*, 2334–2338..

9. Bulman Page, P. C.; Bartlett, C. J.; Chan, Y.; Day, D.; Parker, P.; Buckley, B. R.; Rassias, G. A.; Slawin, A. M. Z.; Allin, S. M.; Lacour, J.; et al. Asymmetric Epoxidation Using Iminium Salt Organocatalysts Featuring Dynamically Controlled Atropoisomerism. *J. Org. Chem.* **2012**, *77*, 6128–6138.

10. Uto, Y.; Ae, S.; Koyama, D.; Sakakibara, M.; Otomo, N.; Otsuki, M.; Nagasawa, H.; Kirk, K. L.; Hori, H. Artepillin C Isoprenomics: Design and Synthesis of Artepillin C Isoprene Analogues as Lipid Peroxidation Inhibitor Having Low Mitochondrial Toxicity. *Bioorganic Med. Chem.* **2006**, *14*, 5721–5728.

11. Zhang, X.; Beaudry, C. M. Synthesis of Highly Substituted Phenols and Benzenes with Complete Regiochemical Control. *Org. Lett.* **2020**, *22*, 6086–6090.

12. Lebrasseur, N.; Gagnepain, J.; Ozanne-Beaudenon, A.; Léger, J. M.; Quideau, S. Efficient Access to Orthoquinols and Their [4+2] Cyclodimers via SIBX-Mediated Hydroxylative Phenol Dearomatization. *J. Org. Chem.* **2007**, *72*, 6280–6283.

13. Kneijel, B. H.; Poszich-buscher.; Rittich, S.; and Breitmaier, E. C The Diels-Alder Dimer of 6-Hydroxy-2,6-dimethyl-cyclohexa-2,4-dienone, an Unusual Metabolite in the Bacterial Degradation of 2,6-Xylenol. *Angew. Chem., Int. Ed.*, **1991**, *2*, 201–203.

14. Carman, R. M.; Lambert, L. K.; Robinson, W. T.; and Van Dongen, J. M. 3,10-Dihydroxydielmentha-5,11-diene-4,9-dione. A Diterpenoid (Bismonoterpenoid?) with a Novel Carbon Skeleton. *Aust. J. Chem.,* **1986**, *39,* 1843-50.

15. Dong, S.; Zhu, J.; Porco, J. A. Enantioselective Synthesis of Bicyclo[2.2.2]Octenones Using a Copper-Mediated Oxidative Dearomatization/[4 + 2] Dimerization Cascade. *J. Am. Chem. Soc.* **2008**, *130*, 2738–2739.

16. Grandclaudon, C.; Toullec, P. Y. Phase-Transfer-Catalyzed Oxaziridine-Mediated Hydroxylative Phenol and Naphthol Dearomatization. *European J. Org. Chem.* **2016**, *2*, 260–264.

17. Darras, F. H.; Kling, B.; Heilmann, J.; Decker, M. Neuroprotective Tri- and Tetracyclic BChE Inhibitors Releasing Reversible Inhibitors upon Carbamate Transfer. *ACS Med. Chem. Lett.* **2012**, *3*, 914–919.

18. Bosset, C.; Coffinier, R.; Peixoto, P. A.; El Assal, M.; Miqueu, K.; Sotiropoulos, J.-M.; Pouységu, L.; Quideau, S. Asymmetric Hydroxylative Phenol Dearomatization Promoted by Chiral Binaphthylic and Biphenylic Iodanes. *Angew. Chem., Int. Ed.* **2014**, *126*, 10018–10022.

19. Hanquet, G. M.; Lusinchi, X.; Milliet, P. Action de l’acide Paranitroperbenzoique Sur Le Tetrafluoroborate de n-Methyl-3,4-Dihydroisoquinolinium. Formation d’un Sel d’oxaziridinium. *Tetrahedron* **1993**, *49*, 423–438.
